# Supplementary material for: Central Sensitisation and functioning in patients with chronic low back pain: protocol for a cross-sectional and cohort study
Source: BMJ Open. 2020 Mar 8;10(3):e031592. doi: 10.1136/bmjopen-2019-031592 (PMC7064083; doi:10.1136/bmjopen-2019-031592)

SUPPLEMENTARY FILE

Supplementary material 1. Description of the locations for the QST assessment.

Measuring Location	Description
**1. m. Deltoid – Training location**	In the pars medialis of m. Deltoid, at the contralateral side of the body to the most painful area located; 4 cm below the Acromion.<br>For the CPM test the measurement is at the non-dominant m. Deltoid.
**2. Most painful area**	As stated by the patient. This location is thoroughly described in the form to repeat the measurement in the same exact location at discharge.
**3. Low back – Control location**	Contralateral side of the body mirror-wise to the most painful area located. A specific description of the most painful area is important to identify this location as well.
**4. m. Trapezius – Control location**	In the pars medialis of m. Trapezius, both ipsilateral and contralateral to the most painful area located. At the level of the spine of the Scapula (T3); 4 cm lateral / external to the backbone.
**5. m. Quadriceps – Control location**	In the rectus femoris of m. Quadriceps, both ipsilateral and contralateral to the most painful area located; 15 cm above the upper edge of the Patella.<br>For the CPM test the measurement is at the non-dominant m. Quadriceps.

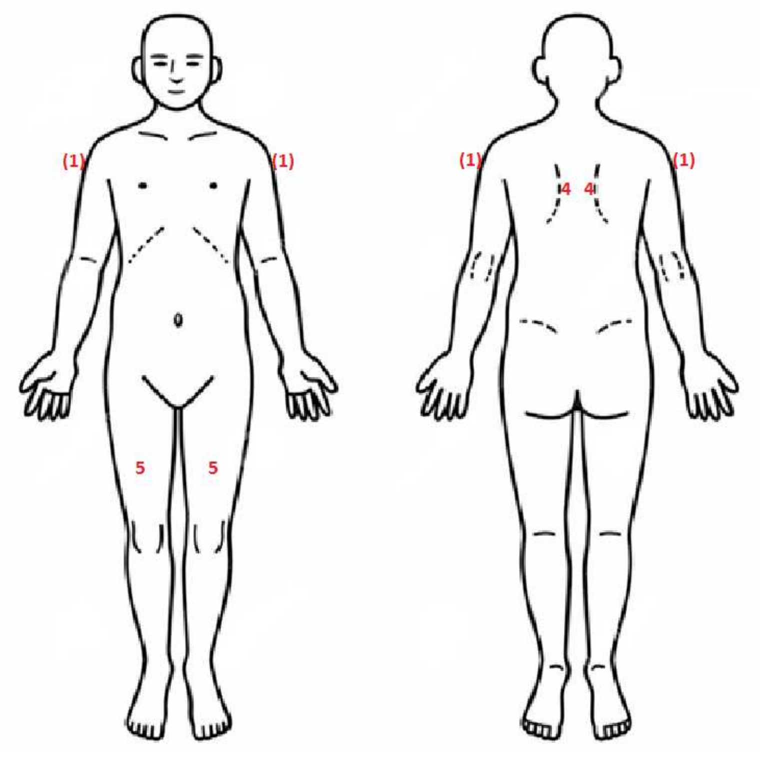

Supplement: Supplementary data [file bmjopen-2019-031592supp001.pdf]
