# Supplementary material for: Central Sensitisation and functioning in patients with chronic low back pain: protocol for a cross-sectional and cohort study
Source: BMJ Open. 2020 Mar 8;10(3):e031592. doi: 10.1136/bmjopen-2019-031592 (PMC7064083; doi:10.1136/bmjopen-2019-031592)
Supplement: Author's manuscript [file bmjopen-2019-031592.draft_revisions.pdf]

BMJ Open is committed to open peer review. As part of this commitment we make the peer review history of every article we publish publicly available.

When an article is published we post the peer reviewers' comments and the authors' responses online. We also post the versions of the paper that were used during peer review. These are the versions that the peer review comments apply to.

The versions of the paper that follow are the versions that were submitted during the peer review process. They are not the versions of record or the final published versions. They should not be cited or distributed as the published version of this manuscript.

BMJ Open is an open access journal and the full, final, typeset and author-corrected version of record of the manuscript is available on our site with no access controls, subscription charges or pay-per-view fees (http://bmjopen.bmj.com).

If you have any questions on BMJ Open's open peer review process please email info.bmjopen@bmj.com

BMJ Open

Central Sensitization and Functioning in patients with Chronic Low Back Pain: Protocol for a cross-sectional and cohort study

Journal:	*BMJ Open*
Manuscript ID	bmjopen-2019-031592
Article Type:	Protocol
Date Submitted by the Author:	10-May-2019
Complete List of Authors:	Ansuategui Echeita, Jone; University Medical Center Groningen, Rehabilitation Medicine<br>Schiphorst Preuper, Henrica ; University Medical Center Groningen, Rehabilitation Medicine<br>Dekker, Rienk; University Medical Center Groningen, Rehabilitation Medicine; University Medical Center Groningen, Center for Sports Medicine<br>Stuive, Ilse; University Medical Center Groningen , Rehabilitation Medicine<br>Timmerman, Hans; Radboud University Medical Center, Department of Anesthesiology, Pain and Palliative Medicine<br>Wolff, Andre; University Medical Center Groningen, Anesthesiology Pain Center<br>Reneman, Michiel; University Medical Center Groningen, Rehabilitation Medicine
Keywords:	Hyperalgesia, Autonomic Nervous System, Lifting, Exercise Test, Physical Activity, Disability

SCHOLARONE™
Manuscripts

I, the Submitting Author has the right to grant and does grant on behalf of all authors of the Work (as defined in the below author licence), an exclusive licence and/or a non-exclusive licence for contributions from authors who are: i) UK Crown employees; ii) where BMJ has agreed a CC-BY licence shall apply, and/or iii) in accordance with the terms applicable for US Federal Government officers or employees acting as part of their official duties; on a worldwide, perpetual, irrevocable, royalty-free basis to BMJ Publishing Group Ltd ("BMJ") its licensees and where the relevant Journal is co-owned by BMJ to the co-owners of the Journal, to publish the Work in this journal and any other BMJ products and to exploit all rights, as set out in our licence.

The Submitting Author accepts and understands that any supply made under these terms is made by BMJ to the Submitting Author unless you are acting as an employee on behalf of your employer or a postgraduate student of an affiliated institution which is paying any applicable article publishing charge ("APC") for Open Access articles. Where the Submitting Author wishes to make the Work available on an Open Access basis (and intends to pay the relevant APC), the terms of reuse of such Open Access shall be governed by a Creative Commons licence – details of these licences and which Creative Commons licence will apply to this Work are set out in our licence referred to above.

Other than as permitted in any relevant BMJ Author's Self Archiving Policies, I confirm this Work has not been accepted for publication elsewhere, is not being considered for publication elsewhere and does not duplicate material already published. I confirm all authors consent to publication of this Work and authorise the granting of this licence.

For peer review only

Central Sensitization and Functioning in patients with Chronic Low Back Pain: Protocol for a cross-sectional and cohort study

J. Ansuategui Echeita [1], H.R. Schiphorst Preuper [1], R. Dekker [1], I. Stuive [1], H. Timmerman [2, 3], A.P. Wolff [3], M.F. Reneman [1].

Affiliations:

[1] Department of Rehabilitation Medicine, University of Groningen, University Medical Center Groningen, Groningen, The Netherlands.

[2] Department of Anesthesiology, Pain and Palliative Medicine, Radboud University Medical Center, Nijmegen, The Netherlands.

[3] Department of Anesthesiology Pain Center, University of Groningen, University Medical Center Groningen, Groningen, The Netherlands.

Corresponding author: Jone Ansuategui Echeita, Department of Rehabilitation Medicine, University Medical Center Groningen, P.O. Box 30.002, 9750 RA Haren, The Netherlands. Email: j.ansuategui.echeita@umcg.nl

For peer review only

ABSTRACT

Introduction. A relevant subsample of patients with chronic low back pain (CLBP) have manifested augmented pain processing, Central Sensitization (CS). Patients with CLBP have limited functioning and participation. Theoretically, physical functioning in patients with CLBP can plausibly be linked to CS; however, evidence to explain such association is scarce. Moreover, there is no gold standard to diagnose CS. The objectives of the study are:

1) To analyze the association between instruments assessing symptoms and signs attributed to CS.
2) To analyze the association between CS and functioning measurements.
3) To analyze whether a decrease in CS is related to better functioning in each of the measurements.

Methods and analysis. Cross-sectional and longitudinal observational study with measurements at baseline and discharge of an interdisciplinary rehabilitation program. 115 adult patients with CLBP are calculated to participate. CS measurements: Central Sensitization Inventory, Quantitative Sensory Testing, and Heart-Rate Variability. Functioning measurements: Lifting capacity, Maximal aerobic capacity, Accelerometry, and Reported functioning. Statistical analyses: 1) correlation between CS measurements, 2) multiple regression between functioning and CS measurements, 3) multiple regression between delta scores of functioning and CS measurements; and corrected for sex and age.

Ethics and dissemination. The study obtained the clearance to its implementation from the Medical Research Ethics Committee of the University Medical Center Groningen (METc UMCG) in July 2017. The results will be disseminated through scientific publications in peer-reviewed journals, presentations at relevant conferences, and reports to stakeholders.

Trial registration number. NTR7167.

For peer review only

STRENGTHS AND LIMITATIONS OF THIS STUDY

- Novel and comprehensive approaches for the assessment of the presence of CS.
- Extensive amount of measurements from multiple domains for the determination of functioning status.
- Longitudinal study with measurements at baseline and discharge of an interdisciplinary rehabilitation program implemented as 'Care as Usual'.
- Strategies allocated to promote participation and adherence, and to mitigate to the largest extent possible selection bias.
- Statistical analyses are performed with proxies of CS and functioning measurements based on their relevance and contribution; the decision may not be beyond debate.

For peer review only

INTRODUCTION

Background

Twenty percent of the general population experiences prolonged or recurrent pain for at least three months, such pain is then considered to be chronic.[1,2] Low back pain is the principal cause of Years Lived with Disability (YLD) and work days lost globally and in Europe,[3] and its sequelae affect 9% of the global population.[4] Moreover, although there are several treatment intervention strategies available for patients with Chronic Low Back Pain (CLBP), treatment effect sizes have only been moderate.[5] Such evidence may explain that 40% of the patients are not satisfied with the treatment received.[2] The economic consequences of chronic pain include direct costs of medical care and pain management treatments, as well as indirect costs of decreased work productivity and sick-leave, and of disability and sickness-retirement costs.[2,6] The indirect costs determine 86-88% of the total costs produced by patients with CLBP.[7,8] In particular, in the Netherlands, the costs of back pain to Dutch society were 3.5 billion in 2007, corresponding to 0.6% of the Gross National Product.[8]

In the past decennia, a relevant subsample of patients with chronic pain, including individuals with CLBP, have shown an increased responsiveness to noxious and non-noxious stimuli, described as Central Sensitization (CS).[9] CS is a result of an imbalance in the nociceptive pathways ("pain pathways") and supraspinal structures due to an amplified facilitation and/or reduced inhibition.[10–12] The phenomenon, CS, is manifested by an amplified pain perception regarding its intensity (hyperalgesia and allodynia), duration (aftersensations and temporal summation), and distribution (expansion of the receptive field); and a reduced conditioned pain modulation.[12] There is currently no gold standard to diagnose CS. The identification of reference clinical symptoms and signs, however, may indicate the presence of CS: disproportionate pain, diffuse distribution, allodynia and hyperalgesia, hypersensitivity unrelated to mechanical stimulus but to environmental sensations (light, temperature, noise, or other stressors), maladaptive psychosocial factors, and low vagal nerve activity.[13–15]

Chronic pain is a multifactorial condition which has impact on physical or body structures and functions, on psychological processes, and on daily activities and quality of life of individuals.[2,16,17] Limited functioning or capacity to perform everyday tasks, essential for an independent living, affects the mobility and self-care, social relationships, and work and leisure performance of individuals with chronic pain.[18] Individual's functioning is composed of body functions (body level), described by impairments individuals experience; and of activities and participation (personal and social levels, respectively) described by capacity and performance tests.[17,19] Patients with CLBP are likely to be less physically active, deconditioned, and disabled. Nowadays much is still unknown about the association of functioning with CLBP, either because research is immature or because the results are conflicting mostly due to differences in methodology;[20–22] let alone the association with CS. The consideration of multiple domains of

For peer review only

functioning, such as work, Physical Activity (PA), fitness, or daily activities measurements during the determination of the functioning status of centrally sensitized patients with CLBP, may improve the assessment and management of pain.

Theoretically, CS can plausibly be linked to physical functioning; more specifically, the presence of CS and/or higher levels of CS can be associated with lower physical functioning performance, and vice versa. Such association could exist, because: 1) the existence of CS has been evidenced due to an overlap in the symptoms and common comorbid disorders in patients who have been considered to experience medically unexplained syndromes, among which CLBP.[23–25] And 2) individuals with CLBP are expected to have limited functioning and participation on their daily living activities. However, not only there is no straightforward evidence of the association between CS and functioning at the moment, but also no gold standard diagnostic instrument for CS exists. The present study described in this research protocol is created to fill these existing gaps in knowledge. It intends to assist on the phenotyping of centrally sensitized patients with CLBP by means of the description of mechanisms involved in CS (CS reference clinical symptoms), and to improve the understanding of the CS phenomenon and of the association and influence of CS on multiple physical functioning elements. Including the influence of various physiological and psychological processes related to chronic pain disorders.

Objective(s)

The objectives of the studies covered by this research protocol are:

1. To analyze the association between three instruments assessing the symptoms and signs attributed to CS in patients with CLBP: Central Sensitization Inventory (CSI), Quantitative Sensory Testing (QST), and Heart-Rate Variability (HRV).
2. To analyze the association between CS and functioning measurements in patients with CLBP. In specific, the association between the three instruments assessing the symptoms and signs attributed to CS (CSI, QST, and HRV) and: Lifting capacity, Aerobic capacity, PA levels and/or PA distribution or patterns, and Patient-reported measurements.
3. To analyze whether a decrease in symptoms and signs attributed to CS are related to better functioning outcomes in each of the measurements in patients with CLBP. In particular, whether a decrease in CS described as: a lower CSI score, an increase in the pain thresholds (QST), and an increase in parasympathetic/vagal tone (HRV); is related to a better functioning described as: an increase in lifting capacity, an increase in aerobic capacity, an increase in PA levels and/or a change in PA distribution or patterns, and better outcome scores in patient-reported measurements.

For peer review only - http://bmjopen.bmj.com/site/about/guidelines.xhtml

METHODS AND ANALYSES

Trial design

An observational study project with both cross-sectional and longitudinal designs is being conducted from September 2017 to September 2019 in the Center for Rehabilitation of the University Medical Center Groningen (CvR – UMCG) in The Netherlands.

Participants

Consecutive patients between 18 and 65 years of age who are primarily referred to the outpatient Pain Rehabilitation Department of the CvR - UMCG because of chronic low back pain (longer than 3 months) and who are admitted for the interdisciplinary pain rehabilitation program are eligible to participate in the study. Additionally, patients are invited to the study if they are mentally competent and can follow instructions. Furthermore, patients are excluded from the study if, based on their file, they: have been diagnosed of neuralgia and/or radicular pain in the legs, have some major disorder or co-morbidity that can affect their physical and/or mental functioning (e.g. psychiatric disorders) or that can interfere with the study, are pregnant (or planning to be), have any specific contraindication according to the standards of practice guidelines of the assessments that they are going to be subject to,[26,27] or take any medication that may interfere with the assessment outcomes such as calcium channel blockers on HRV.

Procedures

Measurements are performed as part of 'Care As Usual' (CAU) for outpatients following the Pain Rehabilitation guidelines of the CvR - UMCG. However, not all measurements of the research protocol belong to the battery of assessments of the CvR – UMCG, as pointed out in the legend of Figure 1.

During the first appointment of the patients with the rehabilitation physician, patients are invited to participate in the studies if they are eligible according to the criteria defined for the studies. The eligible patients who are invited to participate and receive information about the study, but have not reacted, are contacted in a week's time to remind them about the study participation. Patients who signed the informed consent fill in forms with their relevant personal information during the baseline assessment, and they are given vouchers for the travel expenses during the discharge assessment. The participating patients follow CAU trajectory with the Pain Rehabilitation team in terms of assessments, screening, and implementation of the interdisciplinary rehabilitation program. As part of the research protocol, the

For peer review only - http://bmjopen.bmj.com/site/about/guidelines.xhtml

performance of additional assessments and filling of questionnaires on CS and functioning are required. All CS, and objective monitoring and capacity tests are measured at two time points, baseline and discharge: the questionnaire CSI as CAU; the physical assessments lifting capacity and HRV as CAU, and aerobic capacity and QST as additional; and the monitoring of PA with an accelerometer as additional during the first and last week of the rehabilitation program. The pain intensity and functioning questionnaires are measured at 13 time points, baseline and once a week during the 12-week rehabilitation program: PDI as CAU at baseline but as additional during the rehabilitation program, and VAS Pain, WAS and Rand36-PF as additional. A diagram of the project procedure is presented as Figure 1.

(Insert Figure 1 around here)

For peer review only

Measurements

Central Sensitization measurements

To comprehensively assess the symptoms and signs attributed to CS, data is obtained from a questionnaire (CSI) and two physical assessments (HRV and QST).

CSI. The inventory is a self-reported questionnaire with two sections to estimate the severity of CS-related symptoms. Section A is a 25-item questionnaire to assess the presence of the most common CS-related symptoms. Each of the questions is measured on a 5-point Likert scale from 0 (never) to 4 (always), and the total score ranges from 0 to 100, where higher scoring is associated with more severe symptomatology. Section B is a 10-item questionnaire to assess the previously diagnosed CS Syndromes (CSS) and/or conditions related to CS. Higher amount of CSS is associated with higher chances of CS being present. This instrument has shown excellent test-retest reliability (ICC = 0.82-0.97) and internal consistency (Cronbach α = 0.87-0.91) in the general population and in patients with chronic pain,[28–31] as well as good concurrent validity (sensitivity = 81%, specificity = 75%) for CSS diagnosis within patients with chronic pain.[29] For the purposes of the study the Dutch translation of the CSI is used,[32] which has also shown excellent internal consistency (Cronbach α = 0.91) and test-retest reliability (ICC = 0.91 and 0.88) in controls and in patients with chronic musculoskeletal pain respectively.[33] The psychometric information of CSI in patients with CLBP is not known.

QST. A battery of standardized sensory tests that quantifies the function of the somatosensory system. QST tests the characteristics of various sensation and nociceptive processes as representative of afferent nerve fibers and central pathways performance. The whole assessment lasts for 1h, and it consists of six sensory tests, which are measured in seven different predefined body locations: one training location to familiarize the patient with the tests, the most painful location (as pointed out by the patient), and five control locations. See Supplementary material 1 for full description of

locations. The standardized instructions of the sensory tests are read to the patient at each test performance. Of the tests, five consist of mechanical stimuli based on and executed following the QST examination of the German Research Network on Neuropathic Pain (DFNS) protocol,[34] and one for conditioned pain modulation following the Nijmegen-Aalborg Screening QST (NASQ) protocol.[35,36] Overall, the DFNS protocol has shown good test-retest (mean r = 0.86) and inter-observer reliability (mean r = 0.83) in patients with affections in the somatosensory system either with or without pain.[37] For the purpose of avoiding bias due to the order of the measurements and locations, a counterbalancing procedure is established which accounts for the locations and the measurements to be made. This procedure provides three different assessment order groups, all of which begin with the measurements at the training location and end with the conditioned pain modulation test. An additional counterbalancing procedure has been made for allocating patients to these assessment order groups (either 1, 2, or 3); every nine patients a different allocation order is performed to ensure a similar amount of QST assessments per group. All assessors are trained in the QST protocol. The following sensory tests are performed:

- *The Dynamic Mechanical Allodynia (DMA)* to test the presence of abnormal stimulus-evoked pain, is assessed with a soft brush (SENSElab™, Brush-05, Somedic AB, Hörby, Sweden) that is swiped 1-2 cm over the skin. This is performed on a single repetition and on a train of three repetitions (wind-up response). Whether patients feel the sweeping and its pain intensity with a Numeric Rating Scale (NRS) ranging from 0 (no pain) to 100 (most intense pain imaginable) for both the single swipe and the series are recorded in each of the seven locations (see Supplementary material 1).[38,39] The DMA (ratio) is calculated as: DMA = (NRS-3 / NRS-1). The DMA test according to the DFNS protocol has shown to have good test-retest (r = 0.87) and inter-observer reliability (r = 0.79).[37]
- *The Mechanical Pain Threshold (MPT)* to test the ability to perceive sharp, pricking, or stinging stimuli is assessed with a set of standardized weighted pinprick stimulators with seven different intensities (The PinPrick stimulator set, MRC Systems GmbH, Germany) and with a modified method of limits. Starting at the lowest intensity (8mN) this is progressively increased until patients report the stimulus to change from touch to painful (sharp, pricking, or stinging). The intensity of the pinprick which produces the first painful stimulus in mN is recorded in each of the seven locations (see Supplementary material 1).[39–42] The MPT test according to the DFNS protocol has shown to have good test-retest (r = 0.80) and inter-observer reliability (r = 0.80).[37]
- *The Wind-Up Ratio (WUR)* to test the Nervous System's painful stimuli temporal summation or responsiveness to repeated noxious stimuli. Such test is assessed with a suprathreshold pinprick stimulator of 256mN (The PinPrick stimulator set, MRC Systems GmbH, Germany), which is applied on a single repetition and on a train of ten repetitions in an area of 1cm^2 with intervals of one second (wind-up response). The pain intensity

For peer review only - http://bmjopen.bmj.com/site/about/guidelines.xhtml

measured with a Numeric Rating Scale (NRS) ranging from 0 (no pain) to 100 (most intense pain imaginable) for both the single repetition and the series are recorded in each of the seven locations (see Supplementary material 1).[43,44] The WUR (ratio) is calculated as: WUR = (NRS-10 / NRS-1). The WUR test according to the DFNS protocol has shown to have poor test-retest (r = 0.67) and inter-observer reliability (r = 0.56).[37]

- *Mechanical Detection Threshold (MDT)* to test the ability to detect light touch, is assessed with a standardized set of von Frey filaments (OptiHair2-Set, Marstock Nervtest, Germany) with a modified method of limits.[45,46] Starting at the intensity of 16mN, the stimulus application is progressively decreased until patients do not report any sensation. The intensity of the filament that is not perceived in mN is recorded in each of the seven locations (see Supplementary material 1).[47] The MDT test according to the DFNS protocol has shown to have good test-retest (r = 0.90) and inter-observer reliability (r = 0.89).[37]
- *Pressure Pain Threshold (PPT)* to test the ability to perceive pressure pain stimuli on muscles, is assessed with an electronic pressure algometer (Force Ten ™ FDX, Wagner Instruments, USA), with which pressure in the contact point (1cm^2 tip), is progressively but steadily increased until patients report to feel pressure as painful (burning, drilling, or aching). The intensity in N when pressure becomes painful and its pain intensity with a Numeric Rating Scale (NRS) ranging from 0 (no pain) to 100 (most intense pain imaginable) scale is recorded in each of the seven locations (see Supplementary material 1).[34] The PPT test according to the DFNS protocol has shown to have good test-retest (r = 0.88) and inter-observer reliability (r = 0.84).[37]
- *Conditioned Pain Modulation (CPM)* to test the Nervous System's descending pain processing. The CPM test methodology selected for this study follows the recommendations of the EFIC meeting (2013).[48] The pain modulation is assessed by means of PPT tests (Test Stimuli, TS) in a distant location from patients dominant hand (m. Deltoid and m. Quadriceps of the non-dominant side, see Supplementary material 1) before and after immersing the dominant hand in a bucket full with ice and cold water (Cold Conditioning Stimuli, cCS). First, PPT measurements at distant locations are made; then, patients immerse the dominant hand in the bucket for as long as they possibly can with a maximal duration of 3 minutes; finally, PPT measurements are repeated. The PPT results, intensity in N and pain intensity with a Numeric Rating Scale (NRS) ranging from 0 (no pain) to 100 (most intense pain imaginable) scale, in both locations and the time the hand is immersed are recorded. The CPM (%) is calculated as: CPM = [(PPTpost – PPTpre) / PPTpre] ∗ 100; where lower values indicate lower alteration on the nociceptive pathways.[35,36] The CPM test, as the combination of the PPT handheld algometer and cold stimuli, has shown to be one of the most reliable methods with a modest test-retest reliability (ICC = 0.49 and Coefficient of Variation, CV = 63.6%).[49]

For peer review only - http://bmjopen.bmj.com/site/about/guidelines.xhtml

HRV. The test provides an indication of the function and balance of the Autonomic Nervous System (ANS), which is responsible for the control of internal functions including self-regulation, adaptability, and resilience capacities.[50] The ANS is susceptible to mental and emotional processes, which in turn can produce an imbalance in self-regulation (affecting among others HR) by an hyper-activation of the sympathetic tone and reduced parasympathetic/vagal tone.[15,51] HRV testing can monitor heart rhythms, or time and pattern variation on HR and inter-beat (RR) intervals, and can be used to assess the function of the ANS.[52,53] The HRV test is performed by assessors trained by the HeartMath institute, and the 5-minute protocol is executed to guarantee the comparison of the results as recommended by the Task Force for short-term recordings.[52] Patients are instructed to sit calmly and breathe normally, while they have an ear pulse sensor placed which is connected to a computer.[54] The following measures are collected and recorded with the emWave PC Software (emWave®, HeartMath Inc., Boulder Creek, California): mean HR in beats per minute (bpm), mean RR interval in ms; time domain measures in ms as Standard Deviation of Normal-to-Normal interval (SDNN) indicator for cyclic components responsible of RR interval variability, Root Mean Square of Successive Differences (RMSSD) for beat-to-beat variance in HR and parasympathetic/vagal tone; frequency domain measures in ms^2/Hz as Very Low Frequency (VLF, 0.0033-0.04 Hz) indicator for long term regulation and sympathetic tone, Low Frequency (LF, 0.04-0.15 Hz) for sympathetic and parasympathetic/vagal tones, and High Frequency (HF, 0.15-0.40 Hz) for parasympathetic/vagal tone related to breathing rate; and Normalized Coherence indicator for HRV stable or regular pattern over time in %, where higher scores mean more harmonic signal.

Physical Functioning measurements

To determine the functioning status of individuals with chronic pain in multiple domains, data is obtained from two physical assessments (lifting and aerobic capacity tests), an accelerometer (PA level), and a battery of reported functioning questionnaires.

Lifting capacity test. The Floor-to-waist lift capacity test is based on the WorkWell Functional Capacity Evaluation (FCE) protocol [55] and is performed to measure patients work functioning or capacity. Such FCE lift test has been shown to have high test–retest (ICC = 0.81) and inter-rater reliability (ICC = 0.76) in patients with CLBP.[56,57] Assessors are trained in the floor-to-waist lift capacity test procedure. Patients are given standardized instruction to repetitively lift a crate with weights from a shelf at waist height to the floor and back. The test begins with a weight that can easily be lifted, progressively the load is increased, and the test ends when the maximum capacity is reached. The final endpoint can be reached due to: cardiac endpoint (85% of maximum heart rate); biomechanical endpoint (unsafe increasing weight because of lack of load handling control); patient endpoint (patient decided to stop); and criterion endpoint (normal end of the test).[58] The maximal load lifted in kg is recorded along with the endpoint reason, and the patient-reported and the assessor-observed mechanical effort as measured with Borg's Category Ratio scale (CR-

For peer review only - http://bmjopen.bmj.com/site/about/guidelines.xhtml

10).[59] Borg's CR-10 scale ranges from 0 (at rest) to 10 (very, very hard), and has demonstrated to be a valid visual observation of the effort level of patients during their performance of a lifting capacity test.[60]

Aerobic capacity test. The maximal aerobic capacity is assessed with a cardio-pulmonary exercise test (CPET). The test is performed with a cycle ergometer (Ergoselect 200p or Ergoselect 200k, Ergoline, Bitz, Germany) following a defined continuous ramp protocol. Before any cycling begins, patients are interviewed about their daily activities, and their resting heart rhythm and function is recorded with an electrocardiogram (ECG) (assy cam-14, GE medical systems, US). Patients then have an unloaded warm-up of three minutes at 60-70 rotations per minute (rpm) before the test begins. At the start of the test, an experienced exercise physiologist in discussion with a specialized physician or nurse determine the starting workload (25-100 W) and ramp (5-25 W increase per minute) depending on patients estimated fitness level. During the test patients are asked to maintain a constant cadence while the working load is progressively being increased. Also, they are continuously being verbally encouraged to obtain their maximum performance. The maximal performance is determined by various parameters: a temporary loss of strength and energy (= exhaustion), a plateau in the peak oxygen uptake (VO2max), a respiratory exchange ratio (RER) higher than 1.15, and/or a heart-rate (HR) higher than 85% of the predicted maximal HR.[27] Overall, the test lasts for 8-12 minutes and throughout the whole assessment patients are monitored on their cardiac activity with an ECG, on their blood pressure with a cuff algometer, and on their ventilatory gases on a breath-by-breath basis with a metabolic cart (JAEGER ® Vyntus ® CPX, Jaeger, Germany, Hoechberg). The VO2max in ml/min and per kilogram in ml/min/kg is collected as the mean of VO2 over the last 30 seconds of exercise. Along with VO2max are collected: peak workload in watts, HR at end of test in beats per minute (bpm), breathing frequency (BF) at end of test in breaths per min, expiratory tidal volume (VT) in l, peak energy expenditure in Metabolic Equivalent of Task (MET), test duration in min, and the patient-reported and the assessor-observed mechanical effort as measured with Borg's rating of perceived exertion (RPE) scale. Borg's RPE scale ranges from 6 (no exertion at all) to 20 (maximal exertion) [59] and has shown to be highly correlated with HR ($r = 0.74$) and blood lactate ($r = 0.83$),[61] demonstrating to be a reliable instrument to measure work intensity.

PA levels. Objective measurements of PA levels are obtained by means of an accelerometer (ActiGraph GT9X Link, ActiGraph, Pensacola, FL, USA). The accelerometer is a small and light device that monitors wearers' daily PA and energy expenditure. Participating patients wear the accelerometer attached at their hip level for one week continuously during walking hours, in order to ensure the minimum of 4-full-day recording as recommended by activity monitoring reviewers and experts.[62,63] Different algorithms are chosen from the software provided by the manufacturer, ActiLife6 (ActiGraph, Pensacola, FL, USA), to discriminate between low PAs and split patients in three activity intensity categories (sedentary, light, and moderate) [64] and to obtain patients' energy expenditure in kcal [65] and

For peer review only - http://bmjopen.bmj.com/site/about/guidelines.xhtml

MET.[66] The daily and weekly time in hours and percentage of time spent in each of the activity intensity categories as well as the estimated energy expenditure in both kcal and MET are collected.

Reported Functioning. Data from this domain is obtained from four questionnaires sent to patients and filled by them via internet. All the questionnaires require 10-15 minutes to be filled in.

- *Physical functioning (Rand36 Physical Functioning subscale – Rand36-PF)*. The Rand36 is a survey that measures the impact of health on patients' quality of life, patients are asked to only answer the physical functioning (PF) subscale which looks at the limitations patients experience during their daily activities. Each of the 10 items belonging to the PF subscale have 3-level response choices ranging from 0 (limited a lot) to 100 (not limited at all), the total average score ranges from 0 to 100 too; where a higher score is associated with a greater limitation in performing daily activities. For the purposes of the study the Dutch translation of the Rand36 is used, which has shown good internal consistency (Cronbach α = 0.92) and test-retest reliability (r = 0.72-0.82) in the general population.[67,68]
- *Disability (Pain Disability Index – PDI)*. The index is a 7-item questionnaire which measures the interference of pain with functioning in different daily life activities: family/home responsibilities, recreation, social activity, occupation, sexual behavior, self-care, and life-support activity. Each item ranges from 0 (no interference) to 10 (total interference), and the total score ranges from 0 to 70; where scoring higher is associated with higher interference in daily life activities. The PDI has shown modest test-retest reliability (r = 0.44) and high internal consistency (Cronbach α = 0.86) in patients with chronic pain.[69] For the purposes of the study the Dutch translation of the PDI was used, which has shown a good internal consistency (Cronbach α = 0.85) and test-retest reliability (ICC = 0.76) in patients with musculoskeletal pain.[70]
- *Work ability (Work Ability Score – WAS)*. The score is a single-item question which compares patient's current work ability compared with their lifetime best. The result is a score ranging from 0 (unable to work) to 10 (best working ability). The single-item question is part of the Work Ability Index (WAI), a questionnaire which measures work ability and has shown to be test-retest reliable (difference between test and re-test = -0.53) in construction workers.[71] The WAS has shown to be highly correlated to the 28 items of the WAI among women on a long-term sick leave (r = 0.87) [72] and among active workers (r = 0.63).[73]

Supplementary measurements

Demographic characteristics. Patients filled in a form with their details when the QST assessment took place. Such included: age, sex, nationality, height, weight, pain characteristics (affected body area, duration and medication, if

For peer review only - http://bmjopen.bmj.com/site/about/guidelines.xhtml

applicable), educational level, and employment characteristics (work-status and physical work demands per Dictionary of Occupational Titles (DOT) [74]).

Pain-related questionnaires.

- *Pain intensity (Visual Analogue Scale – VAS Pain)*. The scale is a single-item question to assess the current pain intensity in adults. It is a straight line with 'no pain' (score = 0) at one end and 'pain as bad as it could be' (score = 100) at the other, in which patients are asked to mark the line at the level that represents their pain level. The score is the distance between the mark and the 'no pain' end, where scoring higher is associated with greater pain intensity. The VAS Pain has shown good validity (r = 0.62-0.91) and good test-retest reliability (literate r = 0.94 and illiterate r = 0.71) in patients with rheumatic pain conditions.[75] VAS Pain is sent to patients and filled in by them via internet along with the battery of functioning questionnaires (described above); i.e. at baseline and weekly during the interdisciplinary rehabilitation program.
- *Body response to a straining exercise (Pain Response Questionnaire – PRQ).*[76] The questionnaire assesses the pain intensity of the different body locations on an 11-point numeric scale (NRS) ranging from 0 (no pain) to 10 (worst pain imaginable), and the type of pain felt (muscle soreness, other, both, or unknown). Additionally it controls for the use of medication and for any other strenuous physical activity. For the purposes of this study, the body locations measured are predefined to four and the questionnaire is being filled in three occasions: before the CPET, immediately afterwards, and 24h later. PRQ is filled at the same moment when the aerobic capacity test is performed; i.e. during baseline and discharge assessments.

Psychological screening questionnaires. Patients filled in the questionnaires via internet which were sent per CAU procedures by the Pain Rehabilitation team. Such were:

- *Pain Catastrophizing Scale (PCS)*. The 13-item reported questionnaire measures measured in a 5-point Likert scale (0 - not at all, 4 - all the time) the degree to which patients have had catastrophic thoughts or feelings during their pain experience and accounts for three subscales or dimensions: rumination, magnification, and helplessness.[77] For the purposes of the study the Dutch translation of the PCS was used,[78] which has shown good internal consistency (Cronbach α = 0.85-0.91) and test-retest reliability (r = 0.92) among healthy individuals (students) and patients with chronic low back pain.[79]
- *Injustice Experience Questionnaire (IEQ)*. The 12-item reported questionnaire assesses in a 5-point Likert scale from (0 – never, 4 - all the time) four different aspects of the perceived injustice due to injury: severity, blame, unfairness, and irreparability. The questionnaire has shown to have good internal consistency (Cronbach α =

For peer review only - http://bmjopen.bmj.com/site/about/guidelines.xhtml

0.92) and test-retest reliability (ICC = 0.98) in a group of patients with fibromyalgia.[80] For the purposes of the study the Dutch translation of the IEQ was used, as translated by the Pain in Motion group.[81]

- *Brief Symptom Inventory (BSI)*. The inventory is a 53-item reported questionnaire which assesses patient's current level of psychological distress with a 5-point Likert scale questions (0 - not at all, 4 - extremely), the amount of symptoms present, and their severity.[82] BSI is composed of nine subscales: somatization, obsession-compulsion, interpersonal sensitivity, depression, anxiety, hostility, phobic anxiety, paranoid ideation, and psychoticism. For the purposes of the study the Dutch translation of the BSI was used, which has shown good internal consistency (Cronbach α = 0.71-0.87) and test-retest reliability (r = 0.71-0.89) among psychiatric out-patient and healthy individual groups.[83]

Sample size

Due to the lack of relevant previous or similar studies, the sample sizes for the several analyses described in this research protocol are estimated a priori and are exploratory as a result.

The following sample size calculations as well as the planned statistical analyses later described will be performed with a proxy for each of the main variables being three variables for CS and four for functioning. Additionally, due to the influence of patient's sex and age in the assessment outcomes, all analyses will include them for a moderator effect.

Sample size for objective 1: To analyze the association between three instruments assessing symptoms and signs attributed to CS.

Correlation analyses will be performed for this objective. Because the correlation between the different CS measurements is expected to be medium, for a correlation coefficient of 0.3, an α error probability of 0.05, and a power of 0.8, a minimum sample size of 84 is calculated (G*Power for Windows, Version 3.1.9.2).

Sample size for objective 2: To analyze the association between CS and various functioning measurements; and for objective 3: To analyze whether a decrease in CS is related to better functioning outcomes in various measurements.

Multiple regression analyses will be performed for objectives 2 and 3. Valid multiple regression models require a minimum of 10 participants per variable added.[84] Thus, because minimally three CS measurements and sex and age are required as independent variables, a sample of 50 patients would be necessary. However, since models may also need to be controlled for the confounding effect of other demographic and test-related variables, a minimum of 80 patients is aimed. Finally, patients measured at discharge could be about 30% less than at baseline due to either not

For peer review only - http://bmjopen.bmj.com/site/about/guidelines.xhtml

following the interdisciplinary rehabilitation program or personal reasons, a sample size of 115 patients may be required in this scenario.

Analyses

Data records from the different tests and questionnaires will be collected and merged into one database. The database management will be managed by the project team and handled according to the internal regulations of the UMCG. The analyses of the study will be performed with SPSS software version 22.0 (IBM Corp., NY).

Before any analysis, data will be prepared:

- Variables need to be calculated: BMI; DMA, WUR, and CPM tests (see Methods section, Main measurements: CS); PDI, Rand36-PF, PCS, BSI, and IEQ questionnaires.
- HRV: data will be pre-processed to correct for any artifacts that may be present at the beginning or at the end of the recording.
- QST: all measurements will be transformed to standard normal distribution (Z-transformation).
- To perform the analyses to respond objective 3, Delta scores are computed (difference in scores between baseline and discharge) for CS, functioning, and those confounder variables which are also measured at the same time points as the main measurements.

Proxies of the main measurements were selected to perform analyses. CS variables are: the sum score of CSI part A, the RMSSD for HRV, and the MPT for QST. Functioning variables are: the maximal weight lifted for lifting capacity, the VO2max per kg for the maximal aerobic capacity, the daily VM counts for PA, and the average score of Rand36-PF for reported physical functioning.

The presence of missing data will be checked. If a variable has more than 10% of missing data cases, its distribution will be further examined. When the distribution is not of influence, analyses with listwise deletion are planned. Regarding the presence of outliers, cases at 3 SD from the mean will be considered as outliers. Their influence will be measured with Cook's distance and leverage values. When the outliers are not statistically influential and/or there is rationale for the outlier, no further actions are planned. The descriptive statistics of the sample characteristics and main test results will be at this point calculated; numerical variables as mean and SD, and categorical variables as counts and percentage of each category level.

Statistical approach for objective 1: To analyze the association between three instruments assessing CS.

For peer review only - http://bmjopen.bmj.com/site/about/guidelines.xhtml

For peer review only

Multiple partial correlation analyses will be performed corrected for potential confounders. CS measurements will be the main variables and sex and age will be covariates. Results are reported as Pearson's r coefficient and p-values, with and without controlling for sex and age. The significance level for all analyses is established at 0.05 level.

Statistical approach for objective 2: To analyze the association between CS and various functioning measurements.

First multiple correlation analyses, followed by multiple regression analyses with data obtained at baseline will be performed. Each of the main outcomes from functioning measurements will be introduced in the models as dependent variable, CS measurements as independent variables, and sex, age, and other demographic characteristics and/or test-related variables as potential confounders.

Statistical approach for objective 3: To analyze whether a decrease in CS is related to better functioning outcomes in various measurements.

Similar approach to objective 2 will be performed: first multiple correlation analyses will be performed with data obtained at baseline and discharge, followed by multiple correlation analyses with delta scores of the data, and finally multiple regression analyses again with delta scores. As in objective 2, each of the main outcomes from functioning measurements will be introduced in the models as dependent variable, CS measurements as independent variables, and sex, age, and other demographic characteristics and/or test-related variables as potential confounders.

Confounders entering the final models of objectives 2 and 3 will be selected based on their partial correlation coefficient with CS and functioning and they will be included if $p \leq 0.10$. All multiple regression models will have the assumptions checked. Results of objectives 2 and 3 will be reported as coefficient (b), confidence intervals (95% CI), p-values, and explained variance, with and without controlling for confounders. The significance level of the correlation analyses and of the multiple regression analyses is established at 0.05 level.

DISCUSSION

Several patients with CLBP have shown common manifestations with other chronic pain conditions, which may evidence the presence of CS in these patients. Yet knowledge on CS is developing, especially in patients with CLBP, and evidence explaining its association with functioning is lacking. This research protocol aims to bridge that gap of knowledge with a novel approach. In the first place, the study (objective 1) will describe the mechanisms of CS in patients with CLBP with instruments that assess CS symptoms from different perspectives. Furthermore, the study (objectives 2 and 3) will also analyze longitudinally whether the presence of CS and/or higher levels of CS can be associated with lower physical functioning performance, for which functioning will be assessed with several objective monitoring and capacity tests, and self-reported measurements.

For peer review only - http://bmjopen.bmj.com/site/about/guidelines.xhtml

Different CS assessment instruments are selected for the research protocol to examine the CS symptoms and signs attributed to CS. 1) The hypersensitivity unrelated to mechanical stimulus are assessed with CSI. A questionnaire designed to measure CS as it has been shown to be associated to factors known to contribute to CS and is able to identify somatic and emotional symptoms related to CSS.[28,85] 2) The disproportionate pain, diffuse distribution, and allodynia and hyperalgesia, are measured with QST. The QST is a battery of non-invasive standardized tests that assists in the identification of sensory and pain thresholds, the determination of pain intensity perception, and the assessment of the conditioned pain modulation. Its results can allow pain characterization (phenotyping) and processing, and monitoring its progression.[86–90] 3) The low vagal nerve activity is examined with HRV. A non-invasive standardized measure that characterizes the function of the ANS.[51] The somatic and emotional symptoms related to CS syndromes may generate an imbalance between a hyperactivation of the sympathetic tone and a reduction of the parasympathetic/vagal tone.[51,91,92] 4) The maladaptive psychosocial factors, although they are not necessary for the clinical classification of CS, they are nevertheless important for patient's assessment and measured with psychological screening questionnaires for perceived injustice, catastrophizing, and psychological distress. Some of the CS assessment instruments used in this study are more acknowledged than others in CS literature. CSI is widely used to measure CS as it was designed as a proxy for it; however, its internal consistency and reliability in patients with CLBP has not yet been made available. Also, currently evidence is piling up on the necessity of routinely implementing the elements of QST relevant to patients' conditions in clinical practice due to being able to assess the somatosensory function,[90,93] even though evidence in patients with CLBP is limited. Finally, literature on HRV as an instrument that may reflect an imbalance of ANS in patients with chronic pain conditions, specifically in those patients that have shown an overlap in the CS symptoms, is scarce. In absence of an instrument that can diagnose CS, we are confident that the selected CS assessment instruments are considered best available, and will be able to comprehensively measure from different perspectives the clinical symptoms and signs indicative of CS being present, to provide further insights on the mechanisms of CS and to assist on the phenotyping of centrally sensitized patients with CLBP.

Various functioning assessments are selected for the research protocol to determine the functioning status of centrally sensitized patients with CLBP, including several objective monitoring and capacity tests, and self-reported measurements. 1) The floor-to-waist lifting capacity test is a physical assessment from the FCE that has demonstrated the best predictability for Return-to-Work.[94] 2) The aerobic capacity, expressed as VO2max, can be best assessed with a maximal aerobic capacity test such as the CPET. 3) The PA is measured with an accelerometer, an easy wearable activity-monitoring device that provides objective measurements of PA levels and PA distribution or patterns. 4) Patient-reported functioning questionnaires present patients pain experience, how they perceive or believe their functioning and participation is. Although questionnaires results can differ from their actual physical performance, both

For peer review only - http://bmjopen.bmj.com/site/about/guidelines.xhtml

measurements complement each other and together they may provide a more comprehensive overview of patients with CLBP.[95-97] Assessments such as lifting capacity and self-reported measurements have been routinely used to determine the functioning status of patients with CLBP. CPET on the other hand, even though it is scientifically superior to a submaximal aerobic capacity test, has barely been implemented with patients with CLBP and, as a result, whether a maximal CPET could be applied systematically with these patients and their pain response to it is unknown. Moreover, even if there is a large amount of literature on PA, the methodology implemented is very broad regarding population groups and/or measuring instruments, which hinders the synthesis of the understanding of PA of patients with CLBP. To bridge the gap, it may be of use an objective measurement of PA and monitored with accelerometers such as those of ActiGraph, who has shown to produce the most reliable devices for recording PA in free-living conditions.[98,99] It is believed that the assessments described are suitable and relevant for the purpose of measuring functioning from multiple domains, and that the extended knowledge and the better understanding of the mechanisms involved obtained is not limited to CLBP but can be extended to CS.

Data collected for this project is large and the measurements of CS and functioning collected involve a broad amount of variables, as a result proxies will be used for the statistical analyses. Previous research in CS assessments, on the one hand, and in functioning assessments, on the other, has reported different methodologies and outcome parameters. Reason for which, even if authors have selected the proxy variables based on their relevance and contribution to the purposes of the current studies, the choice on the following measurements may not be beyond debate: for HRV the RMSSD, as it is a measurement of the parasympathetic/vagal tone which is not influenced by breathing;[100] for QST the MPT, since it measures the perceived skin mechanical hyperalgesia which is one of the main indicators of CS;[12] for the maximal aerobic capacity the VO2max corrected for the body mass of the individual, due to the effect of body mass on peak oxygen uptake; and for PA the daily VM counts, because it characterizes movement duration and intensity.[101]

Notwithstanding the efforts of the team in the design and implementation to obtain the most extensive insights of CS and its association with functioning, it should be considered that patients participating are volunteers; and that the exclusion criteria, although based on patient's file, is dependent on doctor's judgment, which may imply some subjectivity. These circumstances may have some influence in the results; however, their impact of is so far unknown to the authors. Additionally, despite the efforts of the team to promote the study participation and participant retention, it is likely that patient recruitment may be slower than anticipated and that patient may not have a complete follow-up. However, since the measurement period is ample and a withdrawal of 30% of patients has been calculated (see Methods section, Sample size), we remain positive about the sample size and expect to have enough participants to perform analyses with sufficient power. Also, measurements are planned to be within three weeks from the actual baseline and

For peer review only - http://bmjopen.bmj.com/site/about/guidelines.xhtml

discharge assessments performed by the Pain Rehabilitation team for comparability of the results. The CSI and PDI questionnaires during baseline assessment are sent to be filled six weeks before the rest of the measurements are obtained. The distance is due to waiting list time and no important changes in patients' behavior and/or mindset are foreseen; thus, these measurements are expected to be comparable to the rest of measurements at baseline.

All things considered, the results obtained from the measurements described in this research protocol can help better describe and understand the mechanisms involved in CS and, of these, the ones affecting the functionality of patients with CLBP; and then be used to optimize improve the effectiveness of pain management interventions. To the authors' knowledge, the study described in this research protocol are the first off its kind and it may become an important breakthrough for researchers involved in the study of pain, for the treatment strategy of patients with chronic pain by healthcare professionals, and for patient's pain education.

ETHICS AND DISSEMINATION

All participating patients sign the informed consent before any assessment takes place. Formal ethical clearance to perform the study has been obtained from the METC University Medical Center Groningen (The Netherlands) in July 2017 (METC 2016/702). All procedures are in accordance with the ethical standards of the Helsinki Declaration of 1975 as revised in 2014 [102] and with Dutch regulations of the Medical Research Involving Human Subjects Act, in Dutch: Wet Medisch-Wetenschappelijk Onderzoek (WMO), amended in 2018.[103]

The dissemination strategy of the research study results to will be through scientific publications in peer-reviewed journals and presentations in relevant national and international conferences. In addition to it, reports will be send by post to participants and reports and presentations in meetings will be provided to stakeholders.

For peer review only

ABBREVIATIONS

ANS	Autonomic Nervous System
BF	Breathing Frequency at the end of CPET
BMI	Body Mass Index
BSI	Brief Symptom Inventory
CAU	Care As Usual
cCS	cold Conditioning Stimulus
CLBP	Chronic Low Back Pain
CPET	Cardio-Pulmonary Exercise Test
CPM	Conditioned Pain Modulation
CS	Central Sensitization
CSI	Central Sensitization Inventory
CSS	Central Sensitization Syndromes
CvR-UMCG	Center for Rehabilitation of the UMCG; in Dutch: Centrum voor Revalidatie
CR-10 scale	Category Ratio (0-10) scale
DMA	Dynamic Mechanical Allodynia
DFNS	German Research Network on Neuropathic Pain; in German: Deutschen Forschungsverbund Neuropathischer Schmerz
DOT	Dictionary of Occupational Titles
ECG	Electrocardiogram
FCE	Functional Capacity Evaluation
HF	High Frequency
HR	Heart Rate
HRV	Heart Rate Variability
IC	Informed Consent
ICC	Intra-Class Correlation
IEQ	Injustice Experience Questionnaire
LF	Low Frequency
MDT	Mechanical Detection Threshold
MET	Metabolic Equivalent of Task
METc	Medical Research Ethics committee; in Dutch: Medisch Ethische Toetsing commissie
MPT	Mechanical Pain Threshold
NASQ	Nijmegen-Aalborg Screening QST
NRS	Numeric Rating Scale
PA	Physical Activity
PCS	Pain Catastrophizing Scale
PDI	Pain Disability Index
PF	Physical Functioning
PPT	Pressure Pain Threshold
PRQ	Pain Response Questionnaire
QST	Quantitative Sensory Testing
Rand36-PF	Physical Functioning subscale of the Rand36 questionnaire
RER	Respiratory Exchange Ratio

For peer review only

rMSSD	Root Mean Square of Successive Differences between NN intervals
RPE	Rating of Perceived Exertion
RR interval	Inter-beat interval
SDNN	Standard Deviation of Normal-to-Normal (NN) intervals
TS	Test Stimulus
UMCG	Universitair Medisch Centrum Groningen
VAS	Visual Analogue Scale
VLF	Very Low Frequency
VO2max	Peak VO2, Peak oxygen uptake
VT	Expiratory Tidal Volume
WAI	Work Ability Index
WAS	Work Ability Score
WMO	Medical Research Involving Human Subjects Act; in Dutch: Wet Medisch-wetenschappelijk Onderzoek met Mensen
WUR	Wind-Up Ratio
YLD	Years Lived with Disability

For peer review only

STATEMENTS

Author's Contributions: JAE was responsible for the study concept and design, data collection, and manuscript preparation and edit; HRSP, RD, and MR were responsible for the study concept and design, and critical review of the manuscript; IS, HT and APW were responsible for the study design and critical review of the manuscript.

Competing interests: The authors declare that there are no conflicts of interest.

Patient and Public Involvement: Personnel of the CvR-UMCG collaborate in the study, in particular, the Pain rehabilitation team during patient management and data collection process and the Central exercise lab team on data collection process. The authors wish to express their gratitude to the collaborators and to the patients that participate.

Funding: The Pain Rehabilitation Development Center (in Dutch: Ontwikkelcentrum PijnRevalidatie (OPR)) of the CvR-UMCG funded the research study.

Trial registration: NTR7167, April 16th 2018; retrospectively registered.

Ethics approval: The Medical Research Ethics Committee of the University Medical Center Groningen (METc UMCG) provided a positive reaction in July 2017 to the development of the research study "*Central Sensitization and Functioning in patients with Chronic Low Back Pain*" (METc 2016/702). Its first amendment was approved in October 2017 and its second amendment in August 2018.

For peer review only

REFERENCES

1. The Joint Task Force (JTF). ICD-11 Beta Draft - Mortality and Morbidity Statistics[Internet]. Available from: https://icd.who.int/browse11/l-m/en#/ (Accessed 24 May 2018).
2. Breivik H, Collett B, Ventafridda V, et al. Survey of chronic pain in Europe: Prevalence, impact on daily life, and treatment. Eur J Pain. 2006;10(4):287–333.
3. Hoy D, March L, Brooks P, et al. The global burden of low back pain: estimates from the Global Burden of Disease 2010 study. Ann Rheum Dis. 2014;73(6):968–74.
4. Vos T, Flaxman AD, Naghavi M, et al. Years lived with disability (YLDs) for 1160 sequelae of 289 diseases and injuries 1990–2010: A systematic analysis for the Global Burden of Disease Study 2010. Lancet. 2012;380(9859):2163–96.
5. Foster NE, Anema JR, Cherkin D, et al. Prevention and treatment of low back pain: evidence, challenges, and promising directions. Lancet. 2018;391(10137):2368–83.
6. Boonen A, van den Heuvel R, van Tubergen A, et al. Large differences in cost of illness and wellbeing between patients with fibromyalgia, chronic low back pain, or ankylosing spondylitis. Ann Rheum Dis. 2005;64:396–402.
7. Dagenais S, Caro J, Haldeman S. A systematic review of low back pain cost of illness studies in the United States and internationally. Spine J. 2008;8(1):8–20.
8. Lambeek LC, van Tulder MW, Swinkels ICS, K et al. The Trend in Total Cost of Back Pain in the Netherlands in the Period 2002 to 2007. Spine. 2011;36(13):1050–8.
9. Giesecke T, Gracely RH, Grant MAB, et al. Evidence of augmented central pain processing in idiopathic chronic low back pain. Arthritis Rheum. 2004;50(2):613–23.
10. Woolf CJ. Central sensitization: implications for the diagnosis and treatment of pain. Pain. 2011;152(3 Suppl.):S2–15.
11. Arendt-Nielsen L, Morlion B, Perrot S, et al. Assessment and manifestation of central sensitisation across different chronic pain conditions. Eur J Pain. 2018;22(2):216–41.
12. den Boer C, Dries L, Terluin B, et al. Central sensitization in chronic pain and medically unexplained symptom research: A systematic review of definitions, operationalizations and measurement instruments. J Psychosom Res. 2019;117:32–40.
13. Nijs J, Torres-Cueco R, van Wilgen P, et al. Applying Modern Pain Neuroscience in Clinical Practice: Criteria for the Classification of Central Sensitization Pain. Pain Physician. 2014;17(5):447–57.
14. Smart KM, Blake C, Staines A, et al. The Discriminative Validity of “Nociceptive,” “Peripheral Neuropathic,” and “Central Sensitization” as Mechanisms-based Classifications of Musculoskeletal Pain. Clin J Pain. 2011;27(8):655–

For peer review only

63.

15. De Couck M, Nijs J, Gidron Y. You May Need a Nerve to Treat Pain: The Neurobiological Rationale for Vagal Nerve Activation in Pain Management. Clin J Pain. 2014;30(12):1099–105.

16. O'Brien T, Breivik H. The impact of chronic pain — European patients' perspective over 12 months. Scand J Pain. 2012;3(1):23–9.

17. Nugraha B, Gutenbrunner C, Barke A, et al. The IASP classification of chronic pain for ICD-11. Pain. 2019;160(1):88–94.

18. Cieza A, Stucki G, Weigl M, et al. ICF Core Sets for low back pain. J Rehabil Med. 2004; Suppl. 44:69–74.

19. Schneidert M, Hurst R, Miller J, et al. The role of Environment in the International Classification of Functioning, Disability and Health (ICF). Disabil Rehabil. 2003;25(11–12):588–95.

20. Verbunt JA, Seelen HA, Vlaeyen JW, et al. Disuse and deconditioning in chronic low back pain: concepts and hypotheses on contributing mechanisms. Eur J Pain. 2003;7(1):9–21.

21. Ryan CG, Grant PM, Dall PM, et al. Individuals with chronic low back pain have a lower level, and an altered pattern, of physical activity compared with matched controls: An observational study. Aust J Physiother. 2009;55(1):53–8.

22. van Dieën JH, Reeves NP, Kawchuk G, et al. Motor Control Changes in Low-Back Pain: Divergence in Presentations and Mechanisms. J Orthop Sport Phys Ther. 2018;1–24.

23. Schur EA, Afari N, Furberg H, et al. Feeling Bad in More Ways than One: Comorbidity Patterns of Medically Unexplained and Psychiatric Conditions. J Gen Intern Med. 2007;22:818–21.

24. Kindler LL, Jones KD, Perrin N, et al. Risk Factors Predicting the Development of Widespread Pain From Chronic Back or Neck Pain. J Pain. 2010;11(12):1320–8.

25. Fleming KC, Volcheck MM. Central sensitization syndrome and the initial evaluation of a patient with fibromyalgia: a review. Rambam Maimonides Med J. 2015;6(2):e0020.

26. Hart DL, Isernhagen SJ, Matheson LN. Guidelines for Functional Capacity Evaluation of People With Medical Conditions. J Orthop Sport Phys Ther. 1993;18(6):682–6.

27. American Thoracic Society, American College of Chest Physicians. ATS/ACCP Statement on Cardiopulmonary Exercise Testing. Am J Respir Crit Care Med. 2003;167(2):211–77.

28. Mayer TG, Neblett R, Cohen H, et al. The development and psychometric validation of the central sensitization inventory. Pain Pract. 2012;12(4):276–85.

29. Neblett R, Cohen H, Choi Y, et al. The Central Sensitization Inventory (CSI): establishing clinically significant values for identifying central sensitivity syndromes in an outpatient chronic pain sample. J Pain. 2013;14(5):438–45.

30. Neblett R, Hartzell MM, Mayer TG, et al. Establishing Clinically Relevant Severity Levels for the Central

For peer review only

Sensitization Inventory. Pain Pract. 2017;17(2):166–75.

31. Scerbo T, Colasurdo J, Dunn S, et al. Measurement Properties of the Central Sensitization Inventory: A Systematic Review. Pain Pract. 2018;18(4):544–54.

32. van Wilgen CP, Meeus M, Descheemaeker F, et al. Central Sensitization Inventory – Nederlandse consensusvertaling [Internet]. 2013. Available from: http://www.paininmotion.be/storage/app/media//materials/CSI-consensusvertaling.pdf (Accessed 19 Jul 2018).

33. Kregel J, Vuijk PJ, Descheemaeker F, et al. The Dutch Central Sensitization Inventory (CSI). Clin J Pain. 2016;32(7):624–30.

34. Rolke R, Baron R, Maier C, et al. Quantitative sensory testing in the German Research Network on Neuropathic Pain (DFNS): Standardized protocol and reference values. Pain. 2006;123(3):231–43.

35. Wilder-Smith O. A paradigm-shift in pain medicine: implementing a systematic approach to altered pain processing in everyday clinical practice based on quantitative sensory testing. Center for Sensory-Motor Interaction (SMI), Department of Health Science and Technology, Aalborg University. 2013:76p.

36. Timmerman H, Wilder-Smith O, van Weel C, et al. Detecting the neuropathic pain component in the clinical setting: a study protocol for validation of screening instruments for the presence of a neuropathic pain component. BMC Neurol. 2014;14:94–102.

37. Geber C, Klein T, Azad S, et al. Test–retest and interobserver reliability of quantitative sensory testing according to the protocol of the German Research Network on Neuropathic Pain (DFNS): A multi-centre study. Pain. 2011;152(3):548–56.

38. LaMotte RH, Shain CN, Simone DA, et al. Neurogenic hyperalgesia: psychophysical studies of underlying mechanisms. J Neurophysiol. 1991;66(1):190–211.

39. Ziegler EA, Magerl W, Meyer RA, et al. Secondary hyperalgesia to punctate mechanical stimuli. Brain. 1999;122(12):2245–57.

40. Chan AW, MacFarlane IA, Bowsher D, et al. Weighted needle pinprick sensory thresholds: a simple test of sensory function in diabetic peripheral neuropathy. J Neurol Neurosurg Psychiatry. 1992;55(1):56–9.

41. Greenspan JD, McGillis SLB. Thresholds for the Perception of Pressure, Sharpness, and Mechanically Evoked Cutaneous Pain: Effects of Laterality and Repeated Testing. Somatosens Mot Res. 1994;11(4):311–7.

42. Hampf G, Bowsher D, Wells C, et al. Sensory and autonomic measurements in idiopathic trigeminal neuralgia before and after radiofrequency thermocoagulation: differentiation from some other causes of facial pain. Pain. 1990;40(3):241–8.

43. Price DD, Hu JW, Dubner R, et al. Peripheral suppression of first pain and central summation of second pain evoked by noxious heat pulses. Pain. 1977;3(1):57–68.

For peer review only

For peer review only - http://bmjopen.bmj.com/site/about/guidelines.xhtml

44. Magerl W, Wilk SH, Treede R-D. Secondary hyperalgesia and perceptual wind-up following intradermal injection of capsaicin in humans. Pain. 1998;74(2–3):257–68.

45. Weinstein S. Intensive and extensive aspects of tactile sensitivity as a function of body part, sex and laterality. In: Kenshalo DR, ed. The skin senses: Proceedings. Springfield, IL, US: Charles C Thomas. 1968:195–222.

46. Fruhstorfer H, Gross W, Selbmann O. von Frey hairs: new materials for a new design. Eur J Pain. 2001;5(3):341–2.

47. Baumgärtner U, Magerl W, Klein T, et al. Neurogenic hyperalgesia versus painful hypoalgesia: two distinct mechanisms of neuropathic pain. Pain. 2002;96(1–2):141–51.

48. Yarnitsky D, Bouhassira D, Drewes AM, et al. Recommendations on practice of conditioned pain modulation (CPM) testing. Eur J Pain. 2015;19(6):805–6.

49. Imai Y, Petersen KK, Mørch CD, et al. Comparing test–retest reliability and magnitude of conditioned pain modulation using different combinations of test and conditioning stimuli. Somatosens Mot Res. 2016;33(3–4):169–77.

50. McCorry LK. Physiology of the autonomic nervous system. Am J Pharm Educ. 2007;71(4):78–88.

51. McCraty R, Atkinson M, Tomasino D, et al. The coherent heart: heartbrain interactions, psychophysiological coherence, and the emergence of system-wide order. Boulder Creek, CA: Institute of Heartmath; 2009.

52. Task Force of the European Society of Cardiology and the North American Society of Pacing and Electrophysiology. Heart rate variability, standards of measurement, physiological interpretation, and clinical use. Circulation. 1996;93(5):1043–65.

53. McCraty R, Shaffer F. Heart Rate Variability: New Perspectives on Physiological Mechanisms, Assessment of Self-regulatory Capacity, and Health Risk. Glob Adv Heal Med. 2015;4(1):46–61.

54. HeartMath Inc. emWave Pro Plus Assessments [Internet]. 2018. Available from: https://heartcloud.com/library/asmts.html (Accessed 27 Feb 2019).

55. Isernhagen SJ, Hart DL, Matheson LM. Reliability of independent observer judgments of level of lift effort in a kinesiophysical Functional Capacity Evaluation. Work. 1999;12(2):145–50.

56. de Baets S, Calders P, Schalley N, et al. Updating the Evidence on Functional Capacity Evaluation Methods: A Systematic Review. J Occup Rehabil. 2018;28(3):418–28.

57. Brouwer S, Reneman MF, Dijkstra PU, et al. Test–Retest Reliability of the Isernhagen Work Systems Functional Capacity Evaluation in Patients with Chronic Low Back Pain. J Occup Rehabil. 2003;13(4):207–18.

58. Soer R, van der Schans CP, Geertzen JH, et al. Normative Values for a Functional Capacity Evaluation. Arch Phys Med Rehabil. 2009;90(10):1785–94.

59. Borg G. Borg's perceived exertion and pain scales.. Champaign, IL, US: Human Kinetics; 1998.

60. Reneman MF, Fokkens AS, Dijkstra PU, et al. Testing Lifting Capacity: Validity of Determining Effort Level by

For peer review only

Means of Observation. Spine. 2005;30(2):e40–6.

61. Scherr J, Wolfarth B, Christle JW, et al. Associations between Borg's rating of perceived exertion and physiological measures of exercise intensity. Eur J Appl Physiol. 2013;113(1):147–55.

62. Trost SG, McIver KL, Pate RR. Conducting Accelerometer-Based Activity Assessments in Field-Based Research. Med Sci Sports Exerc. 2005;37(11 Suppl.):S531–43.

63. Migueles JH, Cadenas-Sanchez C, Ekelund U, et al. Accelerometer Data Collection and Processing Criteria to Assess Physical Activity and Other Outcomes: A Systematic Review and Practical Considerations. Sport Med. 2017;47(9):1821–45.

64. Keadle SK, Shiroma EJ, Freedson PS, et al. Impact of accelerometer data processing decisions on the sample size, wear time and physical activity level of a large cohort study. BMC Public Health. 2014;14(1):1210.

65. Sasaki JE, John D, Freedson PS. Validation and comparison of ActiGraph activity monitors. J Sci Med Sport. 2011;14(5):411–6.

66. Crouter SE, Kuffel E, Haas JD, et al. Refined two-regression model for the ActiGraph accelerometer. Med Sci Sports Exerc. 2010;42(5):1029–37.

67. van der Zee KI, Sanderman R. RAND-36. umcg.nl [Internet]. Available from: https://www.umcg.nl/SiteCollectionDocuments/research/institutes/SHARE/assessment tools/handleiding_rand36_2e_druk.pdf (Accessed 28 Jun 2018).

68. van der Zee KI, Sanderman R, Heyink JW, et al. Psychometric qualities of the rand 36-item health survey 1.0: A multidimensional measure of general health status. Int J Behav Med. 1996;3(2):104–22.

69. Tait RC, Chibnall JT, Krause S. The Pain Disability Index: psychometric properties. Pain. 1990;40(2):171–82.

70. Soer R, Köke AJA, Vroomen PCAJ, et al. Extensive Validation of the Pain Disability Index in 3 Groups of Patients With Musculoskeletal Pain. Spine. 2013;38(9):e562–8.

71. de Zwart BCH, Frings-Dresen MHW, van Duivenbooden JC. Test-retest reliability of the Work Ability Index questionnaire. Occup Med. 2002;52(4):177–81.

72. Ahlstrom L, Grimby-Ekman A, Hagberg M, et al. The work ability index and single-item question: associations with sick leave, symptoms, and health – A prospective study of women on long-term sick leave. Scand J Work Environ Heal. 2010;36(5):404–12.

73. El Fassi M, Bocquet V, Majery N, et al. Work ability assessment in a worker population: comparison and determinants of Work Ability Index and Work Ability score. BMC Public Health. 2013;13:305–14.

74. U.S. Department of Labor, Employment and Training Administration. Dictionary of Occupational Titles. 4th ed. Washington; 1991.

75. Hawker GA, Mian S, Kendzerska T, et al. Measures of adult pain: Visual Analog Scale for Pain (VAS Pain),

For peer review only

Numeric Rating Scale for Pain (NRS Pain), McGill Pain Questionnaire (MPQ), Short-Form McGill Pain Questionnaire (SF-MPQ), Chronic Pain Grade Scale (CPGS), Short Form-36 Bodily Pain Scale (SF36 BPS), and Measure of Intermittent and Constant Osteoarthritis Pain (ICOAP). Arthritis Care Res. 2011;63(S11):S240–52.

76. Soer R, Groothoff JW, Geertzen JHB, et al. Pain Response of Healthy Workers Following a Functional Capacity Evaluation and Implications for Clinical Interpretation. J Occup Rehabil. 2008;18(3):290–8.

77. Sullivan MJL, Bishop SR, Pivik J. The pain catastrophizing scale: development and validation. Psychol Assess. 1995;7(4):524–32.

78. Crombez G, Vlaeyen JWS. Pain Catastrophizing Scale (PCS) - Dutch Version (PCS-DV) [Internet]. 1996. Available from: https://meetinstrumentenzorg.blob.core.windows.net/test-documents/Instrument197/197_3_N.pdf (Accessed 8 Aug 2018).

79. van Damme S, Crombez G, Vlaeyen JWS, et al. De Pain Catastrophizing Scale: Psychometrische karakteristieken en normering. Gedragstherapie. 2000;33:209–20.

80. Rodero B, Luciano J V, Montero-Marín J, et al. Perceived injustice in fibromyalgia: Psychometric characteristics of the Injustice Experience Questionnaire and relationship with pain catastrophising and pain acceptance. J Psychosom Res. 2012;73(2):86–91.

81. van Wilgen CP, Nijs J, Don S, et al. The Injustice Experience Questionnaire: Nederlandstalige consensusvertaling [Internet]. 2014. Available from: http://www.paininmotion.be/storage/app/media//materials/IEQ-Dutch.pdf (Accessed 19 Jul 2018).

82. Derogatis LR. BSI brief symptom inventory: Administration, Scoring, and Procedure Manual (4th Ed.). Minneapolis, MN: National Computer Systems;1993.

83. de Beurs E, Zitman FG. The Brief Symptom Inventory (BSI): Reliability and validity of a practical alternative to SCL-90. Maandbl Geestelijke Volksgezond. 2006;61:120–41.

84. Peduzzi P, Concato J, Feinstein AR, et al. Importance of events per independent variable in proportional hazards regression analysis. II. Accuracy and precision of regression estimates. J Clin Epidemiol. 1995;48(12):1503–10.

85. van Wilgen CP, Vuijk PJ, Kregel J, et al. Psychological Distress and Widespread Pain Contribute to the Variance of the Central Sensitization Inventory: A Cross-Sectional Study in Patients with Chronic Pain. Pain Pract. 2018;18(2):239–46.

86. Arendt-Nielsen L, Yarnitsky D. Experimental and Clinical Applications of Quantitative Sensory Testing Applied to Skin, Muscles and Viscera. J Pain. 2009;10(6):556–72.

87. Backonja M-M, Walk D, Edwards RR, et al. Quantitative Sensory Testing in Measurement of Neuropathic Pain Phenomena and Other Sensory Abnormalities. Clin J Pain. 2009;25(7):641–7.

88. Pavlaković G, Petzke F. The Role of Quantitative Sensory Testing in the Evaluation of Musculoskeletal Pain

For peer review only

Conditions. Curr Rheumatol Rep. 2010;12(6):455–61.

89. Krumova EK, Geber C, Westermann A, et al. Neuropathic Pain: Is Quantitative Sensory Testing Helpful? Curr Diab Rep. 2012;12(4):393–402.

90. Uddin Z, MacDermid JC. Quantitative Sensory Testing in Chronic Musculoskeletal Pain. Pain Med. 2016;17(9):1694–703.

91. Martínez-Martínez L-A, Mora T, Vargas A, et al. Sympathetic Nervous System Dysfunction in Fibromyalgia, Chronic Fatigue Syndrome, Irritable Bowel Syndrome, and Interstitial Cystitis. JCR J Clin Rheumatol. 2014;20(3):146–50.

92. Tracy LM, Ioannou L, Baker KS, et al. Meta-analytic evidence for decreased heart rate variability in chronic pain implicating parasympathetic nervous system dysregulation. Pain. 2016;157(1):7–29.

93. Starkweather AR, Heineman A, Storey S, et al. Methods to measure peripheral and central sensitization using quantitative sensory testing: A focus on individuals with low back pain. Appl Nurs Res. 2016;29:237–41.

94. Kuijer PPFM, Gouttebarge V, Brouwer S, et al. Are performance-based measures predictive of work participation in patients with musculoskeletal disorders? A systematic review. Int Arch Occup Environ Health. 2012;85(2):109–23.

95. Reneman MF, Jorritsma W, Schellekens JMH, et al. Concurrent validity of questionnaire and performance-based disability measurements in patients with chronic nonspecific low back pain. J Occup Rehabil. 2002;12(3):119–29.

96. Brouwer S, Dijkstra PU, Stewart RE, et al. Comparing self-report, clinical examination and functional testing in the assessment of work-related limitations in patients with chronic low back pain. Disabil Rehabil. 2005;27(17):999–1005.

97. Caporaso F, Pulkovski N, Sprott H, et al. How well do observed functional limitations explain the variance in Roland Morris scores in patients with chronic non-specific low back pain undergoing physiotherapy? Eur Spine J. 2012;21(Suppl 2):S187–95.

98. Vanhelst J, Gottrand F, Baquet G, et al. Comparative Interinstrument Reliability of Uniaxial and Triaxial Accelerometers in Free-Living Conditions. Percept Mot Skills. 2012;114(2):584–94.

99. Aadland E, Ylvisåker E. Reliability of the Actigraph GT3X+ Accelerometer in Adults under Free-Living Conditions. PLoS One. 2015;10(8):e0134606.

100. Laborde S, Mosley E, Thayer JF. Heart Rate Variability and Cardiac Vagal Tone in Psychophysiological Research – Recommendations for Experiment Planning, Data Analysis, and Data Reporting. Front Psychol. 2017;08:213.

101. Matthews CE, Hagströmer M, Pober DM, et al. Best practices for using physical activity monitors in population-based research. Med Sci Sports Exerc. 2012;44(Suppl 1):S68-76.

102. General Assembly of the World Medical Association. World Medical Association Declaration of Helsinki: Ethical principles for medical research involving human subjects. J Am Coll Dent. 2014;81(3):14–8.

For peer review only

103. Dutch Government. The Medical Research Involving Human Subjects Act, in Dutch: Wet medisch-wetenschappelijk onderzoek met mensen (WMO) [Internet]. Available from: https://wetten.overheid.nl/BWBR0009408/2018-08-01 (Accessed 28 Feb 2019).

For peer review only

FIGURE LEGENDS

Figure 1. Flow-chart of the project procedure.

* , measurements obtained which do not belong to CAU of the Pain Rehabilitation of the CvR – UMCG .

For peer review only

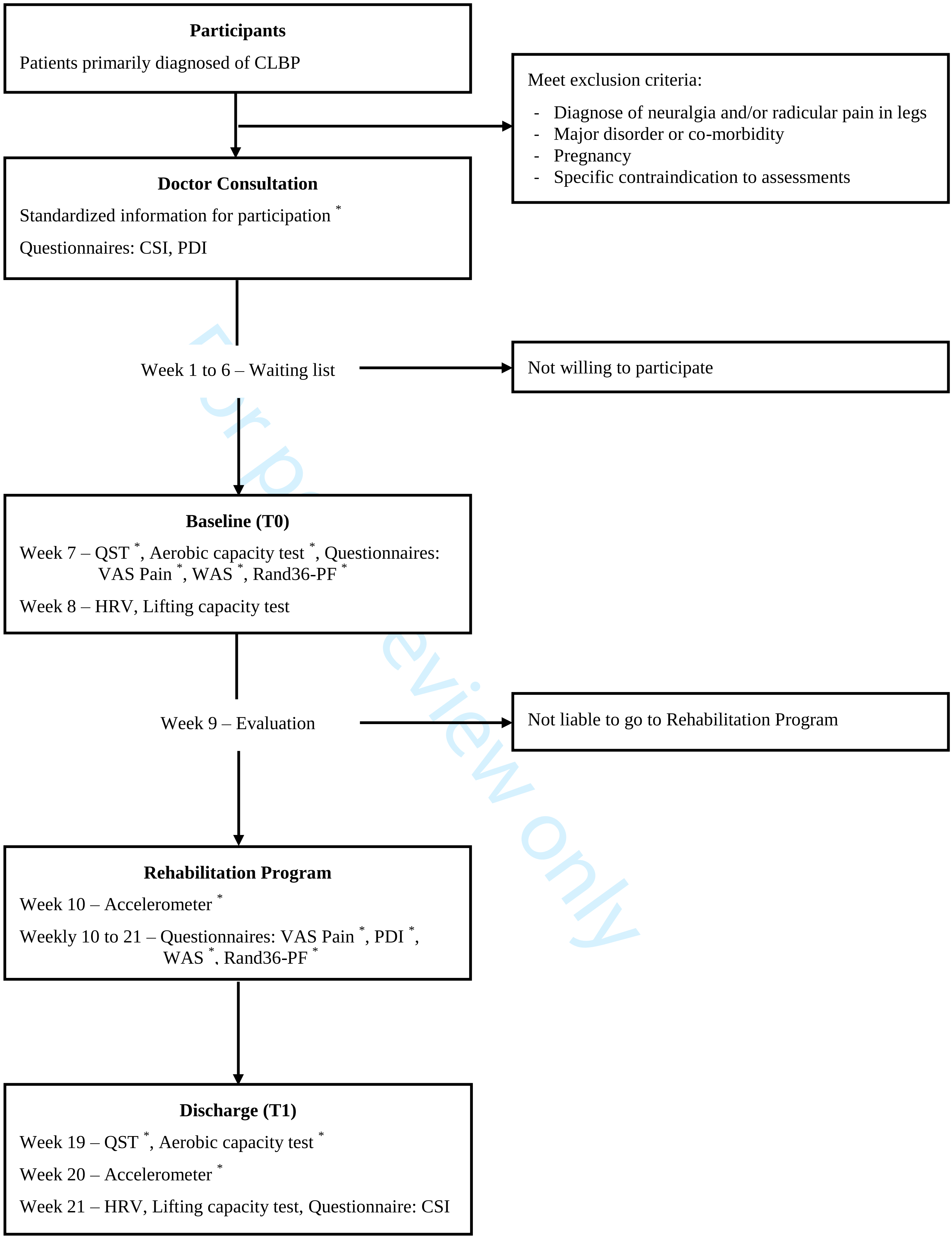
Participants
Patients primarily diagnosed of CLBP
Meet exclusion criteria:
- Diagnose of neuralgia and/or radicular pain in legs
- Major disorder or co-morbidity
- Pregnancy
- Specific contraindication to assessments
Doctor Consultation
Standardized information for participation *
Questionnaires: CSI, PDI
Week 1 to 6 – Waiting list
Not willing to participate
Baseline (T0)
Week 7 – QST *, Aerobic capacity test *, Questionnaires: VAS Pain *, WAS *, Rand36-PF *
Week 8 – HRV, Lifting capacity test
Week 9 – Evaluation
Not liable to go to Rehabilitation Program
Rehabilitation Program
Week 10 – Accelerometer *
Weekly 10 to 21 – Questionnaires: VAS Pain *, PDI *, WAS *, Rand36-PF *
Discharge (T1)
Week 19 – QST *, Aerobic capacity test *
Week 20 – Accelerometer *
Week 21 – HRV, Lifting capacity test, Questionnaire: CSI

SUPPLEMENTARY FILE

Supplemenary material 1. Description of the locations for the QST assessment.

Measuring Location	Description
**1. m. Deltoid – Training location**	In the pars medialis of m. Deltoid, at the contralateral side of the body to the most painful area located; 4 cm below the Acromion.<br>For the CPM test the measurement is at the non-dominant m. Deltoid.
**2. Most painful area**	As stated by the patient. This location is thoroughly described in the form to repeat the measurement in the same exact location at discharge.
**3. Low back – Control location**	Contralateral side of the body mirror-wise to the most painful area located. A specific description of the most painful area is important to identify this location as well.
**4. m. Trapezius – Control location**	In the pars medialis of m. Trapezius, both ipsilateral and contralateral to the most painful area located. At the level of the spine of the Scapula (T3); 4 cm lateral / external to the backbone.
**5. m. Quadriceps – Control location**	In the rectus femoris of m. Quadriceps, both ipsilateral and contralateral to the most painful area located; 15 cm above the upper edge of the Patella.<br>For the CPM test the measurement is at the non-dominant m. Quadriceps.

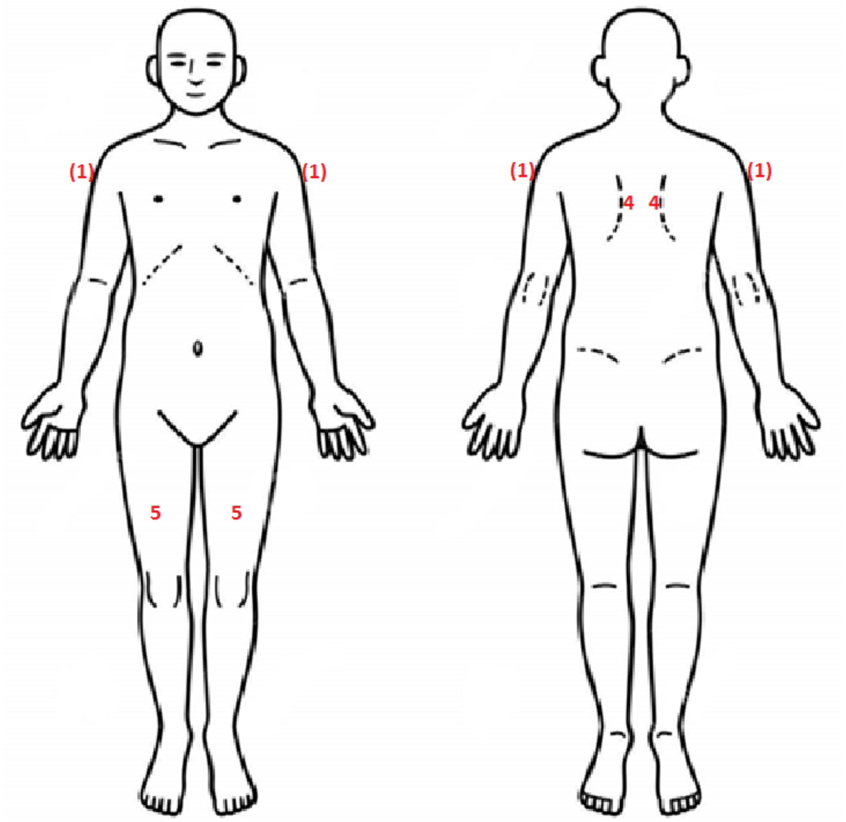

For peer review only - http://bmjopen.bmj.com/site/about/guidelines.xhtml

STANDARD PROTOCOL ITEMS: RECOMMENDATIONS FOR INTERVENTIONAL TRIALS

SPIRIT 2013 Checklist: Recommended items to address in a clinical trial protocol and related documents*

Section / Item	Item No.	Description	Check
**Administrative information**			
Title	1	Descriptive title identifying the study design, population, interventions, and, if applicable, trial acronym	X
Trial registration	2a	Trial identifier and registry name. If not yet registered, name of intended registry	X
	2b	All items from the World Health Organization Trial Registration Data Set	X
Protocol version	3	Date and version identifier	X
Funding	4	Sources and types of financial, material, and other support	X
Roles and responsibilities	5a	Names, affiliations, and roles of protocol contributors	X
	5b	Name and contact information for the trial sponsor	NA
	5c	Role of study sponsor and funders, if any, in study design; collection, management, analysis, and interpretation of data; writing of the report; and the decision to submit the report for publication, including whether they will have ultimate authority over any of these activities	NA
	5d	Composition, roles, and responsibilities of the coordinating centre, steering committee, endpoint adjudication committee, data management team, and other individuals or groups overseeing the trial, if applicable (see Item 21a for data monitoring committee)	NA
**Introduction**			
Background and rationale	6a	Description of research question and justification for undertaking the trial, including summary of relevant studies (published and unpublished) examining benefits and harms for each intervention	X
	6b	Explanation for choice of comparators	X
Objectives	7	Specific objectives or hypotheses	X
Trial design	8	Description of trial design including type of trial (eg, parallel group, crossover, factorial, single group), allocation ratio, and framework (eg, superiority, equivalence, noninferiority, exploratory)	X

For peer review only

**Methods: Participants, interventions, and outcomes**			
Study setting	9	Description of study settings (eg, community clinic, academic hospital) and list of countries where data will be collected. Reference to where list of study sites can be obtained	**X**
Eligibility criteria	10	Inclusion and exclusion criteria for participants. If applicable, eligibility criteria for study centres and individuals who will perform the interventions (eg, surgeons, psychotherapists)	**X**
Interventions	11a	Interventions for each group with sufficient detail to allow replication, including how and when they will be administered	**NA**
	11b	Criteria for discontinuing or modifying allocated interventions for a given trial participant (eg, drug dose change in response to harms, participant request, or improving/worsening disease)	**NA**
	11c	Strategies to improve adherence to intervention protocols, and any procedures for monitoring adherence (eg, drug tablet return, laboratory tests)	**NA**
	11d	Relevant concomitant care and interventions that are permitted or prohibited during the trial	**NA**
Outcomes	12	Primary, secondary, and other outcomes, including the specific measurement variable (eg, systolic blood pressure), analysis metric (eg, change from baseline, final value, time to event), method of aggregation (eg, median, proportion), and time point for each outcome. Explanation of the clinical relevance of chosen efficacy and harm outcomes is strongly recommended	**X**
Participant timeline	13	Time schedule of enrolment, interventions (including any run-ins and washouts), assessments, and visits for participants. A schematic diagram is highly recommended (see Figure)	**X**
Sample size	14	Estimated number of participants needed to achieve study objectives and how it was determined, including clinical and statistical assumptions supporting any sample size calculations	**X**
Recruitment	15	Strategies for achieving adequate participant enrolment to reach target sample size	**X**
**Methods: Assignment of interventions (for controlled trials)**			**NA**
Allocation:			
Sequence generation	16a	Method of generating the allocation sequence (eg, computer-generated random numbers), and list of any factors for stratification. To reduce predictability of a random sequence, details of any planned restriction (eg, blocking) should be provided in a separate document that is unavailable to those who enrol participants or assign interventions	**NA**

Allocation concealment mechanism	16b	Mechanism of implementing the allocation sequence (eg, central telephone; sequentially numbered, opaque, sealed envelopes), describing any steps to conceal the sequence until interventions are assigned	**NA**
Implementation	16c	Who will generate the allocation sequence, who will enrol participants, and who will assign participants to interventions	**NA**
Blinding (masking)	17a	Who will be blinded after assignment to interventions (eg, trial participants, care providers, outcome assessors, data analysts), and how	**NA**
	17b	If blinded, circumstances under which unblinding is permissible, and procedure for revealing a participant's allocated intervention during the trial	**NA**
**Methods: Data collection, management, and analysis**			
Data collection methods	18a	Plans for assessment and collection of outcome, baseline, and other trial data, including any related processes to promote data quality (eg, duplicate measurements, training of assessors) and a description of study instruments (eg, questionnaires, laboratory tests) along with their reliability and validity, if known. Reference to where data collection forms can be found, if not in the protocol	**X**
	18b	Plans to promote participant retention and complete follow-up, including list of any outcome data to be collected for participants who discontinue or deviate from intervention protocols	**NA**
Data management	19	Plans for data entry, coding, security, and storage, including any related processes to promote data quality (eg, double data entry; range checks for data values). Reference to where details of data management procedures can be found, if not in the protocol	**X**
Statistical methods	20a	Statistical methods for analysing primary and secondary outcomes. Reference to where other details of the statistical analysis plan can be found, if not in the protocol	**X**
	20b	Methods for any additional analyses (eg, subgroup and adjusted analyses)	**X**
	20c	Definition of analysis population relating to protocol non-adherence (eg, as randomised analysis), and any statistical methods to handle missing data (eg, multiple imputation)	**X**
**Methods: Monitoring**			**NA**
Data monitoring	21a	Composition of data monitoring committee (DMC); summary of its role and reporting structure; statement of whether it is independent from the sponsor and competing interests; and reference to where further details about its charter can be found, if not in the protocol. Alternatively, an explanation of why a DMC is not needed	**NA**

	21b	Description of any interim analyses and stopping guidelines, including who will have access to these interim results and make the final decision to terminate the trial	**NA**
Harms	22	Plans for collecting, assessing, reporting, and managing solicited and spontaneously reported adverse events and other unintended effects of trial interventions or trial conduct	**NA**
Auditing	23	Frequency and procedures for auditing trial conduct, if any, and whether the process will be independent from investigators and the sponsor	**NA**
**Ethics and dissemination**			
Research ethics approval	24	Plans for seeking research ethics committee/institutional review board (REC/IRB) approval	**X**
Protocol amendments	25	Plans for communicating important protocol modifications (eg, changes to eligibility criteria, outcomes, analyses) to relevant parties (eg, investigators, REC/IRBs, trial participants, trial registries, journals, regulators)	**X**
Consent or assent	26a	Who will obtain informed consent or assent from potential trial participants or authorised surrogates, and how (see Item 32)	**X**
	26b	Additional consent provisions for collection and use of participant data and biological specimens in ancillary studies, if applicable	**NA**
Confidentiality	27	How personal information about potential and enrolled participants will be collected, shared, and maintained in order to protect confidentiality before, during, and after the trial	**X**
Declaration of interests	28	Financial and other competing interests for principal investigators for the overall trial and each study site	**X**
Access to data	29	Statement of who will have access to the final trial dataset, and disclosure of contractual agreements that limit such access for investigators	**X**
Ancillary and post-trial care	30	Provisions, if any, for ancillary and post-trial care, and for compensation to those who suffer harm from trial participation	**NA**
Dissemination policy	31a	Plans for investigators and sponsor to communicate trial results to participants, healthcare professionals, the public, and other relevant groups (eg, via publication, reporting in results databases, or other data sharing arrangements), including any publication restrictions	**X**
	31b	Authorship eligibility guidelines and any intended use of professional writers	**X**
	31c	Plans, if any, for granting public access to the full protocol, participant-level dataset, and statistical code	**NA**

For peer review only

Appendices

Informed consent materials	32	Model consent form and other related documentation given to participants and authorised surrogates	-
Biological specimens	33	Plans for collection, laboratory evaluation, and storage of biological specimens for genetic or molecular analysis in the current trial and for future use in ancillary studies, if applicable	**NA**

*It is strongly recommended that this checklist be read in conjunction with the SPIRIT 2013 Explanation & Elaboration for important clarification on the items. Amendments to the protocol should be tracked and dated. The SPIRIT checklist is copyrighted by the SPIRIT Group under the Creative Commons "Attribution-NonCommercial-NoDerivs 3.0 Unported" license.

For peer review only

BMJ Open

Central Sensitization and Functioning in patients with Chronic Low Back Pain: Protocol for a cross-sectional and cohort study

Journal:	*BMJ Open*
Manuscript ID	bmjopen-2019-031592.R1
Article Type:	Protocol
Date Submitted by the Author:	30-Aug-2019
Complete List of Authors:	Ansuategui Echeita, Jone; University Medical Center Groningen, Rehabilitation Medicine<br>Schiphorst Preuper, Henrica ; University Medical Center Groningen, Rehabilitation Medicine<br>Dekker, Rienk; University Medical Center Groningen, Rehabilitation Medicine; University Medical Center Groningen, Center for Sports Medicine<br>Stuive, Ilse; University Medical Center Groningen , Rehabilitation Medicine<br>Timmerman, Hans; Radboud University Medical Center, Department of Anesthesiology, Pain and Palliative Medicine<br>Wolff, Andre; University Medical Center Groningen, Anesthesiology Pain Center<br>Reneman, Michiel; University Medical Center Groningen, Rehabilitation Medicine
**Primary Subject Heading**:	Rehabilitation medicine
Secondary Subject Heading:	Anaesthesia, Sports and exercise medicine, Occupational and environmental medicine
Keywords:	Hyperalgesia, Autonomic Nervous System, Lifting, Exercise Test, Physical Activity, Disability

SCHOLARONE™
Manuscripts

I, the Submitting Author has the right to grant and does grant on behalf of all authors of the Work (as defined in the below author licence), an exclusive licence and/or a non-exclusive licence for contributions from authors who are: i) UK Crown employees; ii) where BMJ has agreed a CC-BY licence shall apply, and/or iii) in accordance with the terms applicable for US Federal Government officers or employees acting as part of their official duties; on a worldwide, perpetual, irrevocable, royalty-free basis to BMJ Publishing Group Ltd ("BMJ") its licensees and where the relevant Journal is co-owned by BMJ to the co-owners of the Journal, to publish the Work in this journal and any other BMJ products and to exploit all rights, as set out in our licence.

The Submitting Author accepts and understands that any supply made under these terms is made by BMJ to the Submitting Author unless you are acting as an employee on behalf of your employer or a postgraduate student of an affiliated institution which is paying any applicable article publishing charge ("APC") for Open Access articles. Where the Submitting Author wishes to make the Work available on an Open Access basis (and intends to pay the relevant APC), the terms of reuse of such Open Access shall be governed by a Creative Commons licence – details of these licences and which Creative Commons licence will apply to this Work are set out in our licence referred to above.

Other than as permitted in any relevant BMJ Author's Self Archiving Policies, I confirm this Work has not been accepted for publication elsewhere, is not being considered for publication elsewhere and does not duplicate material already published. I confirm all authors consent to publication of this Work and authorise the granting of this licence.

For peer review only

Central Sensitization and Functioning in patients with Chronic Low Back Pain: Protocol for a cross-sectional and cohort study

J. Ansuategui Echeita [1], H.R. Schiphorst Preuper [1], R. Dekker [1], I. Stuive [1], H. Timmerman [2, 3], A.P. Wolff [3], M.F. Reneman [1].

Affiliations:

[1] Department of Rehabilitation Medicine, University of Groningen, University Medical Center Groningen, Groningen, The Netherlands.

[2] Department of Anesthesiology, Pain and Palliative Medicine, Radboud university medical center, Nijmegen, The Netherlands.

[3] Department of Anesthesiology Pain Center, University of Groningen, University Medical Center Groningen, Groningen, The Netherlands.

Corresponding author: Jone Ansuategui Echeita, Department of Rehabilitation Medicine, University Medical Center Groningen, P.O. Box 30.002, 9750 RA Haren, The Netherlands. Email: j.ansuategui.echeita@umcg.nl

For peer review only

ABSTRACT

Introduction. A relevant subsample of patients with chronic low back pain (CLBP) have manifested augmented pain processing, Central Sensitization (CS). Patients with CLBP have limited functioning and participation. Theoretically, physical functioning in patients with CLBP can plausibly be linked to CS; however, evidence to explain such association is scarce. Moreover, there is no gold standard to diagnose CS. The objectives of the study are:

1) To analyze the association between instruments assessing symptoms and signs attributed to CS.
2) To analyze whether symptoms and signs attributed to CS are associated with functioning measurement outcomes.
3) To analyze whether changes (between baseline and discharge) in symptoms and signs attributed to CS are related to changes in each of the functioning measurement outcomes.

Methods and analysis. Cross-sectional and longitudinal observational study with measurements at baseline and discharge of an interdisciplinary rehabilitation program. 115 adult patients with CLBP are calculated to participate. CS measurements: Central Sensitization Inventory, Quantitative Sensory Testing, and Heart-Rate Variability. Functioning measurements: Lifting capacity, Maximal aerobic capacity, Accelerometry, and Reported functioning. Statistical analyses: 1) correlation between CS measurements, 2) multiple regression between functioning (dependent variable) and CS measurements (independent variable), 3) multiple regression between change in scores of functioning (dependent variable) and CS measurements (independent variable); and corrected for sex and age.

Ethics and dissemination. The study obtained the clearance to its implementation from the Medical Research Ethics Committee of the University Medical Center Groningen (METc UMCG) in July 2017. The results will be disseminated through scientific publications in peer-reviewed journals, presentations at relevant conferences, and reports to stakeholders.

Trial registration number. NTR7167.

KEYWORDS

Hyperalgesia, Autonomic Nervous System, Somatosensory, Lifting, Exercise Test, Physical Activity, Disability.

For peer review only

STRENGTHS AND LIMITATIONS OF THIS STUDY

- Novel and comprehensive approaches for the assessment of the presence of CS.
- Extensive amount of measurements from multiple domains for the determination of functioning status.
- Longitudinal study with measurements at baseline and discharge of an interdisciplinary rehabilitation program implemented as 'Care as Usual'.
- Strategies allocated to promote participation and adherence, and to mitigate to the largest extent possible selection bias.
- Statistical analyses are performed with selections of CS and functioning measurements, and these selections may influence study results.

For peer review only

INTRODUCTION

Background

Twenty percent of the general population experiences prolonged or recurrent pain for at least three months, such pain is then considered to be chronic.[1,2] Low back pain is the principal cause of Years Lived with Disability (YLD) and work days lost globally and in Europe,[3] and its sequelae affect 9% of the global population.[4] Moreover, although there are several treatment intervention strategies available for patients with Chronic Low Back Pain (CLBP), treatment effect sizes have only been moderate.[5] Such evidence may explain that 40% of the patients are not satisfied with the treatment received.[2] The economic consequences of chronic pain include direct costs of medical care and pain management treatments, as well as indirect costs of decreased work productivity and sick-leave, and of disability and sickness-retirement costs.[2,6] The indirect costs determine 86-88% of the total costs produced by patients with CLBP.[7,8] In particular, in the Netherlands, the costs of back pain to Dutch society were 3.5 billion in 2007, corresponding to 0.6% of the Gross National Product.[8]

In the past decennia, a relevant subsample of patients with chronic pain, including individuals with CLBP, have shown an increased responsiveness to noxious and non-noxious stimuli, described as Central Sensitization (CS).[9] CS is a result of an imbalance in the nociceptive pathways ("pain pathways") and supraspinal structures due to an amplified facilitation and/or reduced inhibition.[10–12] The phenomenon, CS, is manifested by an amplified pain perception regarding its intensity (hyperalgesia and allodynia), duration (aftersensations and temporal summation), and distribution (expansion of the receptive field); and a reduced conditioned pain modulation.[12] There is currently no gold standard to diagnose CS. The identification of reference clinical symptoms and signs, however, may indicate the presence of CS: disproportionate pain, diffuse distribution, allodynia and hyperalgesia, hypersensitivity unrelated to mechanical stimulus but to environmental sensations (light, temperature, noise, or other stressors), maladaptive psychosocial factors, and low vagal nerve activity.[13–16] Instruments like Central Sensitization Inventory (CSI),[17] Quantitative Sensory Testing (QST) [18,19] and Heart-Rate Variability (HRV);[20] are non-invasive, feasible, and assess different reference clinical symptoms and signs indicative of CS.[12] In absence of a gold standard, these instruments comprehensively measure different aspects of CS.

Chronic pain is a multifactorial condition which has impact on physical or body structures and functions, on psychological processes, and on daily activities and quality of life of individuals.[2,21,22] Limited functioning or capacity to perform everyday tasks, essential for an independent living, affects the mobility and self-care, social relationships, and work and leisure performance of individuals with chronic pain.[23] Individual's functioning is composed of body functions (body level), described by impairments individuals experience; and of activities and participation (personal and social levels, respectively) described by capacity and performance tests.[22,24] Lifting

For peer review only

For peer review only - http://bmjopen.bmj.com/site/about/guidelines.xhtml

capacity, Physical Activity (PA) levels and/or PA distribution or patterns, aerobic capacity, or daily activities participation, are measures that account for the multiple operationalizations of functioning and together provide a more comprehensive overview of different domains of the functioning status.

Patients with CLBP are likely to be less physically active, deconditioned, and disabled. Nowadays much is still unknown about the association of functioning with CLBP, either because research is immature or because the results are conflicting mostly due to differences in methodology.[25-27] Furthermore, the association with CS is scarce due to the limited and diverse use of valid CS measures.[28]The assessment and management of pain of centrally sensitized patients with CLBP may be improved with: 1) an ample selection of capacity and performance tests and experienced impairments measures to determine individual's functioning status; and 2) an extensive assessment of the presence of CS reference clinical symptoms and signs with different instruments. Theoretically, CS can plausibly be linked to physical functioning in individuals with CLBP, because: 1) CS has been identified in a subsample of patients with CLBP. And 2) individuals with CLBP experience limitations in (physical) functioning and participation. Because patients with CS are more reactive to stimuli, the pain experience is enhanced, and, consequently, the presence of CS and/or higher levels of CS could be associated with lower physical functioning and participation in patients with CLBP, and vice versa. However, there is no straightforward evidence of the association between CS and functioning at the moment because both constructs have consistently been reported with different methodologies and outcome parameters.

The present study described in this research protocol is created to fill these existing gaps in knowledge. It intends to assist on the phenotyping of centrally sensitized patients with CLBP by means of the description of mechanisms involved in CS (CS reference clinical symptoms), and to improve the understanding of the CS phenomenon and of the association and influence of CS on multiple physical functioning elements. Including the influence of various physiological and psychological processes related to chronic pain disorders.

Objective(s)

The objectives of the studies covered by this research protocol are:

1. To analyze the association between three instruments assessing the symptoms and signs attributed to CS in patients with CLBP: CSI, QST, and HRV.
2. To analyze whether symptoms and signs attributed to CS are associated with functioning measurement outcomes in patients with CLBP. Specifically, to analyze the association between the three instruments assessing CS (CSI, QST, and HRV) and: Lifting capacity, Aerobic capacity, PA levels and/or PA distribution or patterns, and patient-reported measurements.

For peer review only - http://bmjopen.bmj.com/site/about/guidelines.xhtml

3. To analyze whether changes (between baseline and discharge) in symptoms and signs attributed to CS are related to changes in each of the functioning measurement outcomes in patients with CLBP. Specifically, to analyze whether decreases in CS described as: a lower CSI score, an increase in the pain thresholds (QST), and an increase in parasympathetic/vagal tone (HRV); are related to a better functioning described as: an increase in lifting capacity, an increase in aerobic capacity, an increase in PA levels and/or a change in PA distribution or patterns, and better outcome scores in patient-reported measurements.

METHODS AND ANALYSES

Study design

An observational study project with both cross-sectional and longitudinal designs is being conducted from September 2017 to September 2019 in the Center for Rehabilitation of the University Medical Center Groningen (CvR – UMCG) in The Netherlands.

Patient and Public Involvement

Personnel of the CvR-UMCG collaborate in the study, in particular, the Pain rehabilitation team during patient management and data collection process and the Central exercise lab team on data collection process. The authors wish to express their gratitude to the collaborators and to the patients that participate.

Participants

Consecutive patients between 18 and 65 years of age who are primarily referred to the outpatient Pain Rehabilitation Department of the CvR - UMCG because of chronic primary low back pain [29]and who are admitted for the interdisciplinary pain rehabilitation program are eligible to participate in the study. Additionally, patients are invited to the study if they are mentally competent, can follow instructions. Furthermore, patients are excluded from the study if, based on their file, they: have a specific diagnosis that would better account for the symptoms (e.g. cancer, osteoporosis, and/or spinal fractures), have neuralgia and/or radicular pain in the legs, have some major disorder or co-morbidity that can affect their physical and/or mental functioning (e.g. psychiatric disorders) or that can interfere with the study, are pregnant (or planning to be), have any specific contraindication according to the standards of practice guidelines of the assessments that they are going to be subject to,[30,31] or take any medication that may interfere with

For peer review only

the assessment outcomes such as calcium channel blockers on HRV. CS-related comorbidities (i.e. fibromyalgia, osteoarthritis, or chronic fatigue syndrome) are no reason for exclusion from the study.

Procedures

Measurements are performed as part of 'Care As Usual' (CAU) for outpatients following the Pain Rehabilitation guidelines of the CvR - UMCG. However, not all measurements of the research protocol belong to the battery of assessments of the CvR – UMCG, as pointed out in the legend of Figure 1.

During the first appointment of the patients with the rehabilitation physician, patients are invited to participate in the studies if they are eligible according to the criteria defined for the studies. The eligible patients who are invited to participate and receive information about the study, but have not reacted, are contacted in a week's time to remind them about the study participation. Patients who signed the informed consent fill in forms with their relevant personal information during the baseline assessment, and they are given vouchers for the travel expenses during the discharge assessment. The participating patients follow CAU trajectory with the Pain Rehabilitation team in terms of assessments, screening, and implementation of the interdisciplinary rehabilitation program. As part of the research protocol, the performance of additional assessments and filling of questionnaires on CS and functioning are required. All CS, and objective monitoring and capacity tests are measured at two time points, baseline and discharge: the questionnaire CSI as CAU; the physical assessments lifting capacity and HRV as CAU, and aerobic capacity and QST as additional; and the monitoring of PA with an accelerometer as additional during the first and last week of the rehabilitation program. The pain intensity and functioning questionnaires are measured at 13 time points, baseline and once a week during the 12-week rehabilitation program: PDI as CAU at baseline but as additional during the rehabilitation program, and VAS Pain, WAS and Rand36-PF as additional. A diagram of the project procedure is presented as Figure 1.

(Insert Figure 1 around here)

Measurements

Central Sensitization measurements

To comprehensively assess the symptoms and signs attributed to CS, data is obtained from a questionnaire (CSI) and two physical assessments (HRV and QST).

CSI. The inventory is a self-reported questionnaire with two sections to estimate the severity of CS-related symptoms. Section A is a 25-item questionnaire to assess the presence of the most common CS-related symptoms. Each of the

For peer review only - http://bmjopen.bmj.com/site/about/guidelines.xhtml

questions is measured on a 5-point Likert scale from 0 (never) to 4 (always), and the total score ranges from 0 to 100, where higher scoring is associated with more severe symptomatology. Section B is a 10-item questionnaire to assess the previously diagnosed CS Syndromes (CSS) and/or conditions related to CS. Higher amount of CSS is associated with higher chances of CS being present. This instrument has shown excellent test-retest reliability (ICC = 0.82-0.97) and internal consistency (Cronbach α = 0.87-0.91) in the general population and in patients with chronic pain,[17,32-34] as well as good concurrent validity (sensitivity = 81%, specificity = 75%) for CSS diagnosis within patients with chronic pain.[32] For the purposes of the study the Dutch translation of the CSI is used,[35] which has also shown excellent internal consistency (Cronbach α = 0.91) and test-retest reliability (ICC = 0.91 and 0.88) in controls and in patients with chronic musculoskeletal pain respectively.[36] The psychometric information of CSI in patients with CLBP is not known.

QST. A battery of standardized sensory tests that quantifies the function of the somatosensory system. QST tests the characteristics of various sensation and nociceptive processes as representative of afferent nerve fibers and central pathways performance. The whole assessment lasts for 1h, and it consists of six sensory tests, which are measured in seven different predefined body locations: one training location to familiarize the patient with the tests, the most painful location (as pointed out by the patient), and five control locations. See Supplementary material 1 for full description of locations. The standardized instructions of the sensory tests are read to the patient at each test performance. Of the tests, five consist of mechanical stimuli based on and executed following the QST examination of the German Research Network on Neuropathic Pain (DFNS) protocol,[18] and one for conditioned pain modulation following the Nijmegen-Aalborg Screening QST (NASQ) protocol.[19,37] Overall, the DFNS protocol has shown good test-retest (mean r = 0.86) and inter-observer reliability (mean r = 0.83) in patients with affections in the somatosensory system either with or without pain.[38] For the purpose of avoiding bias due to the order of the measurements and locations, a counterbalancing procedure is established which accounts for the locations and the measurements to be made. This procedure provides three different assessment order groups, all of which begin with the measurements at the training location and end with the conditioned pain modulation test. An additional counterbalancing procedure has been made for allocating patients to these assessment order groups (either 1, 2, or 3); every nine patients a different allocation order is performed to ensure a similar amount of QST assessments per group. All assessors are trained in the QST protocol. The following sensory tests are performed:

- *The Dynamic Mechanical Allodynia (DMA)* to test the presence of abnormal stimulus-evoked pain, is assessed with a soft brush (SENSElab^{TM}, Brush-05, Somedic AB, Hörby, Sweden) that is swiped 1-2 cm over the skin. This is performed on a single repetition and on a train of three repetitions (wind-up response). Whether patients feel the sweeping and its pain intensity with a Numeric Rating Scale (NRS) ranging

For peer review only - http://bmjopen.bmj.com/site/about/guidelines.xhtml

from 0 (no pain) to 100 (most intense pain imaginable) for both the single swipe and the series are recorded in each of the seven locations (see Supplementary material 1).[39,40] The DMA (ratio) is calculated as: DMA = (NRS-3 / NRS-1). The DMA test according to the DFNS protocol has shown to have good test-retest (r = 0.87) and inter-observer reliability (r = 0.79).[38]

- *The Mechanical Pain Threshold (MPT)* to test the ability to perceive sharp, pricking, or stinging stimuli is assessed with a set of standardized weighted pinprick stimulators with seven different intensities (The PinPrick stimulator set, MRC Systems GmbH, Germany) and with a modified method of limits. Starting at the lowest intensity (8mN) this is progressively increased until patients report the stimulus to change from touch to painful (sharp, pricking, or stinging). The intensity of the pinprick which produces the first painful stimulus in mN is recorded in each of the seven locations (see Supplementary material 1).[40-43] The MPT test according to the DFNS protocol has shown to have good test-retest (r = 0.80) and inter-observer reliability (r = 0.80).[38]

- *The Wind-Up Ratio (WUR)* to test the Nervous System's painful stimuli temporal summation or responsiveness to repeated noxious stimuli. Such test is assessed with a suprathreshold pinprick stimulator of 256mN (The PinPrick stimulator set, MRC Systems GmbH, Germany), which is applied on a single repetition and on a train of ten repetitions in an area of $1cm^2$ with intervals of one second (wind-up response). The pain intensity measured with a Numeric Rating Scale (NRS) ranging from 0 (no pain) to 100 (most intense pain imaginable) for both the single repetition and the series are recorded in each of the seven locations (see Supplementary material 1).[44,45] The WUR (ratio) is calculated as: WUR = (NRS-10 / NRS-1). The WUR test according to the DFNS protocol has shown to have poor test-retest (r = 0.67) and inter-observer reliability (r = 0.56).[38]

- *Mechanical Detection Threshold (MDT)* to test the ability to detect light touch, is assessed with a standardized set of von Frey filaments (OptiHair2-Set, Marstock Nervtest, Germany) with a modified method of limits.[46,47] Starting at the intensity of 16mN, the stimulus application is progressively decreased until patients do not report any sensation. The intensity of the filament that is not perceived in mN is recorded in each of the seven locations (see Supplementary material 1).[48] The MDT test according to the DFNS protocol has shown to have good test-retest (r = 0.90) and inter-observer reliability (r = 0.89).[38]

- *Pressure Pain Threshold (PPT)* to test the ability to perceive pressure pain stimuli on muscles, is assessed with an electronic pressure algometer (Force Ten ™ FDX, Wagner Instruments, USA), with which pressure in the contact point ($1cm^2$ tip), is progressively but steadily increased until patients report to feel pressure as painful (burning, drilling, or aching). The intensity in N when pressure becomes painful and its pain intensity with a Numeric Rating Scale (NRS) ranging from 0 (no pain) to 100 (most intense pain imaginable) scale is recorded

For peer review only - http://bmjopen.bmj.com/site/about/guidelines.xhtml

in each of the seven locations (see Supplementary material 1).[18] The PPT test according to the DFNS protocol has shown to have good test-retest (r = 0.88) and inter-observer reliability (r = 0.84).[38]

- *Conditioned Pain Modulation (CPM)* to test the Nervous System's descending pain processing. The CPM test methodology selected for this study follows the recommendations of the EFIC meeting (2013).[49] The pain modulation is assessed by means of PPT tests (Test Stimuli, TS) in a distant location from patients dominant hand (m. Deltoid and m. Quadriceps of the non-dominant side, see Supplementary material 1) before and after immersing the dominant hand in a bucket full with ice and cold water (Cold Conditioning Stimuli, cCS). First, PPT measurements at distant locations are made; then, patients immerse the dominant hand in the bucket for as long as they possibly can with a maximal duration of 3 minutes; finally, PPT measurements are repeated. The PPT results, intensity in N and pain intensity with a Numeric Rating Scale (NRS) ranging from 0 (no pain) to 100 (most intense pain imaginable) scale, in both locations and the time the hand is immersed are recorded. The CPM (%) is calculated as: CPM = [(PPTpost – PPTpre) / PPTpre] ∗ 100; where lower values indicate lower alteration on the nociceptive pathways.[19,37] The CPM test, as the combination of the PPT handheld algometer and cold stimuli, has shown to be one of the most reliable methods with a modest test-retest reliability (ICC = 0.49 and Coefficient of Variation, CV = 63.6%).[50]

HRV. The test provides an indication of the function and balance of the Autonomic Nervous System (ANS), which is responsible for the control of internal functions including self-regulation, adaptability, and resilience capacities.[51] The ANS is susceptible to mental and emotional processes, which in turn can produce an imbalance in self-regulation (affecting among others HR) by an hyper-activation of the sympathetic tone and reduced parasympathetic/vagal tone.[15,17] HRV testing can monitor heart rhythms, or time and pattern variation on HR and inter-beat (RR) intervals, and can be used to assess the function of the ANS.[52,53] The HRV test is performed by assessors trained by the HeartMath institute, and the 5-minute protocol is executed to guarantee the comparison of the results as recommended by the Task Force for short-term recordings.[52] Patients are instructed to sit calmly and breathe normally, while they have an ear pulse sensor placed which is connected to a computer.[54] The following measures are collected and recorded with the emWave PC Software (emWave®, HeartMath Inc., Boulder Creek, California): mean HR in beats per minute (bpm), mean RR interval in ms; time domain measures in ms as Standard Deviation of Normal-to-Normal interval (SDNN) indicator for cyclic components responsible of RR interval variability, Root Mean Square of Successive Differences (RMSSD) for beat-to-beat variance in HR and parasympathetic/vagal tone; frequency domain measures in ms^2/Hz as Very Low Frequency (VLF, 0.0033-0.04 Hz) indicator for long term regulation and sympathetic tone, Low Frequency (LF, 0.04-0.15 Hz) for sympathetic and parasympathetic/vagal tones, and High Frequency (HF,

For peer review only

0.15-0.40 Hz) for parasympathetic/vagal tone related to breathing rate; and Normalized Coherence indicator for HRV stable or regular pattern over time in %, where higher scores mean more harmonic signal.

Physical Functioning measurements

To determine the functioning status of individuals with chronic pain in multiple domains, data is obtained from two physical assessments (lifting and aerobic capacity tests), an accelerometer (PA level), and a battery of reported functioning questionnaires.

Lifting capacity test. The Floor-to-waist lift capacity test is based on the WorkWell Functional Capacity Evaluation (FCE) protocol [55] and is performed to measure patients work functioning or capacity. Such FCE lift test has been shown to have high test–retest (ICC = 0.81) and inter-rater reliability (ICC = 0.76) in patients with CLBP.[56,57] Assessors are trained in the floor-to-waist lift capacity test procedure. Patients are given standardized instruction to repetitively lift a crate with weights from a shelf at waist height to the floor and back. The test begins with a weight that can easily be lifted, progressively the load is increased, and the test ends when the maximum capacity is reached. The final endpoint can be reached due to: cardiac endpoint (85% of maximum heart rate); biomechanical endpoint (unsafe increasing weight because of lack of load handling control); patient endpoint (patient decided to stop); and criterion endpoint (normal end of the test).[58] The maximal load lifted in kg is recorded along with the endpoint reason, and the patient-reported and the assessor-observed mechanical effort as measured with Borg's Category Ratio scale (CR-10).[59] Borg's CR-10 scale ranges from 0 (at rest) to 10 (very, very hard), and has demonstrated to be a valid visual observation of the effort level of patients during their performance of a lifting capacity test.[60]

Aerobic capacity test. The maximal aerobic capacity is assessed with a cardio-pulmonary exercise test (CPET). The test is performed with a cycle ergometer (Ergoselect 200p or Ergoselect 200k, Ergoline, Bitz, Germany) following a defined continuous ramp protocol. Before any cycling begins, patients are interviewed about their daily activities, and their resting heart rhythm and function is recorded with an electrocardiogram (ECG) (assy cam-14, GE medical systems, US). Patients then have an unloaded warm-up of three minutes at 60-70 rotations per minute (rpm) before the test begins. At the start of the test, an experienced exercise physiologist in discussion with a specialized physician or nurse determine the starting workload (25-100 W) and ramp (5-25 W increase per minute) depending on patients estimated fitness level. During the test patients are asked to maintain a constant cadence while the working load is progressively being increased. Also, they are continuously being verbally encouraged to obtain their maximum performance. The maximal performance is determined by various parameters: a temporary loss of strength and energy (= exhaustion), a plateau in the peak oxygen uptake (VO2max), a respiratory exchange ratio (RER) higher than 1.15, and/or a heart-rate (HR) higher than 85% of the predicted maximal HR.[31] Overall, the test lasts for 8-12 minutes and throughout the whole assessment patients are monitored on their cardiac activity with an ECG, on their blood pressure with a cuff

For peer review only

algometer, and on their ventilatory gases on a breath-by-breath basis with a metabolic cart (JAEGER ® Vyntus ® CPX, Jaeger, Germany, Hoechberg). The VO2max in ml/min and per kilogram in ml/min/kg is collected as the mean of VO2 over the last 30 seconds of exercise. Along with VO2max are collected: peak workload in watts, HR at end of test in beats per minute (bpm), breathing frequency (BF) at end of test in breaths per min, expiratory tidal volume (VT) in l, peak energy expenditure in Metabolic Equivalent of Task (MET), test duration in min, and the patient-reported and the assessor-observed mechanical effort as measured with Borg's rating of perceived exertion (RPE) scale. Borg's RPE scale ranges from 6 (no exertion at all) to 20 (maximal exertion) [59] and has shown to be highly correlated with HR (r = 0.74) and blood lactate (r = 0.83),[61] demonstrating to be a reliable instrument to measure work intensity.

PA levels. Objective measurements of PA levels are obtained by means of an accelerometer (ActiGraph GT9X Link, ActiGraph, Pensacola, FL, USA). The accelerometer is a small and light device that monitors wearers' daily PA and energy expenditure. Participating patients wear the accelerometer attached at their hip level for one week continuously during walking hours, in order to ensure the minimum of 4-full-day recording as recommended by activity monitoring reviewers and experts.[62,63] Different algorithms are chosen from the software provided by the manufacturer, ActiLife6 (ActiGraph, Pensacola, FL, USA), to discriminate between low PAs and split patients in three activity intensity categories (sedentary, light, and moderate) [64] and to obtain patients' energy expenditure in kcal [65] and MET.[66] The daily and weekly time in hours and percentage of time spent in each of the activity intensity categories as well as the estimated energy expenditure in both kcal and MET are collected.

Reported Functioning. Data from this domain is obtained from four questionnaires sent to patients and filled by them via internet. All the questionnaires require 10-15 minutes to be filled in.

- *Physical functioning (Rand36 Physical Functioning subscale – Rand36-PF)*. The Rand36 is a survey that measures the impact of health on patients' quality of life, patients are asked to only answer the physical functioning (PF) subscale which looks at the limitations patients experience during their daily activities. Each of the 10 items belonging to the PF subscale have 3-level response choices ranging from 0 (limited a lot) to 100 (not limited at all), the total average score ranges from 0 to 100 too; where a higher score is associated with a greater limitation in performing daily activities. For the purposes of the study the Dutch translation of the Rand36 is used, which has shown good internal consistency (Cronbach α = 0.92) and test-retest reliability (r = 0.72-0.82) in the general population.[67,68]
- *Disability (Pain Disability Index – PDI)*. The index is a 7-item questionnaire which measures the interference of pain with functioning in different daily life activities: family/home responsibilities, recreation, social activity, occupation, sexual behavior, self-care, and life-support activity. Each item ranges from 0 (no interference) to 10

For peer review only - http://bmjopen.bmj.com/site/about/guidelines.xhtml

(total interference), and the total score ranges from 0 to 70; where scoring higher is associated with higher interference in daily life activities. The PDI has shown modest test-retest reliability (r = 0.44) and high internal consistency (Cronbach α = 0.86) in patients with chronic pain.[69] For the purposes of the study the Dutch translation of the PDI was used, which has shown a good internal consistency (Cronbach α = 0.85) and test-retest reliability (ICC = 0.76) in patients with musculoskeletal pain.[70]

- *Work ability (Work Ability Score – WAS)*. The score is a single-item question which compares patient's current work ability compared with their lifetime best. The result is a score ranging from 0 (unable to work) to 10 (best working ability). The single-item question is part of the Work Ability Index (WAI), a questionnaire which measures work ability and has shown to be test-retest reliable (difference between test and re-test = -0.53) in construction workers.[71] The WAS has shown to be highly correlated to the 28 items of the WAI among women on a long-term sick leave (r = 0.87) [72] and among active workers (r = 0.63).[73]

Supplementary measurements

Demographic characteristics. Patients filled in a form with their details when the QST assessment took place. Such included: age, sex, nationality, height, weight, pain characteristics (affected body area, duration and medication, if applicable), educational level, and employment characteristics (work-status and physical work demands per Dictionary of Occupational Titles (DOT) [74]).

Pain-related questionnaires.

- *Pain intensity (Visual Analogue Scale – VAS Pain)*. The scale is a single-item question to assess the current pain intensity in adults. It is a straight line with 'no pain' (score = 0) at one end and 'pain as bad as it could be' (score = 100) at the other, in which patients are asked to mark the line at the level that represents their pain level. The score is the distance between the mark and the 'no pain' end, where scoring higher is associated with greater pain intensity. The VAS Pain has shown good validity (r = 0.62-0.91) and good test-retest reliability (literate r = 0.94 and illiterate r = 0.71) in patients with rheumatic pain conditions.[75] VAS Pain is sent to patients and filled in by them via internet along with the battery of functioning questionnaires (described above); i.e. at baseline and weekly during the interdisciplinary rehabilitation program.

- *Body response to a straining exercise (Pain Response Questionnaire – PRQ).*[76] The questionnaire assesses the pain intensity of the different body locations on an 11-point numeric scale (NRS) ranging from 0 (no pain) to 10 (worst pain imaginable), and the type of pain felt (muscle soreness, other, both, or unknown). Additionally it controls for the use of medication and for any other strenuous physical activity. For the purposes of this study, the body locations measured are predefined to four and the questionnaire is being filled in three occasions:

For peer review only

before the CPET, immediately afterwards, and 24h later. PRQ is filled at the same moment when the aerobic capacity test is performed; i.e. during baseline and discharge assessments.

Psychological screening questionnaires. These questionnaires measure psychosocial features common to CS syndromes that have demonstrated to affect pain experience and functioning.[77] Patients filled in the questionnaires via internet which were sent per CAU procedures by the Pain Rehabilitation team. Such were:

- *Pain Catastrophizing Scale (PCS)*. The 13-item reported questionnaire measures measured in a 5-point Likert scale (0 - not at all, 4 - all the time) the degree to which patients have had catastrophic thoughts or feelings during their pain experience and accounts for three subscales or dimensions: rumination, magnification, and helplessness.[78] For the purposes of the study the Dutch translation of the PCS was used,[79] which has shown good internal consistency (Cronbach α = 0.85-0.91) and test-retest reliability (r = 0.92) among healthy individuals (students) and patients with chronic low back pain.[80]
- *Injustice Experience Questionnaire (IEQ)*. The 12-item reported questionnaire assesses in a 5-point Likert scale from (0 – never, 4 - all the time) four different aspects of the perceived injustice due to injury: severity, blame, unfairness, and irreparability. The questionnaire has shown to have good internal consistency (Cronbach α = 0.92) and test-retest reliability (ICC = 0.98) in a group of patients with fibromyalgia.[81] For the purposes of the study the Dutch translation of the IEQ was used, as translated by the Pain in Motion group.[82]
- *Brief Symptom Inventory (BSI)*. The inventory is a 53-item reported questionnaire which assesses patient's current level of psychological distress with a 5-point Likert scale questions (0 - not at all, 4 - extremely), the amount of symptoms present, and their severity.[83] BSI is composed of nine subscales: somatization, obsession-compulsion, interpersonal sensitivity, depression, anxiety, hostility, phobic anxiety, paranoid ideation, and psychoticism. For the purposes of the study the Dutch translation of the BSI was used, which has shown good internal consistency (Cronbach α = 0.71-0.87) and test-retest reliability (r = 0.71-0.89) among psychiatric out-patient and healthy individual groups.[84]

Sample size

Due to the lack of relevant previous or similar studies, the sample sizes for the several analyses described in this research protocol are estimated a priori and are exploratory as a result.

The following sample size calculations as well as the planned statistical analyses later described will be performed with a proxy for each of the main variables being three variables for CS and four for functioning. Additionally, due to the influence of patient's sex and age in the assessment outcomes, all analyses will include them for a moderator effect.

For peer review only - http://bmjopen.bmj.com/site/about/guidelines.xhtml

Sample size for objective 1: To analyze the association between instruments assessing symptoms and signs attributed to CS.

Correlation analyses will be performed for this objective. Because the correlation between the different CS measurements is expected to be medium, for a correlation coefficient of 0.3, an α error probability of 0.05, and a power of 0.8, a minimum sample size of 84 is calculated (G*Power for Windows, Version 3.1.9.2).

Sample size for objective 2: To analyze whether symptoms and signs attributed to CS are associated with functioning measurement outcomes; and for objective 3: To analyze whether changes (between baseline and discharge) in symptoms and signs attributed to CS are related to changes in each of the functioning measurement outcomes.

Multiple regression analyses will be performed for objectives 2 and 3. Valid multiple regression models require a minimum of 10 participants per variable added.[85] Thus, because minimally three CS measurements and sex and age are required as independent variables, a sample of 50 patients would be necessary. However, since models may also need to be controlled for the confounding effect of other demographic and test-related variables, a minimum of 80 patients is aimed. Finally, patients measured at discharge could be about 30% less than at baseline due to either not following the interdisciplinary rehabilitation program or personal reasons, a sample size of 115 patients may be required in this scenario.

Analyses

Data records from the different tests and questionnaires will be collected and merged into one database. The database management will be managed by the project team and handled according to the internal regulations of the UMCG. The analyses of the study will be performed with SPSS software version 22.0 (IBM Corp., NY).

Before any analysis, data will be prepared:

- Variables need to be calculated: BMI; DMA, WUR, and CPM tests (see Methods section, Main measurements: CS); PDI, Rand36-PF, PCS, BSI, and IEQ questionnaires.
- HRV: data will be pre-processed to correct for any artifacts that may be present at the beginning or at the end of the recording.
- QST: all measurements will be transformed to standard normal distribution (Z-transformation).
- To perform the analyses to respond objective 3, Delta scores are computed (difference in scores between baseline and discharge) for CS, functioning, and those confounder variables which are also measured at the same time points as the main measurements.

For peer review only

Proxies of the main measurements were selected to perform analyses. CS variables are: the sum score of CSI part A, the RMSSD for HRV, and the MPT for QST. Functioning variables are: the maximal weight lifted for lifting capacity, the VO2max per kg for the maximal aerobic capacity, the daily VM counts for PA, and the average score of Rand36-PF for reported physical functioning.

The presence of missing data will be checked. If a variable has more than 10% of missing data cases, its distribution will be further examined. When the distribution of missing data is missing completely at random, analyses with pairwise deletions are planned. If data is not missing completely at random, other suitable solutions will be applied such as multiple imputation.[86] Regarding the presence of outliers, cases at 3 SD from the mean will be considered as outliers. Their influence will be measured with Cook's distance and leverage values. When the outliers are not statistically influential and/or there is rationale for the outlier, no further actions are planned. The descriptive statistics of the sample characteristics and main test results will be at this point calculated; numerical variables as mean and SD in normally distributed data and as median and interquartile range (IQR 25-75) in non-normally distributed data, and categorical variables as counts and percentage of each category level.

Statistical approach for objective 1: To analyze the association between instruments assessing symptoms and signs attributed to CS.

Multiple partial correlation analyses will be performed corrected for potential confounders. CS measurements will be the main variables and sex and age will be covariates. Additional covariates may enter the partial correlations based on their correlation coefficient with CS instruments and if $p<0.01$. Results are reported correlation coefficients (Pearson's r and/or Spearman's rho) and p-values, with and without controlling for covariates. The significance level for all analyses is established at 0.01 level.

Statistical approach for objective 2: To analyze whether symptoms and signs attributed to CS are associated with functioning measurement outcomes.

First multiple correlation analyses, followed by multiple regression analyses with data obtained at baseline will be performed. Each of the main outcomes from functioning measurements will be introduced in the models as dependent variable, CS measurements as independent variables, and sex, age, and other demographic characteristics and/or test-related variables as potential confounders and/or moderators.

Statistical approach for objective 3: To analyze whether changes (between baseline and discharge) in symptoms and signs attributed to CS are related to changes in each of the functioning measurement outcomes.

Similar approach to objective 2 will be performed: first multiple correlation analyses will be performed with data obtained at baseline and discharge, followed by multiple correlation analyses with Delta scores of the data, and finally

For peer review only - http://bmjopen.bmj.com/site/about/guidelines.xhtml

For peer review only

multiple regression analyses again with Delta scores. As in objective 2, each of the main outcomes from functioning measurements will be introduced in the models as dependent variable, CS measurements as independent variables, and sex, age, and other demographic characteristics and/or test-related variables as potential confounders and/or moderators.

Confounders entering the final models of objectives 2 and 3 will be selected based on their partial correlation coefficient with CS and functioning and they will be included if $p \leq 0.10$. All multiple regression models will have the assumptions checked. Results of objectives 2 and 3 will be reported as coefficient (b), confidence intervals (95% CI), p-values, and explained variance, with and without controlling for confounders and/or moderators. The significance level of the correlation analyses is established at 0.01 level and of the multiple regression analyses is established at 0.05 level.

DISCUSSION

Several patients with CLBP have shown common manifestations with other chronic pain conditions, which may evidence the presence of CS in these patients. Yet knowledge on CS is developing, especially in patients with CLBP, and evidence explaining its association with functioning is lacking. This research protocol aims to bridge that gap of knowledge with a novel approach. In the first place, the study (objective 1) will describe the mechanisms of CS in patients with CLBP with instruments that assess CS symptoms from different perspectives. Furthermore, the study (objectives 2 and 3) will also analyze longitudinally whether the presence of CS and changes of CS can be associated with changes physical functioning performance, for which functioning will be assessed with several objective monitoring and capacity tests, and self-reported measurements.

Different CS assessment instruments are selected for the research protocol to examine the CS symptoms and signs attributed to CS. 1) The hypersensitivity unrelated to mechanical stimulus are assessed with CSI. A questionnaire designed to measure CS as it has been shown to be associated to factors known to contribute to CS and is able to identify somatic and emotional symptoms related to CSS.[32,87] 2) The disproportionate pain, diffuse distribution, and allodynia and hyperalgesia, are measured with QST. The QST is a battery of non-invasive standardized tests that assists in the identification of sensory and pain thresholds, the determination of pain intensity perception, and the assessment of the conditioned pain modulation. Its results can allow pain characterization (phenotyping) and processing, and monitoring its progression.[88-92] 3) The low vagal nerve activity is examined with HRV. A non-invasive standardized measure that characterizes the function of the ANS.[20] The somatic and emotional symptoms related to CS syndromes may generate an imbalance between a hyperactivation of the sympathetic tone and a reduction of the parasympathetic/vagal tone.[20,93,94] 4) Psychosocial factors are measured with psychological screening questionnaires for perceived injustice (IEQ), catastrophizing (PCS), and psychological distress (BSI). These

For peer review only - http://bmjopen.bmj.com/site/about/guidelines.xhtml

psychosocial factors are not necessary for the clinical classification of CS but are included in the study, and potentially in the analyses, due to their consistent effect on functioning and on symptom beliefs and pain behaviors common in chronic pain patient including CS syndromes. Some of the CS assessment instruments used in this study are more acknowledged than others in CS literature. CSI is widely used to measure CS as it was designed as a proxy for it; however, its internal consistency and reliability in patients with CLBP has not yet been made available. Also, currently evidence is piling up on the necessity of routinely implementing the elements of QST relevant to patients' conditions in clinical practice due to being able to assess the somatosensory function,[92,95] even though evidence in patients with CLBP is limited. Finally, literature on HRV as an instrument that may reflect an imbalance of ANS in patients with chronic pain conditions, specifically in those patients that have shown an overlap in the CS symptoms, is scarce. In absence of an instrument that can diagnose CS, we are confident that the selected CS assessment instruments are considered best available, and will be able to comprehensively measure from different perspectives the clinical symptoms and signs indicative of CS being present, to provide further insights on the mechanisms of CS and to assist on the phenotyping of centrally sensitized patients with CLBP.

Various functioning assessments are selected for the research protocol to determine the functioning status of centrally sensitized patients with CLBP, including several objective monitoring and capacity tests, and self-reported measurements. 1) The floor-to-waist lifting capacity test is a physical assessment from the FCE that has demonstrated the best predictability for Return-to-Work.[96] 2) The aerobic capacity, expressed as VO2max, can be best assessed with a maximal aerobic capacity test such as the CPET. 3) The PA is measured with an accelerometer, an easy wearable activity-monitoring device that provides objective measurements of PA levels and PA distribution or patterns. 4) Patient-reported functioning questionnaires present patients pain experience, how they perceive or believe their functioning and participation is. Although questionnaires results can differ from their actual physical performance, both measurements complement each other and together they may provide a more comprehensive overview of patients with CLBP.[97-99] Assessments such as lifting capacity and self-reported measurements have been routinely used to determine the functioning status of patients with CLBP. CPET on the other hand, even though it is scientifically superior to a submaximal aerobic capacity test, has barely been implemented with patients with CLBP and, as a result, whether a maximal CPET could be applied systematically with these patients and their pain response to it is unknown. Moreover, even if there is a large amount of literature on PA, the methodology implemented is very broad regarding population groups and/or measuring instruments, which hinders the synthesis of the understanding of PA of patients with CLBP. To bridge the gap, it may be of use an objective measurement of PA and monitored with accelerometers such as those of ActiGraph, who has shown to produce the most reliable devices for recording PA in free-living conditions.[100,101] It is believed that the assessments described are suitable and relevant for the purpose of measuring

For peer review only - http://bmjopen.bmj.com/site/about/guidelines.xhtml

functioning from multiple domains, and that the extended knowledge and the better understanding of the mechanisms involved obtained is not limited to CLBP but can be extended to CS.

Data collected for this project is large and the measurements of CS and functioning collected involve a broad amount of variables, as a result proxies will be used for the statistical analyses. Previous research in CS assessments, on the one hand, and in functioning assessments, on the other, has reported different methodologies and outcome parameters. Reason for which, even if authors have selected the proxy variables based on their relevance and contribution to the purposes of the current studies, the selection of the following measurements as proxies may not be beyond debate: for HRV the RMSSD, as it is a measurement of the parasympathetic/vagal tone which is not influenced by breathing;[102] for QST the MPT, since it measures the perceived skin mechanical hyperalgesia which is one of the main indicators of CS;[12] for the maximal aerobic capacity the VO2max corrected for the body mass of the individual, due to the effect of body mass on peak oxygen uptake; and for PA the daily VM counts, because it characterizes movement duration and intensity.[103]

Notwithstanding the efforts of the team in the design and implementation to obtain the most extensive insights of CS and its association with functioning, it should be considered that patients participating are volunteers; and that the exclusion criteria, although based on patient's file, is dependent on doctor's judgment, which may imply some subjectivity. These circumstances may have some influence in the results; however, their impact of is so far unknown to the authors. Additionally, despite the efforts of the team to promote the study participation and participant retention, it is likely that patient recruitment may be slower than anticipated and that patient may not have a complete follow-up. However, since the measurement period is ample and a withdrawal of 30% of patients has been calculated (see Methods section, Sample size), we remain positive about the sample size and expect to have enough participants to perform analyses with sufficient power. Also, measurements are planned to be within three weeks from the actual baseline and discharge assessments performed by the Pain Rehabilitation team for comparability of the results. The CSI, PDI, and psychological screening questionnaires during baseline assessment are sent to be filled six weeks before the rest of the measurements are obtained, a time distance due to waiting list time. Since no important changes in patients with respect to medication, surgery, or personal conditions are foreseen in the meanwhile and the questionnaires have shown to have good clinimetric properties, these measurements are expected to be comparable to the rest of measurements at baseline.

All things considered, the results obtained from the measurements described in this research protocol can help better describe and understand the mechanisms involved in CS and, of these, the ones affecting the functioning of patients with CLBP; and then be used to optimize improve the effectiveness of pain management interventions. To the authors' knowledge, the study described in this research protocol are the first off its kind and it may become an important

For peer review only

breakthrough for researchers involved in the study of pain, for the treatment strategy of patients with chronic pain by healthcare professionals, and for patient's pain education.

ETHICS AND DISSEMINATION

All participating patients sign the informed consent before any assessment takes place. Formal ethical clearance to perform the study has been obtained from the METC University Medical Center Groningen (The Netherlands) in July 2017 (METC 2016/702). All procedures are in accordance with the ethical standards of the Helsinki Declaration of 1975 as revised in 2014 [104] and with Dutch regulations of the Medical Research Involving Human Subjects Act, in Dutch: Wet Medisch-Wetenschappelijk Onderzoek (WMO), amended in 2018.[105]

The dissemination strategy of the research study results to will be through scientific publications in peer-reviewed journals and presentations in relevant national and international conferences. In addition to it, reports will be send by post to participants and reports and presentations in meetings will be provided to stakeholders.

For peer review only

ABBREVIATIONS

ANS	Autonomic Nervous System
BF	Breathing Frequency at the end of CPET
BMI	Body Mass Index
BSI	Brief Symptom Inventory
CAU	Care As Usual
cCS	cold Conditioning Stimulus
CLBP	Chronic Low Back Pain
CPET	Cardio-Pulmonary Exercise Test
CPM	Conditioned Pain Modulation
CS	Central Sensitization
CSI	Central Sensitization Inventory
CSS	Central Sensitization Syndromes
CvR-UMCG	Center for Rehabilitation of the UMCG; in Dutch: Centrum voor Revalidatie
CR-10 scale	Category Ratio (0-10) scale
DMA	Dynamic Mechanical Allodynia
DFNS	German Research Network on Neuropathic Pain; in German: Deutschen Forschungsverbund Neuropathischer Schmerz
DOT	Dictionary of Occupational Titles
ECG	Electrocardiogram
FCE	Functional Capacity Evaluation
HF	High Frequency
HR	Heart Rate
HRV	Heart Rate Variability
IC	Informed Consent
ICC	Intra-Class Correlation
IEQ	Injustice Experience Questionnaire
LF	Low Frequency
MDT	Mechanical Detection Threshold
MET	Metabolic Equivalent of Task
METc	Medical Research Ethics committee; in Dutch: Medisch Ethische Toetsing commissie
MPT	Mechanical Pain Threshold
NASQ	Nijmegen-Aalborg Screening QST
NRS	Numeric Rating Scale
PA	Physical Activity
PCS	Pain Catastrophizing Scale
PDI	Pain Disability Index
PF	Physical Functioning
PPT	Pressure Pain Threshold
PRQ	Pain Response Questionnaire
QST	Quantitative Sensory Testing
Rand36-PF	Physical Functioning subscale of the Rand36 questionnaire
RER	Respiratory Exchange Ratio

For peer review only

rMSSD	Root Mean Square of Successive Differences between NN intervals
RPE	Rating of Perceived Exertion
RR interval	Inter-beat interval
SDNN	Standard Deviation of Normal-to-Normal (NN) intervals
TS	Test Stimulus
UMCG	Universitair Medisch Centrum Groningen
VAS	Visual Analogue Scale
VLF	Very Low Frequency
VO2max	Peak VO2, Peak oxygen uptake
VT	Expiratory Tidal Volume
WAI	Work Ability Index
WAS	Work Ability Score
WMO	Medical Research Involving Human Subjects Act; in Dutch: Wet Medisch-wetenschappelijk Onderzoek met Mensen
WUR	Wind-Up Ratio
YLD	Years Lived with Disability

For peer review only

STATEMENTS

Author's Contributions: JAE was responsible for the study concept and design, data collection, and manuscript preparation and edit; HRSP, RD, and MR were responsible for the study concept and design, and critical review of the manuscript; IS, HT and APW were responsible for the study design and critical review of the manuscript.

Competing interests: The authors declare that there are no conflicts of interest.

Funding: The Pain Rehabilitation Development Center (in Dutch: Ontwikkelcentrum PijnRevalidatie (OPR)) of the CvR-UMCG funded the research study.

Trial registration: NTR7167, April 16th 2018; retrospectively registered.

Ethics approval: The Medical Research Ethics Committee of the University Medical Center Groningen (METc UMCG) provided a positive reaction in July 2017 to the development of the research study "*Central Sensitization and Functioning in patients with Chronic Low Back Pain*" (METc 2016/702). Its first amendment was approved in October 2017 and its second amendment in August 2018.

For peer review only

REFERENCES

1. The Joint Task Force (JTF). ICD-11 Beta Draft - Mortality and Morbidity Statistics[Internet]. Available from: https://icd.who.int/browse11/l-m/en#/ (Accessed 24 May 2018).
2. Breivik H, Collett B, Ventafridda V, et al. Survey of chronic pain in Europe: Prevalence, impact on daily life, and treatment. Eur J Pain. 2006;10(4):287–333.
3. Hoy D, March L, Brooks P, et al. The global burden of low back pain: estimates from the Global Burden of Disease 2010 study. Ann Rheum Dis. 2014;73(6):968–74.
4. Vos T, Flaxman AD, Naghavi M, et al. Years lived with disability (YLDs) for 1160 sequelae of 289 diseases and injuries 1990–2010: A systematic analysis for the Global Burden of Disease Study 2010. Lancet. 2012;380(9859):2163–96.
5. Foster NE, Anema JR, Cherkin D, et al. Prevention and treatment of low back pain: evidence, challenges, and promising directions. Lancet. 2018;391(10137):2368–83.
6. Boonen A, van den Heuvel R, van Tubergen A, et al. Large differences in cost of illness and wellbeing between patients with fibromyalgia, chronic low back pain, or ankylosing spondylitis. Ann Rheum Dis. 2005;64:396–402.
7. Dagenais S, Caro J, Haldeman S. A systematic review of low back pain cost of illness studies in the United States and internationally. Spine J. 2008;8(1):8–20.
8. Lambeek LC, van Tulder MW, Swinkels ICS, et al. The Trend in Total Cost of Back Pain in the Netherlands in the Period 2002 to 2007. Spine. 2011;36(13):1050–8.
9. Giesecke T, Gracely RH, Grant MAB, et al. Evidence of augmented central pain processing in idiopathic chronic low back pain. Arthritis Rheum. 2004;50(2):613–23.
10. Woolf CJ. Central sensitization: implications for the diagnosis and treatment of pain. Pain. 2011;152(3 Suppl.):S2–15.
11. Arendt-Nielsen L, Morlion B, Perrot S, et al. Assessment and manifestation of central sensitisation across different chronic pain conditions. Eur J Pain. 2018;22(2):216–41.
12. den Boer C, Dries L, Terluin B, et al. Central sensitization in chronic pain and medically unexplained symptom research: A systematic review of definitions, operationalizations and measurement instruments. J Psychosom Res. 2019;117:32–40.
13. Nijs J, Torres-Cueco R, van Wilgen P, et al. Applying Modern Pain Neuroscience in Clinical Practice: Criteria for the Classification of Central Sensitization Pain. Pain Physician. 2014;17(5):447–57.
14. Smart KM, Blake C, Staines A, et al. The Discriminative Validity of “Nociceptive,” “Peripheral Neuropathic,” and “Central Sensitization” as Mechanisms-based Classifications of Musculoskeletal Pain. Clin J Pain. 2011;27(8):655–

For peer review only

63.

15. De Couck M, Nijs J, Gidron Y. You May Need a Nerve to Treat Pain: The Neurobiological Rationale for Vagal Nerve Activation in Pain Management. Clin J Pain. 2014 ;30(12):1099–105.

16. Smart KM, Blake C, Staines A, et al. Mechanisms-based classifications of musculoskeletal pain: Part 1 of 3: Symptoms and signs of central sensitisation in patients with low back (±leg) pain. Man Ther. 2012; 17(4), 336–44.

17. Mayer TG, Neblett R, Cohen H, et al. The development and psychometric validation of the central sensitization inventory. Pain Pract. 2012;12(4):276–85.

18. Rolke R, Baron R, Maier C, et al. Quantitative sensory testing in the German Research Network on Neuropathic Pain (DFNS): Standardized protocol and reference values. Pain. 2006;123(3):231–43.

19. Wilder-Smith O. A paradigm-shift in pain medicine: implementing a systematic approach to altered pain processing in everyday clinical practice based on quantitative sensory testing. Center for Sensory-Motor Interaction (SMI), Department of Health Science and Technology, Aalborg University. 2013:76p.

20. McCraty R, Atkinson M, Tomasino D, et al. The coherent heart: heartbrain interactions, psychophysiological coherence, and the emergence of system-wide order. Boulder Creek, CA: Institute of Heartmath; 2009.

21. O'Brien T, Breivik H. The impact of chronic pain — European patients' perspective over 12 months. Scand J Pain. 2012;3(1):23–9.

22. Nugraha B, Gutenbrunner C, Barke A, et al. The IASP classification of chronic pain for ICD-11. Pain. 2019;160(1):88–94.

23. Cieza A, Stucki G, Weigl M, et al. ICF Core Sets for low back pain. J Rehabil Med. 2004; Suppl. 44:69–74.

24. Schneidert M, Hurst R, Miller J, et al. The role of Environment in the International Classification of Functioning, Disability and Health (ICF). Disabil Rehabil. 2003;25(11–12):588–95.

25. Verbunt JA, Seelen HA, Vlaeyen JW, et al. Disuse and deconditioning in chronic low back pain: concepts and hypotheses on contributing mechanisms. Eur J Pain. 2003;7(1):9–21.

26. Ryan CG, Grant PM, Dall PM, et al. Individuals with chronic low back pain have a lower level, and an altered pattern, of physical activity compared with matched controls: An observational study. Aust J Physiother. 2009;55(1):53–8.

27. van Dieën JH, Reeves NP, Kawchuk G, et al. Motor Control Changes in Low-Back Pain: Divergence in Presentations and Mechanisms. J Orthop Sport Phys Ther. 2018;1–24.

28. Hübscher M, Moloney N, Leaver A, et al. Relationship between quantitative sensory testing and pain or disability in people with spinal pain—A systematic review and meta-analysis. Pain. 2013; 154(9), 1497–504.

29. IASP Taskforce for the Classification of Chronic Pain. The IASP classification of chronic pain for ICD-11: chronic primary pain. Pain. 2019; 160(1); 28-37.

For peer review only

30. Hart DL, Isernhagen SJ, Matheson LN. Guidelines for Functional Capacity Evaluation of People With Medical Conditions. J Orthop Sport Phys Ther. 1993;18(6):682–6.

31. American Thoracic Society, American College of Chest Physicians. ATS/ACCP Statement on Cardiopulmonary Exercise Testing. Am J Respir Crit Care Med. 2003;167(2):211–77.

32. Neblett R, Cohen H, Choi Y, et al. The Central Sensitization Inventory (CSI): establishing clinically significant values for identifying central sensitivity syndromes in an outpatient chronic pain sample. J Pain. 2013;14(5):438–45.

33. Neblett R, Hartzell MM, Mayer TG, et al. Establishing Clinically Relevant Severity Levels for the Central Sensitization Inventory. Pain Pract. 2017;17(2):166–75.

34. Scerbo T, Colasurdo J, Dunn S, et al. Measurement Properties of the Central Sensitization Inventory: A Systematic Review. Pain Pract. 2018;18(4):544–54.

35. van Wilgen CP, Meeus M, Descheemaeker F, et al. Central Sensitization Inventory – Nederlandse consensusvertaling [Internet]. 2013. Available from: http://www.paininmotion.be/storage/app/media//materials/CSI-consensusvertaling.pdf (Accessed 19 Jul 2018).

36. Kregel J, Vuijk PJ, Descheemaeker F, et al. The Dutch Central Sensitization Inventory (CSI). Clin J Pain. 2016;32(7):624–30.

37. Timmerman H, Wilder-Smith O, van Weel C, et al. Detecting the neuropathic pain component in the clinical setting: a study protocol for validation of screening instruments for the presence of a neuropathic pain component. BMC Neurol. 2014;14:94–102.

38. Geber C, Klein T, Azad S, et al. Test–retest and interobserver reliability of quantitative sensory testing according to the protocol of the German Research Network on Neuropathic Pain (DFNS): A multi-centre study. Pain. 2011;152(3):548–56.

39. LaMotte RH, Shain CN, Simone DA, et al. Neurogenic hyperalgesia: psychophysical studies of underlying mechanisms. J Neurophysiol. 1991;66(1):190–211.

40. Ziegler EA, Magerl W, Meyer RA, et al. Secondary hyperalgesia to punctate mechanical stimuli. Brain. 1999;122(12):2245–57.

41. Chan AW, MacFarlane IA, Bowsher D, et al. Weighted needle pinprick sensory thresholds: a simple test of sensory function in diabetic peripheral neuropathy. J Neurol Neurosurg Psychiatry. 1992;55(1):56–9.

42. Greenspan JD, McGillis SLB. Thresholds for the Perception of Pressure, Sharpness, and Mechanically Evoked Cutaneous Pain: Effects of Laterality and Repeated Testing. Somatosens Mot Res. 1994;11(4):311–7.

43. Hampf G, Bowsher D, Wells C, et al. Sensory and autonomic measurements in idiopathic trigeminal neuralgia before and after radiofrequency thermocoagulation: differentiation from some other causes of facial pain. Pain.

For peer review only

1990;40(3):241–8.

44. Price DD, Hu JW, Dubner R, et al. Peripheral suppression of first pain and central summation of second pain evoked by noxious heat pulses. Pain. 1977;3(1):57–68.

45. Magerl W, Wilk SH, Treede R-D. Secondary hyperalgesia and perceptual wind-up following intradermal injection of capsaicin in humans. Pain. 1998;74(2–3):257–68.

46. Weinstein S. Intensive and extensive aspects of tactile sensitivity as a function of body part, sex and laterality. In: Kenshalo DR, ed. The skin senses: Proceedings. Springfield, IL, US: Charles C Thomas. 1968:195–222.

47. Fruhstorfer H, Gross W, Selbmann O. von Frey hairs: new materials for a new design. Eur J Pain. 2001;5(3):341–2.

48. Baumgärtner U, Magerl W, Klein T, et al. Neurogenic hyperalgesia versus painful hypoalgesia: two distinct mechanisms of neuropathic pain. Pain. 2002;96(1–2):141–51.

49. Yarnitsky D, Bouhassira D, Drewes AM, et al. Recommendations on practice of conditioned pain modulation (CPM) testing. Eur J Pain. 2015;19(6):805–6.

50. Imai Y, Petersen KK, Mørch CD, et al. Comparing test–retest reliability and magnitude of conditioned pain modulation using different combinations of test and conditioning stimuli. Somatosens Mot Res. 2016;33(3–4):169–77.

51. McCorry LK. Physiology of the autonomic nervous system. Am J Pharm Educ. 2007;71(4):78–88.

52. Task Force of the European Society of Cardiology and the North American Society of Pacing and Electrophysiology. Heart rate variability, standards of measurement, physiological interpretation, and clinical use. Circulation. 1996;93(5):1043–65.

53. McCraty R, Shaffer F. Heart Rate Variability: New Perspectives on Physiological Mechanisms, Assessment of Self-regulatory Capacity, and Health Risk. Glob Adv Heal Med. 2015;4(1):46–61.

54. HeartMath Inc. emWave Pro Plus Assessments [Internet]. 2018. Available from: https://heartcloud.com/library/asmts.html (Accessed 27 Feb 2019).

55. Isernhagen SJ, Hart DL, Matheson LM. Reliability of independent observer judgments of level of lift effort in a kinesiophysical Functional Capacity Evaluation. Work. 1999;12(2):145–50.

56. de Baets S, Calders P, Schalley N, et al. Updating the Evidence on Functional Capacity Evaluation Methods: A Systematic Review. J Occup Rehabil. 2018;28(3):418–28.

57. Brouwer S, Reneman MF, Dijkstra PU, et al. Test–Retest Reliability of the Isernhagen Work Systems Functional Capacity Evaluation in Patients with Chronic Low Back Pain. J Occup Rehabil. 2003;13(4):207–18.

58. Soer R, van der Schans CP, Geertzen JH, et al. Normative Values for a Functional Capacity Evaluation. Arch Phys Med Rehabil. 2009;90(10):1785–94.

59. Borg G. Borg's perceived exertion and pain scales.. Champaign, IL, US: Human Kinetics; 1998.

For peer review only

60. Reneman MF, Fokkens AS, Dijkstra PU, et al. Testing Lifting Capacity: Validity of Determining Effort Level by Means of Observation. Spine. 2005;30(2):e40–6.

61. Scherr J, Wolfarth B, Christle JW, et al. Associations between Borg's rating of perceived exertion and physiological measures of exercise intensity. Eur J Appl Physiol. 2013;113(1):147–55.

62. Trost SG, McIver KL, Pate RR. Conducting Accelerometer-Based Activity Assessments in Field-Based Research. Med Sci Sports Exerc. 2005;37(11 Suppl.):S531–43.

63. Migueles JH, Cadenas-Sanchez C, Ekelund U, et al. Accelerometer Data Collection and Processing Criteria to Assess Physical Activity and Other Outcomes: A Systematic Review and Practical Considerations. Sport Med. 2017;47(9):1821–45.

64. Keadle SK, Shiroma EJ, Freedson PS, et al. Impact of accelerometer data processing decisions on the sample size, wear time and physical activity level of a large cohort study. BMC Public Health. 2014;14(1):1210.

65. Sasaki JE, John D, Freedson PS. Validation and comparison of ActiGraph activity monitors. J Sci Med Sport. 2011;14(5):411–6.

66. Crouter SE, Kuffel E, Haas JD, et al. Refined two-regression model for the ActiGraph accelerometer. Med Sci Sports Exerc. 2010;42(5):1029–37.

67. van der Zee KI, Sanderman R. RAND-36. umcg.nl [Internet]. Available from: https://www.umcg.nl/SiteCollectionDocuments/research/institutes/SHARE/assessment tools/handleiding_rand36_2e_druk.pdf (Accessed 28 Jun 2018).

68. van der Zee KI, Sanderman R, Heyink JW, et al. Psychometric qualities of the rand 36-item health survey 1.0: A multidimensional measure of general health status. Int J Behav Med. 1996;3(2):104–22.

69. Tait RC, Chibnall JT, Krause S. The Pain Disability Index: psychometric properties. Pain. 1990;40(2):171–82.

70. Soer R, Köke AJA, Vroomen PCAJ, et al. Extensive Validation of the Pain Disability Index in 3 Groups of Patients With Musculoskeletal Pain. Spine. 2013;38(9):e562–8.

71. de Zwart BCH, Frings-Dresen MHW, van Duivenbooden JC. Test-retest reliability of the Work Ability Index questionnaire. Occup Med. 2002;52(4):177–81.

72. Ahlstrom L, Grimby-Ekman A, Hagberg M, et al. The work ability index and single-item question: associations with sick leave, symptoms, and health – A prospective study of women on long-term sick leave. Scand J Work Environ Heal. 2010;36(5):404–12.

73. El Fassi M, Bocquet V, Majery N, et al. Work ability assessment in a worker population: comparison and determinants of Work Ability Index and Work Ability score. BMC Public Health. 2013;13:305–14.

74. U.S. Department of Labor, Employment and Training Administration. Dictionary of Occupational Titles. 4th ed. Washington; 1991.

For peer review only

75. Hawker GA, Mian S, Kendzerska T, et al. Measures of adult pain: Visual Analog Scale for Pain (VAS Pain), Numeric Rating Scale for Pain (NRS Pain), McGill Pain Questionnaire (MPQ), Short-Form McGill Pain Questionnaire (SF-MPQ), Chronic Pain Grade Scale (CPGS), Short Form-36 Bodily Pain Scale (SF36 BPS), and Measure of Intermittent and Constant Osteoarthritis Pain (ICOAP). Arthritis Care Res. 2011;63(S11):S240–52.
76. Soer R, Groothoff JW, Geertzen JHB, et al. Pain Response of Healthy Workers Following a Functional Capacity Evaluation and Implications for Clinical Interpretation. J Occup Rehabil. 2008;18(3):290–8.
77. Adams LM, Turk DC. Psychosocial Factors and Central Sensitivity Syndromes. Curr Rheumatol Rev. 2015 ; 11(2): 96–108.
78. Sullivan MJL, Bishop SR, Pivik J. The pain catastrophizing scale: development and validation. Psychol Assess. 1995;7(4):524–32.
79. Crombez G, Vlaeyen JWS. Pain Catastrophizing Scale (PCS) - Dutch Version (PCS-DV) [Internet]. 1996. Available from: https://meetinstrumentenzorg.blob.core.windows.net/test-documents/Instrument197/197_3_N.pdf (Accessed 8 Aug 2018).
80. van Damme S, Crombez G, Vlaeyen JWS, et al. De Pain Catastrophizing Scale: Psychometrische karakteristieken en normering. Gedragstherapie. 2000;33:209–20.
81. Rodero B, Luciano J V, Montero-Marín J, et al. Perceived injustice in fibromyalgia: Psychometric characteristics of the Injustice Experience Questionnaire and relationship with pain catastrophising and pain acceptance. J Psychosom Res. 2012;73(2):86–91.
82. van Wilgen CP, Nijs J, Don S, et al. The Injustice Experience Questionnaire: Nederlandstalige consensusvertaling [Internet]. 2014. Available from: http://www.paininmotion.be/storage/app/media//materials/IEQ-Dutch.pdf (Accessed 19 Jul 2018).
83. Derogatis LR. BSI brief symptom inventory: Administration, Scoring, and Procedure Manual (4th Ed.). Minneapolis, MN: National Computer Systems;1993.
84. de Beurs E, Zitman FG. The Brief Symptom Inventory (BSI): Reliability and validity of a practical alternative to SCL-90. Maandbl Geestelijke Volksgezond. 2006;61:120–41.
85. Peduzzi P, Concato J, Feinstein AR, et al. Importance of events per independent variable in proportional hazards regression analysis. II. Accuracy and precision of regression estimates. J Clin Epidemiol. 1995;48(12):1503–10.
86. van Ginkel JR, Linting M, Rippe RC, et al. Rebutting Existing Misconceptions About Multiple Imputation as a Method for Handling Missing Data. J Pers Assess. 2019; 1-12.
87. van Wilgen CP, Vuijk PJ, Kregel J, et al. Psychological Distress and Widespread Pain Contribute to the Variance of the Central Sensitization Inventory: A Cross-Sectional Study in Patients with Chronic Pain. Pain Pract. 2018;18(2):239–46.

For peer review only

88. Arendt-Nielsen L, Yarnitsky D. Experimental and Clinical Applications of Quantitative Sensory Testing Applied to Skin, Muscles and Viscera. J Pain. 2009;10(6):556–72.

89. Backonja M-M, Walk D, Edwards RR, et al. Quantitative Sensory Testing in Measurement of Neuropathic Pain Phenomena and Other Sensory Abnormalities. Clin J Pain. 2009;25(7):641–7.

90. Pavlaković G, Petzke F. The Role of Quantitative Sensory Testing in the Evaluation of Musculoskeletal Pain Conditions. Curr Rheumatol Rep. 2010;12(6):455–61.

91. Krumova EK, Geber C, Westermann A, et al. Neuropathic Pain: Is Quantitative Sensory Testing Helpful? Curr Diab Rep. 2012;12(4):393–402.

92. Uddin Z, MacDermid JC. Quantitative Sensory Testing in Chronic Musculoskeletal Pain. Pain Med. 2016;17(9):1694–703.

93. Martínez-Martínez L-A, Mora T, Vargas A, et al. Sympathetic Nervous System Dysfunction in Fibromyalgia, Chronic Fatigue Syndrome, Irritable Bowel Syndrome, and Interstitial Cystitis. JCR J Clin Rheumatol. 2014;20(3):146–50.

94. Tracy LM, Ioannou L, Baker KS, et al. Meta-analytic evidence for decreased heart rate variability in chronic pain implicating parasympathetic nervous system dysregulation. Pain. 2016;157(1):7–29.

95. Starkweather AR, Heineman A, Storey S, et al. Methods to measure peripheral and central sensitization using quantitative sensory testing: A focus on individuals with low back pain. Appl Nurs Res. 2016;29:237–41.

96. Kuijer PPFM, Gouttebarge V, Brouwer S, et al. Are performance-based measures predictive of work participation in patients with musculoskeletal disorders? A systematic review. Int Arch Occup Environ Health. 2012;85(2):109–23.

97. Reneman MF, Jorritsma W, Schellekens JMH, et al. Concurrent validity of questionnaire and performance-based disability measurements in patients with chronic nonspecific low back pain. J Occup Rehabil. 2002;12(3):119–29.

98. Brouwer S, Dijkstra PU, Stewart RE, et al. Comparing self-report, clinical examination and functional testing in the assessment of work-related limitations in patients with chronic low back pain. Disabil Rehabil. 2005;27(17):999–1005.

99. Caporaso F, Pulkovski N, Sprott H, et al. How well do observed functional limitations explain the variance in Roland Morris scores in patients with chronic non-specific low back pain undergoing physiotherapy? Eur Spine J. 2012;21(Suppl 2):S187–95.

100. Vanhelst J, Gottrand F, Baquet G, et al. Comparative Interinstrument Reliability of Uniaxial and Triaxial Accelerometers in Free-Living Conditions. Percept Mot Skills. 2012;114(2):584–94.

101. Aadland E, Ylvisåker E. Reliability of the Actigraph GT3X+ Accelerometer in Adults under Free-Living Conditions. PLoS One. 2015;10(8):e0134606.

102. Laborde S, Mosley E, Thayer JF. Heart Rate Variability and Cardiac Vagal Tone in Psychophysiological Research –

For peer review only

Recommendations for Experiment Planning, Data Analysis, and Data Reporting. Front Psychol. 2017;08:213.

103. Matthews CE, Hagströmer M, Pober DM, et al. Best practices for using physical activity monitors in population-based research. Med Sci Sports Exerc. 2012;44(Suppl 1):S68-76.

104. General Assembly of the World Medical Association. World Medical Association Declaration of Helsinki: Ethical principles for medical research involving human subjects. J Am Coll Dent. 2014;81(3):14–8.

105. Dutch Government. The Medical Research Involving Human Subjects Act, in Dutch: Wet medisch-wetenschappelijk onderzoek met mensen (WMO) [Internet]. Available from: https://wetten.overheid.nl/BWBR0009408/2018-08-01 (Accessed 28 Feb 2019).

For peer review only

FIGURE LEGENDS

Figure 1. Flow-chart of the project procedure.

* , measurements obtained which do not belong to CAU of the Pain Rehabilitation of the CvR – UMCG .

For peer review only

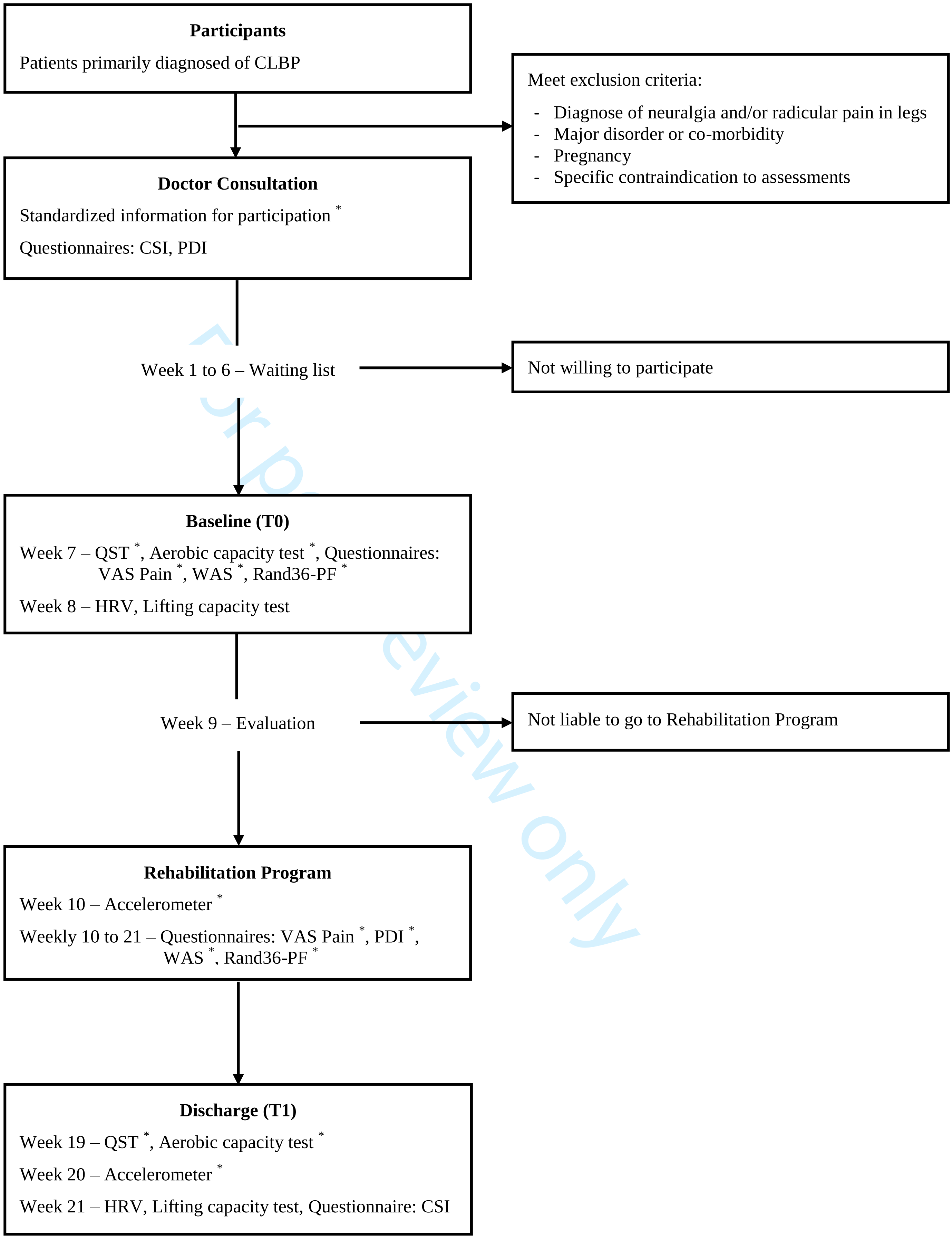

For peer review only

SUPPLEMENTARY FILE

Supplemenary material 1. Description of the locations for the QST assessment.

Measuring Location	Description
**1. m. Deltoid – Training location**	In the pars medialis of m. Deltoid, at the contralateral side of the body to the most painful area located; 4 cm below the Acromion. For the CPM test the measurement is at the non-dominant m. Deltoid.
**2. Most painful area**	As stated by the patient. This location is thoroughly described in the form to repeat the measurement in the same exact location at discharge.
**3. Low back – Control location**	Contralateral side of the body mirror-wise to the most painful area located. A specific description of the most painful area is important to identify this location as well.
**4. m. Trapezius – Control location**	In the pars medialis of m. Trapezius, both ipsilateral and contralateral to the most painful area located. At the level of the spine of the Scapula (T3); 4 cm lateral / external to the backbone.
**5. m. Quadriceps – Control location**	In the rectus femoris of m. Quadriceps, both ipsilateral and contralateral to the most painful area located; 15 cm above the upper edge of the Patella. For the CPM test the measurement is at the non-dominant m. Quadriceps.

For peer review

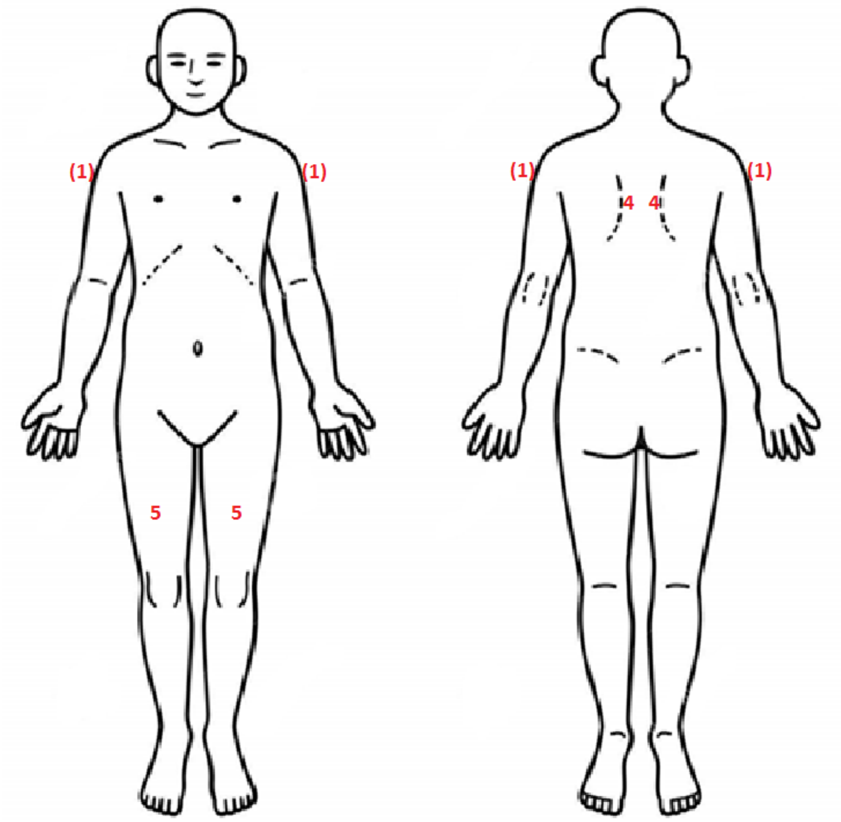

SPIRIT — Standard Protocol Items: Recommendations for Interventional Trials

SPIRIT 2013 Checklist: Recommended items to address in a clinical trial protocol and related documents*

Section / Item	Item No.	Description	Check
**Administrative information**			
Title	1	Descriptive title identifying the study design, population, interventions, and, if applicable, trial acronym	X
Trial registration	2a	Trial identifier and registry name. If not yet registered, name of intended registry	X
	2b	All items from the World Health Organization Trial Registration Data Set	X
Protocol version	3	Date and version identifier	X
Funding	4	Sources and types of financial, material, and other support	X
Roles and responsibilities	5a	Names, affiliations, and roles of protocol contributors	X
	5b	Name and contact information for the trial sponsor	NA
	5c	Role of study sponsor and funders, if any, in study design; collection, management, analysis, and interpretation of data; writing of the report; and the decision to submit the report for publication, including whether they will have ultimate authority over any of these activities	NA
	5d	Composition, roles, and responsibilities of the coordinating centre, steering committee, endpoint adjudication committee, data management team, and other individuals or groups overseeing the trial, if applicable (see Item 21a for data monitoring committee)	NA
**Introduction**			
Background and rationale	6a	Description of research question and justification for undertaking the trial, including summary of relevant studies (published and unpublished) examining benefits and harms for each intervention	X
	6b	Explanation for choice of comparators	X
Objectives	7	Specific objectives or hypotheses	X
Trial design	8	Description of trial design including type of trial (eg, parallel group, crossover, factorial, single group), allocation ratio, and framework (eg, superiority, equivalence, noninferiority, exploratory)	X

For peer review only - http://bmjopen.bmj.com/site/about/guidelines.xhtml

**Methods: Participants, interventions, and outcomes**			
Study setting	9	Description of study settings (eg, community clinic, academic hospital) and list of countries where data will be collected. Reference to where list of study sites can be obtained	**X**
Eligibility criteria	10	Inclusion and exclusion criteria for participants. If applicable, eligibility criteria for study centres and individuals who will perform the interventions (eg, surgeons, psychotherapists)	**X**
Interventions	11a	Interventions for each group with sufficient detail to allow replication, including how and when they will be administered	**NA**
	11b	Criteria for discontinuing or modifying allocated interventions for a given trial participant (eg, drug dose change in response to harms, participant request, or improving/worsening disease)	**NA**
	11c	Strategies to improve adherence to intervention protocols, and any procedures for monitoring adherence (eg, drug tablet return, laboratory tests)	**NA**
	11d	Relevant concomitant care and interventions that are permitted or prohibited during the trial	**NA**
Outcomes	12	Primary, secondary, and other outcomes, including the specific measurement variable (eg, systolic blood pressure), analysis metric (eg, change from baseline, final value, time to event), method of aggregation (eg, median, proportion), and time point for each outcome. Explanation of the clinical relevance of chosen efficacy and harm outcomes is strongly recommended	**X**
Participant timeline	13	Time schedule of enrolment, interventions (including any run-ins and washouts), assessments, and visits for participants. A schematic diagram is highly recommended (see Figure)	**X**
Sample size	14	Estimated number of participants needed to achieve study objectives and how it was determined, including clinical and statistical assumptions supporting any sample size calculations	**X**
Recruitment	15	Strategies for achieving adequate participant enrolment to reach target sample size	**X**
**Methods: Assignment of interventions (for controlled trials)**			**NA**
Allocation:			
Sequence generation	16a	Method of generating the allocation sequence (eg, computer-generated random numbers), and list of any factors for stratification. To reduce predictability of a random sequence, details of any planned restriction (eg, blocking) should be provided in a separate document that is unavailable to those who enrol participants or assign interventions	**NA**

Allocation concealment mechanism	16b	Mechanism of implementing the allocation sequence (eg, central telephone; sequentially numbered, opaque, sealed envelopes), describing any steps to conceal the sequence until interventions are assigned	**NA**
Implementation	16c	Who will generate the allocation sequence, who will enrol participants, and who will assign participants to interventions	**NA**
Blinding (masking)	17a	Who will be blinded after assignment to interventions (eg, trial participants, care providers, outcome assessors, data analysts), and how	**NA**
	17b	If blinded, circumstances under which unblinding is permissible, and procedure for revealing a participant's allocated intervention during the trial	**NA**
**Methods: Data collection, management, and analysis**			
Data collection methods	18a	Plans for assessment and collection of outcome, baseline, and other trial data, including any related processes to promote data quality (eg, duplicate measurements, training of assessors) and a description of study instruments (eg, questionnaires, laboratory tests) along with their reliability and validity, if known. Reference to where data collection forms can be found, if not in the protocol	**X**
	18b	Plans to promote participant retention and complete follow-up, including list of any outcome data to be collected for participants who discontinue or deviate from intervention protocols	**NA**
Data management	19	Plans for data entry, coding, security, and storage, including any related processes to promote data quality (eg, double data entry; range checks for data values). Reference to where details of data management procedures can be found, if not in the protocol	**X**
Statistical methods	20a	Statistical methods for analysing primary and secondary outcomes. Reference to where other details of the statistical analysis plan can be found, if not in the protocol	**X**
	20b	Methods for any additional analyses (eg, subgroup and adjusted analyses)	**X**
	20c	Definition of analysis population relating to protocol non-adherence (eg, as randomised analysis), and any statistical methods to handle missing data (eg, multiple imputation)	**X**
**Methods: Monitoring**			**NA**
Data monitoring	21a	Composition of data monitoring committee (DMC); summary of its role and reporting structure; statement of whether it is independent from the sponsor and competing interests; and reference to where further details about its charter can be found, if not in the protocol. Alternatively, an explanation of why a DMC is not needed	**NA**

For peer review only - http://bmjopen.bmj.com/site/about/guidelines.xhtml

	21b	Description of any interim analyses and stopping guidelines, including who will have access to these interim results and make the final decision to terminate the trial	**NA**
Harms	22	Plans for collecting, assessing, reporting, and managing solicited and spontaneously reported adverse events and other unintended effects of trial interventions or trial conduct	**NA**
Auditing	23	Frequency and procedures for auditing trial conduct, if any, and whether the process will be independent from investigators and the sponsor	**NA**
**Ethics and dissemination**			
Research ethics approval	24	Plans for seeking research ethics committee/institutional review board (REC/IRB) approval	**X**
Protocol amendments	25	Plans for communicating important protocol modifications (eg, changes to eligibility criteria, outcomes, analyses) to relevant parties (eg, investigators, REC/IRBs, trial participants, trial registries, journals, regulators)	**X**
Consent or assent	26a	Who will obtain informed consent or assent from potential trial participants or authorised surrogates, and how (see Item 32)	**X**
	26b	Additional consent provisions for collection and use of participant data and biological specimens in ancillary studies, if applicable	**NA**
Confidentiality	27	How personal information about potential and enrolled participants will be collected, shared, and maintained in order to protect confidentiality before, during, and after the trial	**X**
Declaration of interests	28	Financial and other competing interests for principal investigators for the overall trial and each study site	**X**
Access to data	29	Statement of who will have access to the final trial dataset, and disclosure of contractual agreements that limit such access for investigators	**X**
Ancillary and post-trial care	30	Provisions, if any, for ancillary and post-trial care, and for compensation to those who suffer harm from trial participation	**NA**
Dissemination policy	31a	Plans for investigators and sponsor to communicate trial results to participants, healthcare professionals, the public, and other relevant groups (eg, via publication, reporting in results databases, or other data sharing arrangements), including any publication restrictions	**X**
	31b	Authorship eligibility guidelines and any intended use of professional writers	**X**
	31c	Plans, if any, for granting public access to the full protocol, participant-level dataset, and statistical code	**NA**

For peer review only - http://bmjopen.bmj.com/site/about/guidelines.xhtml

Appendices

Informed consent materials	32	Model consent form and other related documentation given to participants and authorised surrogates	**-**
Biological specimens	33	Plans for collection, laboratory evaluation, and storage of biological specimens for genetic or molecular analysis in the current trial and for future use in ancillary studies, if applicable	**NA**

*It is strongly recommended that this checklist be read in conjunction with the SPIRIT 2013 Explanation & Elaboration for important clarification on the items. Amendments to the protocol should be tracked and dated. The SPIRIT checklist is copyrighted by the SPIRIT Group under the Creative Commons "Attribution-NonCommercial-NoDerivs 3.0 Unported" license.

For peer review only

BMJ Open

Central Sensitization and Functioning in patients with Chronic Low Back Pain: Protocol for a cross-sectional and cohort study

Journal:	*BMJ Open*
Manuscript ID	bmjopen-2019-031592.R2
Article Type:	Protocol
Date Submitted by the Author:	14-Oct-2019
Complete List of Authors:	Ansuategui Echeita, Jone; University Medical Center Groningen, Rehabilitation Medicine<br>Schiphorst Preuper, Henrica ; University Medical Center Groningen, Rehabilitation Medicine<br>Dekker, Rienk; University Medical Center Groningen, Rehabilitation Medicine; University Medical Center Groningen, Center for Sports Medicine<br>Stuive, Ilse; University Medical Center Groningen , Rehabilitation Medicine<br>Timmerman, Hans; Radboud University Medical Center, Department of Anesthesiology, Pain and Palliative Medicine<br>Wolff, Andre; University Medical Center Groningen, Anesthesiology Pain Center<br>Reneman, Michiel; University Medical Center Groningen, Rehabilitation Medicine
**Primary Subject Heading**:	Rehabilitation medicine
Secondary Subject Heading:	Anaesthesia, Sports and exercise medicine, Occupational and environmental medicine
Keywords:	Hyperalgesia, Autonomic Nervous System, Lifting, Exercise Test, Physical Activity, Disability

SCHOLARONE™ Manuscripts

I, the Submitting Author has the right to grant and does grant on behalf of all authors of the Work (as defined in the below author licence), an exclusive licence and/or a non-exclusive licence for contributions from authors who are: i) UK Crown employees; ii) where BMJ has agreed a CC-BY licence shall apply, and/or iii) in accordance with the terms applicable for US Federal Government officers or employees acting as part of their official duties; on a worldwide, perpetual, irrevocable, royalty-free basis to BMJ Publishing Group Ltd ("BMJ") its licensees and where the relevant Journal is co-owned by BMJ to the co-owners of the Journal, to publish the Work in this journal and any other BMJ products and to exploit all rights, as set out in our licence.

The Submitting Author accepts and understands that any supply made under these terms is made by BMJ to the Submitting Author unless you are acting as an employee on behalf of your employer or a postgraduate student of an affiliated institution which is paying any applicable article publishing charge ("APC") for Open Access articles. Where the Submitting Author wishes to make the Work available on an Open Access basis (and intends to pay the relevant APC), the terms of reuse of such Open Access shall be governed by a Creative Commons licence – details of these licences and which Creative Commons licence will apply to this Work are set out in our licence referred to above.

Other than as permitted in any relevant BMJ Author's Self Archiving Policies, I confirm this Work has not been accepted for publication elsewhere, is not being considered for publication elsewhere and does not duplicate material already published. I confirm all authors consent to publication of this Work and authorise the granting of this licence.

For peer review only

Central Sensitization and Functioning in patients with Chronic Low Back Pain: Protocol for a cross-sectional and cohort study

J. Ansuategui Echeita [1], H.R. Schiphorst Preuper [1], R. Dekker [1], I. Stuive [1], H. Timmerman [2, 3], A.P. Wolff [3], M.F. Reneman [1].

Affiliations:

[1] Department of Rehabilitation Medicine, University of Groningen, University Medical Center Groningen, Groningen, The Netherlands.

[2] Department of Anesthesiology, Pain and Palliative Medicine, Radboud university medical center, Nijmegen, The Netherlands.

[3] Department of Anesthesiology Pain Center, University of Groningen, University Medical Center Groningen, Groningen, The Netherlands.

Corresponding author: Jone Ansuategui Echeita, Department of Rehabilitation Medicine, University Medical Center Groningen, P.O. Box 30.002, 9750 RA Haren, The Netherlands. Email: j.ansuategui.echeita@umcg.nl

For peer review only

ABSTRACT

Introduction. A relevant subsample of patients with chronic low back pain (CLBP) have manifested augmented pain processing, Central Sensitization (CS). Patients with CLBP have limited functioning and participation. Theoretically, physical functioning in patients with CLBP can plausibly be linked to CS; however, evidence to explain such association is scarce. Moreover, there is no gold standard to diagnose CS. The objectives of the study are:

1) To analyze the association between instruments assessing symptoms and signs attributed to CS.
2) To analyze whether symptoms and signs attributed to CS are associated with functioning measurement outcomes.
3) To analyze whether changes (between baseline and discharge) in symptoms and signs attributed to CS are related to changes in each of the functioning measurement outcomes.

Methods and analysis. Cross-sectional and longitudinal observational study with measurements at baseline and discharge of an interdisciplinary rehabilitation program. 115 adult patients with CLBP are calculated to participate. CS measurements: Central Sensitization Inventory, Quantitative Sensory Testing, and Heart-Rate Variability. Functioning measurements: Lifting capacity, Maximal aerobic capacity, Accelerometry, and Reported functioning. Statistical analyses: 1) correlation between CS measurements, 2) multiple regression between functioning (dependent variable) and CS measurements (independent variable), 3) multiple regression between change in scores of functioning (dependent variable) and CS measurements (independent variable); and corrected for sex and age.

Ethics and dissemination. The study obtained the clearance to its implementation from the Medical Research Ethics Committee of the University Medical Center Groningen (METc UMCG) in July 2017. The results will be disseminated through scientific publications in peer-reviewed journals, presentations at relevant conferences, and reports to stakeholders.

Trial registration number. NTR7167.

KEYWORDS

Hyperalgesia, Autonomic Nervous System, Somatosensory, Lifting, Exercise Test, Physical Activity, Disability.

For peer review only

STRENGTHS AND LIMITATIONS OF THIS STUDY

- Novel and comprehensive approaches for the assessment of the presence of CS.
- Extensive amount of measurements from multiple domains for the determination of functioning status.
- Longitudinal study with measurements at baseline and discharge of an interdisciplinary rehabilitation program implemented as 'Care as Usual'.
- Strategies allocated to promote participation and adherence, and to mitigate to the largest extent possible selection bias.
- Statistical analyses are performed with selections of CS and functioning measurements, and these selections may influence study results.

For peer review only

INTRODUCTION

Background

Twenty percent of the general population experiences prolonged or recurrent pain for at least three months, such pain is then considered to be chronic.[1,2] Low back pain is the principal cause of Years Lived with Disability (YLD) and work days lost globally and in Europe,[3] and its sequelae affect 9% of the global population.[4] Moreover, although there are several treatment intervention strategies available for patients with Chronic Low Back Pain (CLBP), treatment effect sizes have only been moderate.[5] Such evidence may explain that 40% of the patients are not satisfied with the treatment received.[2] The economic consequences of chronic pain include direct costs of medical care and pain management treatments, as well as indirect costs of decreased work productivity and sick-leave, and of disability and sickness-retirement costs.[2,6] The indirect costs determine 86-88% of the total costs produced by patients with CLBP.[7,8] In particular, in the Netherlands, the costs of back pain to Dutch society were 3.5 billion in 2007, corresponding to 0.6% of the Gross National Product.[8]

In the past decennia, a relevant subsample of patients with chronic pain, including individuals with CLBP, have shown an increased responsiveness to noxious and non-noxious stimuli, described as Central Sensitization (CS).[9] CS is a result of an imbalance in the nociceptive pathways ("pain pathways") and supraspinal structures due to an amplified facilitation and/or reduced inhibition.[10–12] The phenomenon, CS, is manifested by an amplified pain perception regarding its intensity (hyperalgesia and allodynia), duration (aftersensations and temporal summation), and distribution (expansion of the receptive field); and a reduced conditioned pain modulation.[12] There is currently no gold standard to diagnose CS. The identification of reference clinical symptoms and signs, however, may indicate the presence of CS: disproportionate pain, diffuse distribution, allodynia and hyperalgesia, hypersensitivity unrelated to mechanical stimulus but to environmental sensations (light, temperature, noise, or other stressors), maladaptive psychosocial factors, and low vagal nerve activity.[13–16] Instruments like Central Sensitization Inventory (CSI),[17] Quantitative Sensory Testing (QST) [18,19] and Heart-Rate Variability (HRV);[20] are non-invasive, feasible, and assess different reference clinical symptoms and signs indicative of CS.[12] In absence of a gold standard, these instruments comprehensively measure different aspects of CS.

Chronic pain is a multifactorial condition which has impact on physical or body structures and functions, on psychological processes, and on daily activities and quality of life of individuals.[2,21,22] Limited functioning or capacity to perform everyday tasks, essential for an independent living, affects the mobility and self-care, social relationships, and work and leisure performance of individuals with chronic pain.[23] Individual's functioning is composed of body functions (body level), described by impairments individuals experience; and of activities and participation (personal and social levels, respectively) described by capacity and performance tests.[22,24] Lifting

For peer review only - http://bmjopen.bmj.com/site/about/guidelines.xhtml

capacity, Physical Activity (PA) levels and/or PA distribution or patterns, aerobic capacity, or daily activities participation, are measures that account for the multiple operationalizations of functioning and together provide a more comprehensive overview of different domains of the functioning status.

Patients with CLBP are likely to be less physically active, deconditioned, and disabled. Nowadays much is still unknown about the association of functioning with CLBP, either because research is immature or because the results are conflicting mostly due to differences in methodology.[25-27] Furthermore, the association with CS is scarce due to the limited and diverse use of valid CS measures.[28-30] The assessment and management of pain of centrally sensitized patients with CLBP may be improved with: 1) an ample selection of capacity and performance tests and experienced impairments measures to determine individual's functioning status; and 2) an extensive assessment of the presence of CS reference clinical symptoms and signs with different instruments. Theoretically, CS can plausibly be linked to physical functioning in individuals with CLBP, because: 1) CS has been identified in a subsample of patients with CLBP. And 2) individuals with CLBP experience limitations in (physical) functioning and participation. Because patients with CS are more reactive to stimuli, the pain experience is enhanced, and, consequently, the presence of CS and/or higher levels of CS could be associated with lower physical functioning and participation in patients with CLBP, and vice versa. However, there is no straightforward evidence of the association between CS and functioning at the moment because both constructs have consistently been reported with different methodologies and outcome parameters.

The present study described in this research protocol is created to fill these existing gaps in knowledge. It intends to assist on the phenotyping of centrally sensitized patients with CLBP by means of the description of mechanisms involved in CS (CS reference clinical symptoms), and to improve the understanding of the CS phenomenon and of the association and influence of CS on multiple physical functioning elements. Including the influence of various physiological and psychological processes related to chronic pain disorders.

Objective(s)

The objectives of the studies covered by this research protocol are:

1. To analyze the association between three instruments assessing the symptoms and signs attributed to CS in patients with CLBP: CSI, QST, and HRV.
2. To analyze whether symptoms and signs attributed to CS are associated with functioning measurement outcomes in patients with CLBP. Specifically, to analyze the association between the three instruments assessing CS (CSI, QST, and HRV) and: Lifting capacity, Aerobic capacity, PA levels and/or PA distribution or patterns, and patient-reported measurements.

For peer review only - http://bmjopen.bmj.com/site/about/guidelines.xhtml

3. To analyze whether changes (between baseline and discharge) in symptoms and signs attributed to CS are related to changes in each of the functioning measurement outcomes in patients with CLBP. Specifically, to analyze whether decreases in CS described as: a lower CSI score, an increase in the pain thresholds (QST), and an increase in parasympathetic/vagal tone (HRV); are related to a better functioning described as: an increase in lifting capacity, an increase in aerobic capacity, an increase in PA levels and/or a change in PA distribution or patterns, and better outcome scores in patient-reported measurements.

METHODS AND ANALYSES

Study design

An observational study project with both cross-sectional and longitudinal designs is being conducted from September 2017 to September 2019 in the Center for Rehabilitation of the University Medical Center Groningen (CvR – UMCG) in The Netherlands.

Patient and Public Involvement

Personnel of the CvR-UMCG collaborate in the study, in particular, the Pain rehabilitation team during patient management and data collection process and the Central exercise lab team on data collection process. The authors wish to express their gratitude to the collaborators and to the patients that participate.

Participants

Consecutive patients between 18 and 65 years of age who are primarily referred to the outpatient Pain Rehabilitation Department of the CvR - UMCG because of chronic primary low back pain, even if patients have gone under surgery in the past,[31] and who are admitted for the interdisciplinary pain rehabilitation program are eligible to participate in the study. Additionally, patients are invited to the study if they are mentally competent and can follow instructions. Furthermore, patients are excluded from the study if, based on their file, they: have a specific diagnosis that would better account for the symptoms (e.g. cancer, osteoporosis, and/or spinal fractures), have neuralgia and/or radicular pain in the legs, have some major disorder or co-morbidity that can affect their physical and/or mental functioning (e.g. psychiatric disorders) or that can interfere with the study, are pregnant (or planning to be), have any specific contraindication according to the standards of practice guidelines of the assessments that they are going to be subject to,[32,33] or take any medication that may interfere with the assessment outcomes such as calcium channel blockers on

For peer review only - http://bmjopen.bmj.com/site/about/guidelines.xhtml

HRV. CS-related comorbidities (i.e. fibromyalgia, osteoarthritis, or chronic fatigue syndrome) are no reason for exclusion from the study.

Procedures

Measurements are performed as part of 'Care As Usual' (CAU) for outpatients following the Pain Rehabilitation guidelines of the CvR - UMCG. However, not all measurements of the research protocol belong to the battery of assessments of the CvR – UMCG, as pointed out in the legend of Figure 1.

During the first appointment of the patients with the rehabilitation physician, patients are invited to participate in the studies if they are eligible according to the criteria defined for the studies. The eligible patients who are invited to participate and receive information about the study, but have not reacted, are contacted in a week's time to remind them about the study participation. Patients who signed the informed consent fill in forms with their relevant personal information during the baseline assessment, and they are given vouchers for the travel expenses during the discharge assessment. The participating patients follow CAU trajectory with the Pain Rehabilitation team in terms of assessments, screening, and implementation of the interdisciplinary rehabilitation program. As part of the research protocol, the performance of additional assessments and filling of questionnaires on CS and functioning are required. All CS, and objective monitoring and capacity tests are measured at two time points, baseline and discharge: the questionnaire CSI as CAU; the physical assessments lifting capacity and HRV as CAU, and aerobic capacity and QST as additional; and the monitoring of PA with an accelerometer as additional during the first and last week of the rehabilitation program. The pain intensity and functioning questionnaires are measured at 13 time points, baseline and once a week during the 12-week rehabilitation program: PDI as CAU at baseline but as additional during the rehabilitation program, and VAS Pain, WAS and Rand36-PF as additional. A diagram of the project procedure is presented as Figure 1.

(Insert Figure 1 around here)

Measurements

Central Sensitization measurements

To comprehensively assess the symptoms and signs attributed to CS, data is obtained from a questionnaire (CSI) and two physical assessments (HRV and QST).

CSI. The inventory is a self-reported questionnaire with two sections to estimate the severity of CS-related symptoms. Section A is a 25-item questionnaire to assess the presence of the most common CS-related symptoms. Each of the

For peer review only - http://bmjopen.bmj.com/site/about/guidelines.xhtml

questions is measured on a 5-point Likert scale from 0 (never) to 4 (always), and the total score ranges from 0 to 100, where higher scoring is associated with more severe symptomatology. Section B is a 10-item questionnaire to assess the previously diagnosed CS Syndromes (CSS) and/or conditions related to CS. Higher amount of CSS is associated with higher chances of CS being present. This instrument has shown excellent test-retest reliability (ICC = 0.82-0.97) and internal consistency (Cronbach α = 0.87-0.91) in the general population and in patients with chronic pain,[17,34-36] as well as good concurrent validity (sensitivity = 81%, specificity = 75%) for CSS diagnosis within patients with chronic pain.[34] For the purposes of the study the Dutch translation of the CSI is used,[37] which has also shown excellent internal consistency (Cronbach α = 0.91) and test-retest reliability (ICC = 0.91 and 0.88) in controls and in patients with chronic musculoskeletal pain respectively.[38] The psychometric information of CSI in patients with CLBP is not known.

QST. A battery of standardized sensory tests that quantifies the function of the somatosensory system. QST tests the characteristics of various sensation and nociceptive processes as representative of afferent nerve fibers and central pathways performance. The whole assessment lasts for 1h, and it consists of six sensory tests, which are measured in seven different predefined body locations: one training location to familiarize the patient with the tests, the most painful location (as pointed out by the patient), and five control locations. See Supplementary material 1 for full description of locations. The standardized instructions of the sensory tests are read to the patient at each test performance. Of the tests, five consist of mechanical stimuli based on and executed following the QST examination of the German Research Network on Neuropathic Pain (DFNS) protocol,[18] and one for conditioned pain modulation following the Nijmegen-Aalborg Screening QST (NASQ) protocol.[19,39] Overall, the DFNS protocol has shown good test-retest (mean r = 0.86) and inter-observer reliability (mean r = 0.83) in patients with affections in the somatosensory system either with or without pain.[40] For the purpose of avoiding bias due to the order of the measurements and locations, a counterbalancing procedure is established which accounts for the locations and the measurements to be made. This procedure provides three different assessment order groups, all of which begin with the measurements at the training location and end with the conditioned pain modulation test. An additional counterbalancing procedure has been made for allocating patients to these assessment order groups (either 1, 2, or 3); every nine patients a different allocation order is performed to ensure a similar amount of QST assessments per group. All assessors are trained in the QST protocol. The following sensory tests are performed:

- *The Dynamic Mechanical Allodynia (DMA)* to test the presence of abnormal stimulus-evoked pain is assessed with a soft brush (SENSElab^{TM}, Brush-05, Somedic AB, Hörby, Sweden) that is swiped 1-2 cm over the skin. This is performed on a single repetition and on a train of three repetitions (wind-up response). Whether patients feel the sweeping and its pain intensity with a Numeric Rating Scale (NRS) ranging

For peer review only - http://bmjopen.bmj.com/site/about/guidelines.xhtml

from 0 (no pain) to 100 (most intense pain imaginable) for both the single swipe and the series are recorded in each of the seven locations (see Supplementary material 1).[41,42] The DMA (ratio) is calculated as: DMA = (NRS-3 / NRS-1). The DMA test according to the DFNS protocol has shown to have good test-retest (r = 0.87) and inter-observer reliability (r = 0.79).[40]

- *The Mechanical Pain Threshold (MPT)* to test the ability to perceive sharp, pricking, or stinging stimuli is assessed with a set of standardized weighted pinprick stimulators with seven different intensities (The PinPrick stimulator set, MRC Systems GmbH, Germany) and with a modified method of limits. Starting at the lowest intensity (8mN) this is progressively increased until patients report the stimulus to change from touch to painful (sharp, pricking, or stinging). The intensity of the pinprick which produces the first painful stimulus in mN is recorded in each of the seven locations (see Supplementary material 1).[42-45] The MPT test according to the DFNS protocol has shown to have good test-retest (r = 0.80) and inter-observer reliability (r = 0.80).[40]

- *The Wind-Up Ratio (WUR)* to test the Nervous System's painful stimuli temporal summation or responsiveness to repeated noxious stimuli. Such test is assessed with a suprathreshold pinprick stimulator of 256mN (The PinPrick stimulator set, MRC Systems GmbH, Germany), which is applied on a single repetition and on a train of ten repetitions in an area of 1cm^2 with intervals of one second (wind-up response). The pain intensity measured with a Numeric Rating Scale (NRS) ranging from 0 (no pain) to 100 (most intense pain imaginable) for both the single repetition and the series are recorded in each of the seven locations (see Supplementary material 1).[46,47] The WUR (ratio) is calculated as: WUR = (NRS-10 / NRS-1). The WUR test according to the DFNS protocol has shown to have poor test-retest (r = 0.67) and inter-observer reliability (r = 0.56).[40]

- *Mechanical Detection Threshold (MDT)* to test the ability to detect light touch, is assessed with a standardized set of von Frey filaments (OptiHair2-Set, Marstock Nervtest, Germany) with a modified method of limits.[48,49] Starting at the intensity of 16mN, the stimulus application is progressively decreased until patients do not report any sensation. The intensity of the filament that is not perceived in mN is recorded in each of the seven locations (see Supplementary material 1).[50] The MDT test according to the DFNS protocol has shown to have good test-retest (r = 0.90) and inter-observer reliability (r = 0.89).[40]

- *Pressure Pain Threshold (PPT)* to test the ability to perceive pressure pain stimuli on muscles, is assessed with an electronic pressure algometer (Force Ten ™ FDX, Wagner Instruments, USA), with which pressure in the contact point (1cm^2 tip), is progressively but steadily increased until patients report to feel pressure as painful (burning, drilling, or aching). The intensity in N when pressure becomes painful and its pain intensity with a Numeric Rating Scale (NRS) ranging from 0 (no pain) to 100 (most intense pain imaginable) scale is recorded

For peer review only - http://bmjopen.bmj.com/site/about/guidelines.xhtml

in each of the seven locations (see Supplementary material 1).[18] The PPT test according to the DFNS protocol has shown to have good test-retest (r = 0.88) and inter-observer reliability (r = 0.84).[40]

- *Conditioned Pain Modulation (CPM)* to test the Nervous System's descending pain processing. The CPM test methodology selected for this study follows the recommendations of the EFIC meeting (2013).[51] The pain modulation is assessed by means of PPT tests (Test Stimuli, TS) in a distant location from patients dominant hand (m. Deltoid and m. Quadriceps of the non-dominant side, see Supplementary material 1) before and after immersing the dominant hand in a bucket full with ice and cold water (Cold Conditioning Stimuli, cCS). First, PPT measurements at distant locations are made; then, patients immerse the dominant hand in the bucket for as long as they possibly can with a maximal duration of 3 minutes; finally, PPT measurements are repeated. The PPT results, intensity in N and pain intensity with a Numeric Rating Scale (NRS) ranging from 0 (no pain) to 100 (most intense pain imaginable) scale, in both locations and the time the hand is immersed are recorded. The CPM (%) is calculated as: $CPM = [(PPTpost - PPTpre) / PPTpre] * 100$; where lower values indicate lower alteration on the nociceptive pathways.[19,39] The CPM test, as the combination of the PPT handheld algometer and cold stimuli, has shown to be one of the most reliable methods with a modest test-retest reliability (ICC = 0.49 and Coefficient of Variation, CV = 63.6%).[52]

HRV. The test provides an indication of the function and balance of the Autonomic Nervous System (ANS), which is responsible for the control of internal functions including self-regulation, adaptability, and resilience capacities.[53] The ANS is susceptible to mental and emotional processes, which in turn can produce an imbalance in self-regulation (affecting among others HR) by an hyper-activation of the sympathetic tone and reduced parasympathetic/vagal tone.[15,17] HRV testing can monitor heart rhythms, or time and pattern variation on HR and inter-beat (RR) intervals, and can be used to assess the function of the ANS.[54,55] The HRV test is performed by assessors trained by the HeartMath institute, and the 5-minute protocol is executed to guarantee the comparison of the results as recommended by the Task Force for short-term recordings.[54] Patients are instructed to sit calmly and breathe normally, while they have an ear pulse sensor placed which is connected to a computer.[56] The following measures are collected and recorded with the emWave PC Software (emWave®, HeartMath Inc., Boulder Creek, California): mean HR in beats per minute (bpm), mean RR interval in ms; time domain measures in ms as Standard Deviation of Normal-to-Normal interval (SDNN) indicator for cyclic components responsible of RR interval variability, Root Mean Square of Successive Differences (RMSSD) for beat-to-beat variance in HR and parasympathetic/vagal tone; frequency domain measures in ms^2/Hz as Very Low Frequency (VLF, 0.0033-0.04 Hz) indicator for long term regulation and sympathetic tone, Low Frequency (LF, 0.04-0.15 Hz) for sympathetic and parasympathetic/vagal tones, and High Frequency (HF,

For peer review only

0.15-0.40 Hz) for parasympathetic/vagal tone related to breathing rate; and Normalized Coherence indicator for HRV stable or regular pattern over time in %, where higher scores mean more harmonic signal.

Physical Functioning measurements

To determine the functioning status of individuals with chronic pain in multiple domains, data is obtained from two physical assessments (lifting and aerobic capacity tests), an accelerometer (PA level), and a battery of reported functioning questionnaires.

Lifting capacity test. The Floor-to-waist lift capacity test is based on the WorkWell Functional Capacity Evaluation (FCE) protocol [57] and is performed to measure patients work functioning or capacity. Such FCE lift test has been shown to have high test–retest (ICC = 0.81) and inter-rater reliability (ICC = 0.76) in patients with CLBP.[58,59] Assessors are trained in the floor-to-waist lift capacity test procedure. Patients are given standardized instruction to repetitively lift a crate with weights from a shelf at waist height to the floor and back. The test begins with a weight that can easily be lifted, progressively the load is increased, and the test ends when the maximum capacity is reached. The final endpoint can be reached due to: cardiac endpoint (85% of maximum heart rate); biomechanical endpoint (unsafe increasing weight because of lack of load handling control); patient endpoint (patient decided to stop); and criterion endpoint (normal end of the test).[60] The maximal load lifted in kg is recorded along with the endpoint reason, and the patient-reported and the assessor-observed mechanical effort as measured with Borg's Category Ratio scale (CR-10).[61] Borg's CR-10 scale ranges from 0 (at rest) to 10 (very, very hard), and has demonstrated to be a valid visual observation of the effort level of patients during their performance of a lifting capacity test.[62]

Aerobic capacity test. The maximal aerobic capacity is assessed with a cardio-pulmonary exercise test (CPET). The test is performed with a cycle ergometer (Ergoselect 200p or Ergoselect 200k, Ergoline, Bitz, Germany) following a defined continuous ramp protocol. Before any cycling begins, patients are interviewed about their daily activities, and their resting heart rhythm and function are recorded with an electrocardiogram (ECG) (assy cam-14, GE medical systems, US). Patients then have an unloaded warm-up of three minutes at 60-70 rotations per minute (rpm) before the test begins. At the start of the test, an experienced exercise physiologist in discussion with a specialized physician or nurse determine the starting workload (25-100 W) and ramp (5-25 W increase per minute) depending on patients estimated fitness level. During the test patients are asked to maintain a constant cadence while the working load is progressively being increased. Also, they are continuously being verbally encouraged to obtain their maximum performance. The maximal performance is determined by various parameters: a temporary loss of strength and energy (= exhaustion), a plateau in the peak oxygen uptake (VO2max), a respiratory exchange ratio (RER) higher than 1.15, and/or a heart-rate (HR) higher than 85% of the predicted maximal HR.[33] Overall, the test lasts for 8-12 minutes and throughout the whole assessment patients are monitored on their cardiac activity with an ECG, on their blood pressure with a cuff

For peer review only - http://bmjopen.bmj.com/site/about/guidelines.xhtml

algometer, and on their ventilatory gases on a breath-by-breath basis with a metabolic cart (JAEGER ® Vyntus ® CPX, Jaeger, Germany, Hoechberg). The VO2max in ml/min and per kilogram in ml/min/kg is collected as the mean of VO2 over the last 30 seconds of exercise. Along with VO2max are collected: peak workload in watts, HR at end of test in beats per minute (bpm), breathing frequency (BF) at end of test in breaths per min, expiratory tidal volume (VT) in l, peak energy expenditure in Metabolic Equivalent of Task (MET), test duration in min, and the patient-reported and the assessor-observed mechanical effort as measured with Borg's rating of perceived exertion (RPE) scale. Borg's RPE scale ranges from 6 (no exertion at all) to 20 (maximal exertion) [61] and has shown to be highly correlated with HR (r = 0.74) and blood lactate (r = 0.83),[63] demonstrating to be a reliable instrument to measure work intensity.

PA levels. Objective measurements of PA levels are obtained by means of an accelerometer (ActiGraph GT9X Link, ActiGraph, Pensacola, FL, USA). The accelerometer is a small and light device that monitors wearers' daily PA and energy expenditure. Participating patients wear the accelerometer attached at their hip level for one week continuously during walking hours, in order to ensure the minimum of 4-full-day recording as recommended by activity monitoring reviewers and experts.[64,65] Different algorithms are chosen from the software provided by the manufacturer, ActiLife6 (ActiGraph, Pensacola, FL, USA), to discriminate between low PAs and split patients in three activity intensity categories (sedentary, light, and moderate) [66] and to obtain patients' energy expenditure in kcal [67] and MET.[68] The daily and weekly time in hours and percentage of time spent in each of the activity intensity categories as well as the estimated energy expenditure in both kcal and MET are collected.

Reported Functioning. Data from this domain is obtained from four questionnaires sent to patients and filled by them via internet. All the questionnaires require 10-15 minutes to be filled in.

- *Physical functioning (Rand36 Physical Functioning subscale – Rand36-PF)*. The Rand36 is a survey that measures the impact of health on patients' quality of life, patients are asked to only answer the physical functioning (PF) subscale which looks at the limitations patients experience during their daily activities. Each of the 10 items belonging to the PF subscale have 3-level response choices ranging from 0 (limited a lot) to 100 (not limited at all), the total average score ranges from 0 to 100 too; where a higher score is associated with a greater limitation in performing daily activities. For the purposes of the study the Dutch translation of the Rand36 is used, which has shown good internal consistency (Cronbach α = 0.92) and test-retest reliability (r = 0.72-0.82) in the general population.[69,70]

- *Disability (Pain Disability Index – PDI)*. The index is a 7-item questionnaire which measures the interference of pain with functioning in different daily life activities: family/home responsibilities, recreation, social activity, occupation, sexual behavior, self-care, and life-support activity. Each item ranges from 0 (no interference) to 10

For peer review only

(total interference), and the total score ranges from 0 to 70; where scoring higher is associated with higher interference in daily life activities. The PDI has shown modest test-retest reliability (r = 0.44) and high internal consistency (Cronbach α = 0.86) in patients with chronic pain.[71] For the purposes of the study the Dutch translation of the PDI was used, which has shown a good internal consistency (Cronbach α = 0.85) and test-retest reliability (ICC = 0.76) in patients with musculoskeletal pain.[72]

- *Work ability (Work Ability Score – WAS)*. The score is a single-item question which compares patient's current work ability compared with their lifetime best. The result is a score ranging from 0 (unable to work) to 10 (best working ability). The single-item question is part of the Work Ability Index (WAI), a questionnaire which measures work ability and has shown to be test-retest reliable (difference between test and re-test = -0.53) in construction workers.[73] The WAS has shown to be highly correlated to the 28 items of the WAI among women on a long-term sick leave (r = 0.87) [74] and among active workers (r = 0.63).[75]

Supplementary measurements

Demographic characteristics. Patients filled in a form with their details when the QST assessment took place. Such included: age, sex, nationality, height, weight, pain characteristics (affected body area, duration and medication, if applicable), educational level, and employment characteristics (work-status and physical work demands per Dictionary of Occupational Titles (DOT) [76]).

Pain-related questionnaires.

- *Pain intensity (Visual Analogue Scale – VAS Pain)*. The scale is a single-item question to assess the current pain intensity in adults. It is a straight line with 'no pain' (score = 0) at one end and 'pain as bad as it could be' (score = 100) at the other, in which patients are asked to mark the line at the level that represents their pain level. The score is the distance between the mark and the 'no pain' end, where scoring higher is associated with greater pain intensity. The VAS Pain has shown good validity (r = 0.62-0.91) and good test-retest reliability (literate r = 0.94 and illiterate r = 0.71) in patients with rheumatic pain conditions.[77] VAS Pain is sent to patients and filled in by them via internet along with the battery of functioning questionnaires (described above); i.e. at baseline and weekly during the interdisciplinary rehabilitation program.

- *Body response to a straining exercise (Pain Response Questionnaire – PRQ).*[78] The questionnaire assesses the pain intensity of the different body locations on an 11-point numeric scale (NRS) ranging from 0 (no pain) to 10 (worst pain imaginable), and the type of pain felt (muscle soreness, other, both, or unknown). Additionally it controls for the use of medication and for any other strenuous physical activity. For the purposes of this study, the body locations measured are predefined to four and the questionnaire is being filled in three occasions:

For peer review only - http://bmjopen.bmj.com/site/about/guidelines.xhtml

before the CPET, immediately afterwards, and 24h later. PRQ is filled at the same moment when the aerobic capacity test is performed; i.e. during baseline and discharge assessments.

Psychological screening questionnaires. These questionnaires measure psychosocial features common to CS syndromes that have demonstrated to affect pain experience and functioning.[79] Patients filled in the questionnaires via internet which were sent per CAU procedures by the Pain Rehabilitation team. Such were:

- *Pain Catastrophizing Scale (PCS)*. The 13-item reported questionnaire measures measured in a 5-point Likert scale (0 - not at all, 4 - all the time) the degree to which patients have had catastrophic thoughts or feelings during their pain experience and accounts for three subscales or dimensions: rumination, magnification, and helplessness.[80] For the purposes of the study the Dutch translation of the PCS was used,[81] which has shown good internal consistency (Cronbach α = 0.85-0.91) and test-retest reliability (r = 0.92) among healthy individuals (students) and patients with chronic low back pain.[82]
- *Injustice Experience Questionnaire (IEQ)*. The 12-item reported questionnaire assesses in a 5-point Likert scale from (0 – never, 4 - all the time) four different aspects of the perceived injustice due to injury: severity, blame, unfairness, and irreparability. The questionnaire has shown to have good internal consistency (Cronbach α = 0.92) and test-retest reliability (ICC = 0.98) in a group of patients with fibromyalgia.[83] For the purposes of the study the Dutch translation of the IEQ was used, as translated by the Pain in Motion group.[84]
- *Brief Symptom Inventory (BSI)*. The inventory is a 53-item reported questionnaire which assesses patient's current level of psychological distress with a 5-point Likert scale questions (0 - not at all, 4 - extremely), the amount of symptoms present, and their severity.[85] BSI is composed of nine subscales: somatization, obsession-compulsion, interpersonal sensitivity, depression, anxiety, hostility, phobic anxiety, paranoid ideation, and psychoticism. For the purposes of the study the Dutch translation of the BSI was used, which has shown good internal consistency (Cronbach α = 0.71-0.87) and test-retest reliability (r = 0.71-0.89) among psychiatric out-patient and healthy individual groups.[86]

Sample size

Due to the limited amount of relevant previous or similar studies, the sample sizes for the several analyses described in this research protocol are estimated a priori with GPower (G*Power for Windows, Version 3.1.9.2), and are exploratory as a result.

For peer review only

The following sample size calculations as well as the planned statistical analyses later described will be performed with a proxy for each of the main variables being three variables for CS and four for functioning. Additionally, due to the influence of patient's sex and age in the assessment outcomes, all analyses will include them for a moderator effect.

Sample size for objective 1: To analyze the association between instruments assessing symptoms and signs attributed to CS.

Correlation analyses will be performed for this objective. Because the correlation between the different CS measurements is expected to be medium, for a correlation coefficient of 0.3, an α error probability of 0.05, and a power of 0.8, a minimum sample size of 84 is calculated.

Sample size for objective 2: To analyze whether symptoms and signs attributed to CS are associated with functioning measurement outcomes; and for objective 3: To analyze whether changes (between baseline and discharge) in symptoms and signs attributed to CS are related to changes in each of the functioning measurement outcomes.

Multiple regression analyses will be performed for objectives 2 and 3. Based on the associations between CSI and psychosocial and cognitive behavioral factors,[30] the effect size is hypothesized to be medium. Because minimally three CS measurements are tested with sex and age required as independent variables, and controlled for the confounding effect of other demographic and test-related variables, eight predictors are foreseen. Considering the factors mentioned above into account, the sample size calculation results in a minimum of 77 patients, calculated with an α error probability of 0.05 and a power of 0.8. Finally, patients measured at discharge could be about 30% less than at baseline due to either not following the interdisciplinary rehabilitation program or personal reasons, a sample size of 110 patients may be required in this scenario.

Analyses

Data records from the different tests and questionnaires will be collected and merged into one database. The database management will be managed by the project team and handled according to the internal regulations of the UMCG. The analyses of the study will be performed with SPSS software version 22.0 (IBM Corp., NY).

Before any analysis, data will be prepared:

- Variables need to be calculated: BMI; DMA, WUR, and CPM tests (see Methods section, Main measurements: CS); PDI, Rand36-PF, PCS, BSI, and IEQ questionnaires.
- HRV: data will be pre-processed to correct for any artifacts that may be present at the beginning or at the end of the recording.

For peer review only

- QST: all measurements will be transformed to standard normal distribution (Z-transformation).
- To perform the analyses to respond objective 3, Delta scores are computed (difference in scores between baseline and discharge) for CS, functioning, and those confounder variables which are also measured at the same time points as the main measurements.

Proxies of the main measurements were selected to perform analyses. CS variables are: the sum score of CSI part A, the RMSSD for HRV, and the MPT for QST. Functioning variables are: the maximal weight lifted for lifting capacity, the VO2max per kg for the maximal aerobic capacity, the daily VM counts for PA, and the average score of Rand36-PF for reported physical functioning.

The presence of missing data will be checked. If a variable has more than 10% of missing data cases, its distribution will be further examined. When the distribution of missing data is missing completely at random, analyses with pairwise deletions are planned. If data is not missing completely at random, other suitable solutions will be applied such as multiple imputation.[87] Regarding the presence of outliers, cases at 3 SD from the mean will be considered as outliers. Their influence will be measured with Cook's distance and leverage values. When the outliers are not statistically influential and/or there is rationale for the outlier, no further actions are planned. The descriptive statistics of the sample characteristics and main test results will be at this point calculated; numerical variables as mean and SD in normally distributed data and as median and interquartile range (IQR 25-75) in non-normally distributed data, and categorical variables as counts and percentage of each category level.

Statistical approach for objective 1: To analyze the association between instruments assessing symptoms and signs attributed to CS.

Multiple partial correlation analyses will be performed corrected for potential confounders. CS measurements will be the main variables and sex and age will be covariates. Additional covariates may enter the partial correlations based on their correlation coefficient with CS instruments and if $p<0.01$. Results are reported correlation coefficients (Pearson's r and/or Spearman's rho) and p-values, with and without controlling for covariates. The significance level for all analyses is established at 0.01 level.

Statistical approach for objective 2: To analyze whether symptoms and signs attributed to CS are associated with functioning measurement outcomes.

First multiple correlation analyses, followed by multiple regression analyses with data obtained at baseline will be performed. Each of the main outcomes from functioning measurements will be introduced in the models as dependent variable, CS measurements as independent variables, and sex, age, and other demographic characteristics and/or test-related variables as potential confounders and/or moderators.

For peer review only

Statistical approach for objective 3: To analyze whether changes (between baseline and discharge) in symptoms and signs attributed to CS are related to changes in each of the functioning measurement outcomes.

Similar approach to objective 2 will be performed: first multiple correlation analyses will be performed with data obtained at baseline and discharge, followed by multiple correlation analyses with Delta scores of the data, and finally multiple regression analyses again with Delta scores. As in objective 2, each of the main outcomes from functioning measurements will be introduced in the models as dependent variable, CS measurements as independent variables, and sex, age, and other demographic characteristics and/or test-related variables as potential confounders and/or moderators.

Confounders entering the final models of objectives 2 and 3 will be selected based on their partial correlation coefficient with CS and functioning and they will be included if $p \leq 0.10$. All multiple regression models will have the assumptions checked. Results of objectives 2 and 3 will be reported as coefficient (b), confidence intervals (95% CI), p-values, and explained variance, with and without controlling for confounders and/or moderators. The significance level of the correlation analyses is established at 0.01 level and of the multiple regression analyses is established at 0.05 level.

For peer review only

DISCUSSION

Several patients with CLBP have shown common manifestations with other chronic pain conditions, which may evidence the presence of CS in these patients. Yet knowledge on CS is developing, especially in patients with CLBP, and evidence explaining its association with functioning is lacking. This research protocol aims to bridge that gap of knowledge with a novel approach. In the first place, the study (objective 1) will describe the mechanisms of CS in patients with CLBP with instruments that assess CS symptoms from different perspectives. Furthermore, the study (objectives 2 and 3) will also analyze longitudinally whether the presence of CS and changes of CS can be associated with changes physical functioning performance, for which functioning will be assessed with several objective monitoring and capacity tests, and self-reported measurements.

Within the constraints of CAU and in absence of a single gold standard instrument that can diagnose CS, in this research protocol multiple instruments are selected to examine symptoms and signs attributed to CS. 1) The hypersensitivity unrelated to mechanical stimulus are assessed with CSI. A questionnaire designed to measure CS as it has been shown to be associated to factors known to contribute to CS and is able to identify somatic and emotional symptoms related to CSS.[34,88] 2) The disproportionate pain, diffuse distribution, and allodynia and hyperalgesia, are measured with QST. The QST is a battery of non-invasive standardized tests that assists in the identification of sensory and pain thresholds, the determination of pain intensity perception, and the assessment of the conditioned pain modulation. Its results can allow pain characterization (phenotyping) and processing, and monitoring its

progression.[89-93] 3) The low vagal nerve activity is examined with HRV. A non-invasive standardized measure that characterizes the function of the ANS.[20] The somatic and emotional symptoms related to CS syndromes may generate an imbalance between a hyperactivation of the sympathetic tone and a reduction of the parasympathetic/vagal tone.[20,94,95] 4) Psychosocial factors are measured with psychological screening questionnaires for perceived injustice (IEQ), catastrophizing (PCS), and psychological distress (BSI). These psychosocial factors are not necessary for the clinical classification of CS but are included in the study, and potentially in the analyses, due to their consistent effect on functioning and on symptom beliefs and pain behaviors common in chronic pain patient including CS syndromes. Some of the CS assessment instruments used in this study are more acknowledged than others in CS literature. CSI is widely used to measure CS as it was designed as a proxy for it; however, its internal consistency and reliability in patients with CLBP has not yet been made available. Also, currently evidence is piling up on the necessity of routinely implementing the elements of QST relevant to patients' conditions in clinical practice due to being able to assess the somatosensory function,[93,96] even though evidence in patients with CLBP is limited. Finally, literature on HRV as an instrument that may reflect an imbalance of ANS in patients with chronic pain conditions, specifically in those patients that have shown an overlap in the CS symptoms, is scarce. We are confident that the selected CS assessment instruments are considered best available, and will be able to comprehensively measure from different perspectives the clinical symptoms and signs indicative of CS being present, to provide further insights on the mechanisms of CS and to assist on the phenotyping of centrally sensitized patients with CLBP.

Various functioning assessments are selected for the research protocol to determine the functioning status of centrally sensitized patients with CLBP, including several objective monitoring and capacity tests, and self-reported measurements. 1) The floor-to-waist lifting capacity test is a physical assessment from the FCE that has demonstrated the best predictability for Return-to-Work in CLBP.[97] 2) The aerobic capacity, expressed as VO2max, can be best assessed with a maximal aerobic capacity test such as the CPET. The cycle ergometer and the treadmill are the most used assessments for aerobic capacity, but the cycle ergometer is most appropriate for patients.[33] 3) The PA is measured with an accelerometer, an easy wearable activity-monitoring device that provides objective measurements of PA levels and PA distribution or patterns. 4) Patient-reported functioning questionnaires present patients pain experience, how they perceive or believe their functioning and participation is. The work ability (WAI), pain disability, and Rand36 Physical Functioning subscale additionally show the advantage of being of short length and easy to interpret due to their interval scale. Although questionnaires results can differ from their actual physical performance, both measurements complement each other and together they may provide a more comprehensive overview of patients with CLBP.[98-100] Assessments such as lifting capacity and self-reported measurements have been routinely used to determine the functioning status of patients with CLBP. CPET on the other hand, even though it is scientifically

For peer review only - http://bmjopen.bmj.com/site/about/guidelines.xhtml

superior to a submaximal aerobic capacity test, has barely been implemented with patients with CLBP and, as a result, whether a maximal CPET could be applied systematically with these patients and their pain response to it is unknown. Moreover, even if there is a large amount of literature on PA, the methodology implemented is very broad regarding population groups and/or measuring instruments, which hinders the synthesis of the understanding of PA of patients with CLBP. To bridge the gap, it may be of use an objective measurement of PA and monitored with accelerometers such as those of ActiGraph, who has shown to produce the most reliable devices for recording PA in free-living conditions.[101,102] It is believed that the assessments described are suitable and relevant for the purpose of measuring functioning from multiple domains, and that the extended knowledge and the better understanding of the mechanisms involved obtained is not limited to CLBP but can be extended to CS.

Data collected for this project is large and the measurements of CS and functioning collected involve a broad amount of variables, as a result proxies will be used for the statistical analyses. Previous research in CS assessments, on the one hand, and in functioning assessments, on the other, has reported different methodologies and outcome parameters. Reason for which, even if authors have selected the proxy variables based on their relevance and contribution to the purposes of the current studies, the selection of the following measurements as proxies may not be beyond debate: for HRV the RMSSD, as it is a measurement of the parasympathetic/vagal tone which is not influenced by breathing;[103] for QST the MPT, since it measures the perceived skin mechanical hyperalgesia which is one of the main indicators of CS;[12] for the maximal aerobic capacity the VO2max corrected for the body mass of the individual, due to the effect of body mass on peak oxygen uptake; and for PA the daily VM counts, because it characterizes movement duration and intensity.[104]

Notwithstanding the efforts of the team in the design and implementation to obtain the most extensive insights of CS and its association with functioning, it should be considered that patients participating are volunteers; and that the exclusion criteria, although based on patient's file, is dependent on doctor's judgment, which may imply some subjectivity. These circumstances may have some influence in the results; however, their impact of is so far unknown to the authors. Likewise, due to the CAU design of the study the choice of measurements have to be feasible and compatible with the regular clinical procedures. As a result, the fMRI a commonly mentioned instrument to measure CS could not be performed in the study. The HRV on the other hand, although less often used, can be an insightful and an innovative approach to the assessment of one other indicator of the presence of CS. Additionally, the sample sizes are estimated a priori for the analyses due to insufficient previous studies and, as a result, they are of exploratory nature and used as a guide. Also, despite the efforts of the team to promote the study participation and participant retention, it is likely that patient recruitment may be slower than anticipated and that patient may not have a complete follow-up. However, since the measurement period is ample, the calculations are vigilantly made with generous margins (see

For peer review only

Methods section, Sample size), and the literature related to the topic has shown smaller sample sizes;[30,105] we remain positive about the sample size and expect to have more than enough participants to perform analyses with sufficient power. Also, measurements are planned to be within three weeks from the actual baseline and discharge assessments performed by the Pain Rehabilitation team for comparability of the results. The CSI, PDI, and psychological screening questionnaires during baseline assessment are sent to be filled six weeks before the rest of the measurements are obtained, a time distance due to waiting list time. Since no important changes in patients with respect to medication, surgery, or personal conditions are foreseen in the meanwhile and the questionnaires have shown to have good clinimetric properties, these measurements are expected to be comparable to the rest of measurements at baseline. As opposed to the limitations presented, in sight of the large amount of correlations in the multiple correlation analyses, a strict significance level was applied, i.e. 0.01. We are confident that the results obtained will provide meaningful and valuable information.

All things considered, the results obtained from the measurements described in this research protocol can help better describe and understand the mechanisms involved in CS and, of these, the ones affecting the functioning of patients with CLBP; and then be used to optimize improve the effectiveness of pain management interventions. To the authors' knowledge, the study described in this research protocol are the first off its kind and it may become an important breakthrough for researchers involved in the study of pain, for the treatment strategy of patients with chronic pain by healthcare professionals, and for patient's pain education.

ETHICS AND DISSEMINATION

All participating patients sign the informed consent before any assessment takes place. Formal ethical clearance to perform the study has been obtained from the METC University Medical Center Groningen (The Netherlands) in July 2017 (METC 2016/702). All procedures are in accordance with the ethical standards of the Helsinki Declaration of 1975 as revised in 2014 [106] and with Dutch regulations of the Medical Research Involving Human Subjects Act, in Dutch: Wet Medisch-Wetenschappelijk Onderzoek (WMO), amended in 2018.[107]

The dissemination strategy of the research study results to will be through scientific publications in peer-reviewed journals and presentations in relevant national and international conferences. In addition to it, reports will be sent by post to participants and reports and presentations in meetings will be provided to stakeholders.

For peer review only - http://bmjopen.bmj.com/site/about/guidelines.xhtml

1 **ABBREVIATIONS**

ANS	Autonomic Nervous System
BF	Breathing Frequency at the end of CPET
BMI	Body Mass Index
BSI	Brief Symptom Inventory
CAU	Care As Usual
cCS	cold Conditioning Stimulus
CLBP	Chronic Low Back Pain
CPET	Cardio-Pulmonary Exercise Test
CPM	Conditioned Pain Modulation
CS	Central Sensitization
CSI	Central Sensitization Inventory
CSS	Central Sensitization Syndromes
CvR-UMCG	Center for Rehabilitation of the UMCG; in Dutch: Centrum voor Revalidatie
CR-10 scale	Category Ratio (0-10) scale
DMA	Dynamic Mechanical Allodynia
DFNS	German Research Network on Neuropathic Pain; in German: Deutschen Forschungsverbund Neuropathischer Schmerz
DOT	Dictionary of Occupational Titles
ECG	Electrocardiogram
FCE	Functional Capacity Evaluation
HF	High Frequency
HR	Heart Rate
HRV	Heart Rate Variability
IC	Informed Consent
ICC	Intra-Class Correlation
IEQ	Injustice Experience Questionnaire
LF	Low Frequency
MDT	Mechanical Detection Threshold
MET	Metabolic Equivalent of Task
METc	Medical Research Ethics committee; in Dutch: Medisch Ethische Toetsing commissie
MPT	Mechanical Pain Threshold
NASQ	Nijmegen-Aalborg Screening QST
NRS	Numeric Rating Scale
PA	Physical Activity
PCS	Pain Catastrophizing Scale
PDI	Pain Disability Index
PF	Physical Functioning
PPT	Pressure Pain Threshold
PRQ	Pain Response Questionnaire
QST	Quantitative Sensory Testing
Rand36-PF	Physical Functioning subscale of the Rand36 questionnaire
RER	Respiratory Exchange Ratio

For peer review only

rMSSD	Root Mean Square of Successive Differences between NN intervals
RPE	Rating of Perceived Exertion
RR interval	Inter-beat interval
SDNN	Standard Deviation of Normal-to-Normal (NN) intervals
TS	Test Stimulus
UMCG	Universitair Medisch Centrum Groningen
VAS	Visual Analogue Scale
VLF	Very Low Frequency
VO2max	Peak VO2, Peak oxygen uptake
VT	Expiratory Tidal Volume
WAI	Work Ability Index
WAS	Work Ability Score
WMO	Medical Research Involving Human Subjects Act; in Dutch: Wet Medisch-wetenschappelijk Onderzoek met Mensen
WUR	Wind-Up Ratio
YLD	Years Lived with Disability

For peer review only

STATEMENTS

Author's Contributions: JAE was responsible for the study concept and design, data collection, and manuscript preparation and edit; HRSP, RD, and MR were responsible for the study concept and design, and critical review of the manuscript; IS, HT and APW were responsible for the study design and critical review of the manuscript.

Competing interests: The authors declare that there are no conflicts of interest.

Funding: The Pain Rehabilitation Development Center (in Dutch: Ontwikkelcentrum PijnRevalidatie (OPR)) of the CvR-UMCG funded the research study.

Trial registration: NTR7167, April 16th 2018; retrospectively registered.

Ethics approval: The Medical Research Ethics Committee of the University Medical Center Groningen (METc UMCG) provided a positive reaction in July 2017 to the development of the research study "*Central Sensitization and Functioning in patients with Chronic Low Back Pain*" (METc 2016/702). Its first amendment was approved in October 2017 and its second amendment in August 2018.

For peer review only

REFERENCES

1. The Joint Task Force (JTF). ICD-11 Beta Draft - Mortality and Morbidity Statistics[Internet]. Available from: https://icd.who.int/browse11/l-m/en#/ (Accessed 24 May 2018).
2. Breivik H, Collett B, Ventafridda V, et al. Survey of chronic pain in Europe: Prevalence, impact on daily life, and treatment. Eur J Pain. 2006;10(4):287–333.
3. Hoy D, March L, Brooks P, et al. The global burden of low back pain: estimates from the Global Burden of Disease 2010 study. Ann Rheum Dis. 2014;73(6):968–74.
4. Vos T, Flaxman AD, Naghavi M, et al. Years lived with disability (YLDs) for 1160 sequelae of 289 diseases and injuries 1990–2010: A systematic analysis for the Global Burden of Disease Study 2010. Lancet. 2012;380(9859):2163–96.
5. Foster NE, Anema JR, Cherkin D, et al. Prevention and treatment of low back pain: evidence, challenges, and promising directions. Lancet. 2018;391(10137):2368–83.
6. Boonen A, van den Heuvel R, van Tubergen A, et al. Large differences in cost of illness and wellbeing between patients with fibromyalgia, chronic low back pain, or ankylosing spondylitis. Ann Rheum Dis. 2005;64:396–402.
7. Dagenais S, Caro J, Haldeman S. A systematic review of low back pain cost of illness studies in the United States and internationally. Spine J. 2008;8(1):8–20.
8. Lambeek LC, van Tulder MW, Swinkels ICS, et al. The Trend in Total Cost of Back Pain in the Netherlands in the Period 2002 to 2007. Spine. 2011;36(13):1050–8.
9. Giesecke T, Gracely RH, Grant MAB, et al. Evidence of augmented central pain processing in idiopathic chronic low back pain. Arthritis Rheum. 2004;50(2):613–23.
10. Woolf CJ. Central sensitization: implications for the diagnosis and treatment of pain. Pain. 2011;152(3 Suppl.):S2–15.
11. Arendt-Nielsen L, Morlion B, Perrot S, et al. Assessment and manifestation of central sensitisation across different chronic pain conditions. Eur J Pain. 2018;22(2):216–41.
12. den Boer C, Dries L, Terluin B, et al. Central sensitization in chronic pain and medically unexplained symptom research: A systematic review of definitions, operationalizations and measurement instruments. J Psychosom Res. 2019;117:32–40.
13. Nijs J, Torres-Cueco R, van Wilgen P, et al. Applying Modern Pain Neuroscience in Clinical Practice: Criteria for the Classification of Central Sensitization Pain. Pain Physician. 2014;17(5):447–57.
14. Smart KM, Blake C, Staines A, et al. The Discriminative Validity of "Nociceptive," "Peripheral Neuropathic," and "Central Sensitization" as Mechanisms-based Classifications of Musculoskeletal Pain. Clin J Pain. 2011;27(8):655–

For peer review only - http://bmjopen.bmj.com/site/about/guidelines.xhtml

63.

15. De Couck M, Nijs J, Gidron Y. You May Need a Nerve to Treat Pain: The Neurobiological Rationale for Vagal Nerve Activation in Pain Management. Clin J Pain. 2014 ;30(12):1099–105.

16. Smart KM, Blake C, Staines A, et al. Mechanisms-based classifications of musculoskeletal pain: Part 1 of 3: Symptoms and signs of central sensitisation in patients with low back (±leg) pain. Man Ther. 2012; 17(4), 336–44.

17. Mayer TG, Neblett R, Cohen H, et al. The development and psychometric validation of the central sensitization inventory. Pain Pract. 2012;12(4):276–85.

18. Rolke R, Baron R, Maier C, et al. Quantitative sensory testing in the German Research Network on Neuropathic Pain (DFNS): Standardized protocol and reference values. Pain. 2006;123(3):231–43.

19. Wilder-Smith O. A paradigm-shift in pain medicine: implementing a systematic approach to altered pain processing in everyday clinical practice based on quantitative sensory testing. Center for Sensory-Motor Interaction (SMI), Department of Health Science and Technology, Aalborg University. 2013:76p.

20. McCraty R, Atkinson M, Tomasino D, et al. The coherent heart: heartbrain interactions, psychophysiological coherence, and the emergence of system-wide order. Boulder Creek, CA: Institute of Heartmath; 2009.

21. O'Brien T, Breivik H. The impact of chronic pain — European patients' perspective over 12 months. Scand J Pain. 2012;3(1):23–9.

22. Nugraha B, Gutenbrunner C, Barke A, et al. The IASP classification of chronic pain for ICD-11. Pain. 2019;160(1):88–94.

23. Cieza A, Stucki G, Weigl M, et al. ICF Core Sets for low back pain. J Rehabil Med. 2004; Suppl. 44:69–74.

24. Schneidert M, Hurst R, Miller J, et al. The role of Environment in the International Classification of Functioning, Disability and Health (ICF). Disabil Rehabil. 2003;25(11–12):588–95.

25. Verbunt JA, Seelen HA, Vlaeyen JW, et al. Disuse and deconditioning in chronic low back pain: concepts and hypotheses on contributing mechanisms. Eur J Pain. 2003;7(1):9–21.

26. Ryan CG, Grant PM, Dall PM, et al. Individuals with chronic low back pain have a lower level, and an altered pattern, of physical activity compared with matched controls: An observational study. Aust J Physiother. 2009;55(1):53–8.

27. van Dieën JH, Reeves NP, Kawchuk G, et al. Motor Control Changes in Low-Back Pain: Divergence in Presentations and Mechanisms. J Orthop Sport Phys Ther. 2018;1–24.

28. Hübscher M, Moloney N, Leaver A, et al. Relationship between quantitative sensory testing and pain or disability in people with spinal pain—A systematic review and meta-analysis. Pain. 2013; 154(9), 1497–504.

29. Gockel M, Lindholm H, Niemistö L, et al. Perceived disability but not pain is connected with autonomic nervous function among patients with chronic low back pain. J Rehabil Med. 2008;40(5):355-8.

For peer review only

For peer review only - http://bmjopen.bmj.com/site/about/guidelines.xhtml

30. Huysmans E, Ickmans K, Van Dyck D, et al. Association Between Symptoms of Central Sensitization and Cognitive Behavioral Factors in People With Chronic Nonspecific Low Back Pain: A Cross-sectional Study. J Manipulative Physiol Ther. 2018;41(2):92-101.

31. IASP Taskforce for the Classification of Chronic Pain. The IASP classification of chronic pain for ICD-11: chronic primary pain. Pain. 2019; 160(1); 28-37.

32. Hart DL, Isernhagen SJ, Matheson LN. Guidelines for Functional Capacity Evaluation of People With Medical Conditions. J Orthop Sport Phys Ther. 1993;18(6):682–6.

33. American Thoracic Society, American College of Chest Physicians. ATS/ACCP Statement on Cardiopulmonary Exercise Testing. Am J Respir Crit Care Med. 2003;167(2):211–77.

34. Neblett R, Cohen H, Choi Y, et al. The Central Sensitization Inventory (CSI): establishing clinically significant values for identifying central sensitivity syndromes in an outpatient chronic pain sample. J Pain. 2013;14(5):438–45.

35. Neblett R, Hartzell MM, Mayer TG, et al. Establishing Clinically Relevant Severity Levels for the Central Sensitization Inventory. Pain Pract. 2017;17(2):166–75.

36. Scerbo T, Colasurdo J, Dunn S, et al. Measurement Properties of the Central Sensitization Inventory: A Systematic Review. Pain Pract. 2018;18(4):544–54.

37. van Wilgen CP, Meeus M, Descheemaeker F, et al. Central Sensitization Inventory – Nederlandse consensusvertaling [Internet]. 2013. Available from: http://www.paininmotion.be/storage/app/media//materials/CSI-consensusvertaling.pdf (Accessed 19 Jul 2018).

38. Kregel J, Vuijk PJ, Descheemaeker F, et al. The Dutch Central Sensitization Inventory (CSI). Clin J Pain. 2016;32(7):624–30.

39. Timmerman H, Wilder-Smith O, van Weel C, et al. Detecting the neuropathic pain component in the clinical setting: a study protocol for validation of screening instruments for the presence of a neuropathic pain component. BMC Neurol. 2014;14:94–102.

40. Geber C, Klein T, Azad S, et al. Test–retest and interobserver reliability of quantitative sensory testing according to the protocol of the German Research Network on Neuropathic Pain (DFNS): A multi-centre study. Pain. 2011;152(3):548–56.

41. LaMotte RH, Shain CN, Simone DA, et al. Neurogenic hyperalgesia: psychophysical studies of underlying mechanisms. J Neurophysiol. 1991;66(1):190–211.

42. Ziegler EA, Magerl W, Meyer RA, et al. Secondary hyperalgesia to punctate mechanical stimuli. Brain. 1999;122(12):2245–57.

43. Chan AW, MacFarlane IA, Bowsher D, et al. Weighted needle pinprick sensory thresholds: a simple test of sensory

For peer review only

function in diabetic peripheral neuropathy. J Neurol Neurosurg Psychiatry. 1992;55(1):56–9.

44. Greenspan JD, McGillis SLB. Thresholds for the Perception of Pressure, Sharpness, and Mechanically Evoked Cutaneous Pain: Effects of Laterality and Repeated Testing. Somatosens Mot Res. 1994;11(4):311–7.
45. Hampf G, Bowsher D, Wells C, et al. Sensory and autonomic measurements in idiopathic trigeminal neuralgia before and after radiofrequency thermocoagulation: differentiation from some other causes of facial pain. Pain. 1990;40(3):241–8.
46. Price DD, Hu JW, Dubner R, et al. Peripheral suppression of first pain and central summation of second pain evoked by noxious heat pulses. Pain. 1977;3(1):57–68.
47. Magerl W, Wilk SH, Treede R-D. Secondary hyperalgesia and perceptual wind-up following intradermal injection of capsaicin in humans. Pain. 1998;74(2–3):257–68.
48. Weinstein S. Intensive and extensive aspects of tactile sensitivity as a function of body part, sex and laterality. In: Kenshalo DR, ed. The skin senses: Proceedings. Springfield, IL, US: Charles C Thomas. 1968:195–222.
49. Fruhstorfer H, Gross W, Selbmann O. von Frey hairs: new materials for a new design. Eur J Pain. 2001;5(3):341–2.
50. Baumgärtner U, Magerl W, Klein T, et al. Neurogenic hyperalgesia versus painful hypoalgesia: two distinct mechanisms of neuropathic pain. Pain. 2002;96(1–2):141–51.
51. Yarnitsky D, Bouhassira D, Drewes AM, et al. Recommendations on practice of conditioned pain modulation (CPM) testing. Eur J Pain. 2015;19(6):805–6.
52. Imai Y, Petersen KK, Mørch CD, et al. Comparing test–retest reliability and magnitude of conditioned pain modulation using different combinations of test and conditioning stimuli. Somatosens Mot Res. 2016;33(3–4):169–77.
53. McCorry LK. Physiology of the autonomic nervous system. Am J Pharm Educ. 2007;71(4):78–88.
54. Task Force of the European Society of Cardiology and the North American Society of Pacing and Electrophysiology. Heart rate variability, standards of measurement, physiological interpretation, and clinical use. Circulation. 1996;93(5):1043–65.
55. McCraty R, Shaffer F. Heart Rate Variability: New Perspectives on Physiological Mechanisms, Assessment of Self-regulatory Capacity, and Health Risk. Glob Adv Heal Med. 2015;4(1):46–61.
56. HeartMath Inc. emWave Pro Plus Assessments [Internet]. 2018. Available from: https://heartcloud.com/library/asmts.html (Accessed 27 Feb 2019).
57. Isernhagen SJ, Hart DL, Matheson LM. Reliability of independent observer judgments of level of lift effort in a kinesiophysical Functional Capacity Evaluation. Work. 1999;12(2):145–50.
58. de Baets S, Calders P, Schalley N, et al. Updating the Evidence on Functional Capacity Evaluation Methods: A Systematic Review. J Occup Rehabil. 2018;28(3):418–28.

For peer review only

59. Brouwer S, Reneman MF, Dijkstra PU, et al. Test–Retest Reliability of the Isernhagen Work Systems Functional Capacity Evaluation in Patients with Chronic Low Back Pain. J Occup Rehabil. 2003;13(4):207–18.

60. Soer R, van der Schans CP, Geertzen JH, et al. Normative Values for a Functional Capacity Evaluation. Arch Phys Med Rehabil. 2009;90(10):1785–94.

61. Borg G. Borg's perceived exertion and pain scales.. Champaign, IL, US: Human Kinetics; 1998.

62. Reneman MF, Fokkens AS, Dijkstra PU, et al. Testing Lifting Capacity: Validity of Determining Effort Level by Means of Observation. Spine. 2005;30(2):e40–6.

63. Scherr J, Wolfarth B, Christle JW, et al. Associations between Borg's rating of perceived exertion and physiological measures of exercise intensity. Eur J Appl Physiol. 2013;113(1):147–55.

64. Trost SG, McIver KL, Pate RR. Conducting Accelerometer-Based Activity Assessments in Field-Based Research. Med Sci Sports Exerc. 2005;37(11 Suppl.):S531–43.

65. Migueles JH, Cadenas-Sanchez C, Ekelund U, et al. Accelerometer Data Collection and Processing Criteria to Assess Physical Activity and Other Outcomes: A Systematic Review and Practical Considerations. Sport Med. 2017;47(9):1821–45.

66. Keadle SK, Shiroma EJ, Freedson PS, et al. Impact of accelerometer data processing decisions on the sample size, wear time and physical activity level of a large cohort study. BMC Public Health. 2014;14(1):1210.

67. Sasaki JE, John D, Freedson PS. Validation and comparison of ActiGraph activity monitors. J Sci Med Sport. 2011;14(5):411–6.

68. Crouter SE, Kuffel E, Haas JD, et al. Refined two-regression model for the ActiGraph accelerometer. Med Sci Sports Exerc. 2010;42(5):1029–37.

69. van der Zee KI, Sanderman R. RAND-36. umcg.nl [Internet]. Available from: https://www.umcg.nl/SiteCollectionDocuments/research/institutes/SHARE/assessment tools/handleiding_rand36_2e_druk.pdf (Accessed 28 Jun 2018).

70. van der Zee KI, Sanderman R, Heyink JW, et al. Psychometric qualities of the rand 36-item health survey 1.0: A multidimensional measure of general health status. Int J Behav Med. 1996;3(2):104–22.

71. Tait RC, Chibnall JT, Krause S. The Pain Disability Index: psychometric properties. Pain. 1990;40(2):171–82.

72. Soer R, Köke AJA, Vroomen PCAJ, et al. Extensive Validation of the Pain Disability Index in 3 Groups of Patients With Musculoskeletal Pain. Spine. 2013;38(9):e562–8.

73. de Zwart BCH, Frings-Dresen MHW, van Duivenbooden JC. Test-retest reliability of the Work Ability Index questionnaire. Occup Med. 2002;52(4):177–81.

74. Ahlstrom L, Grimby-Ekman A, Hagberg M, et al. The work ability index and single-item question: associations with sick leave, symptoms, and health – A prospective study of women on long-term sick leave. Scand J Work Environ

For peer review only

Heal. 2010;36(5):404–12.

75. El Fassi M, Bocquet V, Majery N, et al. Work ability assessment in a worker population: comparison and determinants of Work Ability Index and Work Ability score. BMC Public Health. 2013;13:305–14.

76. U.S. Department of Labor, Employment and Training Administration. Dictionary of Occupational Titles. 4th ed. Washington; 1991.

77. Hawker GA, Mian S, Kendzerska T, et al. Measures of adult pain: Visual Analog Scale for Pain (VAS Pain), Numeric Rating Scale for Pain (NRS Pain), McGill Pain Questionnaire (MPQ), Short-Form McGill Pain Questionnaire (SF-MPQ), Chronic Pain Grade Scale (CPGS), Short Form-36 Bodily Pain Scale (SF36 BPS), and Measure of Intermittent and Constant Osteoarthritis Pain (ICOAP). Arthritis Care Res. 2011;63(S11):S240–52.

78. Soer R, Groothoff JW, Geertzen JHB, et al. Pain Response of Healthy Workers Following a Functional Capacity Evaluation and Implications for Clinical Interpretation. J Occup Rehabil. 2008;18(3):290–8.

79. Adams LM, Turk DC. Psychosocial Factors and Central Sensitivity Syndromes. Curr Rheumatol Rev. 2015 ; 11(2): 96–108.

80. Sullivan MJL, Bishop SR, Pivik J. The pain catastrophizing scale: development and validation. Psychol Assess. 1995;7(4):524–32.

81. Crombez G, Vlaeyen JWS. Pain Catastrophizing Scale (PCS) - Dutch Version (PCS-DV) [Internet]. 1996. Available from: https://meetinstrumentenzorg.blob.core.windows.net/test-documents/Instrument197/197_3_N.pdf (Accessed 8 Aug 2018).

82. van Damme S, Crombez G, Vlaeyen JWS, et al. De Pain Catastrophizing Scale: Psychometrische karakteristieken en normering. Gedragstherapie. 2000;33:209–20.

83. Rodero B, Luciano J V, Montero-Marín J, et al. Perceived injustice in fibromyalgia: Psychometric characteristics of the Injustice Experience Questionnaire and relationship with pain catastrophising and pain acceptance. J Psychosom Res. 2012;73(2):86–91.

84. van Wilgen CP, Nijs J, Don S, et al. The Injustice Experience Questionnaire: Nederlandstalige consensusvertaling [Internet]. 2014. Available from: http://www.paininmotion.be/storage/app/media//materials/IEQ-Dutch.pdf (Accessed 19 Jul 2018).

85. Derogatis LR. BSI brief symptom inventory: Administration, Scoring, and Procedure Manual (4th Ed.). Minneapolis, MN: National Computer Systems;1993.

86. de Beurs E, Zitman FG. The Brief Symptom Inventory (BSI): Reliability and validity of a practical alternative to SCL-90. Maandbl Geestelijke Volksgezond. 2006;61:120–41.

87. van Ginkel JR, Linting M, Rippe RC, et al. Rebutting Existing Misconceptions About Multiple Imputation as a

For peer review only - http://bmjopen.bmj.com/site/about/guidelines.xhtml

Method for Handling Missing Data. J Pers Assess. 2019; 1-12.

88. van Wilgen CP, Vuijk PJ, Kregel J, et al. Psychological Distress and Widespread Pain Contribute to the Variance of the Central Sensitization Inventory: A Cross-Sectional Study in Patients with Chronic Pain. Pain Pract. 2018;18(2):239–46.

89. Arendt-Nielsen L, Yarnitsky D. Experimental and Clinical Applications of Quantitative Sensory Testing Applied to Skin, Muscles and Viscera. J Pain. 2009;10(6):556–72.

90. Backonja M-M, Walk D, Edwards RR, et al. Quantitative Sensory Testing in Measurement of Neuropathic Pain Phenomena and Other Sensory Abnormalities. Clin J Pain. 2009;25(7):641–7.

91. Pavlaković G, Petzke F. The Role of Quantitative Sensory Testing in the Evaluation of Musculoskeletal Pain Conditions. Curr Rheumatol Rep. 2010;12(6):455–61.

92. Krumova EK, Geber C, Westermann A, et al. Neuropathic Pain: Is Quantitative Sensory Testing Helpful? Curr Diab Rep. 2012;12(4):393–402.

93. Uddin Z, MacDermid JC. Quantitative Sensory Testing in Chronic Musculoskeletal Pain. Pain Med. 2016;17(9):1694–703.

94. Martínez-Martínez L-A, Mora T, Vargas A, et al. Sympathetic Nervous System Dysfunction in Fibromyalgia, Chronic Fatigue Syndrome, Irritable Bowel Syndrome, and Interstitial Cystitis. JCR J Clin Rheumatol. 2014;20(3):146–50.

95. Tracy LM, Ioannou L, Baker KS, et al. Meta-analytic evidence for decreased heart rate variability in chronic pain implicating parasympathetic nervous system dysregulation. Pain. 2016;157(1):7–29.

96. Starkweather AR, Heineman A, Storey S, et al. Methods to measure peripheral and central sensitization using quantitative sensory testing: A focus on individuals with low back pain. Appl Nurs Res. 2016;29:237–41.

97. Kuijer PPFM, Gouttebarge V, Brouwer S, et al. Are performance-based measures predictive of work participation in patients with musculoskeletal disorders? A systematic review. Int Arch Occup Environ Health. 2012;85(2):109–23.

98. Reneman MF, Jorritsma W, Schellekens JMH, et al. Concurrent validity of questionnaire and performance-based disability measurements in patients with chronic nonspecific low back pain. J Occup Rehabil. 2002;12(3):119–29.

99. Brouwer S, Dijkstra PU, Stewart RE, et al. Comparing self-report, clinical examination and functional testing in the assessment of work-related limitations in patients with chronic low back pain. Disabil Rehabil. 2005;27(17):999–1005.

100. Caporaso F, Pulkovski N, Sprott H, et al. How well do observed functional limitations explain the variance in Roland Morris scores in patients with chronic non-specific low back pain undergoing physiotherapy? Eur Spine J. 2012;21(Suppl 2):S187–95.

101. Vanhelst J, Gottrand F, Baquet G, et al. Comparative Interinstrument Reliability of Uniaxial and Triaxial

For peer review only

Accelerometers in Free-Living Conditions. Percept Mot Skills. 2012;114(2):584–94.

102. Aadland E, Ylvisåker E. Reliability of the Actigraph GT3X+ Accelerometer in Adults under Free-Living Conditions. PLoS One. 2015;10(8):e0134606.

103. Laborde S, Mosley E, Thayer JF. Heart Rate Variability and Cardiac Vagal Tone in Psychophysiological Research – Recommendations for Experiment Planning, Data Analysis, and Data Reporting. Front Psychol. 2017;08:213.

104. Matthews CE, Hagströmer M, Pober DM, et al. Best practices for using physical activity monitors in population-based research. Med Sci Sports Exerc. 2012;44(Suppl 1):S68-76.

105. Clauw DJ, Williams D, Lauerman W, et al. Pain Sensitivity as a Correlate of Clinical Status in Individuals With Chronic Low Back Pain. Spine. 1999; 24(19), 2035-41.

106. General Assembly of the World Medical Association. World Medical Association Declaration of Helsinki: Ethical principles for medical research involving human subjects. J Am Coll Dent. 2014;81(3):14–8.

107. Dutch Government. The Medical Research Involving Human Subjects Act, in Dutch: Wet medisch-wetenschappelijk onderzoek met mensen (WMO) [Internet]. Available from: https://wetten.overheid.nl/BWBR0009408/2018-08-01 (Accessed 28 Feb 2019).

For peer review only

FIGURE LEGENDS

Figure 1. Flow-chart of the project procedure.

* , measurements obtained which do not belong to CAU of the Pain Rehabilitation of the CvR – UMCG .

For peer review only

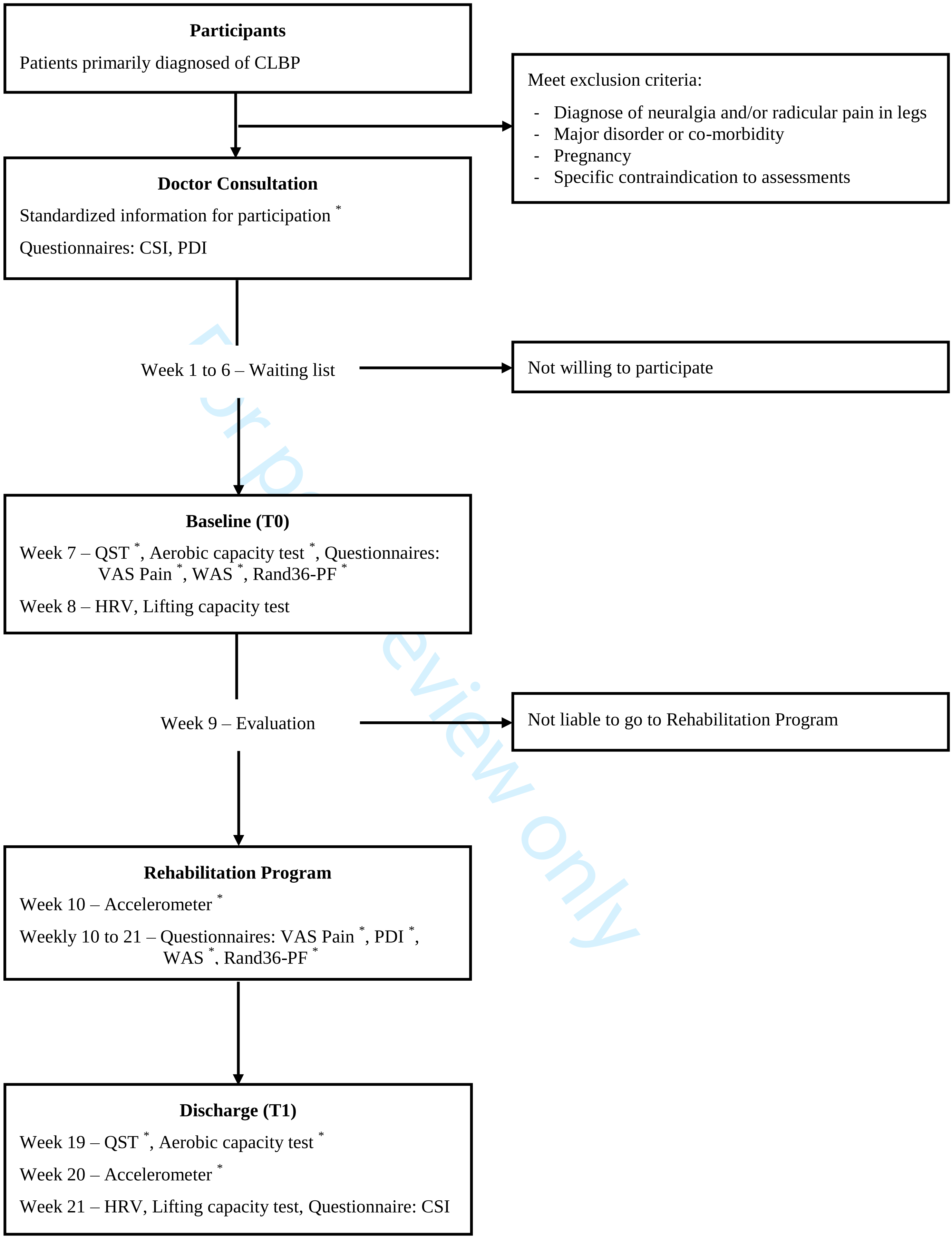
Participants
Patients primarily diagnosed of CLBP
Meet exclusion criteria:
- Diagnose of neuralgia and/or radicular pain in legs
- Major disorder or co-morbidity
- Pregnancy
- Specific contraindication to assessments
Doctor Consultation
Standardized information for participation *
Questionnaires: CSI, PDI
Week 1 to 6 – Waiting list
Not willing to participate
Baseline (T0)
Week 7 – QST *, Aerobic capacity test *, Questionnaires: VAS Pain *, WAS *, Rand36-PF *
Week 8 – HRV, Lifting capacity test
Week 9 – Evaluation
Not liable to go to Rehabilitation Program
Rehabilitation Program
Week 10 – Accelerometer *
Weekly 10 to 21 – Questionnaires: VAS Pain *, PDI *, WAS *, Rand36-PF *
Discharge (T1)
Week 19 – QST *, Aerobic capacity test *
Week 20 – Accelerometer *
Week 21 – HRV, Lifting capacity test, Questionnaire: CSI

SUPPLEMENTARY FILE

Supplemenary material 1. Description of the locations for the QST assessment.

Measuring Location	Description
**1. m. Deltoid – Training location**	In the pars medialis of m. Deltoid, at the contralateral side of the body to the most painful area located; 4 cm below the Acromion.<br>For the CPM test the measurement is at the non-dominant m. Deltoid.
**2. Most painful area**	As stated by the patient. This location is thoroughly described in the form to repeat the measurement in the same exact location at discharge.
**3. Low back – Control location**	Contralateral side of the body mirror-wise to the most painful area located. A specific description of the most painful area is important to identify this location as well.
**4. m. Trapezius – Control location**	In the pars medialis of m. Trapezius, both ipsilateral and contralateral to the most painful area located. At the level of the spine of the Scapula (T3); 4 cm lateral / external to the backbone.
**5. m. Quadriceps – Control location**	In the rectus femoris of m. Quadriceps, both ipsilateral and contralateral to the most painful area located; 15 cm above the upper edge of the Patella.<br>For the CPM test the measurement is at the non-dominant m. Quadriceps.

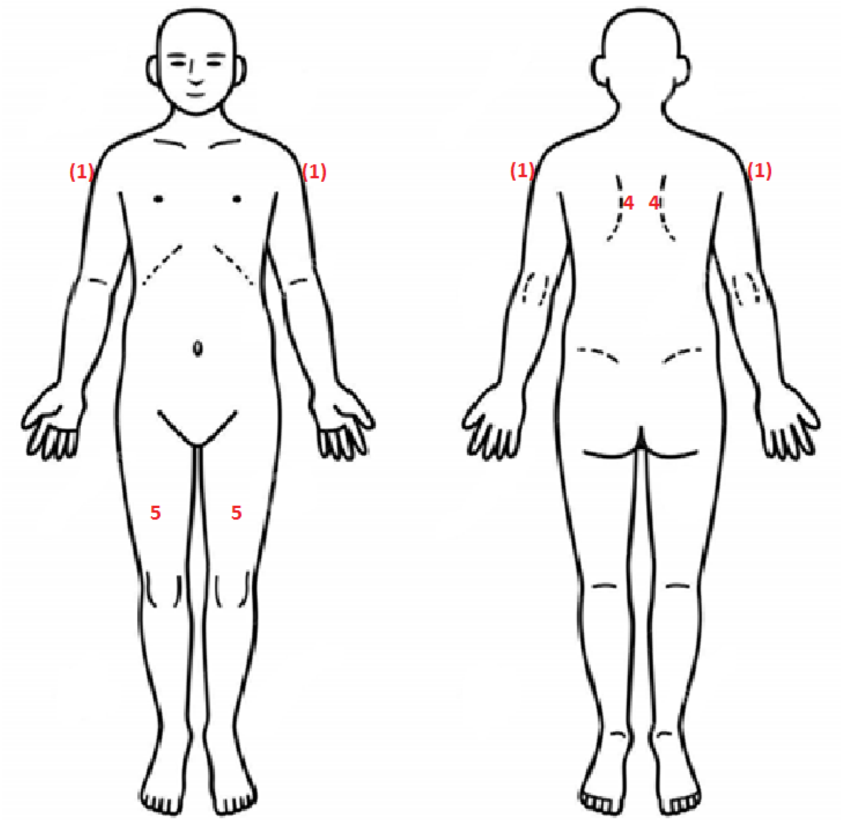

For peer review

SPIRIT

STANDARD PROTOCOL ITEMS: RECOMMENDATIONS FOR INTERVENTIONAL TRIALS

SPIRIT 2013 Checklist: Recommended items to address in a clinical trial protocol and related documents*

Section / Item	Item No.	Description	Check
**Administrative information**			
Title	1	Descriptive title identifying the study design, population, interventions, and, if applicable, trial acronym	X
Trial registration	2a	Trial identifier and registry name. If not yet registered, name of intended registry	X
	2b	All items from the World Health Organization Trial Registration Data Set	X
Protocol version	3	Date and version identifier	X
Funding	4	Sources and types of financial, material, and other support	X
Roles and responsibilities	5a	Names, affiliations, and roles of protocol contributors	X
	5b	Name and contact information for the trial sponsor	NA
	5c	Role of study sponsor and funders, if any, in study design; collection, management, analysis, and interpretation of data; writing of the report; and the decision to submit the report for publication, including whether they will have ultimate authority over any of these activities	NA
	5d	Composition, roles, and responsibilities of the coordinating centre, steering committee, endpoint adjudication committee, data management team, and other individuals or groups overseeing the trial, if applicable (see Item 21a for data monitoring committee)	NA
**Introduction**			
Background and rationale	6a	Description of research question and justification for undertaking the trial, including summary of relevant studies (published and unpublished) examining benefits and harms for each intervention	X
	6b	Explanation for choice of comparators	X
Objectives	7	Specific objectives or hypotheses	X
Trial design	8	Description of trial design including type of trial (eg, parallel group, crossover, factorial, single group), allocation ratio, and framework (eg, superiority, equivalence, noninferiority, exploratory)	X

For peer review only

Methods: Participants, interventions, and outcomes

Study setting	9	Description of study settings (eg, community clinic, academic hospital) and list of countries where data will be collected. Reference to where list of study sites can be obtained	**X**
Eligibility criteria	10	Inclusion and exclusion criteria for participants. If applicable, eligibility criteria for study centres and individuals who will perform the interventions (eg, surgeons, psychotherapists)	**X**
Interventions	11a	Interventions for each group with sufficient detail to allow replication, including how and when they will be administered	**NA**
	11b	Criteria for discontinuing or modifying allocated interventions for a given trial participant (eg, drug dose change in response to harms, participant request, or improving/worsening disease)	**NA**
	11c	Strategies to improve adherence to intervention protocols, and any procedures for monitoring adherence (eg, drug tablet return, laboratory tests)	**NA**
	11d	Relevant concomitant care and interventions that are permitted or prohibited during the trial	**NA**
Outcomes	12	Primary, secondary, and other outcomes, including the specific measurement variable (eg, systolic blood pressure), analysis metric (eg, change from baseline, final value, time to event), method of aggregation (eg, median, proportion), and time point for each outcome. Explanation of the clinical relevance of chosen efficacy and harm outcomes is strongly recommended	**X**
Participant timeline	13	Time schedule of enrolment, interventions (including any run-ins and washouts), assessments, and visits for participants. A schematic diagram is highly recommended (see Figure)	**X**
Sample size	14	Estimated number of participants needed to achieve study objectives and how it was determined, including clinical and statistical assumptions supporting any sample size calculations	**X**
Recruitment	15	Strategies for achieving adequate participant enrolment to reach target sample size	**X**

Methods: Assignment of interventions (for controlled trials) **NA**

Allocation:

Sequence generation	16a	Method of generating the allocation sequence (eg, computer-generated random numbers), and list of any factors for stratification. To reduce predictability of a random sequence, details of any planned restriction (eg, blocking) should be provided in a separate document that is unavailable to those who enrol participants or assign interventions	**NA**

For peer review only - http://bmjopen.bmj.com/site/about/guidelines.xhtml

Allocation concealment mechanism	16b	Mechanism of implementing the allocation sequence (eg, central telephone; sequentially numbered, opaque, sealed envelopes), describing any steps to conceal the sequence until interventions are assigned	**NA**
Implementation	16c	Who will generate the allocation sequence, who will enrol participants, and who will assign participants to interventions	**NA**
Blinding (masking)	17a	Who will be blinded after assignment to interventions (eg, trial participants, care providers, outcome assessors, data analysts), and how	**NA**
	17b	If blinded, circumstances under which unblinding is permissible, and procedure for revealing a participant's allocated intervention during the trial	**NA**
**Methods: Data collection, management, and analysis**			
Data collection methods	18a	Plans for assessment and collection of outcome, baseline, and other trial data, including any related processes to promote data quality (eg, duplicate measurements, training of assessors) and a description of study instruments (eg, questionnaires, laboratory tests) along with their reliability and validity, if known. Reference to where data collection forms can be found, if not in the protocol	**X**
	18b	Plans to promote participant retention and complete follow-up, including list of any outcome data to be collected for participants who discontinue or deviate from intervention protocols	**NA**
Data management	19	Plans for data entry, coding, security, and storage, including any related processes to promote data quality (eg, double data entry; range checks for data values). Reference to where details of data management procedures can be found, if not in the protocol	**X**
Statistical methods	20a	Statistical methods for analysing primary and secondary outcomes. Reference to where other details of the statistical analysis plan can be found, if not in the protocol	**X**
	20b	Methods for any additional analyses (eg, subgroup and adjusted analyses)	**X**
	20c	Definition of analysis population relating to protocol non-adherence (eg, as randomised analysis), and any statistical methods to handle missing data (eg, multiple imputation)	**X**
**Methods: Monitoring**			**NA**
Data monitoring	21a	Composition of data monitoring committee (DMC); summary of its role and reporting structure; statement of whether it is independent from the sponsor and competing interests; and reference to where further details about its charter can be found, if not in the protocol. Alternatively, an explanation of why a DMC is not needed	**NA**

	21b	Description of any interim analyses and stopping guidelines, including who will have access to these interim results and make the final decision to terminate the trial	**NA**
Harms	22	Plans for collecting, assessing, reporting, and managing solicited and spontaneously reported adverse events and other unintended effects of trial interventions or trial conduct	**NA**
Auditing	23	Frequency and procedures for auditing trial conduct, if any, and whether the process will be independent from investigators and the sponsor	**NA**
**Ethics and dissemination**			
Research ethics approval	24	Plans for seeking research ethics committee/institutional review board (REC/IRB) approval	**X**
Protocol amendments	25	Plans for communicating important protocol modifications (eg, changes to eligibility criteria, outcomes, analyses) to relevant parties (eg, investigators, REC/IRBs, trial participants, trial registries, journals, regulators)	**X**
Consent or assent	26a	Who will obtain informed consent or assent from potential trial participants or authorised surrogates, and how (see Item 32)	**X**
	26b	Additional consent provisions for collection and use of participant data and biological specimens in ancillary studies, if applicable	**NA**
Confidentiality	27	How personal information about potential and enrolled participants will be collected, shared, and maintained in order to protect confidentiality before, during, and after the trial	**X**
Declaration of interests	28	Financial and other competing interests for principal investigators for the overall trial and each study site	**X**
Access to data	29	Statement of who will have access to the final trial dataset, and disclosure of contractual agreements that limit such access for investigators	**X**
Ancillary and post-trial care	30	Provisions, if any, for ancillary and post-trial care, and for compensation to those who suffer harm from trial participation	**NA**
Dissemination policy	31a	Plans for investigators and sponsor to communicate trial results to participants, healthcare professionals, the public, and other relevant groups (eg, via publication, reporting in results databases, or other data sharing arrangements), including any publication restrictions	**X**
	31b	Authorship eligibility guidelines and any intended use of professional writers	**X**
	31c	Plans, if any, for granting public access to the full protocol, participant-level dataset, and statistical code	**NA**

For peer review only - http://bmjopen.bmj.com/site/about/guidelines.xhtml

Appendices

Informed consent materials	32	Model consent form and other related documentation given to participants and authorised surrogates	-
Biological specimens	33	Plans for collection, laboratory evaluation, and storage of biological specimens for genetic or molecular analysis in the current trial and for future use in ancillary studies, if applicable	**NA**

*It is strongly recommended that this checklist be read in conjunction with the SPIRIT 2013 Explanation & Elaboration for important clarification on the items. Amendments to the protocol should be tracked and dated. The SPIRIT checklist is copyrighted by the SPIRIT Group under the Creative Commons "Attribution-NonCommercial-NoDerivs 3.0 Unported" license.

For peer review only

BMJ Open

Central Sensitisation and Functioning in patients with Chronic Low Back Pain: Protocol for a cross-sectional and cohort study

Journal:	*BMJ Open*
Manuscript ID	bmjopen-2019-031592.R3
Article Type:	Protocol
Date Submitted by the Author:	13-Dec-2019

Complete List of Authors:	Ansuategui Echeita, Jone; University Medical Center Groningen, Rehabilitation Medicine<br>Schiphorst Preuper, Henrica ; University Medical Center Groningen, Rehabilitation Medicine<br>Dekker, Rienk; University Medical Center Groningen, Rehabilitation Medicine; University Medical Center Groningen, Center for Sports Medicine<br>Stuive, Ilse; University Medical Center Groningen , Rehabilitation Medicine<br>Timmerman, Hans; Radboud University Medical Center, Department of Anesthesiology, Pain and Palliative Medicine<br>Wolff, Andre; University Medical Center Groningen, Anesthesiology Pain Center<br>Reneman, Michiel; University Medical Center Groningen, Rehabilitation Medicine

**Primary Subject Heading**:	Rehabilitation medicine
Secondary Subject Heading:	Anaesthesia, Sports and exercise medicine, Occupational and environmental medicine
Keywords:	Hyperalgesia, Autonomic Nervous System, Lifting, Exercise Test, Physical Activity, Disability

SCHOLARONE™
Manuscripts

I, the Submitting Author has the right to grant and does grant on behalf of all authors of the Work (as defined in the below author licence), an exclusive licence and/or a non-exclusive licence for contributions from authors who are: i) UK Crown employees; ii) where BMJ has agreed a CC-BY licence shall apply, and/or iii) in accordance with the terms applicable for US Federal Government officers or employees acting as part of their official duties; on a worldwide, perpetual, irrevocable, royalty-free basis to BMJ Publishing Group Ltd ("BMJ") its licensees and where the relevant Journal is co-owned by BMJ to the co-owners of the Journal, to publish the Work in this journal and any other BMJ products and to exploit all rights, as set out in our licence.

The Submitting Author accepts and understands that any supply made under these terms is made by BMJ to the Submitting Author unless you are acting as an employee on behalf of your employer or a postgraduate student of an affiliated institution which is paying any applicable article publishing charge ("APC") for Open Access articles. Where the Submitting Author wishes to make the Work available on an Open Access basis (and intends to pay the relevant APC), the terms of reuse of such Open Access shall be governed by a Creative Commons licence – details of these licences and which Creative Commons licence will apply to this Work are set out in our licence referred to above.

Other than as permitted in any relevant BMJ Author's Self Archiving Policies, I confirm this Work has not been accepted for publication elsewhere, is not being considered for publication elsewhere and does not duplicate material already published. I confirm all authors consent to publication of this Work and authorise the granting of this licence.

For peer review only

Central Sensitisation and Functioning in patients with Chronic Low Back Pain: Protocol for a cross-sectional and cohort study

J. Ansuategui Echeita [1], H.R. Schiphorst Preuper [1], R. Dekker [1], I. Stuive [1], H. Timmerman [2, 3], A.P. Wolff [3], M.F. Reneman [1].

Affiliations:

[1] Department of Rehabilitation Medicine, University of Groningen, University Medical Center Groningen, Groningen, The Netherlands.

[2] Department of Anesthesiology, Pain and Palliative Medicine, Radboud university medical center, Nijmegen, The Netherlands.

[3] Department of Anesthesiology Pain Center, University of Groningen, University Medical Center Groningen, Groningen, The Netherlands.

Corresponding author: Jone Ansuategui Echeita, Department of Rehabilitation Medicine, University Medical Center Groningen, P.O. Box 30.002, 9750 RA Haren, The Netherlands. Email: j.ansuategui.echeita@umcg.nl

For peer review only

ABSTRACT

Introduction. A relevant subsample of patients with Chronic Low Back Pain (CLBP) have manifested augmented pain processing, Central Sensitisation (CS). Patients with CLBP have limited functioning and participation. Theoretically, physical functioning in patients with CLBP can plausibly be linked to CS; however, evidence to explain such association is scarce. Moreover, there is no gold standard for CS diagnosis. The objectives of the study are:

1) To analyse the association between instruments assessing reference symptoms and signs attributed to CS.
2) To analyse whether reference symptoms and signs attributed to CS are associated with functioning measurement outcomes.
3) To analyse whether changes (between baseline and discharge) in reference symptoms and signs attributed to CS are related to changes in each of the functioning measurement outcomes.

Methods and analysis. Cross-sectional and longitudinal observational study measuring at baseline and discharge of an interdisciplinary rehabilitation program. A sample size of 110 adult patients with CLBP has been calculated for the study. CS measurements: Central Sensitisation Inventory, Quantitative Sensory Testing and Heart-Rate Variability. Functioning measurements: Lifting capacity, Maximal aerobic capacity, Accelerometry, and Reported functioning. Statistical analyses: 1) correlation between CS measurements, 2) multiple regression between functioning (dependent variable) and CS measurements (independent variable), 3) multiple regression between changes in scores of functioning (dependent variable) and CS measurements (independent variable), and corrected for sex and age.

Ethics and dissemination. The study obtained the clearance to its implementation from the Medical Research Ethics Committee of the University Medical Center Groningen (METc UMCG) in July 2017. The results will be disseminated through scientific publications in peer-reviewed journals, presentations at relevant conferences, and reports to stakeholders.

Trial registration number. NTR7167 / NL6980.

KEYWORDS

Hyperalgesia, Autonomic Nervous System, Somatosensory, Lifting, Exercise Test, Physical Activity, Disability.

For peer review only

STRENGTHS AND LIMITATIONS OF THIS STUDY

- Novel and comprehensive approaches for the assessment of the presence of CS.
- Extensive amount of measurements from multiple domains for the determination of functioning status.
- Longitudinal study with measurements at baseline and discharge of an interdisciplinary rehabilitation program implemented as ‘Care as Usual’.
- Strategies allocated to promote participation and adherence, and to mitigate selection bias to the largest extent possible.
- Statistical analyses are performed with selections of CS and functioning measurements, and these selections may influence study results.

For peer review only

INTRODUCTION

Background

Twenty percent of the general population experiences prolonged or recurrent pain for at least three months, such pain is then considered to be chronic.[1,2] Low back pain is the principal cause of Years Lived with Disability (YLD) and work days lost in Europe and worldwide,[3] and its sequelae affect 9% of the global population.[4] Moreover, although there are several treatment intervention strategies available for patients with Chronic Low Back Pain (CLBP), treatment effect sizes have only been moderate.[5] Such evidence may explain why 40% of the patients are not satisfied with the treatment received.[2] The economic consequences of chronic pain include direct costs of medical care and pain management treatments, as well as indirect costs of decreased work productivity and sick leave, and of disability and sickness-retirement.[2,6] The indirect costs determine 86-88% of the total costs produced by patients with CLBP.[7,8] In particular, in the Netherlands, the costs derived from back pain to Dutch society were 3.5 billion in 2007, corresponding to 0.6% of the Gross National Product.[8]

In the past decennia, a relevant subsample of patients with chronic pain, including individuals with CLBP, have shown an increased responsiveness to noxious and non-noxious stimuli, described as Central Sensitization (CS).[9] CS is a result of an imbalance in the nociceptive pathways ("pain pathways") and supraspinal structures due to an amplified facilitation and/or reduced inhibition.[10–12] The phenomenon, CS, is manifested by an amplified pain perception regarding its intensity (hyperalgesia and allodynia), duration (aftersensations and temporal summation), and distribution (expansion of the receptive field), as well as a reduced conditioned pain modulation.[12] There is currently no gold standard for CS diagnosis. Identifying reference clinical symptoms and signs, however, may indicate the presence of CS: disproportionate pain, diffuse distribution, allodynia and hyperalgesia, hypersensitivity unrelated to mechanical stimulus but rather to environmental sensations (light, temperature, noise, or other stressors), maladaptive psychosocial factors, and low vagal nerve activity.[13–16] Instruments like Central Sensitization Inventory (CSI),[17] Quantitative Sensory Testing (QST) [18,19] and Heart-Rate Variability (HRV),[20] are non-invasive, feasible and assess different reference clinical symptoms and signs indicative of CS.[12] In the absence of a gold standard, these instruments comprehensively measure different indicators of CS.

Chronic pain is a multifactorial condition which has an impact on physical or body structures and functions, on psychological processes and on daily activities and quality of life of individuals.[2,21,22] Limited functioning or capacity to perform everyday tasks (essential for an independent living) affects individuals with chronic pain in their mobility and self-care, social relationships, work and leisure. [23] Individuals' functioning is composed of body functions (body level) –described by impairments they experience–and of activities and participation (personal and social levels, respectively) described by capacity and performance tests.[22,24] Lifting capacity, Physical Activity (PA)

For peer review only

levels and/or PA distribution or patterns, aerobic capacity, or daily activities participation, are measures that account for the multiple operationalizations of functioning and together provide an extensive overview of different domains of the functioning status.

Patients with CLBP are likely to be less physically active, and be more deconditioned and disabled.[3,25-27] A subsample of patients with CLBP can manifest symptoms and signs of CS being present. Because patients with CS are more reactive to stimuli and their pain experience is enhanced, the presence of CS and/or higher levels of CS could plausibly be associated with lower physical functioning and participation in patients with CLBP, and vice versa. More severe symptomatology of CS, decreased parasympathetic/vagal activity, or altered somatosensory responses have been associated with greater self-reported pain-related disability.[28-34] However, the associations between altered somatosensory responses and self-reported disability, were dependent on the site of testing, the stimuli, and the type of tests performed. Also, CS has been assessed with different instruments that measure specific CS clinical symptoms,[12] lacking a comprehensive evaluation of the mechanisms involved in CS. Moreover, functioning has most frequently been assessed with self-reported disability which is not exclusively representative of the functioning status of the patient.

The study described in this research protocol has been created to improve the assessment and management of pain and disability of centrally sensitised patients with CLBP and fill the existing gaps in knowledge. On the one hand, it intends to assist on the phenotyping of centrally sensitised patients with CLBP. To this purpose, an integrated assessment of CS combining the use of various instruments which measure different reference clinical symptoms of CS is aimed. On the other hand, it intends to improve the understanding of CS phenomenon and its contribution to functioning status, as determined by an extensive selection of capacity and performance tests and self-reported functioning measures.

Objective(s)

The objectives of the studies covered by this research protocol are:

1. To analyse the association between three instruments assessing reference symptoms and signs attributed to CS in patients with CLBP: CSI, QST and HRV.
2. To analyse whether reference symptoms and signs attributed to CS are associated with functioning measurement outcomes in patients with CLBP. Specifically, to analyse the association between the three instruments assessing CS (CSI, QST and HRV) and: Lifting capacity, Aerobic capacity, PA levels and/or PA distribution or patterns, and patient-reported measurements.
3. To analyse whether changes (between baseline and discharge) in reference symptoms and signs attributed to CS are related to changes in each of the functioning measurement outcomes in patients with CLBP. Specifically, to

For peer review only - http://bmjopen.bmj.com/site/about/guidelines.xhtml

analyse whether decreases in CS described as: a lower CSI score, an increase in the pain thresholds (QST), and an increase in parasympathetic/vagal tone (HRV), are related to a better functioning described as: an increase in lifting capacity, an increase in aerobic capacity, an increase in PA levels and/or a change in PA distribution or patterns, and better outcome scores in patient-reported measurements.

METHODS AND ANALYSES

Study design

An observational study project with both cross-sectional and longitudinal designs is being conducted from September 2017 to September 2019 in the Center for Rehabilitation of the University Medical Center Groningen (CvR – UMCG) in The Netherlands.

Patient and Public Involvement

Personnel of the CvR-UMCG collaborate in the study, in particular, the Pain rehabilitation team during patient management and data collection process and the Central exercise lab team on data collection process. The authors wish to express their gratitude to the collaborators and to the patients that participate.

Participants

Consecutive patients between 18 and 65 years of age who are primarily referred to the outpatient Pain Rehabilitation Department of the CvR - UMCG due to a chronic primary low back pain–even if patients have undergone surgery in the past[35] and who are admitted for the interdisciplinary pain rehabilitation program, are eligible to participate in the study. Additionally, patients are invited to the study if they are mentally competent and can follow instructions. Furthermore, patients are excluded from the study if, based on their file, they: have a specific diagnosis that would better account for the symptoms (e.g. cancer, osteoporosis, and/or spinal fractures), have neuralgia and/or radicular pain in the legs, have some major disorder or co-morbidity that can affect their physical and/or mental functioning (e.g. psychiatric disorders) or that can interfere with the study, are pregnant (or planning to be), have any specific contraindication according to the standards of practice guidelines of the assessments that they are going to be subjected to,[36,37] or take any medication that may interfere with the assessment outcomes such as calcium channel blockers on HRV. CS-related comorbidities (i.e. fibromyalgia, osteoarthritis, or chronic fatigue syndrome) are no reason for exclusion from the study.

For peer review only - http://bmjopen.bmj.com/site/about/guidelines.xhtml

Procedures

Measurements are performed as part of 'Care As Usual' (CAU) for outpatients following the Pain Rehabilitation guidelines of the CvR – UMCG. However, not all measurements of the research protocol belong to the battery of assessments of the CvR – UMCG, as pointed out in the legend of Figure 1.

During the first appointment of the patients with the rehabilitation physician, patients are invited to participate in the studies if they are eligible according to the criteria defined for the studies. Eligible patients who are invited to participate and receive information about the study, but have not reacted, are contacted in a week's time to remind them about the study participation. Those who sign the informed consent, fill in forms with their relevant personal information during the baseline assessment and are given vouchers for the travel expenses during the discharge assessment. Participating patients follow CAU trajectory with the Pain Rehabilitation team in terms of assessments, screening and implementation of the interdisciplinary rehabilitation program. As part of the research protocol, the performance of additional assessments and filling of questionnaires on CS and functioning are required. All CS, and objective monitoring and capacity tests are measured at two time points, baseline and discharge: the questionnaire CSI as CAU; the physical assessments lifting capacity and HRV as CAU, and aerobic capacity and QST as additional; and the monitoring of PA with an accelerometer as additional during the first and last week of the rehabilitation program. The pain intensity and functioning questionnaires are measured at 13 time points, baseline and once a week during the 12-week rehabilitation program: PDI as CAU at baseline but as additional during the rehabilitation program, and VAS Pain, WAS and Rand36-PF as additional. A diagram of the project procedure is presented as Figure 1.

(Insert Figure 1 around here)

Measurements

Central Sensitisation measurements

In order to comprehensively assess the symptoms and signs attributed to CS, data is obtained from a questionnaire (CSI) and two physical assessments (QST and HRV).

CSI. The inventory is a self-reported questionnaire with two sections to estimate the severity of CS-related symptoms. Section A is a 25-item questionnaire to assess the presence of the most common CS-related symptoms. Each of the questions is measured on a 5-point Likert scale from 0 (never) to 4 (always), and the total score ranges from 0 to 100, where higher scoring is associated with more severe symptomatology. Section B is a 10-item questionnaire to assess the

For peer review only - http://bmjopen.bmj.com/site/about/guidelines.xhtml

previously diagnosed CS Syndromes (CSS) and/or conditions related to CS. Higher amount of CSS is associated with higher chances of CS being present. This instrument has shown excellent test-retest reliability (ICC = 0.82-0.97) and internal consistency (Cronbach α = 0.87-0.91) in the general population and in patients with chronic pain,[17,38-40] as well as good concurrent validity (sensitivity = 81%, specificity = 75%) for CSS diagnosis within patients with chronic pain.[38] For the purposes of the study the Dutch translation of the CSI is used,[41] which has also shown excellent internal consistency (Cronbach α = 0.91) and test-retest reliability (ICC = 0.91 and 0.88) in controls and in patients with chronic musculoskeletal pain respectively.[38-40] as well as good concurrent validity (sensitivity = 81%, specificity = 75%) for CSS diagnosis within patients with chronic pain.[38] For the purposes of the study the Dutch translation of the CSI is used,[41] which has also shown excellent internal consistency (Cronbach α = 0.91) and test-retest reliability (ICC = 0.91 and 0.88) in controls and in patients with chronic musculoskeletal pain respectively.[42] The psychometric information of CSI in patients with CLBP is not known.

QST. A battery of standardised sensory tests that quantifies the function of the somatosensory system. QST tests the characteristics of various sensation and nociceptive processes as representative of afferent nerve fibres and central pathways performance. The whole assessment lasts for one hour, and it consists of six sensory tests, which are measured in seven different predefined body locations: one training location to familiarise the patient with the tests, the most painful location (as pointed out by the patient), and five control locations. See Supplementary material 1 for full description of locations. The standardised instructions of the sensory tests are read to the patient at each test performance. Of the tests, five consist of mechanical stimuli based on and performed following the QST examination of the German Research Network on Neuropathic Pain (DFNS) protocol,[18] and one for conditioned pain modulation following the Nijmegen-Aalborg Screening QST (NASQ) protocol.[19,43] Overall, the DFNS protocol has shown good test-retest (mean r = 0.86) and inter-observer reliability (mean r = 0.83) in patients with affections in the somatosensory system either with or without pain.[44] For the purpose of avoiding bias due to the order of the measurements and locations, a counterbalancing procedure is established, which accounts for the locations and the measurements to be made. This procedure provides three different assessment order groups, all of which begin with the measurements at the training location and end with the conditioned pain modulation test. An additional counterbalancing procedure has been made for allocating patients to these assessment order groups (either 1, 2, or 3) every nine patients a different allocation order is performed to ensure a similar amount of QST assessments per group. All assessors are trained in the QST protocol. The following sensory tests are performed:

- *The Dynamic Mechanical Allodynia (DMA)* to test the presence of abnormal stimuli-evoked pain is assessed with a soft brush (SENSElab™, Brush-05, Somedic AB, Hörby, Sweden) that is swiped 1-2 cm over the skin. This is performed on a single repetition and on a train of three repetitions (wind-up response). At each of the seven

For peer review only - http://bmjopen.bmj.com/site/about/guidelines.xhtml

locations (see Supplementary material 1) it is recorded whether patients feel the sweeping and its pain intensity with a Numeric Rating Scale (NRS) ranging from 0 (no pain) to 100 (most intense pain imaginable) for both the single swipe and the series.[45,46] The DMA (ratio) is calculated as: DMA = (NRS-3 / NRS-1). The DMA test according to the DFNS protocol has shown to have good test-retest (r = 0.87) and inter-observer reliability (r = 0.79).[44]

- *The Mechanical Pain Threshold (MPT)* to test the ability to perceive sharp, pricking, or stinging stimuli is assessed with a set of standardised weighted pinprick stimulators with seven different intensities (The PinPrick stimulator set, MRC Systems GmbH, Germany) and with a modified method of limits. Starting at the lowest intensity (8mN), it progressively increases until patients report the stimulus to change from touch to painful (sharp, pricking or stinging). The intensity of the pinprick that produces the first painful stimulus in mN is recorded in each of the seven locations (see Supplementary material 1).[46-49] The MPT test according to the DFNS protocol has shown to have good test-retest (r = 0.80) and inter-observer reliability (r = 0.80).[44]

- *The Wind-Up Ratio (WUR)* to test the Nervous System's painful stimuli temporal summation or responsiveness to repeated noxious stimuli. Such test is assessed with a suprathreshold pinprick stimulator of 256mN (the PinPrick stimulator set, MRC Systems GmbH, Germany), which is applied on a single repetition and on a train of ten repetitions in an area of 1cm^2 with intervals of one second (wind-up response). The pain intensity measured with a Numeric Rating Scale (NRS) ranging from 0 (no pain) to 100 (most intense pain imaginable) for both the single repetition and the series are recorded in each of the seven locations (see Supplementary material 1).[50,51] The WUR (ratio) is calculated as: WUR = (NRS-10 / NRS-1). The WUR test according to the DFNS protocol has shown to have poor test-retest (r = 0.67) and inter-observer reliability (r = 0.56).[44]

- *Mechanical Detection Threshold (MDT)* to test the ability to detect light touch, is assessed with a standardised set of von Frey filaments (OptiHair2-Set, Marstock Nervtest, Germany) with a modified method of limits.[52,53] Starting at the intensity of 16mN, the stimulus application progressively decreases until patients do not report any sensation. The intensity of the filament that is not perceived in mN is recorded in each of the seven locations (see Supplementary material 1).[54] The MDT test, according to the DFNS protocol, has shown to have good test-retest (r = 0.90) and inter-observer reliability (r = 0.89).[44]

- *Pressure Pain Threshold (PPT)* to test the ability to perceive pressure pain stimuli on muscles, is assessed with an electronic pressure algometer (Force Ten ™ FDX, Wagner Instruments, USA). With this tool pressure in the contact point (1cm^2 tip) is progressively but steadily increased until patients report to feel pressure change towards painful (burning, drilling or aching). The intensity in N when pressure becomes painful and its pain intensity with a Numeric Rating Scale (NRS) ranging from 0 (no pain) to 100 (most intense pain imaginable)

For peer review only - http://bmjopen.bmj.com/site/about/guidelines.xhtml

scale is recorded in each of the seven locations (see Supplementary material 1).[18] The PPT test according to the DFNS protocol has shown to have good test-retest (r = 0.88) and inter-observer reliability (r = 0.84).[44]

- *Conditioned Pain Modulation (CPM)* to test the Nervous System's descending pain processing. The CPM test methodology selected for this study follows the recommendations of the EFIC meeting (2013).[55] The pain modulation is assessed by means of PPT tests (Test Stimuli, TS) in a distant location from patients dominant hand (m. Deltoid and m. Quadriceps of the non-dominant side, see Supplementary material 1) before and after immersing the dominant hand in a bucket full with ice and cold water (Cold Conditioning Stimuli, cCS). First, PPT measurements at distant locations are made; then, patients immerse the dominant hand in the bucket for as long as they possibly can with a maximal duration of three minutes; finally, PPT measurements are repeated. The PPT results, intensity in N and pain intensity with a Numeric Rating Scale (NRS) ranging from 0 (no pain) to 100 (most intense pain imaginable) scale, in both locations and the time the hand is immersed are recorded. The CPM (%) is calculated as: CPM = [(PPTpost – PPTpre) / PPTpre] ∗ 100; where lower values indicate lower alteration on the nociceptive pathways.[19,43] The CPM test, as the combination of the PPT handheld algometer and cold stimuli, has shown to be one of the most reliable methods with a modest test-retest reliability (ICC = 0.49 and Coefficient of Variation, CV = 63.6%).[56]

HRV. The test provides an indication of the function and balance of the Autonomic Nervous System (ANS), which is responsible for the control of internal functions including self-regulation, adaptability and resilience capacities.[57] The ANS is susceptible to mental and emotional processes, which in turn can produce an imbalance in self-regulation (affecting among others HR) by an hyper-activation of the sympathetic tone and reduced parasympathetic/vagal tone.[15,17] HRV testing can monitor heart rhythms, or time and pattern variation on HR and inter-beat (RR) intervals, and can be used to assess the function of the ANS.[58,59] The HRV test is performed by assessors trained by the HeartMath institute, and the 5-minute protocol is executed to guarantee the comparison of the results as recommended by the Task Force for short-term recordings.[58] Patients are instructed to sit calmly and breathe normally, while they have an ear pulse sensor placed which is connected to a computer.[60] The following measures are collected and recorded with the emWave PC Software (emWave®, HeartMath Inc., Boulder Creek, California): mean HR in beats per minute (bpm), mean RR interval in ms; time domain measures in ms as Standard Deviation of Normal-to-Normal interval (SDNN) indicator for cyclic components responsible of RR interval variability, Root Mean Square of Successive Differences (RMSSD) for beat-to-beat variance in HR and parasympathetic/vagal tone; frequency domain measures in ms^2/Hz as Very Low Frequency (VLF, 0.0033-0.04 Hz) indicator for long term regulation and sympathetic tone, Low Frequency (LF, 0.04-0.15 Hz) for sympathetic and parasympathetic/vagal tones, and High Frequency (HF,

For peer review only

0.15-0.40 Hz) for parasympathetic/vagal tone related to breathing rate; and Normalised Coherence indicator for HRV stable or regular pattern over time in %, where higher scores mean more harmonic signal.

Physical Functioning measurements

In order to determine the functioning status of individuals with chronic pain in multiple domains, data is obtained from two physical assessments (lifting and aerobic capacity tests), an accelerometer (PA level), and a battery of reported functioning questionnaires.

Lifting capacity test. The Floor-to-waist lift capacity test is based on the WorkWell Functional Capacity Evaluation (FCE) protocol [61] and is performed to measure patients work functioning or capacity. Such FCE lift test has been shown to have high test–retest (ICC = 0.81) and inter-rater reliability (ICC = 0.76) in patients with CLBP.[62,63] Assessors are trained in the floor-to-waist lift capacity test procedure. Patients are given standardised instructions to repetitively lift a crate with weights from a shelf at waist height to the floor and back. The test begins with a weight that can easily be lifted, progressively the load is increased, and the test ends when the maximum capacity is reached. The final endpoint can be reached due to: cardiac endpoint (85% of maximum heart rate), biomechanical endpoint (unsafe increasing weight because of lack of load handling control), patient endpoint (patient decided to stop), and criterion endpoint (normal end of the test).[64] The maximal load lifted in kg is recorded along with the endpoint reason, and the patient-reported and the assessor-observed mechanical effort as measured with Borg's Category Ratio scale (CR-10).[65] Borg's CR-10 scale ranges from 0 (at rest) to 10 (very, very hard), and has demonstrated to be a valid visual observation of the effort level of patients during their performance of a lifting capacity test.[66]

Aerobic capacity test. The maximal aerobic capacity is assessed with a Cardiopulmonary Exercise Test (CPET). The test is performed with a cycle ergometer (Ergoselect 200p or Ergoselect 200k, Ergoline, Bitz, Germany) following a defined continuous ramp protocol. Before any cycling begins, patients are interviewed about their daily activities, and their resting heart rhythm and function are recorded with an electrocardiogram (ECG) (assy cam-14, GE medical systems, US). Patients then have an unloaded warm-up of three minutes at 60-70 rotations per minute (rpm) before the test begins. At the start of the test, an experienced exercise physiologist in discussion with a specialised physician or nurse determine the starting workload (25-100 W) and ramp (5-25 W increase per minute) depending on patients estimated fitness level. During the test patients are asked to maintain a constant cadence while the working load is progressively being increased. Also, they are continuously being verbally encouraged to obtain their maximum performance. The maximal performance is determined by various parameters: a temporary loss of strength and energy (= exhaustion), a plateau in the peak oxygen uptake (VO2max), a Respiratory Exchange Ratio (RER) higher than 1.15, and/or a Heart-Rate (HR) higher than 85% of the predicted maximal HR.[37] Overall, the test lasts for 8-12 minutes and throughout the whole assessment patients are monitored on their cardiac activity with an ECG, on their blood

For peer review only - http://bmjopen.bmj.com/site/about/guidelines.xhtml

pressure with a cuff algometer, and on their ventilatory gases on a breath-by-breath basis with a metabolic cart (JAEGER ® Vyntus ® CPX, Jaeger, Germany, Hoechberg). The VO2max in ml/min and per kilogram in ml/min/kg is collected as the mean of VO2 over the last 30 seconds of exercise. Along with VO2max, the following measures are collected: peak workload in watts, HR at end of test in beats per minute (bpm), Breathing Frequency (BF) at end of test in breaths per min, expiratory tidal volume (VT) in l, peak energy expenditure in Metabolic Equivalent of Task (MET), test duration in min, and the patient-reported and the assessor-observed mechanical effort as measured with Borg's Rating of Perceived Exertion (RPE) scale. Borg's RPE scale ranges from 6 (no exertion at all) to 20 (maximal exertion) [65] and has shown to be highly correlated with HR (r = 0.74) and blood lactate (r = 0.83),[67] demonstrating to be a reliable instrument to measure work intensity.

PA levels. Objective measurements of PA levels are obtained by means of an accelerometer (ActiGraph GT9X Link, ActiGraph, Pensacola, FL, USA). The accelerometer is a small and light device that monitors wearers' daily PA and energy expenditure. Participating patients wear the accelerometer attached at their hip level for one week continuously during walking hours, in order to ensure the minimum of 4-full-day recording as recommended by activity monitoring reviewers and experts.[68,69] Different algorithms are chosen from the software provided by the manufacturer, ActiLife6 (ActiGraph, Pensacola, FL, USA), to discriminate between low PAs and split patients in three activity intensity categories (sedentary, light, and moderate) [70] and to obtain patients' energy expenditure in kcal [71] and MET.[72] The daily and weekly time in hours and percentage of time spent in each of the activity intensity categories as well as the estimated energy expenditure in both kcal and MET are collected.

Reported Functioning. Data from this domain is obtained from four questionnaires sent to patients and filled by them via internet. All the questionnaires require 10-15 minutes to be filled in.

- *Physical functioning (Rand36 Physical Functioning subscale – Rand36-PF)*. The Rand36 is a health-related quality of life survey that measures the impact of health on patients' well-being and ability to function. Patients are asked to only answer the physical functioning (PF) subscale which looks at the limitations patients experience during their daily activities. Each of the 10 items belonging to the PF subscale have a 3-level response choices ranging from 0 (limited a lot) to 100 (not limited at all), the total average score ranges from 0 to 100 too; where a higher score is associated with a greater limitation in performing daily activities. For the purposes of the study, the Dutch translation of the Rand36 is used, which has shown good internal consistency (Cronbach α = 0.92) and test-retest reliability (r = 0.72-0.82) in the general population.[73,74]

- *Disability (Pain Disability Index – PDI)*. The index is a 7-item questionnaire which measures the interference of pain with the ability to function in different daily life activities: family/home responsibilities, recreation, social activity, occupation, sexual behaviour, self-care and life-support activity. Each item ranges from 0 (no

For peer review only - http://bmjopen.bmj.com/site/about/guidelines.xhtml

interference) to 10 (total interference), and the total score ranges from 0 to 70; where scoring higher is associated with higher interference in daily life activities. The PDI has shown modest test-retest reliability (r = 0.44) and high internal consistency (Cronbach α = 0.86) in patients with chronic pain.[75] For the purposes of the study the Dutch translation of the PDI is used, which has shown a good internal consistency (Cronbach α = 0.85) and test-retest reliability (ICC = 0.76) in patients with musculoskeletal pain.[76]

- *Work ability (Work Ability Score – WAS)*. The score is a single-item question which compares patient's current work ability compared with their lifetime best. The result is a score ranging from 0 (unable to work) to 10 (best working ability). The single-item question is part of the Work Ability Index (WAI), a questionnaire which measures work ability and has shown to be test-retest reliable (difference between test and re-test = -0.53) in construction workers.[77] The WAS has shown to be highly correlated to the 28 items of the WAI among women on a long-term sick leave (r = 0.87) [78] and among active workers (r = 0.63).[79]

Supplementary measurements

Demographic characteristics. Patients fill in a form with their details when the QST assessment takes place. Such include: age, sex, nationality, height, weight, pain characteristics (affected body area, duration and medication, if applicable), educational level and employment characteristics (work-status and physical work demands per Dictionary of Occupational Titles (DOT) [80]).

Pain-related questionnaires.

- *Pain intensity (Visual Analogue Scale – VAS Pain)*. The scale is a single-item question to assess the current pain intensity in adults. It is a straight line with 'no pain' (score = 0) at one end and 'pain as bad as it could be' (score = 100) at the other, in which patients are asked to mark the line at the level that represents their pain level. The score is the distance between the mark and the 'no pain' end, where scoring higher is associated with greater pain intensity. The VAS Pain has shown good validity (r = 0.62-0.91) and good test-retest reliability (literate r = 0.94 and illiterate r = 0.71) in patients with rheumatic pain conditions.[81] VAS Pain is sent to patients and filled in by them via internet along with the battery of functioning questionnaires (described above), i.e. at baseline and weekly during the interdisciplinary rehabilitation program.
- *Body response to a straining exercise (Pain Response Questionnaire – PRQ).*[82] The questionnaire assesses the pain intensity of the different body locations on an 11-point numeric scale (NRS) ranging from 0 (no pain) to 10 (worst pain imaginable), and the type of pain felt (muscle soreness, other, both or unknown). Additionally it controls for the use of medication and for the performance of any other strenuous physical activity. For the purposes of this study, the body locations measured are predefined to four and the

For peer review only - http://bmjopen.bmj.com/site/about/guidelines.xhtml

questionnaire is being filled in on three occasions: before the CPET, immediately afterwards and 24 hours later. PRQ is filled at the same moment when the aerobic capacity test is performed (i.e. during baseline and discharge assessments).

Psychological screening questionnaires. These questionnaires measure psychosocial features common to CS syndromes that have demonstrated to affect pain experience and functioning.[83] Patients fill in the questionnaires via internet that are sent per CAU procedures by the Pain Rehabilitation team. Such are:

- *Pain Catastrophizing Scale (PCS)*. The 13-item questionnaire measures in a 5-point Likert scale (0 - not at all, 4 - all the time) the degree to which patients have had catastrophic thoughts or feelings during their pain experience and accounts for three subscales or dimensions: rumination, magnification and helplessness.[84] For the purposes of the study the Dutch translation of the PCS is used,[85] which has shown good internal consistency (Cronbach α = 0.85-0.91) and test-retest reliability (r = 0.92) among healthy individuals (students) and patients with chronic low back pain.[86]
- *Injustice Experience Questionnaire (IEQ)*. The 12-item reported questionnaire assesses in a 5-point Likert scale from (0 – never, 4 - all the time) four different aspects of the perceived injustice due to injury: severity, blame, unfairness and irreparability. The questionnaire has shown to have good internal consistency (Cronbach α = 0.92) and test-retest reliability (ICC = 0.98) in a group of patients with fibromyalgia.[87] For the purposes of the study the Dutch translation of the IEQ is used, as translated by the Pain in Motion group.[88]
- *Brief Symptom Inventory (BSI)*. The inventory is a 53-item reported questionnaire which assesses patient's current level of psychological distress with a 5-point Likert scale questions (0 - not at all, 4 - extremely), the amount of symptoms present, and their severity.[89] BSI is composed of nine subscales: somatisation, obsession-compulsion, interpersonal sensitivity, depression, anxiety, hostility, phobic anxiety, paranoid ideation and psychoticism. For the purposes of the study the Dutch translation of the BSI is used, which has shown good internal consistency (Cronbach α = 0.71-0.87) and test-retest reliability (r = 0.71-0.89) among psychiatric out-patient and healthy individual groups.[90]

Sample size

Due to the limited amount of relevant previous or similar studies, the sample sizes for the several analyses described in this research protocol are estimated a priori with GPower (G*Power for Windows, Version 3.1.9.2), and are exploratory as a result.

For peer review only - http://bmjopen.bmj.com/site/about/guidelines.xhtml

The following sample size calculations, as well as the planned statistical analyses later described, will be performed with a proxy for each of the main variables – being three variables for CS and four for functioning. Additionally, due to the influence of patient's sex and age in the assessment outcomes, all analyses will include these variables for a moderator effect.

Sample size for objective 1: To analyse the association between instruments assessing reference symptoms and signs attributed to CS.

Correlation analyses will be performed for this objective. Because the correlation between the different CS measurements is expected to be medium (a correlation coefficient of 0.3, an α error probability of 0.05, and a power of 0.8), a minimum sample size of 84 is calculated.

Sample size for objective 2: To analyse whether reference symptoms and signs attributed to CS are associated with functioning measurement outcomes; and for objective 3: To analyse whether changes (between baseline and discharge) in reference symptoms and signs attributed to CS are related to changes in each of the functioning measurement outcomes.

Multiple regression analyses will be performed for objectives 2 and 3. Based on the associations between CSI and psychosocial and cognitive behavioural factors,[28,29] the effect size is hypothesised to be medium. Since minimally three CS measurements are tested with sex and age required as independent variables, and controlled for the confounding effect of other demographic and test-related variables, eight predictors are foreseen. Considering the factors mentioned above into account, the sample size calculation results in a minimum of 77 patients, calculated with an α error probability of 0.05 and a power of 0.8. Finally, patients measured at discharge could be about 30% less than at baseline due to either not following the interdisciplinary rehabilitation program or personal reasons; a sample size of 110 patients may be required in this scenario.

Analyses

Data records from the different tests and questionnaires will be collected and merged into one database. The database management will be done by the project team and handled according to the internal regulations of the UMCG. The analyses of the study will be performed with SPSS software version 22.0 (IBM Corp., NY).

Before any analysis, data will be prepared:

- Variables need to be calculated: BMI; DMA, WUR and CPM tests (see Methods section, Main measurements: CS); PDI, Rand36-PF, PCS, BSI and IEQ questionnaires.

- HRV: data will be pre-processed to correct for any artefacts that may be present at the beginning or at the end of the recording.
- QST: all measurements will be transformed into standard normal distribution (Z-transformation).
- To perform the analyses to respond objective 3, Delta scores are computed (difference in scores between baseline and discharge) for CS, functioning and those confounder variables which are also measured at the same time points as the main measurements.

Proxies of the main measurements were selected to perform analyses. CS variables are: the sum score of CSI part A, the MPT for QST, and the RMSSD for HRV. Functioning variables are: the maximal weight lifted for lifting capacity, the VO2max per kg for the maximal aerobic capacity, the daily VM counts for PA, and the average score of Rand36-PF for reported physical functioning.

The presence of missing data will be checked. If a variable has more than 10% of missing data cases, its distribution will be further examined. When the distribution of missing data is missing completely at random, analyses with pairwise deletions are planned. If data is not missing completely at random, other suitable solutions will be applied such as multiple imputation.[91] Regarding the presence of outliers, cases at 3 SD from the mean will be considered as outliers. Their influence will be measured using Cook's distance and leverage values. When the outliers are not statistically influential and/or there is rationale for the outlier, no further actions are planned. The descriptive statistics of the sample characteristics and main test results will be at this point calculated; numerical variables as mean and SD in normally distributed data and as median and interquartile range (IQR 25-75) in non-normally distributed data, and categorical variables as counts and percentage of each category level.

Statistical approach for objective 1: To analyse the association between instruments assessing reference symptoms and signs attributed to CS.

Multiple partial correlation analyses will be performed and corrected for potential confounders. CS measurements will be the main variables and sex and age will be covariates. Additional covariates may enter the partial correlations based on their correlation coefficient with CS instruments and if $p<0.01$. Results will be reported as correlation coefficients (Pearson's r and/or Spearman's rho) and p-values, with and without controlling for covariates. The significance level for all analyses is established at 0.01 level.

Statistical approach for objective 2: To analyse whether reference symptoms and signs attributed to CS are associated with functioning measurement outcomes.

First multiple correlation analyses, followed by multiple regression analyses with data obtained at baseline, will be performed. Each of the main outcomes from functioning measurements will be introduced in the models as dependent

For peer review only - http://bmjopen.bmj.com/site/about/guidelines.xhtml

variable, CS measurements as independent variables, and sex, age, and other demographic characteristics and/or test-related variables as potential confounders and/or moderators.

Statistical approach for objective 3: To analyse whether changes (between baseline and discharge) in reference symptoms and signs attributed to CS are related to changes in each of the functioning measurement outcomes.

Similar approach to objective 2 will be performed: first multiple correlation analyses will be performed with data obtained at baseline and discharge, followed by multiple correlation analyses using Delta scores of the data, and finally multiple regression analyses again using Delta scores. As in objective 2, each of the main outcomes from functioning measurements will be introduced in the models as dependent variable, CS measurements as independent variables, and sex, age and other demographic characteristics and/or test-related variables as potential confounders and/or moderators.

Confounders entering the final models of objectives 2 and 3 will be selected based on their partial correlation coefficient with CS and functioning, and they will be included if $p \leq 0.10$. All multiple regression models will have the assumptions checked. Results of objectives 2 and 3 will be reported as coefficient (b), confidence intervals (95% CI), p-values and explained variance, with and without controlling for confounders and/or moderators. The significance level of the correlation analyses is established at 0.01 level and at 0.05 level for multiple regression analyses.

DISCUSSION

Several patients with CLBP have shown common manifestations with other chronic pain conditions, which may evidence the presence of CS in these patients. Yet knowledge on CS, especially in patients with CLBP, and on its association with functioning is developing. This research protocol aims to bridge that gap of knowledge with a novel approach. In the first place, the study (objective 1) will describe the mechanisms of CS in patients with CLBP using instruments that assess reference symptoms of CS from different perspectives. Furthermore, the study (objectives 2 and 3) will also analyse longitudinally whether the presence of CS and changes of CS can be associated with changes in physical functioning performance, for which functioning will be assessed with several objective monitoring and capacity tests, and self-reported measurements.

Within the constraints of CAU and in the absence of a single gold standard instrument that can diagnose CS, in this research protocol multiple instruments have been selected to examine reference symptoms and signs attributed to CS. 1) The hypersensitivity unrelated to mechanical stimulus are assessed with CSI. A questionnaire designed to measure CS, as it has been shown to be associated to factors known to contribute to CS and is able to identify somatic and emotional symptoms related to CSS.[28,38,92] 2) The disproportionate pain, diffuse distribution, and allodynia and hyperalgesia, are measured with QST. The QST is a battery of non-invasive standardised tests that assists in the identification of

For peer review only - http://bmjopen.bmj.com/site/about/guidelines.xhtml

sensory and pain thresholds, the determination of pain intensity perception, and the assessment of the conditioned pain modulation. Its results can allow pain characterisation (phenotyping) and processing, and monitoring its progression.[93-97] 3) The low vagal nerve activity is examined with HRV. A non-invasive standardised measure that characterises the function of the ANS.[20] The somatic and emotional symptoms related to CS syndromes may generate an imbalance between a hyperactivation of the sympathetic tone and a reduction of the parasympathetic/vagal tone.[20,98,99] 4) Psychosocial factors are measured with psychological screening questionnaires for perceived injustice (IEQ), catastrophizing (PCS) and psychological distress (BSI). These psychosocial factors have shown a consistent effect on functioning and on symptom beliefs and pain behaviours, which are common in patients with chronic pain, including CS syndromes. Therefore, although not necessary for the clinical classification of CS, these factors are included in the study and potentially in the analyses. Some of the CS assessment instruments used in this study are more acknowledged than others in CS literature. CSI is widely used to measure CS since it was designed as a proxy for it; however, its internal consistency and reliability in patients with CLBP has not yet been made available. Also, evidence is currently piling up on the necessity of routinely implementing the elements of QST relevant to patients' conditions in clinical practice, given its ability to assess the somatosensory function,[97,100] even though evidence in patients with CLBP is limited. Finally, literature on HRV as an instrument that may reflect an imbalance of ANS in patients with chronic pain conditions (specifically in those patients that have shown an overlap in the CS symptoms) is scarce.[101] We are confident that the selected CS assessment instruments are considered the best available and combined will be able to comprehensively measure from the different reference clinical symptoms and signs indicative of CS being present. This will provide further insights on the mechanisms of CS and assist on the phenotyping of centrally sensitised patients with CLBP.

Various functioning assessments are selected for the research protocol to determine the functioning status of centrally sensitised patients with CLBP, including several objective monitoring and capacity tests, and self-reported measurements. 1) The floor-to-waist lifting capacity test is a physical assessment from the FCE that has demonstrated the best predictability for Return-to-Work in CLBP.[102] 2) The aerobic capacity, expressed as VO2max, can be best assessed with a maximal aerobic capacity test such as the CPET. The cycle ergometer and the treadmill are the most used assessments for aerobic capacity, but the cycle ergometer is most appropriate for patients.[37] 3) The PA is measured by an accelerometer, an easy wearable activity-monitoring device that provides objective measurements of PA levels and PA distribution or patterns. 4) Patient-reported functioning questionnaires show patients' pain experience, how they perceive or believe their functioning and participation is. The WAS, PDI and Rand36-PF show the advantage of being of short length and easy to interpret, due to their interval scale. Although questionnaires results can differ from their actual physical performance, both measurements complement each other. Furthermore, together

For peer review only - http://bmjopen.bmj.com/site/about/guidelines.xhtml

they may provide a more comprehensive overview of patients with CLBP.[103-105] Assessments such as lifting capacity and self-reported measurements have been routinely used to determine the functioning status of patients with CLBP. CPET, on the other hand, despite being scientifically superior to a submaximal aerobic capacity test, has barely been implemented with patients with CLBP. As a result, it is unknown whether a maximal CPET could be applied systematically with these patients and their pain response to it. Moreover, even if there is a large amount of literature on PA, the methodology implemented is very broad regarding population groups and/or measuring instruments, which hinders the synthesis of the understanding of PA of patients with CLBP. To bridge the gap, it may be of use an objective measurement of PA and monitored with accelerometers such as those of ActiGraph, which has shown to produce the most reliable devices for recording PA in free-living conditions.[106,107] It is believed that the assessments described are suitable and relevant for the purpose of measuring functioning from multiple domains; and that the extended knowledge and the better understanding of the mechanisms involved obtained is not limited to CLBP but can be extended to CS.

Data collected for this project is large and the measurements of CS and functioning collected involve a broad amount of variables. As a result, proxies will be used for the statistical analyses. Previous research in CS assessments, on the one hand, and in functioning assessments, on the other, have reported different methodologies and outcome parameters. Reason for which, even if authors have selected the proxy variables based on their relevance and contribution to the purposes of the current studies, the selection of the following measurements as proxies may not be beyond debate: for HRV the RMSSD, as it is a measurement of the parasympathetic/vagal tone which is not influenced by breathing;[108] for QST the MPT, since it measures the perceived skin mechanical hyperalgesia which is one of the main indicators of CS;[12] for the maximal aerobic capacity the VO2max corrected for the body mass of the individual, due to the effect of body mass on peak oxygen uptake; and for PA the daily VM counts, because it characterises movement duration and intensity.[109]

There are circumstances that might have influenced the results, in spite of the team's efforts to obtain the most extensive insights of CS and its association with functioning. To begin with, it should be considered that patients participating in the study are volunteers. Moreover, the exclusion criteria, despite being based on patients' files, are also dependent on doctor's judgement. The impact of these circumstances is so far unknown to the authors. Likewise, given the CAU design of the study the choice of measurements have to be feasible and compatible with the regular clinical procedures. As a consequence, the fMRI, a commonly mentioned instrument to measure CS, could not be performed in the study. The HRV on the other hand, although less often used, can be an insightful and innovative approach to the assessment of one of the indicators of CS. Additionally, the sample sizes are estimated a priori for the analyses due to insufficient previous studies and, therefore, they are of exploratory nature and used as a guide. Also, despite the efforts

For peer review only

of the team to promote the study participation and participant retention, it is likely that patient recruitment may be slower than anticipated and that patient may not have a complete follow-up. However, since the measurement period is ample, the calculations are vigilantly made with generous margins (see Methods section, Sample size), and the literature related to the topic has shown smaller sample sizes.[29,32,110] We remain positive about the sample size and expect to have more than enough participants to perform analyses with sufficient power. Also, for comparability purposes, measurements are planned to take place within three weeks from the Pain Rehabilitation team's baseline and discharge assessments. The CSI, PDI and psychological screening questionnaires during baseline assessment are sent to be filled six weeks before the rest of the measurements are obtained, a time distance due to waiting list time. Measurements are expected to be comparable to the rest of measurements at baseline, since no important changes are foreseen in patients regarding medication, surgery or personal conditions during this time, and questionnaires have shown to have good clinimetric properties. As opposed to the limitations presented, given the large amount of correlations in the multiple correlation analyses, a strict significance level was applied, i.e. 0.01. We are confident that the results obtained will provide meaningful and valuable information.

All things considered, the results obtained from the measurements described in this research protocol can, firstly, help to better describe and understand the mechanisms involved in CS, particularly the ones affecting the functioning of patients with CLBP; and secondly, be used to optimise the effectiveness of pain management interventions. To the authors' knowledge, the study described in this research protocol are the first of its kind and it may become an important breakthrough for researchers involved in the study of pain, for the treatment strategy of patients with chronic pain by healthcare professionals, and for patient's pain education.

ETHICS AND DISSEMINATION

All participating patients sign the informed consent before any assessment takes place. Formal ethical clearance to perform the study has been obtained from the METC University Medical Center Groningen (The Netherlands) in July 2017 (METC 2016/702). All procedures are in accordance with the ethical standards of the Helsinki Declaration of 1975 as revised in 2014 [111] and with Dutch regulations of the Medical Research Involving Human Subjects Act, in Dutch: Wet Medisch-Wetenschappelijk Onderzoek (WMO), amended in 2018.[112]

The dissemination strategy of the research study results will be through scientific publications in peer-reviewed journals and presentations in relevant national and international conferences. In addition to it, reports will be sent by post to participants, and reports and presentations in meetings will be provided to stakeholders.

For peer review only - http://bmjopen.bmj.com/site/about/guidelines.xhtml

ABBREVIATIONS

ANS	Autonomic Nervous System
BF	Breathing Frequency at the end of CPET
BMI	Body Mass Index
BSI	Brief Symptom Inventory
CAU	Care As Usual
cCS	cold Conditioning Stimulus
CLBP	Chronic Low Back Pain
CPET	Cardiopulmonary Exercise Test
CPM	Conditioned Pain Modulation
CS	Central Sensitisation
CSI	Central Sensitisation Inventory
CSS	Central Sensitisation Syndromes
CvR-UMCG	Center for Rehabilitation of the UMCG; in Dutch: Centrum voor Revalidatie
CR-10 scale	Category Ratio (0-10) scale
DMA	Dynamic Mechanical Allodynia
DFNS	German Research Network on Neuropathic Pain; in German: Deutschen Forschungsverbund Neuropathischer Schmerz
DOT	Dictionary of Occupational Titles
ECG	Electrocardiogram
FCE	Functional Capacity Evaluation
HF	High Frequency
HR	Heart Rate
HRV	Heart Rate Variability
IC	Informed Consent
ICC	Intra-Class Correlation
IEQ	Injustice Experience Questionnaire
LF	Low Frequency
MDT	Mechanical Detection Threshold
MET	Metabolic Equivalent of Task
METc	Medical Research Ethics committee; in Dutch: Medisch Ethische Toetsing commissie
MPT	Mechanical Pain Threshold
NASQ	Nijmegen-Aalborg Screening QST
NRS	Numeric Rating Scale
PA	Physical Activity
PCS	Pain Catastrophizing Scale
PDI	Pain Disability Index
PF	Physical Functioning
PPT	Pressure Pain Threshold
PRQ	Pain Response Questionnaire

For peer review only

QST	Quantitative Sensory Testing
Rand36-PF	Physical Functioning subscale of the Rand36 questionnaire
RER	Respiratory Exchange Ratio
RMSSD	Root Mean Square of Successive Differences between NN intervals
RPE	Rating of Perceived Exertion
RR interval	Inter-beat interval
SDNN	Standard Deviation of Normal-to-Normal (NN) intervals
TS	Test Stimulus
UMCG	Universitair Medisch Centrum Groningen
VAS	Visual Analogue Scale
VLF	Very Low Frequency
VO2max	Peak VO2, Peak oxygen uptake
VT	Expiratory Tidal Volume
WAI	Work Ability Index
WAS	Work Ability Score
WMO	Medical Research Involving Human Subjects Act; in Dutch: Wet Medisch-wetenschappelijk Onderzoek met Mensen
WUR	Wind-Up Ratio
YLD	Years Lived with Disability

For peer review only - http://bmjopen.bmj.com/site/about/guidelines.xhtml

STATEMENTS

Author's Contributions: JAE was responsible for the study concept and design, data collection, and manuscript preparation and edit; HRSP, RD, and MR were responsible for the study concept and design, and critical review of the manuscript; IS, HT and APW were responsible for the study design and critical review of the manuscript.

Competing interests: The authors declare that there are no conflicts of interest.

Funding: The Pain Rehabilitation Development Center (in Dutch: Ontwikkelcentrum PijnRevalidatie (OPR)) of the CvR-UMCG funded the research study.

Trial registration: NTR7167/NL6980, April 16th 2018; retrospectively registered.

Ethics approval: The Medical Research Ethics Committee of the University Medical Center Groningen (METc UMCG) provided a positive reaction in July 2017 to the development of the research study "*Central Sensitization and Functioning in patients with Chronic Low Back Pain*" (METc 2016/702). Its first amendment was approved in October 2017 and its second amendment in August 2018.

For peer review only

REFERENCES

1. The Joint Task Force (JTF). ICD-11 Beta Draft - Mortality and Morbidity Statistics[Internet]. Available from: https://icd.who.int/browse11/l-m/en#/ (Accessed 24 May 2018).
2. Breivik H, Collett B, Ventafridda V, et al. Survey of chronic pain in Europe: Prevalence, impact on daily life, and treatment. Eur J Pain. 2006;10(4):287–333.
3. Hoy D, March L, Brooks P, et al. The global burden of low back pain: estimates from the Global Burden of Disease 2010 study. Ann Rheum Dis. 2014;73(6):968–74.
4. Vos T, Flaxman AD, Naghavi M, et al. Years lived with disability (YLDs) for 1160 sequelae of 289 diseases and injuries 1990–2010: A systematic analysis for the Global Burden of Disease Study 2010. Lancet. 2012;380(9859):2163–96.
5. Foster NE, Anema JR, Cherkin D, et al. Prevention and treatment of low back pain: evidence, challenges, and promising directions. Lancet. 2018;391(10137):2368–83.
6. Boonen A, van den Heuvel R, van Tubergen A, et al. Large differences in cost of illness and wellbeing between patients with fibromyalgia, chronic low back pain, or ankylosing spondylitis. Ann Rheum Dis. 2005;64:396–402.
7. Dagenais S, Caro J, Haldeman S. A systematic review of low back pain cost of illness studies in the United States and internationally. Spine J. 2008;8(1):8–20.
8. Lambeek LC, van Tulder MW, Swinkels ICS, et al. The Trend in Total Cost of Back Pain in the Netherlands in the Period 2002 to 2007. Spine. 2011;36(13):1050–8.
9. Giesecke T, Gracely RH, Grant MAB, et al. Evidence of augmented central pain processing in idiopathic chronic low back pain. Arthritis Rheum. 2004;50(2):613–23.
10. Woolf CJ. Central sensitization: implications for the diagnosis and treatment of pain. Pain. 2011;152(3 Suppl.):S2–15.
11. Arendt-Nielsen L, Morlion B, Perrot S, et al. Assessment and manifestation of central sensitisation across different chronic pain conditions. Eur J Pain. 2018;22(2):216–41.
12. den Boer C, Dries L, Terluin B, et al. Central sensitization in chronic pain and medically unexplained symptom research: A systematic review of definitions, operationalizations and measurement instruments. J Psychosom Res. 2019;117:32–40.
13. Nijs J, Torres-Cueco R, van Wilgen P, et al. Applying Modern Pain Neuroscience in Clinical Practice: Criteria for the Classification of Central Sensitization Pain. Pain Physician. 2014;17(5):447–57.

For peer review only

14. Smart KM, Blake C, Staines A, et al. The Discriminative Validity of "Nociceptive," "Peripheral Neuropathic," and "Central Sensitization" as Mechanisms-based Classifications of Musculoskeletal Pain. Clin J Pain. 2011;27(8):655–63.

15. De Couck M, Nijs J, Gidron Y. You May Need a Nerve to Treat Pain: The Neurobiological Rationale for Vagal Nerve Activation in Pain Management. Clin J Pain. 2014 ;30(12):1099–105.

16. Smart KM, Blake C, Staines A, et al. Mechanisms-based classifications of musculoskeletal pain: Part 1 of 3: Symptoms and signs of central sensitisation in patients with low back (±leg) pain. Man Ther. 2012; 17(4), 336–44.

17. Mayer TG, Neblett R, Cohen H, et al. The development and psychometric validation of the central sensitization inventory. Pain Pract. 2012;12(4):276–85.

18. Rolke R, Baron R, Maier C, et al. Quantitative sensory testing in the German Research Network on Neuropathic Pain (DFNS): Standardized protocol and reference values. Pain. 2006;123(3):231–43.

19. Wilder-Smith O. A paradigm-shift in pain medicine: implementing a systematic approach to altered pain processing in everyday clinical practice based on quantitative sensory testing. Center for Sensory-Motor Interaction (SMI), Department of Health Science and Technology, Aalborg University. 2013:76p.

20. McCraty R, Atkinson M, Tomasino D, et al. The coherent heart: heartbrain interactions, psychophysiological coherence, and the emergence of system-wide order. Boulder Creek, CA: Institute of Heartmath; 2009.

21. O'Brien T, Breivik H. The impact of chronic pain — European patients' perspective over 12 months. Scand J Pain. 2012;3(1):23–9.

22. Nugraha B, Gutenbrunner C, Barke A, et al. The IASP classification of chronic pain for ICD-11. Pain. 2019;160(1):88–94.

23. Cieza A, Stucki G, Weigl M, et al. ICF Core Sets for low back pain. J Rehabil Med. 2004; Suppl. 44:69–74.

24. Schneidert M, Hurst R, Miller J, et al. The role of Environment in the International Classification of Functioning, Disability and Health (ICF). Disabil Rehabil. 2003;25(11–12):588–95.

25. Smeets RJEM, Wittink H, Hidding A, et al. Do patients with chronic low back pain have a lower level of aerobic fitness than healthy controls?: are pain, disability, fear of injury, working status, or level of leisure time activity associated with the difference in aerobic fitness level?. Spine. 2006;31(1):90-7.

26. Ryan CG, Grant PM, Dall PM, et al. Individuals with chronic low back pain have a lower level, and an altered pattern, of physical activity compared with matched controls: An observational study. Aust J Physiother. 2009;55(1):53–8.

For peer review only

27. Duque I, Parra JH, Duvallet A. Maximal aerobic power in patients with chronic low back pain: a comparison with healthy subjects. European spine journal. 2011;20(1):87-93.

28. Kregel J, Schumacher C, Dolphens M, et al. Convergent validity of the dutch central sensitization inventory: associations with psychophysical pain measures, quality of life, disability, and pain cognitions in patients with chronic spinal pain. Pain Practice. 2018;18(6):777-87.

29. Huysmans E, Ickmans K, Van Dyck D, et al. Association Between Symptoms of Central Sensitization and Cognitive Behavioral Factors in People With Chronic Nonspecific Low Back Pain: A Cross-sectional Study. J Manipulative Physiol Ther. 2018;41(2):92-101.

30. Tanaka K, Murata S, Nishigami T, et al. The Central Sensitization Inventory predict pain-related disability for musculoskeletal disorders in the primary care setting. Eur J Pain. 2019; 23(9):1640-8.

31. Cohen H, Neumann L, Shore M, et al. Buskila. Autonomic dysfunction in patients with fibromyalgia: application of power spectral analysis of heart rate variability. Semin Arthritis Rheum. 2000;29(4):217-27.

32. Gockel M, Lindholm H, Niemistö L, et al. Perceived disability but not pain is connected with autonomic nervous function among patients with chronic low back pain. J Rehabil Med. 2008;40(5):355-8.

33. Hübscher M, Moloney N, Leaver A, et al. Relationship between quantitative sensory testing and pain or disability in people with spinal pain—A systematic review and meta-analysis. Pain. 2013; 154(9), 1497–504.

34. Georgopoulos V, Akin-Akinyosoye K, Zhang W, et al. Quantitative sensory testing and predicting outcomes for musculoskeletal pain, disability, and negative affect. Pain. 2019;160(9):1920-32.

35. IASP Taskforce for the Classification of Chronic Pain. The IASP classification of chronic pain for ICD-11: chronic primary pain. Pain. 2019; 160(1): 28-37.

36. Hart DL, Isernhagen SJ, Matheson LN. Guidelines for Functional Capacity Evaluation of People With Medical Conditions. J Orthop Sport Phys Ther. 1993;18(6):682–6.

37. American Thoracic Society, American College of Chest Physicians. ATS/ACCP Statement on Cardiopulmonary Exercise Testing. Am J Respir Crit Care Med. 2003;167(2):211–77.

38. Neblett R, Cohen H, Choi Y, et al. The Central Sensitization Inventory (CSI): establishing clinically significant values for identifying central sensitivity syndromes in an outpatient chronic pain sample. J Pain. 2013;14(5):438–45.

39. Neblett R, Hartzell MM, Mayer TG, et al. Establishing Clinically Relevant Severity Levels for the Central Sensitization Inventory. Pain Pract. 2017;17(2):166–75.

40. Scerbo T, Colasurdo J, Dunn S, et al. Measurement Properties of the Central Sensitization Inventory: A

For peer review only

Systematic Review. Pain Pract. 2018;18(4):544–54.

41. van Wilgen CP, Meeus M, Descheemaeker F, et al. Central Sensitization Inventory – Nederlandse consensusvertaling [Internet]. 2013. Available from: http://www.paininmotion.be/storage/app/media//materials/CSI-consensusvertaling.pdf (Accessed 19 Jul 2018).
42. Kregel J, Vuijk PJ, Descheemaeker F, et al. The Dutch Central Sensitization Inventory (CSI). Clin J Pain. 2016;32(7):624–30.
43. Timmerman H, Wilder-Smith O, van Weel C, et al. Detecting the neuropathic pain component in the clinical setting: a study protocol for validation of screening instruments for the presence of a neuropathic pain component. BMC Neurol. 2014;14:94–102.
44. Geber C, Klein T, Azad S, et al. Test–retest and interobserver reliability of quantitative sensory testing according to the protocol of the German Research Network on Neuropathic Pain (DFNS): A multi-centre study. Pain. 2011;152(3):548–56.
45. LaMotte RH, Shain CN, Simone DA, et al. Neurogenic hyperalgesia: psychophysical studies of underlying mechanisms. J Neurophysiol. 1991;66(1):190–211.
46. Ziegler EA, Magerl W, Meyer RA, et al. Secondary hyperalgesia to punctate mechanical stimuli. Brain. 1999;122(12):2245–57.
47. Chan AW, MacFarlane IA, Bowsher D, et al. Weighted needle pinprick sensory thresholds: a simple test of sensory function in diabetic peripheral neuropathy. J Neurol Neurosurg Psychiatry. 1992;55(1):56–9.
48. Greenspan JD, McGillis SLB. Thresholds for the Perception of Pressure, Sharpness, and Mechanically Evoked Cutaneous Pain: Effects of Laterality and Repeated Testing. Somatosens Mot Res. 1994;11(4):311–7.
49. Hampf G, Bowsher D, Wells C, et al. Sensory and autonomic measurements in idiopathic trigeminal neuralgia before and after radiofrequency thermocoagulation: differentiation from some other causes of facial pain. Pain. 1990;40(3):241–8.
50. Price DD, Hu JW, Dubner R, et al. Peripheral suppression of first pain and central summation of second pain evoked by noxious heat pulses. Pain. 1977;3(1):57–68.
51. Magerl W, Wilk SH, Treede R-D. Secondary hyperalgesia and perceptual wind-up following intradermal injection of capsaicin in humans. Pain. 1998;74(2–3):257–68.
52. Weinstein S. Intensive and extensive aspects of tactile sensitivity as a function of body part, sex and laterality. In: Kenshalo DR, ed. The skin senses: Proceedings. Springfield, IL, US: Charles C Thomas. 1968:195–222.

For peer review only

53. Fruhstorfer H, Gross W, Selbmann O. von Frey hairs: new materials for a new design. Eur J Pain. 2001;5(3):341–2.

54. Baumgärtner U, Magerl W, Klein T, et al. Neurogenic hyperalgesia versus painful hypoalgesia: two distinct mechanisms of neuropathic pain. Pain. 2002;96(1–2):141–51.

55. Yarnitsky D, Bouhassira D, Drewes AM, et al. Recommendations on practice of conditioned pain modulation (CPM) testing. Eur J Pain. 2015;19(6):805–6.

56. Imai Y, Petersen KK, Mørch CD, et al. Comparing test–retest reliability and magnitude of conditioned pain modulation using different combinations of test and conditioning stimuli. Somatosens Mot Res. 2016;33(3–4):169–77.

57. McCorry LK. Physiology of the autonomic nervous system. Am J Pharm Educ. 2007;71(4):78–88.

58. Task Force of the European Society of Cardiology and the North American Society of Pacing and Electrophysiology. Heart rate variability, standards of measurement, physiological interpretation, and clinical use. Circulation. 1996;93(5):1043–65.

59. McCraty R, Shaffer F. Heart Rate Variability: New Perspectives on Physiological Mechanisms, Assessment of Self-regulatory Capacity, and Health Risk. Glob Adv Heal Med. 2015;4(1):46–61.

60. HeartMath Inc. emWave Pro Plus Assessments [Internet]. 2018. Available from: https://heartcloud.com/library/asmts.html (Accessed 27 Feb 2019).

61. Isernhagen SJ, Hart DL, Matheson LM. Reliability of independent observer judgments of level of lift effort in a kinesiophysical Functional Capacity Evaluation. Work. 1999;12(2):145–50.

62. de Baets S, Calders P, Schalley N, et al. Updating the Evidence on Functional Capacity Evaluation Methods: A Systematic Review. J Occup Rehabil. 2018;28(3):418–28.

63. Brouwer S, Reneman MF, Dijkstra PU, et al. Test–Retest Reliability of the Isernhagen Work Systems Functional Capacity Evaluation in Patients with Chronic Low Back Pain. J Occup Rehabil. 2003;13(4):207–18.

64. Soer R, van der Schans CP, Geertzen JH, et al. Normative Values for a Functional Capacity Evaluation. Arch Phys Med Rehabil. 2009;90(10):1785–94.

65. Borg G. Borg's perceived exertion and pain scales.. Champaign, IL, US: Human Kinetics; 1998.

66. Reneman MF, Fokkens AS, Dijkstra PU, et al. Testing Lifting Capacity: Validity of Determining Effort Level by Means of Observation. Spine. 2005;30(2):e40–6.

67. Scherr J, Wolfarth B, Christle JW, et al. Associations between Borg's rating of perceived exertion and

For peer review only

physiological measures of exercise intensity. Eur J Appl Physiol. 2013;113(1):147–55.

68. Trost SG, McIver KL, Pate RR. Conducting Accelerometer-Based Activity Assessments in Field-Based Research. Med Sci Sports Exerc. 2005;37(11 Suppl.):S531–43.

69. Migueles JH, Cadenas-Sanchez C, Ekelund U, et al. Accelerometer Data Collection and Processing Criteria to Assess Physical Activity and Other Outcomes: A Systematic Review and Practical Considerations. Sport Med. 2017;47(9):1821–45.

70. Keadle SK, Shiroma EJ, Freedson PS, et al. Impact of accelerometer data processing decisions on the sample size, wear time and physical activity level of a large cohort study. BMC Public Health. 2014;14(1):1210.

71. Sasaki JE, John D, Freedson PS. Validation and comparison of ActiGraph activity monitors. J Sci Med Sport. 2011;14(5):411–6.

72. Crouter SE, Kuffel E, Haas JD, et al. Refined two-regression model for the ActiGraph accelerometer. Med Sci Sports Exerc. 2010;42(5):1029–37.

73. van der Zee KI, Sanderman R. RAND-36. umcg.nl [Internet]. Available from: https://www.umcg.nl/SiteCollectionDocuments/research/institutes/SHARE/assessment tools/handleiding_rand36_2e_druk.pdf (Accessed 28 Jun 2018).

74. van der Zee KI, Sanderman R, Heyink JW, et al. Psychometric qualities of the rand 36-item health survey 1.0: A multidimensional measure of general health status. Int J Behav Med. 1996;3(2):104–22.

75. Tait RC, Chibnall JT, Krause S. The Pain Disability Index: psychometric properties. Pain. 1990;40(2):171–82.

76. Soer R, Köke AJA, Vroomen PCAJ, et al. Extensive Validation of the Pain Disability Index in 3 Groups of Patients With Musculoskeletal Pain. Spine. 2013;38(9):e562–8.

77. de Zwart BCH, Frings-Dresen MHW, van Duivenbooden JC. Test-retest reliability of the Work Ability Index questionnaire. Occup Med. 2002;52(4):177–81.

78. Ahlstrom L, Grimby-Ekman A, Hagberg M, et al. The work ability index and single-item question: associations with sick leave, symptoms, and health – A prospective study of women on long-term sick leave. Scand J Work Environ Heal. 2010;36(5):404–12.

79. El Fassi M, Bocquet V, Majery N, et al. Work ability assessment in a worker population: comparison and determinants of Work Ability Index and Work Ability score. BMC Public Health. 2013;13:305–14.

80. U.S. Department of Labor, Employment and Training Administration. Dictionary of Occupational Titles. 4th ed. Washington; 1991.

For peer review only

81. Hawker GA, Mian S, Kendzerska T, et al. Measures of adult pain: Visual Analog Scale for Pain (VAS Pain), Numeric Rating Scale for Pain (NRS Pain), McGill Pain Questionnaire (MPQ), Short-Form McGill Pain Questionnaire (SF-MPQ), Chronic Pain Grade Scale (CPGS), Short Form-36 Bodily Pain Scale (SF36 BPS), and Measure of Intermittent and Constant Osteoarthritis Pain (ICOAP). Arthritis Care Res. 2011;63(S11):S240–52.

82. Soer R, Groothoff JW, Geertzen JHB, et al. Pain Response of Healthy Workers Following a Functional Capacity Evaluation and Implications for Clinical Interpretation. J Occup Rehabil. 2008;18(3):290–8.

83. Adams LM, Turk DC. Psychosocial Factors and Central Sensitivity Syndromes. Curr Rheumatol Rev. 2015 ; 11(2): 96–108.

84. Sullivan MJL, Bishop SR, Pivik J. The pain catastrophizing scale: development and validation. Psychol Assess. 1995;7(4):524–32.

85. Crombez G, Vlaeyen JWS. Pain Catastrophizing Scale (PCS) - Dutch Version (PCS-DV) [Internet]. 1996. Available from: https://meetinstrumentenzorg.blob.core.windows.net/test-documents/Instrument197/197_3_N.pdf (Accessed 8 Aug 2018).

86. van Damme S, Crombez G, Vlaeyen JWS, et al. De Pain Catastrophizing Scale: Psychometrische karakteristieken en normering. Gedragstherapie. 2000;33:209–20.

87. Rodero B, Luciano J V, Montero-Marín J, et al. Perceived injustice in fibromyalgia: Psychometric characteristics of the Injustice Experience Questionnaire and relationship with pain catastrophising and pain acceptance. J Psychosom Res. 2012;73(2):86–91.

88. van Wilgen CP, Nijs J, Don S, et al. The Injustice Experience Questionnaire: Nederlandstalige consensusvertaling [Internet]. 2014. Available from: http://www.paininmotion.be/storage/app/media//materials/IEQ-Dutch.pdf (Accessed 19 Jul 2018).

89. Derogatis LR. BSI brief symptom inventory: Administration, Scoring, and Procedure Manual (4th Ed.). Minneapolis, MN: National Computer Systems;1993.

90. de Beurs E, Zitman FG. The Brief Symptom Inventory (BSI): Reliability and validity of a practical alternative to SCL-90. Maandbl Geestelijke Volksgezond. 2006;61:120–41.

91. van Ginkel JR, Linting M, Rippe RC, et al. Rebutting Existing Misconceptions About Multiple Imputation as a Method for Handling Missing Data. J Pers Assess. 2019; 1-12.

92. van Wilgen CP, Vuijk PJ, Kregel J, et al. Psychological Distress and Widespread Pain Contribute to the Variance of the Central Sensitization Inventory: A Cross-Sectional Study in Patients with Chronic Pain. Pain

For peer review only

Pract. 2018;18(2):239–46.

93. Arendt-Nielsen L, Yarnitsky D. Experimental and Clinical Applications of Quantitative Sensory Testing Applied to Skin, Muscles and Viscera. J Pain. 2009;10(6):556–72.

94. Backonja M-M, Walk D, Edwards RR, et al. Quantitative Sensory Testing in Measurement of Neuropathic Pain Phenomena and Other Sensory Abnormalities. Clin J Pain. 2009;25(7):641–7.

95. Pavlaković G, Petzke F. The Role of Quantitative Sensory Testing in the Evaluation of Musculoskeletal Pain Conditions. Curr Rheumatol Rep. 2010;12(6):455–61.

96. Krumova EK, Geber C, Westermann A, et al. Neuropathic Pain: Is Quantitative Sensory Testing Helpful? Curr Diab Rep. 2012;12(4):393–402.

97. Uddin Z, MacDermid JC. Quantitative Sensory Testing in Chronic Musculoskeletal Pain. Pain Med. 2016;17(9):1694–703.

98. Martínez-Martínez L-A, Mora T, Vargas A, et al. Sympathetic Nervous System Dysfunction in Fibromyalgia, Chronic Fatigue Syndrome, Irritable Bowel Syndrome, and Interstitial Cystitis. JCR J Clin Rheumatol. 2014;20(3):146–50.

99. Tracy LM, Ioannou L, Baker KS, et al. Meta-analytic evidence for decreased heart rate variability in chronic pain implicating parasympathetic nervous system dysregulation. Pain. 2016;157(1):7–29.

100. Starkweather AR, Heineman A, Storey S, et al. Methods to measure peripheral and central sensitization using quantitative sensory testing: A focus on individuals with low back pain. Appl Nurs Res. 2016;29:237–41.

101. Koenig J, Falvay D, Clamor A, et al. Pneumogastric (Vagus) Nerve Activity Indexed by Heart Rate Variability in Chronic Pain Patients Compared to Healthy Controls: A Systematic Review and Meta-Analysis. Pain Physician. 2016;19(1):E55-78.

102. Kuijer PPFM, Gouttebarge V, Brouwer S, et al. Are performance-based measures predictive of work participation in patients with musculoskeletal disorders? A systematic review. Int Arch Occup Environ Health. 2012;85(2):109–23.

103. Reneman MF, Jorritsma W, Schellekens JMH, et al. Concurrent validity of questionnaire and performance-based disability measurements in patients with chronic nonspecific low back pain. J Occup Rehabil. 2002;12(3):119–29.

104. Brouwer S, Dijkstra PU, Stewart RE, et al. Comparing self-report, clinical examination and functional testing in the assessment of work-related limitations in patients with chronic low back pain. Disabil Rehabil. 2005;27(17):999–1005.

For peer review only

105. Caporaso F, Pulkovski N, Sprott H, et al. How well do observed functional limitations explain the variance in Roland Morris scores in patients with chronic non-specific low back pain undergoing physiotherapy? Eur Spine J. 2012;21(Suppl 2):S187–95.

106. Vanhelst J, Gottrand F, Baquet G, et al. Comparative Interinstrument Reliability of Uniaxial and Triaxial Accelerometers in Free-Living Conditions. Percept Mot Skills. 2012;114(2):584–94.

107. Aadland E, Ylvisåker E. Reliability of the Actigraph GT3X+ Accelerometer in Adults under Free-Living Conditions. PLoS One. 2015;10(8):e0134606.

108. Laborde S, Mosley E, Thayer JF. Heart Rate Variability and Cardiac Vagal Tone in Psychophysiological Research – Recommendations for Experiment Planning, Data Analysis, and Data Reporting. Front Psychol. 2017;08:213.

109. Matthews CE, Hagströmer M, Pober DM, et al. Best practices for using physical activity monitors in population-based research. Med Sci Sports Exerc. 2012;44(Suppl 1):S68-76.

110. Clauw DJ, Williams D, Lauerman W, et al. Pain Sensitivity as a Correlate of Clinical Status in Individuals With Chronic Low Back Pain. Spine. 1999; 24(19), 2035-41.

111. General Assembly of the World Medical Association. World Medical Association Declaration of Helsinki: Ethical principles for medical research involving human subjects. J Am Coll Dent. 2014;81(3):14–8.

112. Dutch Government. The Medical Research Involving Human Subjects Act, in Dutch: Wet medisch-wetenschappelijk onderzoek met mensen (WMO) [Internet]. Available from: https://wetten.overheid.nl/BWBR0009408/2018-08-01 (Accessed 28 Feb 2019).

For peer review only

FIGURE LEGENDS

Figure 1. Flow-chart of the project procedure.

* , measurements obtained which do not belong to CAU of the Pain Rehabilitation of the CvR – UMCG .

For peer review only

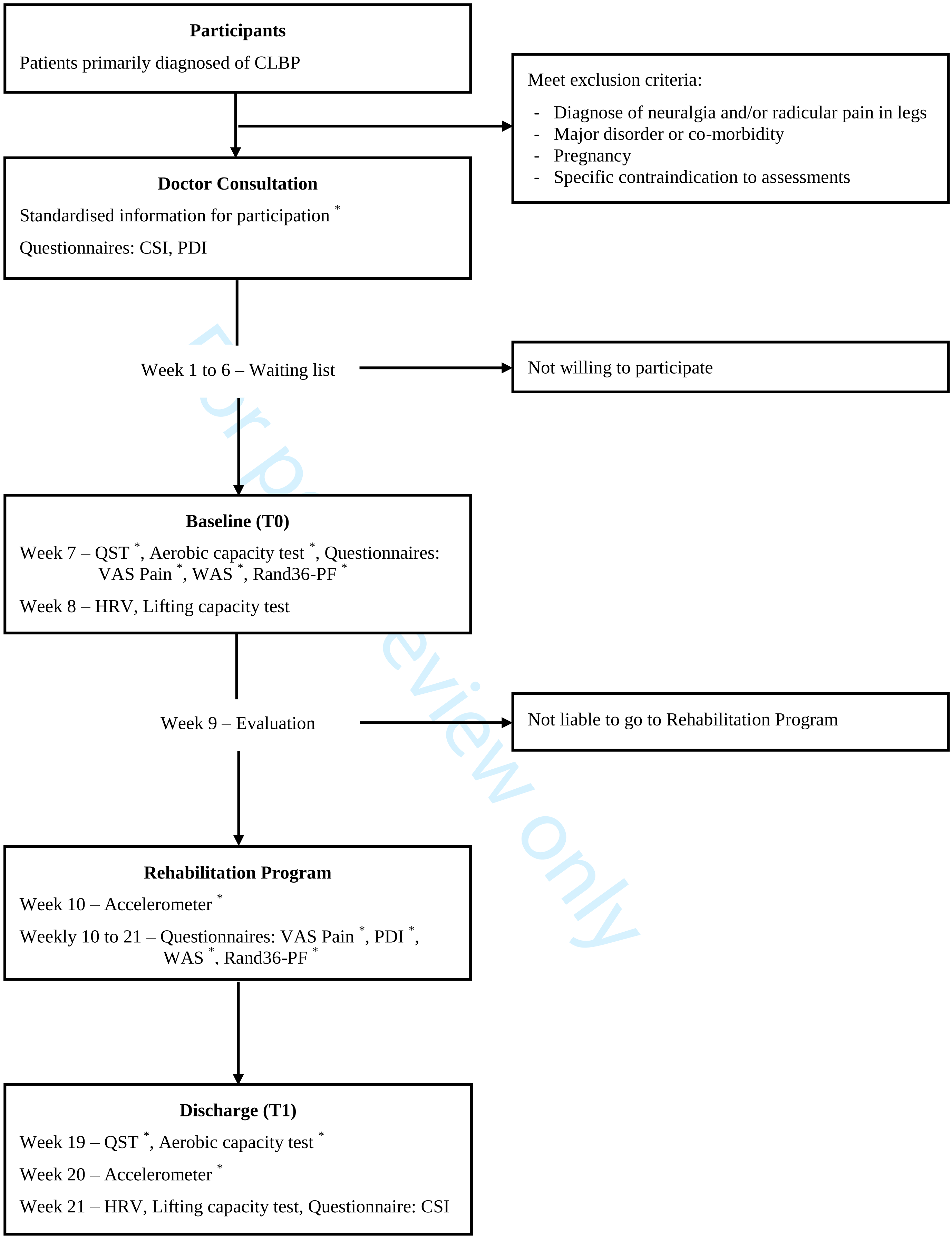

For peer review only

SUPPLEMENTARY FILE

Supplementary material 1. Description of the locations for the QST assessment.

Measuring Location	Description
**1. m. Deltoid – Training location**	In the pars medialis of m. Deltoid, at the contralateral side of the body to the most painful area located; 4 cm below the Acromion. For the CPM test the measurement is at the non-dominant m. Deltoid.
**2. Most painful area**	As stated by the patient. This location is thoroughly described in the form to repeat the measurement in the same exact location at discharge.
**3. Low back – Control location**	Contralateral side of the body mirror-wise to the most painful area located. A specific description of the most painful area is important to identify this location as well.
**4. m. Trapezius – Control location**	In the pars medialis of m. Trapezius, both ipsilateral and contralateral to the most painful area located. At the level of the spine of the Scapula (T3); 4 cm lateral / external to the backbone.
**5. m. Quadriceps – Control location**	In the rectus femoris of m. Quadriceps, both ipsilateral and contralateral to the most painful area located; 15 cm above the upper edge of the Patella. For the CPM test the measurement is at the non-dominant m. Quadriceps.

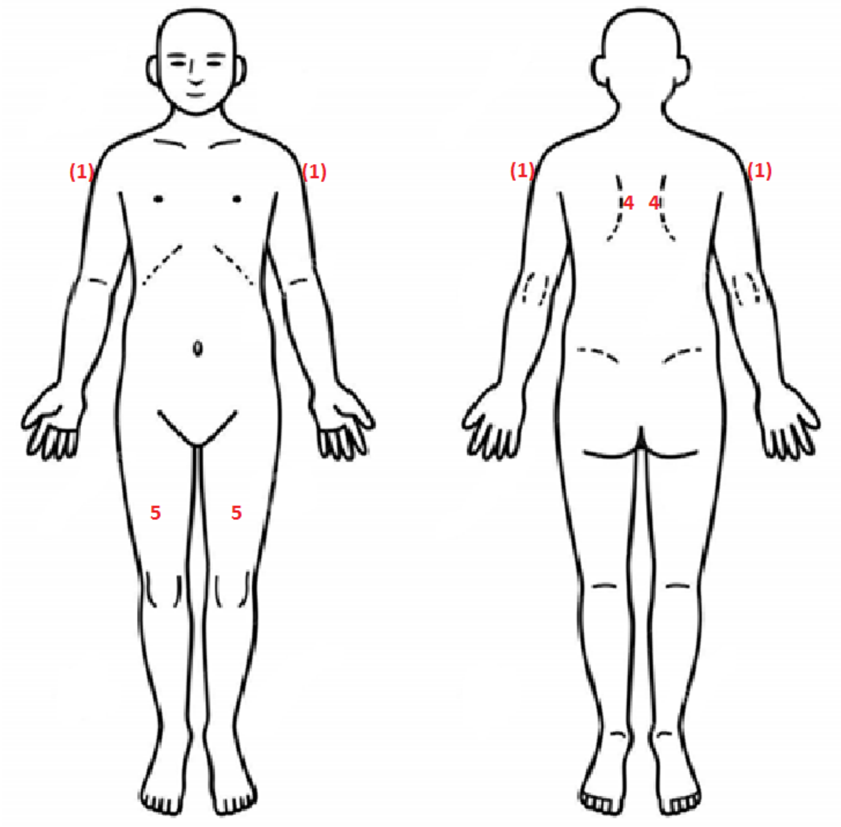

For peer review only - http://bmjopen.bmj.com/site/about/guidelines.xhtml

SPIRIT

STANDARD PROTOCOL ITEMS: RECOMMENDATIONS FOR INTERVENTIONAL TRIALS

SPIRIT 2013 Checklist: Recommended items to address in a clinical trial protocol and related documents*

Section / Item	Item No.	Description	Check
**Administrative information**			
Title	1	Descriptive title identifying the study design, population, interventions, and, if applicable, trial acronym	X
Trial registration	2a	Trial identifier and registry name. If not yet registered, name of intended registry	X
	2b	All items from the World Health Organization Trial Registration Data Set	X
Protocol version	3	Date and version identifier	X
Funding	4	Sources and types of financial, material, and other support	X
Roles and responsibilities	5a	Names, affiliations, and roles of protocol contributors	X
	5b	Name and contact information for the trial sponsor	NA
	5c	Role of study sponsor and funders, if any, in study design; collection, management, analysis, and interpretation of data; writing of the report; and the decision to submit the report for publication, including whether they will have ultimate authority over any of these activities	NA
	5d	Composition, roles, and responsibilities of the coordinating centre, steering committee, endpoint adjudication committee, data management team, and other individuals or groups overseeing the trial, if applicable (see Item 21a for data monitoring committee)	NA
**Introduction**			
Background and rationale	6a	Description of research question and justification for undertaking the trial, including summary of relevant studies (published and unpublished) examining benefits and harms for each intervention	X
	6b	Explanation for choice of comparators	X
Objectives	7	Specific objectives or hypotheses	X
Trial design	8	Description of trial design including type of trial (eg, parallel group, crossover, factorial, single group), allocation ratio, and framework (eg, superiority, equivalence, noninferiority, exploratory)	X

For peer review only

For peer review only - http://bmjopen.bmj.com/site/about/guidelines.xhtml

**Methods: Participants, interventions, and outcomes**			
Study setting	9	Description of study settings (eg, community clinic, academic hospital) and list of countries where data will be collected. Reference to where list of study sites can be obtained	**X**
Eligibility criteria	10	Inclusion and exclusion criteria for participants. If applicable, eligibility criteria for study centres and individuals who will perform the interventions (eg, surgeons, psychotherapists)	**X**
Interventions	11a	Interventions for each group with sufficient detail to allow replication, including how and when they will be administered	**NA**
	11b	Criteria for discontinuing or modifying allocated interventions for a given trial participant (eg, drug dose change in response to harms, participant request, or improving/worsening disease)	**NA**
	11c	Strategies to improve adherence to intervention protocols, and any procedures for monitoring adherence (eg, drug tablet return, laboratory tests)	**NA**
	11d	Relevant concomitant care and interventions that are permitted or prohibited during the trial	**NA**
Outcomes	12	Primary, secondary, and other outcomes, including the specific measurement variable (eg, systolic blood pressure), analysis metric (eg, change from baseline, final value, time to event), method of aggregation (eg, median, proportion), and time point for each outcome. Explanation of the clinical relevance of chosen efficacy and harm outcomes is strongly recommended	**X**
Participant timeline	13	Time schedule of enrolment, interventions (including any run-ins and washouts), assessments, and visits for participants. A schematic diagram is highly recommended (see Figure)	**X**
Sample size	14	Estimated number of participants needed to achieve study objectives and how it was determined, including clinical and statistical assumptions supporting any sample size calculations	**X**
Recruitment	15	Strategies for achieving adequate participant enrolment to reach target sample size	**X**
**Methods: Assignment of interventions (for controlled trials)**			**NA**
Allocation:			
Sequence generation	16a	Method of generating the allocation sequence (eg, computer-generated random numbers), and list of any factors for stratification. To reduce predictability of a random sequence, details of any planned restriction (eg, blocking) should be provided in a separate document that is unavailable to those who enrol participants or assign interventions	**NA**

For peer review only - http://bmjopen.bmj.com/site/about/guidelines.xhtml

Allocation concealment mechanism	16b	Mechanism of implementing the allocation sequence (eg, central telephone; sequentially numbered, opaque, sealed envelopes), describing any steps to conceal the sequence until interventions are assigned	**NA**
Implementation	16c	Who will generate the allocation sequence, who will enrol participants, and who will assign participants to interventions	**NA**
Blinding (masking)	17a	Who will be blinded after assignment to interventions (eg, trial participants, care providers, outcome assessors, data analysts), and how	**NA**
	17b	If blinded, circumstances under which unblinding is permissible, and procedure for revealing a participant's allocated intervention during the trial	**NA**
**Methods: Data collection, management, and analysis**			
Data collection methods	18a	Plans for assessment and collection of outcome, baseline, and other trial data, including any related processes to promote data quality (eg, duplicate measurements, training of assessors) and a description of study instruments (eg, questionnaires, laboratory tests) along with their reliability and validity, if known. Reference to where data collection forms can be found, if not in the protocol	**X**
	18b	Plans to promote participant retention and complete follow-up, including list of any outcome data to be collected for participants who discontinue or deviate from intervention protocols	**NA**
Data management	19	Plans for data entry, coding, security, and storage, including any related processes to promote data quality (eg, double data entry; range checks for data values). Reference to where details of data management procedures can be found, if not in the protocol	**X**
Statistical methods	20a	Statistical methods for analysing primary and secondary outcomes. Reference to where other details of the statistical analysis plan can be found, if not in the protocol	**X**
	20b	Methods for any additional analyses (eg, subgroup and adjusted analyses)	**X**
	20c	Definition of analysis population relating to protocol non-adherence (eg, as randomised analysis), and any statistical methods to handle missing data (eg, multiple imputation)	**X**
**Methods: Monitoring**			**NA**
Data monitoring	21a	Composition of data monitoring committee (DMC); summary of its role and reporting structure; statement of whether it is independent from the sponsor and competing interests; and reference to where further details about its charter can be found, if not in the protocol. Alternatively, an explanation of why a DMC is not needed	**NA**

For peer review only - http://bmjopen.bmj.com/site/about/guidelines.xhtml

	21b	Description of any interim analyses and stopping guidelines, including who will have access to these interim results and make the final decision to terminate the trial	**NA**
Harms	22	Plans for collecting, assessing, reporting, and managing solicited and spontaneously reported adverse events and other unintended effects of trial interventions or trial conduct	**NA**
Auditing	23	Frequency and procedures for auditing trial conduct, if any, and whether the process will be independent from investigators and the sponsor	**NA**
**Ethics and dissemination**			
Research ethics approval	24	Plans for seeking research ethics committee/institutional review board (REC/IRB) approval	**X**
Protocol amendments	25	Plans for communicating important protocol modifications (eg, changes to eligibility criteria, outcomes, analyses) to relevant parties (eg, investigators, REC/IRBs, trial participants, trial registries, journals, regulators)	**X**
Consent or assent	26a	Who will obtain informed consent or assent from potential trial participants or authorised surrogates, and how (see Item 32)	**X**
	26b	Additional consent provisions for collection and use of participant data and biological specimens in ancillary studies, if applicable	**NA**
Confidentiality	27	How personal information about potential and enrolled participants will be collected, shared, and maintained in order to protect confidentiality before, during, and after the trial	**X**
Declaration of interests	28	Financial and other competing interests for principal investigators for the overall trial and each study site	**X**
Access to data	29	Statement of who will have access to the final trial dataset, and disclosure of contractual agreements that limit such access for investigators	**X**
Ancillary and post-trial care	30	Provisions, if any, for ancillary and post-trial care, and for compensation to those who suffer harm from trial participation	**NA**
Dissemination policy	31a	Plans for investigators and sponsor to communicate trial results to participants, healthcare professionals, the public, and other relevant groups (eg, via publication, reporting in results databases, or other data sharing arrangements), including any publication restrictions	**X**
	31b	Authorship eligibility guidelines and any intended use of professional writers	**X**
	31c	Plans, if any, for granting public access to the full protocol, participant-level dataset, and statistical code	**NA**

For peer review

Appendices

Informed consent materials	32	Model consent form and other related documentation given to participants and authorised surrogates	**-**
Biological specimens	33	Plans for collection, laboratory evaluation, and storage of biological specimens for genetic or molecular analysis in the current trial and for future use in ancillary studies, if applicable	**NA**

*It is strongly recommended that this checklist be read in conjunction with the SPIRIT 2013 Explanation & Elaboration for important clarification on the items. Amendments to the protocol should be tracked and dated. The SPIRIT checklist is copyrighted by the SPIRIT Group under the Creative Commons "Attribution-NonCommercial-NoDerivs 3.0 Unported" license.

For peer review only

BMJ Open

Central Sensitisation and Functioning in patients with Chronic Low Back Pain: Protocol for a cross-sectional and cohort study

Journal:	*BMJ Open*
Manuscript ID	bmjopen-2019-031592.R4
Article Type:	Protocol
Date Submitted by the Author:	17-Jan-2020

Complete List of Authors:	Ansuategui Echeita, Jone; University Medical Center Groningen, Rehabilitation Medicine<br>Schiphorst Preuper, Henrica ; University Medical Center Groningen, Rehabilitation Medicine<br>Dekker, Rienk; University Medical Center Groningen, Rehabilitation Medicine; University Medical Center Groningen, Center for Sports Medicine<br>Stuive, Ilse; University Medical Center Groningen , Rehabilitation Medicine<br>Timmerman, Hans; Radboud University Medical Center, Department of Anesthesiology, Pain and Palliative Medicine<br>Wolff, Andre; University Medical Center Groningen, Anesthesiology Pain Center<br>Reneman, Michiel; University Medical Center Groningen, Rehabilitation Medicine

**Primary Subject Heading**:	Rehabilitation medicine
Secondary Subject Heading:	Anaesthesia, Sports and exercise medicine, Occupational and environmental medicine
Keywords:	Hyperalgesia, Autonomic Nervous System, Lifting, Exercise Test, Physical Activity, Disability

SCHOLARONE™
Manuscripts

I, the Submitting Author has the right to grant and does grant on behalf of all authors of the Work (as defined in the below author licence), an exclusive licence and/or a non-exclusive licence for contributions from authors who are: i) UK Crown employees; ii) where BMJ has agreed a CC-BY licence shall apply, and/or iii) in accordance with the terms applicable for US Federal Government officers or employees acting as part of their official duties; on a worldwide, perpetual, irrevocable, royalty-free basis to BMJ Publishing Group Ltd ("BMJ") its licensees and where the relevant Journal is co-owned by BMJ to the co-owners of the Journal, to publish the Work in this journal and any other BMJ products and to exploit all rights, as set out in our licence.

The Submitting Author accepts and understands that any supply made under these terms is made by BMJ to the Submitting Author unless you are acting as an employee on behalf of your employer or a postgraduate student of an affiliated institution which is paying any applicable article publishing charge ("APC") for Open Access articles. Where the Submitting Author wishes to make the Work available on an Open Access basis (and intends to pay the relevant APC), the terms of reuse of such Open Access shall be governed by a Creative Commons licence – details of these licences and which Creative Commons licence will apply to this Work are set out in our licence referred to above.

Other than as permitted in any relevant BMJ Author's Self Archiving Policies, I confirm this Work has not been accepted for publication elsewhere, is not being considered for publication elsewhere and does not duplicate material already published. I confirm all authors consent to publication of this Work and authorise the granting of this licence.

For peer review only

Central Sensitisation and Functioning in patients with Chronic Low Back Pain: Protocol for a cross-sectional and cohort study

J. Ansuategui Echeita [1], H.R. Schiphorst Preuper [1], R. Dekker [1], I. Stuive [1], H. Timmerman [2, 3], A.P. Wolff [3], M.F. Reneman [1].

Affiliations:

[1] Department of Rehabilitation Medicine, University of Groningen, University Medical Center Groningen, Groningen, The Netherlands.

[2] Department of Anesthesiology, Pain and Palliative Medicine, Radboud university medical center, Nijmegen, The Netherlands.

[3] Department of Anesthesiology Pain Center, University of Groningen, University Medical Center Groningen, Groningen, The Netherlands.

Corresponding author: Jone Ansuategui Echeita, Department of Rehabilitation Medicine, University Medical Center Groningen, P.O. Box 30.002, 9750 RA Haren, The Netherlands. Email: j.ansuategui.echeita@umcg.nl

For peer review only

ABSTRACT

Introduction. A relevant subsample of patients with Chronic Low Back Pain (CLBP) have manifested augmented central pain processing, Central Sensitisation (CS). Patients with CLBP have limited functioning and participation. Theoretically, physical functioning in patients with CLBP can plausibly be linked to CS; however, evidence to explain such association is scarce. Moreover, there is no gold standard for CS diagnosis. The objectives of the study are:

1) To analyse the association between instruments assessing reference symptoms and signs attributed to CS.
2) To analyse whether reference symptoms and signs attributed to CS are associated with functioning measurement outcomes.
3) To analyse whether changes (between baseline and discharge) in reference symptoms and signs attributed to CS are related to changes in each of the functioning measurement outcomes.

Methods and analysis. A cross-sectional and longitudinal observational study is performed with measurements taken at baseline and discharge of an interdisciplinary rehabilitation program. A sample size of 110 adult patients with CLBP has been calculated for the study. CS measurements are: Central Sensitisation Inventory, Quantitative Sensory Testing and Heart-Rate Variability. Functioning measurements are: Lifting capacity, Maximal aerobic capacity, Accelerometry, and Reported functioning. Statistical analyses to be performed are: 1) correlation between CS measurements, 2) multiple regression between functioning (dependent variable) and CS measurements (independent variable), 3) multiple regression between changes in scores of functioning (dependent variable) and CS measurements (independent variable), and corrected for sex and age.

Ethics and dissemination. The study obtained the clearance to its implementation from the Medical Research Ethics Committee of the University Medical Center Groningen (METc UMCG) in July 2017. The results will be disseminated through scientific publications in peer-reviewed journals, presentations at relevant conferences, and reports to stakeholders.

Trial registration number. NTR7167 / NL6980.

KEYWORDS

Hyperalgesia, Autonomic Nervous System, Somatosensory, Lifting, Exercise Test, Physical Activity, Disability.

For peer review only

STRENGTHS AND LIMITATIONS OF THIS STUDY

- Novel and comprehensive approaches for the assessment of the presence of CS.
- Extensive amount of measurements from multiple domains for the determination of functioning status.
- Longitudinal study with measurements at baseline and discharge of an interdisciplinary rehabilitation program implemented as ‘Care as Usual’.
- Strategies allocated to promote participation and adherence, and to mitigate selection bias to the largest extent possible.
- Statistical analyses are performed with selections of CS and functioning measurements, and these selections may influence study results.

For peer review only

INTRODUCTION

Background

Twenty percent of the general population experiences prolonged or recurrent pain for at least three months, such pain is then considered to be chronic.[1,2] Low back pain is the principal cause of Years Lived with Disability (YLD) and work days lost in Europe and worldwide,[3] and its sequelae affect 9% of the global population.[4] Moreover, although there are several treatment intervention strategies available for patients with Chronic Low Back Pain (CLBP), treatment effect sizes have only been moderate.[5] Such evidence may explain why 40% of the patients are not satisfied with the treatment received.[2] The economic consequences of chronic pain include direct costs of medical care and pain management treatments, as well as indirect costs of decreased work productivity and sick leave, and of disability and sickness-retirement.[2,6] The indirect costs determine 86-88% of the total costs produced by patients with CLBP.[7,8] In particular, in the Netherlands, the costs derived from back pain to Dutch society were 3.5 billion in 2007, corresponding to 0.6% of the Gross National Product.[8]

In the past decennia, a relevant subsample of patients with chronic pain, including individuals with CLBP, have shown an increased responsiveness to noxious and non-noxious stimuli, described as Central Sensitization (CS).[9] CS is a result of an imbalance in the nociceptive pathways ("pain pathways") and supraspinal structures due to an amplified facilitation and/or reduced inhibition.[10–12] The phenomenon, CS, is manifested by an amplified pain perception regarding its intensity (hyperalgesia and allodynia), duration (aftersensations and temporal summation), and distribution (expansion of the receptive field), as well as a reduced conditioned pain modulation.[12] There is currently no gold standard for CS diagnosis. Identifying reference clinical symptoms and signs, however, may indicate the presence of CS: disproportionate pain, diffuse distribution, allodynia and hyperalgesia, hypersensitivity unrelated to mechanical stimulus but rather to environmental sensations (light, temperature, noise or other stressors), maladaptive psychosocial factors, and low vagal nerve activity.[13–16]

Chronic pain is a multifactorial condition which has an impact on physical or body structures and functions, on psychological processes and on daily activities and quality of life of individuals.[2,17,18] Limited functioning or capacity to perform everyday tasks (essential for an independent living) affects individuals with chronic pain in their mobility and self-care, social relationships, work and leisure.[19] Individuals' functioning is composed of body functions (body level) –described by impairments they experience–and of activities and participation (personal and social levels, respectively) –described by capacity and performance tests.[18,20] An extensive overview of the functioning status is best obtained when the assessment includes suitable and relevant measures from multiple domains of daily living. In particular, Lifting capacity, Physical Activity (PA), aerobic capacity, or daily activities participation, are measures that account for the multiple operationalizations of functioning in the daily living.

For peer review only

Patients with CLBP are likely to be less physically active, and be more deconditioned and disabled.[3,21-23] A subsample of patients with CLBP can manifest symptoms and signs of CS being present.[9] Because patients with CS are more reactive to stimuli and their pain experience is enhanced, the presence of CS and/or higher levels of CS could plausibly be associated with lower physical functioning and participation in patients with CLBP, and vice versa. More severe symptomatology of CS, decreased parasympathetic/vagal activity, or altered somatosensory responses have been associated with greater self-reported pain-related disability.[24-30] However, the associations between altered somatosensory responses and self-reported disability, were dependent on the site of testing, the stimuli and the type of tests performed. Also, CS has been assessed with different instruments that measure specific CS clinical symptoms,[12] lacking a comprehensive assessment of CS in which various reference clinical symptoms are measured together. Moreover, functioning has most frequently been assessed with self-reported disability which is not exclusively representative of the functioning status of the patient.

The study described in this research protocol has been created to improve the assessment and management of pain and disability of centrally sensitised patients with CLBP and fill the existing gaps in knowledge. On the one hand, it intends to assist on the phenotyping of centrally sensitised patients with CLBP. To this purpose, an integrated assessment of CS combining the use of various instruments which measure different reference clinical symptoms of CS is aimed. Instruments like Central Sensitization Inventory (CSI),[31] Quantitative Sensory Testing (QST) [32,33] and Heart-Rate Variability (HRV),[34] are non-invasive, feasible and assess different reference clinical symptoms and signs indicative of CS.[12] In the absence of a gold standard, these instruments measure different reference indicators of CS and can also be complementary to describe CS. On the other hand, it intends to improve the understanding of CS phenomenon and its contribution to functioning status. Lifting capacity, PA levels and/or PA distribution or patterns, aerobic capacity and daily activities participation, are representative of several tasks of the daily living usually limited in patients with CLBP. These capacity and performance tests and self-reported functioning measures can give an extensive overview of the functioning status.

Objective(s)

The objectives of the studies covered by this research protocol are:

1. To analyse the association between three instruments assessing reference symptoms and signs attributed to CS in patients with CLBP: CSI, QST and HRV.
2. To analyse whether reference symptoms and signs attributed to CS are associated with functioning measurement outcomes in patients with CLBP. Specifically, to analyse the association between the three instruments assessing

For peer review only - http://bmjopen.bmj.com/site/about/guidelines.xhtml

CS (CSI, QST and HRV) and: Lifting capacity, Aerobic capacity, PA levels and/or PA distribution or patterns, and patient-reported measurements.

3. To analyse whether changes (between baseline and discharge) in reference symptoms and signs attributed to CS are related to changes in each of the functioning measurement outcomes in patients with CLBP. Specifically, to analyse whether decreases in CS described as: a lower CSI score, an increase in the pain thresholds (QST), and an increase in parasympathetic/vagal tone (HRV), are related to a better functioning described as: an increase in lifting capacity, an increase in aerobic capacity, an increase in PA levels and/or a change in PA distribution or patterns, and better outcome scores in patient-reported measurements.

METHODS AND ANALYSES

Study design

An observational study project with both cross-sectional and longitudinal designs is being conducted from September 2017 to September 2019 in the Center for Rehabilitation of the University Medical Center Groningen (CvR – UMCG) in The Netherlands.

Patient and Public Involvement

Personnel of the CvR-UMCG collaborate in the study, in particular, the Pain rehabilitation team during patient management and data collection process and the Central exercise lab team on data collection process. The authors wish to express their gratitude to the collaborators and to the patients that participate.

Participants

Consecutive patients between 18 and 65 years of age at the time of recruitment who are primarily referred to the outpatient Pain Rehabilitation Department of the CvR - UMCG due to a chronic primary low back pain and who are assessed for the interdisciplinary pain rehabilitation program, are eligible to participate in the study. Chronic primary low back pain is defined as a low back pain recurrent for more than three months, related to emotional distress and/or functional disability, and whose pain is not a result of any other diagnosis [35] even if patients may have undergone surgery in the past. Additionally, patients are invited to the study if they are mentally competent and can follow instructions. Furthermore, patients are excluded from the study if, based on their file, they: have a specific diagnosis that would better account for the symptoms (cancer, osteoporosis, rheumatologic inflammatory diseases and/or spinal

For peer review only

fractures), have neuralgia and/or radicular pain in the legs, have severe psychiatric disorders, show sensitivity alterations in measurement location(s), are pregnant (or planning to be), have any specific contraindication according to the standards of practice guidelines of the assessments that they are going to be subjected to,[36,37] or take any medication that may interfere with the assessment outcomes such as calcium channel blockers on HRV. CS-related comorbidities (i.e. fibromyalgia, osteoarthritis or chronic fatigue syndrome) are no reason for exclusion from the study. The use of opioid or pain-related medication is neither a criterion for exclusion and such information is recorded.

Procedures

Measurements are performed as part of 'Care As Usual' (CAU) for outpatients following the Pain Rehabilitation guidelines of the CvR – UMCG. However, not all measurements of the research protocol belong to the battery of assessments of the CvR – UMCG, as pointed out in the legend of Figure 1.

During the first appointment of the patients with the rehabilitation physician, patients are invited to participate in the studies if based on their file they are eligible according to the criteria defined for the studies. Eligible patients who are invited to participate and receive information about the study, but have not reacted, are contacted in a week's time to remind them about the study participation. Those who sign the informed consent, fill in forms with their relevant personal information during the baseline assessment and are given vouchers for the travel expenses during the discharge assessment. Participating patients follow CAU trajectory with the Pain Rehabilitation team in terms of assessments, screening and implementation of the interdisciplinary rehabilitation program. As part of the research protocol, the performance of additional assessments and filling of questionnaires on CS and functioning are required. CSI, lifting capacity and HRV results are collected as CAU. Aerobic capacity, QST, objective PA monitoring, and pain intensity and functioning questionnaires' results are collected as additional to CAU. All CS measurements, objective PA monitoring and capacity tests are measured at two time points, baseline and discharge. Pain intensity and functioning questionnaires, on the other hand, are measured at 13 time points, baseline and once a week during the 12-week rehabilitation program. PDI is the only functioning questionnaire collected as CAU at baseline but as additional during the rehabilitation program. A diagram of the project procedure is presented as Figure 1.

(Insert Figure 1 around here)

Measurements

Central Sensitisation measurements

For peer review only - http://bmjopen.bmj.com/site/about/guidelines.xhtml

In order to comprehensively assess the symptoms and signs attributed to CS, data is obtained from a questionnaire (CSI) and two physical assessments (QST and HRV).

CSI. The inventory is a self-reported questionnaire with two sections to estimate the severity of CS-related symptoms. Section A is a 25-item questionnaire to assess the presence of the most common CS-related symptoms. Each of the questions is measured on a 5-point Likert scale from 0 (never) to 4 (always), and the total score ranges from 0 to 100, where higher scoring is associated with more severe symptomatology. Section B is a 10-item questionnaire to assess the previously diagnosed CS Syndromes (CSS) and/or conditions related to CS. Higher amount of CSS is associated with higher chances of CS being present. This instrument has shown excellent test-retest reliability (ICC = 0.82-0.97) and internal consistency (Cronbach α = 0.87-0.91) in the general population and in patients with chronic pain,[31,38-40] as well as good concurrent validity (sensitivity = 81%, specificity = 75%) for CSS diagnosis within patients with chronic pain.[38] For the purposes of the study the Dutch translation of the CSI is used,[41] which has also shown excellent internal consistency (Cronbach α = 0.91) and test-retest reliability (ICC = 0.91 and 0.88) in controls and in patients with chronic musculoskeletal pain respectively.[38-40] as well as good concurrent validity (sensitivity = 81%, specificity = 75%) for CSS diagnosis within patients with chronic pain.[38] For the purposes of the study the Dutch translation of the CSI is used,[41] which has also shown excellent internal consistency (Cronbach α = 0.91) and test-retest reliability (ICC = 0.91 and 0.88) in controls and in patients with chronic musculoskeletal pain respectively.[42] The psychometric information of CSI in patients with CLBP is not known.

QST. A battery of standardised sensory tests that quantifies the function of the somatosensory system. QST tests the characteristics of various sensation and nociceptive processes as representative of afferent nerve fibres and central pathways performance. The whole assessment lasts for one hour, and it consists of six sensory tests, which are measured in seven different predefined body locations: one training location to familiarise the patient with the tests, the most painful location (as pointed out by the patient), and five control locations. See Supplementary material 1 for full description of body locations. The standardised instructions of the sensory tests are read to the patient at each test performance. Of the tests, five consist of mechanical stimuli based on and performed following the QST examination of the German Research Network on Neuropathic Pain (DFNS) protocol,[32] and one for conditioned pain modulation following the Nijmegen-Aalborg Screening QST (NASQ) protocol.[33,43] Overall, the DFNS protocol has shown good test-retest (mean r = 0.86) and inter-observer reliability (mean r = 0.83) in patients with affections in the somatosensory system either with or without pain.[44] For the purpose of avoiding bias due to the order of the measurements and locations, a counterbalancing procedure is established, which accounts for the locations and the measurements to be made. This procedure provides three different assessment order groups, all of which begin with the measurements at the training location and end with the conditioned pain modulation test. An additional counterbalancing procedure has been

For peer review only - http://bmjopen.bmj.com/site/about/guidelines.xhtml

made for allocating patients to these assessment order groups (either 1, 2, or 3) every nine patients a different allocation order is performed to ensure a similar amount of QST assessments per group. All assessors are trained in the QST protocol. The following sensory tests are performed:

- *The Dynamic Mechanical Allodynia (DMA)* to test the presence of abnormal stimuli-evoked pain is assessed with a soft brush (SENSElab™, Brush-05, Somedic AB, Hörby, Sweden) that is swiped 1-2 cm over the skin. This is performed on a single repetition and on a train of three repetitions (wind-up response). At each of the seven body locations (see Supplementary material 1) it is recorded whether patients feel the sweeping and its pain intensity with a Numeric Rating Scale (NRS) ranging from 0 (no pain) to 100 (most intense pain imaginable) for both the single swipe and the series.[45,46] The DMA (ratio) is calculated as: DMA = (NRS-3 / NRS-1). If NRS-1 is 0, the DMA for that location cannot be calculated and it will be registered as a missing value. The DMA test according to the DFNS protocol has shown to have good test-retest (r = 0.87) and inter-observer reliability (r = 0.79).[44]
- *The Mechanical Pain Threshold (MPT)* to test the ability to perceive sharp, pricking, or stinging stimuli is assessed with a set of standardised weighted pinprick stimulators with seven different intensities (The PinPrick stimulator set, MRC Systems GmbH, Germany) and with a modified method of limits. Starting at the lowest intensity (8mN), it progressively increases until patients report the stimulus to change from touch to painful (sharp, pricking or stinging). The intensity of the pinprick that produces the first painful stimulus in mN is recorded in each of the seven locations (see Supplementary material 1).[46-49] The MPT test according to the DFNS protocol has shown to have good test-retest (r = 0.80) and inter-observer reliability (r = 0.80).[44]
- *The Wind-Up Ratio (WUR)* to test the Nervous System's painful stimuli temporal summation or responsiveness to repeated noxious stimuli. Such test is assessed with a suprathreshold pinprick stimulator of 256mN (the PinPrick stimulator set, MRC Systems GmbH, Germany), which is applied on a single repetition and on a train of ten repetitions in an area of 1cm^2 with intervals of one second (wind-up response). The pain intensity measured with a Numeric Rating Scale (NRS) ranging from 0 (no pain) to 100 (most intense pain imaginable) for both the single repetition and the series are recorded in each of the seven body locations (see Supplementary material 1).[50,51] The WUR (ratio) is calculated as: WUR = (NRS-10 / NRS-1). If NRS-1 is 0, the WUR for that location cannot be calculated and it will be registered as a missing value. The WUR test according to the DFNS protocol has shown to have poor test-retest (r = 0.67) and inter-observer reliability (r = 0.56).[44]
- *Mechanical Detection Threshold (MDT)* to test the ability to detect light touch, is assessed with a standardised set of von Frey filaments (OptiHair2-Set, Marstock Nervtest, Germany) with a modified method of limits.[52,53] Starting at the intensity of 16mN, the stimulus application progressively decreases until patients do not report

For peer review only

any sensation. The intensity of the filament that is not perceived in mN is recorded in each of the seven body locations (see Supplementary material 1).[54] The MDT test, according to the DFNS protocol, has shown to have good test-retest (r = 0.90) and inter-observer reliability (r = 0.89).[44]

- *Pressure Pain Threshold (PPT)* to test the ability to perceive pressure pain stimuli on muscles, is assessed with an electronic pressure algometer (Force Ten ™ FDX, Wagner Instruments, USA). With this tool pressure in the contact point (1cm^2 tip) is progressively but steadily increased until patients report to feel pressure change towards painful (burning, drilling or aching). The intensity in N when pressure becomes painful and its pain intensity with a Numeric Rating Scale (NRS) ranging from 0 (no pain) to 100 (most intense pain imaginable) scale is recorded in each of the seven body locations (see Supplementary material 1).[32] The PPT test according to the DFNS protocol has shown to have good test-retest (r = 0.88) and inter-observer reliability (r = 0.84).[44]
- *Conditioned Pain Modulation (CPM)* to test the Nervous System's descending pain processing. The CPM test methodology selected for this study follows the recommendations of the EFIC meeting (2013).[55] The pain modulation is assessed by means of PPT tests (Test Stimuli, TS) in a distant location from patients dominant hand (m. Deltoid and m. Quadriceps of the non-dominant side, see Supplementary material 1) before and after immersing the dominant hand in a bucket full with ice and cold water (Cold Conditioning Stimuli, cCS). First, PPT measurements at distant locations are made; then, patients immerse the dominant hand in the bucket for as long as they possibly can with a maximal duration of three minutes; finally, PPT measurements are repeated. The PPT results, intensity in N and pain intensity with a Numeric Rating Scale (NRS) ranging from 0 (no pain) to 100 (most intense pain imaginable) scale, in both locations and the time the hand is immersed are recorded. The CPM (%) is calculated as: CPM = [(PPTpost – PPTpre) / PPTpre] ∗ 100; where lower values indicate lower alteration on the nociceptive pathways.[33,43] The CPM test, as the combination of the PPT handheld algometer and cold stimuli, has shown to be one of the most reliable methods with a modest test-retest reliability (ICC = 0.49 and Coefficient of Variation, CV = 63.6%).[56]

HRV. The test provides an indication of the function and balance of the Autonomic Nervous System (ANS), which is responsible for the control of internal functions including self-regulation, adaptability and resilience capacities.[57] The ANS is susceptible to mental and emotional processes, which in turn can produce an imbalance in self-regulation (affecting among others HR) by an hyper-activation of the sympathetic tone and reduced parasympathetic/vagal tone.[15,31] HRV testing can monitor heart rhythms, or time and pattern variation on HR and inter-beat (RR) intervals, and can be used to assess the function of the ANS.[58,59] The HRV test is performed by assessors trained by the HeartMath institute, and the 5-minute protocol is executed to guarantee the comparison of the results as recommended by the Task Force for short-term recordings.[58] Patients are instructed to sit calmly and breathe normally, while they

For peer review only - http://bmjopen.bmj.com/site/about/guidelines.xhtml

have an ear pulse sensor placed which is connected to a computer.[60] The following measures are collected and recorded with the emWave PC Software (emWave®, HeartMath Inc., Boulder Creek, California): mean HR in beats per minute (bpm), mean RR interval in ms; time domain measures in ms as Standard Deviation of Normal-to-Normal interval (SDNN) indicator for cyclic components responsible of RR interval variability, Root Mean Square of Successive Differences (RMSSD) for beat-to-beat variance in HR and parasympathetic/vagal tone; frequency domain measures in ms^2/Hz as Very Low Frequency (VLF, 0.0033-0.04 Hz) indicator for long term regulation and sympathetic tone, Low Frequency (LF, 0.04-0.15 Hz) for sympathetic and parasympathetic/vagal tones, and High Frequency (HF, 0.15-0.40 Hz) for parasympathetic/vagal tone related to breathing rate; and Normalised Coherence indicator for HRV stable or regular pattern over time in %, where higher scores mean more harmonic signal.

Physical Functioning measurements

In order to determine the functioning status of individuals with chronic pain in multiple domains, data is obtained from two physical assessments (lifting and aerobic capacity tests), an accelerometer (PA level), and a battery of reported functioning questionnaires.

Lifting capacity test. The Floor-to-waist lift capacity test is based on the WorkWell Functional Capacity Evaluation (FCE) protocol [61] and is performed to measure patients work functioning or capacity. Such FCE lift test has been shown to have high test–retest (ICC = 0.81) and inter-rater reliability (ICC = 0.76) in patients with CLBP.[62,63] Assessors are trained in the floor-to-waist lift capacity test procedure. Patients are given standardised instructions to repetitively lift a crate with weights from a shelf at waist height to the floor and back. The test begins with a weight that can easily be lifted, progressively the load is increased, and the test ends when the maximum capacity is reached. The final endpoint can be reached due to: cardiac endpoint (85% of maximum heart rate), biomechanical endpoint (unsafe increasing weight because of lack of load handling control), patient endpoint (patient decided to stop), and criterion endpoint (normal end of the test).[64] The maximal load lifted in kg is recorded along with the endpoint reason, and the patient-reported and the assessor-observed mechanical effort as measured with Borg's Category Ratio scale (CR-10).[65] Borg's CR-10 scale ranges from 0 (at rest) to 10 (very, very hard), and has demonstrated to be a valid visual observation of the effort level of patients during their performance of a lifting capacity test.[66]

Aerobic capacity test. The maximal aerobic capacity is assessed with a Cardiopulmonary Exercise Test (CPET). The test is performed with a cycle ergometer (Ergoselect 200p or Ergoselect 200k, Ergoline, Bitz, Germany) following a defined continuous ramp protocol. Before any cycling begins, patients are interviewed about their daily activities, and their resting heart rhythm and function are recorded with an electrocardiogram (ECG) (assy cam-14, GE medical systems, US). Patients then have an unloaded warm-up of three minutes at 60-70 rotations per minute (rpm) before the test begins. At the start of the test, an experienced exercise physiologist in discussion with a specialised physician or

For peer review only - http://bmjopen.bmj.com/site/about/guidelines.xhtml

nurse determine the starting workload (25-100 W) and ramp (5-25 W increase per minute) depending on patients estimated fitness level. During the test patients are asked to maintain a constant cadence while the working load is progressively being increased. Also, they are continuously being verbally encouraged to obtain their maximum performance. The maximal performance is determined by various parameters: a temporary loss of strength and energy (= exhaustion), a plateau in the peak oxygen uptake (VO2max), a Respiratory Exchange Ratio (RER) higher than 1.15, and/or a Heart-Rate (HR) higher than 85% of the predicted maximal HR.[37] Overall, the test lasts for 8-12 minutes and throughout the whole assessment patients are monitored on their cardiac activity with an ECG, on their blood pressure with a cuff algometer, and on their ventilatory gases on a breath-by-breath basis with a metabolic cart (JAEGER ® Vyntus ® CPX, Jaeger, Germany, Hoechberg). The VO2max in ml/min and per kilogram in ml/min/kg is collected as the mean of VO2 over the last 30 seconds of exercise. Along with VO2max, the following measures are collected: peak workload in watts, HR at end of test in beats per minute (bpm), Breathing Frequency (BF) at end of test in breaths per min, expiratory tidal volume (VT) in l, peak energy expenditure in Metabolic Equivalent of Task (MET), test duration in min, and the patient-reported and the assessor-observed mechanical effort as measured with Borg's Rating of Perceived Exertion (RPE) scale. Borg's RPE scale ranges from 6 (no exertion at all) to 20 (maximal exertion) [65] and has shown to be highly correlated with HR (r = 0.74) and blood lactate (r = 0.83),[67] demonstrating to be a reliable instrument to measure work intensity.

PA levels. Objective measurements of PA levels are obtained by means of an accelerometer (ActiGraph GT9X Link, ActiGraph, Pensacola, FL, USA). The accelerometer is a small and light device that monitors wearers' daily PA and energy expenditure. Participating patients wear the accelerometer attached at their hip level for one week continuously during walking hours, in order to ensure the minimum of 4-full-day recording as recommended by activity monitoring reviewers and experts.[68,69] Different algorithms are chosen from the software provided by the manufacturer, ActiLife6 (ActiGraph, Pensacola, FL, USA), to discriminate between low PAs and split patients in three activity intensity categories (sedentary, light, and moderate) [70] and to obtain patients' energy expenditure in kcal [71] and MET.[72] The daily and weekly time in hours and percentage of time spent in each of the activity intensity categories as well as the estimated energy expenditure in both kcal and MET are collected.

Reported Functioning. Data from this domain is obtained from four questionnaires sent to patients and filled by them via internet. All the questionnaires require 10-15 minutes to be filled in.

- *Physical functioning (Rand36 Physical Functioning subscale – Rand36-PF)*. The Rand36 is a health-related quality of life survey that measures the impact of health on patients' well-being and ability to function. Patients are asked to only answer the physical functioning (PF) subscale which looks at the limitations patients experience during their daily activities. Each of the 10 items belonging to the PF subscale have a 3-level

For peer review only - http://bmjopen.bmj.com/site/about/guidelines.xhtml

response choices ranging from 0 (limited a lot) to 100 (not limited at all), the total average score ranges from 0 to 100 too; where a higher score is associated with a greater limitation in performing daily activities. For the purposes of the study, the Dutch translation of the Rand36 is used, which has shown good internal consistency (Cronbach α = 0.92) and test-retest reliability (r = 0.72-0.82) in the general population.[73,74]

- *Disability (Pain Disability Index – PDI)*. The index is a 7-item questionnaire which measures the interference of pain with the ability to function in different daily life activities: family/home responsibilities, recreation, social activity, occupation, sexual behaviour, self-care and life-support activity. Each item ranges from 0 (no interference) to 10 (total interference), and the total score ranges from 0 to 70; where scoring higher is associated with higher interference in daily life activities. The PDI has shown modest test-retest reliability (r = 0.44) and high internal consistency (Cronbach α = 0.86) in patients with chronic pain.[75] For the purposes of the study the Dutch translation of the PDI is used, which has shown a good internal consistency (Cronbach α = 0.85) and test-retest reliability (ICC = 0.76) in patients with musculoskeletal pain.[76]
- *Work ability (Work Ability Score – WAS)*. The score is a single-item question which compares patient's current work ability compared with their lifetime best. The result is a score ranging from 0 (unable to work) to 10 (best working ability). The single-item question is part of the Work Ability Index (WAI), a questionnaire which measures work ability and has shown to be test-retest reliable (difference between test and re-test = -0.53) in construction workers.[77] The WAS has shown to be highly correlated to the 28 items of the WAI among women on a long-term sick leave (r = 0.87) [78] and among active workers (r = 0.63).[79]

Supplementary measurements

<u>*Demographic characteristics*</u>. Patients fill in a form with their details when the QST assessment takes place. Such include: age, sex, nationality, height, weight, pain characteristics (affected body area, duration and medication, if applicable), educational level and employment characteristics (work-status and physical work demands per Dictionary of Occupational Titles (DOT) [80]).

<u>*Pain-related questionnaires.*</u>

- *Pain intensity (Visual Analogue Scale – VAS Pain)*. The scale is a single-item question to assess the current pain intensity in adults. It is a straight line with 'no pain' (score = 0) at one end and 'pain as bad as it could be' (score = 100) at the other, in which patients are asked to mark the line at the level that represents their pain level. The score is the distance between the mark and the 'no pain' end, where scoring higher is associated with greater pain intensity. The VAS Pain has shown good validity (r = 0.62-0.91) and good test-retest reliability (literate r = 0.94 and illiterate r = 0.71) in patients with rheumatic pain conditions.[81] VAS Pain is sent to patients and filled

For peer review only - http://bmjopen.bmj.com/site/about/guidelines.xhtml

in by them via internet along with the battery of functioning questionnaires (described above), i.e. at baseline and weekly during the interdisciplinary rehabilitation program.

- *Body response to a straining exercise (Pain Response Questionnaire – PRQ)*.[82] The questionnaire assesses the pain intensity of the different body locations on an 11-point numeric scale (NRS) ranging from 0 (no pain) to 10 (worst pain imaginable), and the type of pain felt (muscle soreness, other, both or unknown). Additionally it controls for the use of medication and for the performance of any other strenuous physical activity. For the purposes of this study, the body locations measured are predefined to four and the questionnaire is being filled in on three occasions: before the CPET, immediately afterwards and 24 hours later. PRQ is filled at the same moment when the aerobic capacity test is performed (i.e. during baseline and discharge assessments).

Psychological screening questionnaires. These questionnaires measure psychosocial features common to CS syndromes that have demonstrated to affect pain experience and functioning.[83] Patients fill in the questionnaires via internet that are sent per CAU procedures by the Pain Rehabilitation team. Such are:

- *Pain Catastrophizing Scale (PCS)*. The 13-item questionnaire measures in a 5-point Likert scale (0 - not at all, 4 - all the time) the degree to which patients have had catastrophic thoughts or feelings during their pain experience and accounts for three subscales or dimensions: rumination, magnification and helplessness.[84] For the purposes of the study the Dutch translation of the PCS is used,[85] which has shown good internal consistency (Cronbach $\alpha = 0.85\text{-}0.91$) and test-retest reliability ($r = 0.92$) among healthy individuals (students) and patients with chronic low back pain.[86]

- *Injustice Experience Questionnaire (IEQ)*. The 12-item reported questionnaire assesses in a 5-point Likert scale from (0 – never, 4 - all the time) four different aspects of the perceived injustice due to injury: severity, blame, unfairness and irreparability. The questionnaire has shown to have good internal consistency (Cronbach $\alpha = 0.92$) and test-retest reliability (ICC = 0.98) in a group of patients with fibromyalgia.[87] For the purposes of the study the Dutch translation of the IEQ is used, as translated by the Pain in Motion group.[88]

- *Brief Symptom Inventory (BSI)*. The inventory is a 53-item reported questionnaire which assesses patient's current level of psychological distress with a 5-point Likert scale questions (0 - not at all, 4 - extremely), the amount of symptoms present, and their severity.[89] BSI is composed of nine subscales: somatisation, obsession-compulsion, interpersonal sensitivity, depression, anxiety, hostility, phobic anxiety, paranoid ideation and psychoticism. For the purposes of the study the Dutch translation of the BSI is used, which has shown good

For peer review only - http://bmjopen.bmj.com/site/about/guidelines.xhtml

internal consistency (Cronbach α = 0.71-0.87) and test-retest reliability (r = 0.71-0.89) among psychiatric out-patient and healthy individual groups.[90]

Sample size

Due to the limited amount of relevant previous or similar studies, the sample sizes for the several analyses described in this research protocol are estimated a priori with GPower (G*Power for Windows, Version 3.1.9.2), and are exploratory as a result.

The following sample size calculations, as well as the planned statistical analyses later described, will be performed with proxies for the main variables. Additionally, due to the influence of patient's sex and age in the assessment outcomes, all analyses will include these variables for a moderator effect.

Sample size for objective 1: To analyse the association between instruments assessing reference symptoms and signs attributed to CS.

Correlation analyses will be performed for this objective. The correlation between the different CS measurements is expected to be weak as reported on associations between certain CS measures and tests.[24] For a correlation coefficient of 0.25, an α error probability of 0.05 and a power of 0.8, a minimum sample size of 97 is calculated.

Sample size for objective 2: To analyse whether reference symptoms and signs attributed to CS are associated with functioning measurement outcomes; and for objective 3: To analyse whether changes (between baseline and discharge) in reference symptoms and signs attributed to CS are related to changes in each of the functioning measurement outcomes.

Multiple regression analyses will be performed for objectives 2 and 3. Based on the associations between CSI and psychosocial and cognitive behavioural factors,[24,25] the effect size is hypothesised to be medium. Since minimally three CS measurements are tested with sex and age required as independent variables, and controlled for the confounding effect of other demographic and test-related variables, eight predictors are foreseen. Considering the factors mentioned above into account, the sample size calculation results in a minimum of 77 patients, calculated with an α error probability of 0.05 and a power of 0.8. Finally, patients measured at discharge could be about 30% less than at baseline due to either not following the interdisciplinary rehabilitation program or personal reasons; a sample size of 110 patients may be required in this scenario.

Analyses

For peer review only - http://bmjopen.bmj.com/site/about/guidelines.xhtml

Data records from the different tests and questionnaires will be collected and merged into one database. The database management will be done by the project team and handled according to the internal regulations of the UMCG. The analyses of the study will be performed with SPSS software version 22.0 (IBM Corp., NY).

Before any analysis, data will be prepared:

- Variables need to be calculated: BMI; DMA, WUR and CPM tests (see Methods section, Main measurements: CS); PDI, Rand36-PF, PCS, BSI and IEQ questionnaires.
- HRV: data will be pre-processed to correct for any artefacts that may be present at the beginning or at the end of the recording.
- QST: The overall value of each sensory test is the mean at the most painful location and the five control locations together, provided that at most two locations are missing.
- To perform the analyses to respond objective 3, Delta scores are computed (difference in scores between baseline and discharge) for CS, functioning and those confounder variables which are also measured at the same time points as the main measurements.

Proxies of the main measurements were selected to perform analyses. CS variables are: the sum score of CSI part A and the RMSSD for HRV. For QST no variables are predefined, variables that correlate most strongly with each of the functioning measures will be selected. Functioning variables are: the maximal weight lifted for lifting capacity, the VO2max per kg for the maximal aerobic capacity, the daily VM counts for PA, and the average score of Rand36-PF for reported physical functioning.

The presence of missing data will be checked. If a variable has more than 10% of missing data cases, its distribution will be further examined. When the distribution of missing data is missing completely at random, analyses with pairwise deletions are planned. If data is not missing completely at random, other suitable solutions will be applied such as multiple imputation.[91] Regarding the presence of outliers, cases at 3 SD from the mean will be considered as outliers. Their influence will be measured using Cook's distance and leverage values. When the outliers are not statistically influential and/or there is rationale for the outlier, no further actions are planned. The descriptive statistics of the sample characteristics and main test results will be at this point calculated; numerical variables as mean and SD in normally distributed data and as median and interquartile range (IQR 25-75) in non-normally distributed data, and categorical variables as counts and percentage of each category level.

Statistical approach for objective 1: To analyse the association between instruments assessing reference symptoms and signs attributed to CS.

For peer review only - http://bmjopen.bmj.com/site/about/guidelines.xhtml

Multiple partial correlation analyses will be performed and corrected for potential confounders. CS measurements will be the main variables and sex and age will be covariates. Additional covariates may enter the partial correlations based on their correlation coefficient with CS instruments and if $p<0.01$. Results will be reported as correlation coefficients (Pearson's r and/or Spearman's rho) and p-values, with and without controlling for covariates. The significance level for all analyses is established at 0.01 level, to account for the potential Type I error due to the multitude of partial correlation analyses.

Statistical approach for objective 2: To analyse whether reference symptoms and signs attributed to CS are associated with functioning measurement outcomes.

First multiple correlation analyses, followed by multiple regression analyses with data obtained at baseline, will be performed. Each of the main outcomes from functioning measurements will be introduced in the models as dependent variable, CS measurements as independent variables, and sex, age, and other demographic characteristics and/or test-related variables as potential confounders and/or moderators.

Statistical approach for objective 3: To analyse whether changes (between baseline and discharge) in reference symptoms and signs attributed to CS are related to changes in each of the functioning measurement outcomes.

Similar approach to objective 2 will be performed: first multiple correlation analyses will be performed with data obtained at baseline and discharge, followed by multiple correlation analyses using Delta scores of the data, and finally multiple regression analyses again using Delta scores. As in objective 2, each of the main outcomes from functioning measurements will be introduced in the models as dependent variable, CS measurements as independent variables, and sex, age and other demographic characteristics and/or test-related variables as potential confounders and/or moderators.

Confounders entering the final models of objectives 2 and 3 will be selected based on their partial correlation coefficient with CS and functioning, and they will be included if $p \leq 0.10$. All multiple regression models will have the assumptions checked. Results of objectives 2 and 3 will be reported as coefficient (b), confidence intervals (95% CI), p-values and explained variance, with and without controlling for confounders and/or moderators. Due to the large amount of correlation analyses and the risk of high Type I error, a stricter significance level for correlation analyses is established, $p<0.01$. For multiple regression analyses the significance level is at 0.05 level.

DISCUSSION

Several patients with CLBP have shown common manifestations with other chronic pain conditions, which may evidence the presence of CS in these patients. Yet knowledge on CS, especially in patients with CLBP, and on its association with functioning is developing. This research protocol aims to bridge that gap of knowledge with a novel

For peer review only - http://bmjopen.bmj.com/site/about/guidelines.xhtml

approach. In the first place, the study (objective 1) will describe the mechanisms of CS in patients with CLBP using instruments that assess reference symptoms of CS from different perspectives. Furthermore, the study (objectives 2 and 3) will also analyse longitudinally whether the presence of CS and changes of CS can be associated with changes in physical functioning performance, for which functioning will be assessed with several objective monitoring and capacity tests, and self-reported measurements.

Within the constraints of CAU and in the absence of a single gold standard instrument that can diagnose CS, in this research protocol multiple instruments have been selected to examine reference symptoms and signs attributed to CS. 1) The hypersensitivity unrelated to mechanical stimulus are assessed with CSI. A questionnaire designed to measure CS, as it has been shown to be associated to factors known to contribute to CS and is able to identify somatic and emotional symptoms related to CSS.[24,38,92] 2) The disproportionate pain, diffuse distribution, and allodynia and hyperalgesia, are measured with QST. The QST is a battery of non-invasive standardised tests that assists in the identification of sensory and pain thresholds, the determination of pain intensity perception, and the assessment of the conditioned pain modulation. Its results can allow pain characterisation (phenotyping) and processing, and monitoring its progression.[93-97] 3) The low vagal nerve activity is examined with HRV. A non-invasive standardised measure that characterises the function of the ANS.[34] The somatic and emotional symptoms related to CS syndromes may generate an imbalance between a hyperactivation of the sympathetic tone and a reduction of the parasympathetic/vagal tone.[34,98,99] 4) Psychosocial factors are measured with psychological screening questionnaires for perceived injustice (IEQ), catastrophizing (PCS) and psychological distress (BSI). These psychosocial factors have shown a consistent effect on functioning and on symptom beliefs and pain behaviours, which are common in patients with chronic pain, including CS syndromes. Therefore, although not necessary for the clinical classification of CS, these factors are included in the study and potentially in the analyses. Some of the CS assessment instruments used in this study are more acknowledged than others in CS literature. CSI is widely used to measure CS since it was designed as a proxy for it; however, its internal consistency and reliability in patients with CLBP has not yet been made available. Also, evidence is currently piling up on the necessity of routinely implementing the elements of QST relevant to patients' conditions in clinical practice, given its ability to assess the somatosensory function,[97,100] even though evidence in patients with CLBP is limited. Finally, literature on HRV as an instrument that may reflect an imbalance of ANS in patients with chronic pain conditions (specifically in those patients that have shown an overlap in the CS symptoms) is scarce.[101] We are confident that the selected CS assessment instruments are considered the best available and combined will be able to comprehensively measure from the different reference clinical symptoms and signs indicative of CS being present. This will provide further insights on the mechanisms of CS and assist on the phenotyping of centrally sensitised patients with CLBP.

For peer review only

Various functioning assessments are selected for the research protocol to determine the functioning status of centrally sensitised patients with CLBP, including several objective monitoring and capacity tests, and self-reported measurements. 1) The floor-to-waist lifting capacity test is a physical assessment from the FCE that has demonstrated the best predictability for Return-to-Work in CLBP.[102] 2) The aerobic capacity, expressed as VO2max, can be best assessed with a maximal aerobic capacity test such as the CPET. The cycle ergometer and the treadmill are the most used assessments for aerobic capacity, but the cycle ergometer is most appropriate for patients.[37] 3) The PA is measured by an accelerometer, an easy wearable activity-monitoring device that provides objective measurements of PA levels and PA distribution or patterns. 4) Patient-reported functioning questionnaires show patients' pain experience, how they perceive or believe their functioning and participation is. The WAS, PDI and Rand36-PF show the advantage of being of short length and easy to interpret, due to their interval scale. Although questionnaires results can differ from their actual physical performance, both measurements complement each other. Furthermore, together they may provide a more comprehensive overview of patients with CLBP.[103-105] Assessments such as lifting capacity and self-reported measurements have been routinely used to determine the functioning status of patients with CLBP. CPET, on the other hand, despite being scientifically superior to a submaximal aerobic capacity test, has barely been implemented with patients with CLBP. As a result, it is unknown whether a maximal CPET could be applied systematically with these patients and their pain response to it. Moreover, even if there is a large amount of literature on PA, the methodology implemented is very broad regarding population groups and/or measuring instruments, which hinders the synthesis of the understanding of PA of patients with CLBP. To bridge the gap, it may be of use an objective measurement of PA and monitored with accelerometers such as those of ActiGraph, which has shown to produce the most reliable devices for recording PA in free-living conditions.[106,107] It is believed that the assessments described are suitable and relevant for the purpose of measuring functioning from multiple domains; and that the extended knowledge and the better understanding of the mechanisms involved obtained is not limited to CLBP but can be extended to CS.

Data collected for this project is large and the measurements of CS and functioning collected involve a broad amount of variables. As a result, proxies will be used for the statistical analyses. Previous research in CS assessments, on the one hand, and in functioning assessments, on the other, have reported different methodologies and outcome parameters. Reason for which, even if authors have selected the proxy variables based on their relevance and/or contribution to the purposes of the current studies, the selection of the following measurements as proxies may not be beyond debate: for HRV the RMSSD, as it is a measurement of the parasympathetic/vagal tone which is not influenced by breathing;[108] for the maximal aerobic capacity the VO2max corrected for the body mass of the individual, due to the effect of body

For peer review only - http://bmjopen.bmj.com/site/about/guidelines.xhtml

mass on peak oxygen uptake; and for PA the daily VM counts, because it characterises movement duration and intensity.[109]

There are circumstances that might have influenced the results, in spite of the team's efforts to obtain the most extensive insights of CS and its association with functioning. To begin with, it should be considered that patients participating in the study are volunteers. Moreover, the exclusion criteria, despite being based on patients' files, are also dependent on doctor's judgement. The impact of these circumstances is so far unknown to the authors. Likewise, given the CAU design of the study the choice of measurements have to be feasible and compatible with the regular clinical procedures. As a consequence, the fMRI, a commonly mentioned instrument to measure CS, could not be performed in the study. The HRV on the other hand, although less often used, can be an insightful and innovative approach to the assessment of one of the indicators of CS. Additionally, the sample sizes are estimated a priori for the analyses due to insufficient previous studies and, therefore, they are of exploratory nature and used as a guide. Also, despite the efforts of the team to promote the study participation and participant retention, it is likely that patient recruitment may be slower than anticipated and that patient may not have a complete follow-up. However, since the measurement period is ample, the calculations are vigilantly made with generous margins (see Methods section, Sample size), and the literature related to the topic has shown smaller sample sizes.[25,28,110] We remain positive about the sample size and expect to have more than enough participants to perform analyses with sufficient power. Also, for comparability purposes, measurements are planned to take place within three weeks from the Pain Rehabilitation team's baseline and discharge assessments. The CSI, PDI and psychological screening questionnaires during baseline assessment are sent to be filled six weeks before the rest of the measurements are obtained, a time distance due to waiting list time. Measurements are expected to be comparable to the rest of measurements at baseline, since no important changes are foreseen in patients regarding medication, surgery or personal conditions during this time, and questionnaires have shown to have good clinimetric properties. As opposed to the limitations presented, given the large amount of correlations in the multiple correlation analyses and the risk of Type I error, a more strict significance level was applied, i.e. 0.01. Also, for the QST the proxy will be selected per functioning outcome to obtain the most informative somatosensory measurement for each of the functioning measurements. We are confident that the results obtained will provide meaningful and valuable information.

All things considered, the results obtained from the measurements described in this research protocol can, firstly, help to better describe and understand the mechanisms involved in CS, particularly the ones affecting the functioning of patients with CLBP; and secondly, be used to optimise the effectiveness of pain management interventions. To the authors' knowledge, the study described in this research protocol are the first of its kind and it may become an

For peer review only

important breakthrough for researchers involved in the study of pain, for the treatment strategy of patients with chronic pain by healthcare professionals, and for patient's pain education.

ETHICS AND DISSEMINATION

All participating patients sign the informed consent before any assessment takes place. Formal ethical clearance to perform the study has been obtained from the METC University Medical Center Groningen (The Netherlands) in July 2017 (METC 2016/702). All procedures are in accordance with the ethical standards of the Helsinki Declaration of 1975 as revised in 2014 [111] and with Dutch regulations of the Medical Research Involving Human Subjects Act, in Dutch: Wet Medisch-Wetenschappelijk Onderzoek (WMO), amended in 2018.[112]

The dissemination strategy of the research study results will be through scientific publications in peer-reviewed journals and presentations in relevant national and international conferences. In addition to it, reports will be sent by post to participants, and reports and presentations in meetings will be provided to stakeholders.

For peer review only

ABBREVIATIONS

ANS	Autonomic Nervous System
BF	Breathing Frequency at the end of CPET
BMI	Body Mass Index
BSI	Brief Symptom Inventory
CAU	Care As Usual
cCS	cold Conditioning Stimulus
CLBP	Chronic Low Back Pain
CPET	Cardiopulmonary Exercise Test
CPM	Conditioned Pain Modulation
CS	Central Sensitisation
CSI	Central Sensitisation Inventory
CSS	Central Sensitisation Syndromes
CvR-UMCG	Center for Rehabilitation of the UMCG; in Dutch: Centrum voor Revalidatie
CR-10 scale	Category Ratio (0-10) scale
DMA	Dynamic Mechanical Allodynia
DFNS	German Research Network on Neuropathic Pain; in German: Deutschen Forschungsverbund Neuropathischer Schmerz
DOT	Dictionary of Occupational Titles
ECG	Electrocardiogram
FCE	Functional Capacity Evaluation
HF	High Frequency
HR	Heart Rate
HRV	Heart Rate Variability
IC	Informed Consent
ICC	Intra-Class Correlation
IEQ	Injustice Experience Questionnaire
LF	Low Frequency
MDT	Mechanical Detection Threshold
MET	Metabolic Equivalent of Task
METc	Medical Research Ethics committee; in Dutch: Medisch Ethische Toetsing commissie
MPT	Mechanical Pain Threshold
NASQ	Nijmegen-Aalborg Screening QST
NRS	Numeric Rating Scale
PA	Physical Activity
PCS	Pain Catastrophizing Scale
PDI	Pain Disability Index
PF	Physical Functioning
PPT	Pressure Pain Threshold
PRQ	Pain Response Questionnaire

For peer review only

QST	Quantitative Sensory Testing	VAS	Visual Analogue Scale
Rand36-PF	Physical Functioning subscale of the Rand36 questionnaire	VLF	Very Low Frequency
RER	Respiratory Exchange Ratio	VO2max	Peak VO2, Peak oxygen uptake
RMSSD	Root Mean Square of Successive Differences between NN intervals	VT	Expiratory Tidal Volume
RPE	Rating of Perceived Exertion	WAI	Work Ability Index
RR interval	Inter-beat interval	WAS	Work Ability Score
SDNN	Standard Deviation of Normal-to-Normal (NN) intervals	WMO	Medical Research Involving Human Subjects Act; in Dutch: Wet Medisch-wetenschappelijk Onderzoek met Mensen
TS	Test Stimulus	WUR	Wind-Up Ratio
UMCG	Universitair Medisch Centrum Groningen	YLD	Years Lived with Disability

For peer review only

STATEMENTS

Author's Contributions: JAE was responsible for the study concept and design, data collection, and manuscript preparation and edit; HRSP, RD, and MR were responsible for the study concept and design, and critical review of the manuscript; IS, HT and APW were responsible for the study design and critical review of the manuscript.

Competing interests: The authors declare that there are no conflicts of interest.

Funding: The Pain Rehabilitation Development Center (in Dutch: Ontwikkelcentrum PijnRevalidatie (OPR)) of the CvR-UMCG funded the research study.

Trial registration: NTR7167/NL6980, April 16th 2018; retrospectively registered.

Ethics approval: The Medical Research Ethics Committee of the University Medical Center Groningen (METc UMCG) provided a positive reaction in July 2017 to the development of the research study "*Central Sensitization and Functioning in patients with Chronic Low Back Pain*" (METc 2016/702). Its first amendment was approved in October 2017 and its second amendment in August 2018.

For peer review only

REFERENCES

1. The Joint Task Force (JTF). ICD-11 Beta Draft - Mortality and Morbidity Statistics[Internet]. Available from: https://icd.who.int/browse11/l-m/en#/ (Accessed 24 May 2018).
2. Breivik H, Collett B, Ventafridda V, et al. Survey of chronic pain in Europe: Prevalence, impact on daily life, and treatment. Eur J Pain. 2006;10(4):287–333.
3. Hoy D, March L, Brooks P, et al. The global burden of low back pain: estimates from the Global Burden of Disease 2010 study. Ann Rheum Dis. 2014;73(6):968–74.
4. Vos T, Flaxman AD, Naghavi M, et al. Years lived with disability (YLDs) for 1160 sequelae of 289 diseases and injuries 1990–2010: A systematic analysis for the Global Burden of Disease Study 2010. Lancet. 2012;380(9859):2163–96.
5. Foster NE, Anema JR, Cherkin D, et al. Prevention and treatment of low back pain: evidence, challenges, and promising directions. Lancet. 2018;391(10137):2368–83.
6. Boonen A, van den Heuvel R, van Tubergen A, et al. Large differences in cost of illness and wellbeing between patients with fibromyalgia, chronic low back pain, or ankylosing spondylitis. Ann Rheum Dis. 2005;64:396–402.
7. Dagenais S, Caro J, Haldeman S. A systematic review of low back pain cost of illness studies in the United States and internationally. Spine J. 2008;8(1):8–20.
8. Lambeek LC, van Tulder MW, Swinkels ICS, et al. The Trend in Total Cost of Back Pain in the Netherlands in the Period 2002 to 2007. Spine. 2011;36(13):1050–8.
9. Giesecke T, Gracely RH, Grant MAB, et al. Evidence of augmented central pain processing in idiopathic chronic low back pain. Arthritis Rheum. 2004;50(2):613–23.
10. Woolf CJ. Central sensitization: implications for the diagnosis and treatment of pain. Pain. 2011;152(3 Suppl.):S2–15.
11. Arendt-Nielsen L, Morlion B, Perrot S, et al. Assessment and manifestation of central sensitisation across different chronic pain conditions. Eur J Pain. 2018;22(2):216–41.
12. den Boer C, Dries L, Terluin B, et al. Central sensitization in chronic pain and medically unexplained symptom research: A systematic review of definitions, operationalizations and measurement instruments. J Psychosom Res. 2019;117:32–40.
13. Nijs J, Torres-Cueco R, van Wilgen P, et al. Applying Modern Pain Neuroscience in Clinical Practice: Criteria for the Classification of Central Sensitization Pain. Pain Physician. 2014;17(5):447–57.

For peer review only

14. Smart KM, Blake C, Staines A, et al. The Discriminative Validity of “Nociceptive,” “Peripheral Neuropathic,” and “Central Sensitization” as Mechanisms-based Classifications of Musculoskeletal Pain. Clin J Pain. 2011;27(8):655–63.

15. De Couck M, Nijs J, Gidron Y. You May Need a Nerve to Treat Pain: The Neurobiological Rationale for Vagal Nerve Activation in Pain Management. Clin J Pain. 2014 ;30(12):1099–105.

16. Smart KM, Blake C, Staines A, et al. Mechanisms-based classifications of musculoskeletal pain: Part 1 of 3: Symptoms and signs of central sensitisation in patients with low back (±leg) pain. Man Ther. 2012; 17(4), 336–44.

17. O’Brien T, Breivik H. The impact of chronic pain — European patients’ perspective over 12 months. Scand J Pain. 2012;3(1):23–9.

18. Nugraha B, Gutenbrunner C, Barke A, et al. The IASP classification of chronic pain for ICD-11. Pain. 2019;160(1):88–94.

19. Cieza A, Stucki G, Weigl M, et al. ICF Core Sets for low back pain. J Rehabil Med. 2004; Suppl. 44:69–74.

20. Schneidert M, Hurst R, Miller J, et al. The role of Environment in the International Classification of Functioning, Disability and Health (ICF). Disabil Rehabil. 2003;25(11–12):588–95.

21. Smeets RJEM, Wittink H, Hidding A, et al. Do patients with chronic low back pain have a lower level of aerobic fitness than healthy controls?: are pain, disability, fear of injury, working status, or level of leisure time activity associated with the difference in aerobic fitness level?. Spine. 2006;31(1):90-7.

22. Ryan CG, Grant PM, Dall PM, et al. Individuals with chronic low back pain have a lower level, and an altered pattern, of physical activity compared with matched controls: An observational study. Aust J Physiother. 2009;55(1):53–8.

23. Duque I, Parra JH, Duvallet A. Maximal aerobic power in patients with chronic low back pain: a comparison with healthy subjects. European spine journal. 2011;20(1):87-93.

24. Kregel J, Schumacher C, Dolphens M, et al. Convergent validity of the dutch central sensitization inventory: associations with psychophysical pain measures, quality of life, disability, and pain cognitions in patients with chronic spinal pain. Pain Practice. 2018;18(6):777-87.

25. Huysmans E, Ickmans K, Van Dyck D, et al. Association Between Symptoms of Central Sensitization and Cognitive Behavioral Factors in People With Chronic Nonspecific Low Back Pain: A Cross-sectional Study. J Manipulative Physiol Ther. 2018;41(2):92-101.

26. Tanaka K, Murata S, Nishigami T, et al. The Central Sensitization Inventory predict pain-related disability for

For peer review only

musculoskeletal disorders in the primary care setting. Eur J Pain. 2019; 23(9):1640-8.

27. Cohen H, Neumann L, Shore M, et al. Buskila. Autonomic dysfunction in patients with fibromyalgia: application of power spectral analysis of heart rate variability. Semin Arthritis Rheum. 2000;29(4):217-27.

28. Gockel M, Lindholm H, Niemistö L, et al. Perceived disability but not pain is connected with autonomic nervous function among patients with chronic low back pain. J Rehabil Med. 2008;40(5):355-8.

29. Hübscher M, Moloney N, Leaver A, et al. Relationship between quantitative sensory testing and pain or disability in people with spinal pain—A systematic review and meta-analysis. Pain. 2013; 154(9), 1497–504.

30. Georgopoulos V, Akin-Akinyosoye K, Zhang W, et al. Quantitative sensory testing and predicting outcomes for musculoskeletal pain, disability, and negative affect. Pain. 2019;160(9):1920-32.

31. Mayer TG, Neblett R, Cohen H, et al. The development and psychometric validation of the central sensitization inventory. Pain Pract. 2012;12(4):276–85.

32. Rolke R, Baron R, Maier C, et al. Quantitative sensory testing in the German Research Network on Neuropathic Pain (DFNS): Standardized protocol and reference values. Pain. 2006;123(3):231–43.

33. Wilder-Smith O. A paradigm-shift in pain medicine: implementing a systematic approach to altered pain processing in everyday clinical practice based on quantitative sensory testing. Center for Sensory-Motor Interaction (SMI), Department of Health Science and Technology, Aalborg University. 2013:76p.

34. McCraty R, Atkinson M, Tomasino D, et al. The coherent heart: heartbrain interactions, psychophysiological coherence, and the emergence of system-wide order. Boulder Creek, CA: Institute of Heartmath; 2009.

35. IASP Taskforce for the Classification of Chronic Pain. The IASP classification of chronic pain for ICD-11: chronic primary pain. Pain. 2019; 160(1): 28-37.

36. Hart DL, Isernhagen SJ, Matheson LN. Guidelines for Functional Capacity Evaluation of People With Medical Conditions. J Orthop Sport Phys Ther. 1993;18(6):682–6.

37. American Thoracic Society, American College of Chest Physicians. ATS/ACCP Statement on Cardiopulmonary Exercise Testing. Am J Respir Crit Care Med. 2003;167(2):211–77.

38. Neblett R, Cohen H, Choi Y, et al. The Central Sensitization Inventory (CSI): establishing clinically significant values for identifying central sensitivity syndromes in an outpatient chronic pain sample. J Pain. 2013;14(5):438–45.

39. Neblett R, Hartzell MM, Mayer TG, et al. Establishing Clinically Relevant Severity Levels for the Central Sensitization Inventory. Pain Pract. 2017;17(2):166–75.

40. Scerbo T, Colasurdo J, Dunn S, et al. Measurement Properties of the Central Sensitization Inventory: A

For peer review only

Systematic Review. Pain Pract. 2018;18(4):544–54.

41. van Wilgen CP, Meeus M, Descheemaeker F, et al. Central Sensitization Inventory – Nederlandse consensusvertaling [Internet]. 2013. Available from: http://www.paininmotion.be/storage/app/media//materials/CSI-consensusvertaling.pdf (Accessed 19 Jul 2018).
42. Kregel J, Vuijk PJ, Descheemaeker F, et al. The Dutch Central Sensitization Inventory (CSI). Clin J Pain. 2016;32(7):624–30.
43. Timmerman H, Wilder-Smith O, van Weel C, et al. Detecting the neuropathic pain component in the clinical setting: a study protocol for validation of screening instruments for the presence of a neuropathic pain component. BMC Neurol. 2014;14:94–102.
44. Geber C, Klein T, Azad S, et al. Test–retest and interobserver reliability of quantitative sensory testing according to the protocol of the German Research Network on Neuropathic Pain (DFNS): A multi-centre study. Pain. 2011;152(3):548–56.
45. LaMotte RH, Shain CN, Simone DA, et al. Neurogenic hyperalgesia: psychophysical studies of underlying mechanisms. J Neurophysiol. 1991;66(1):190–211.
46. Ziegler EA, Magerl W, Meyer RA, et al. Secondary hyperalgesia to punctate mechanical stimuli. Brain. 1999;122(12):2245–57.
47. Chan AW, MacFarlane IA, Bowsher D, et al. Weighted needle pinprick sensory thresholds: a simple test of sensory function in diabetic peripheral neuropathy. J Neurol Neurosurg Psychiatry. 1992;55(1):56–9.
48. Greenspan JD, McGillis SLB. Thresholds for the Perception of Pressure, Sharpness, and Mechanically Evoked Cutaneous Pain: Effects of Laterality and Repeated Testing. Somatosens Mot Res. 1994;11(4):311–7.
49. Hampf G, Bowsher D, Wells C, et al. Sensory and autonomic measurements in idiopathic trigeminal neuralgia before and after radiofrequency thermocoagulation: differentiation from some other causes of facial pain. Pain. 1990;40(3):241–8.
50. Price DD, Hu JW, Dubner R, et al. Peripheral suppression of first pain and central summation of second pain evoked by noxious heat pulses. Pain. 1977;3(1):57–68.
51. Magerl W, Wilk SH, Treede R-D. Secondary hyperalgesia and perceptual wind-up following intradermal injection of capsaicin in humans. Pain. 1998;74(2–3):257–68.
52. Weinstein S. Intensive and extensive aspects of tactile sensitivity as a function of body part, sex and laterality. In: Kenshalo DR, ed. The skin senses: Proceedings. Springfield, IL, US: Charles C Thomas. 1968:195–222.

For peer review only

53. Fruhstorfer H, Gross W, Selbmann O. von Frey hairs: new materials for a new design. Eur J Pain. 2001;5(3):341–2.

54. Baumgärtner U, Magerl W, Klein T, et al. Neurogenic hyperalgesia versus painful hypoalgesia: two distinct mechanisms of neuropathic pain. Pain. 2002;96(1–2):141–51.

55. Yarnitsky D, Bouhassira D, Drewes AM, et al. Recommendations on practice of conditioned pain modulation (CPM) testing. Eur J Pain. 2015;19(6):805–6.

56. Imai Y, Petersen KK, Mørch CD, et al. Comparing test–retest reliability and magnitude of conditioned pain modulation using different combinations of test and conditioning stimuli. Somatosens Mot Res. 2016;33(3–4):169–77.

57. McCorry LK. Physiology of the autonomic nervous system. Am J Pharm Educ. 2007;71(4):78–88.

58. Task Force of the European Society of Cardiology and the North American Society of Pacing and Electrophysiology. Heart rate variability, standards of measurement, physiological interpretation, and clinical use. Circulation. 1996;93(5):1043–65.

59. McCraty R, Shaffer F. Heart Rate Variability: New Perspectives on Physiological Mechanisms, Assessment of Self-regulatory Capacity, and Health Risk. Glob Adv Heal Med. 2015;4(1):46–61.

60. HeartMath Inc. emWave Pro Plus Assessments [Internet]. 2018. Available from: https://heartcloud.com/library/asmts.html (Accessed 27 Feb 2019).

61. Isernhagen SJ, Hart DL, Matheson LM. Reliability of independent observer judgments of level of lift effort in a kinesiophysical Functional Capacity Evaluation. Work. 1999;12(2):145–50.

62. de Baets S, Calders P, Schalley N, et al. Updating the Evidence on Functional Capacity Evaluation Methods: A Systematic Review. J Occup Rehabil. 2018;28(3):418–28.

63. Brouwer S, Reneman MF, Dijkstra PU, et al. Test–Retest Reliability of the Isernhagen Work Systems Functional Capacity Evaluation in Patients with Chronic Low Back Pain. J Occup Rehabil. 2003;13(4):207–18.

64. Soer R, van der Schans CP, Geertzen JH, et al. Normative Values for a Functional Capacity Evaluation. Arch Phys Med Rehabil. 2009;90(10):1785–94.

65. Borg G. Borg's perceived exertion and pain scales.. Champaign, IL, US: Human Kinetics; 1998.

66. Reneman MF, Fokkens AS, Dijkstra PU, et al. Testing Lifting Capacity: Validity of Determining Effort Level by Means of Observation. Spine. 2005;30(2):e40–6.

67. Scherr J, Wolfarth B, Christle JW, et al. Associations between Borg's rating of perceived exertion and

For peer review only

physiological measures of exercise intensity. Eur J Appl Physiol. 2013;113(1):147–55.

68. Trost SG, McIver KL, Pate RR. Conducting Accelerometer-Based Activity Assessments in Field-Based Research. Med Sci Sports Exerc. 2005;37(11 Suppl.):S531–43.
69. Migueles JH, Cadenas-Sanchez C, Ekelund U, et al. Accelerometer Data Collection and Processing Criteria to Assess Physical Activity and Other Outcomes: A Systematic Review and Practical Considerations. Sport Med. 2017;47(9):1821–45.
70. Keadle SK, Shiroma EJ, Freedson PS, et al. Impact of accelerometer data processing decisions on the sample size, wear time and physical activity level of a large cohort study. BMC Public Health. 2014;14(1):1210.
71. Sasaki JE, John D, Freedson PS. Validation and comparison of ActiGraph activity monitors. J Sci Med Sport. 2011;14(5):411–6.
72. Crouter SE, Kuffel E, Haas JD, et al. Refined two-regression model for the ActiGraph accelerometer. Med Sci Sports Exerc. 2010;42(5):1029–37.
73. van der Zee KI, Sanderman R. RAND-36. umcg.nl [Internet]. Available from: https://www.umcg.nl/SiteCollectionDocuments/research/institutes/SHARE/assessment tools/handleiding_rand36_2e_druk.pdf (Accessed 28 Jun 2018).
74. van der Zee KI, Sanderman R, Heyink JW, et al. Psychometric qualities of the rand 36-item health survey 1.0: A multidimensional measure of general health status. Int J Behav Med. 1996;3(2):104–22.
75. Tait RC, Chibnall JT, Krause S. The Pain Disability Index: psychometric properties. Pain. 1990;40(2):171–82.
76. Soer R, Köke AJA, Vroomen PCAJ, et al. Extensive Validation of the Pain Disability Index in 3 Groups of Patients With Musculoskeletal Pain. Spine. 2013;38(9):e562–8.
77. de Zwart BCH, Frings-Dresen MHW, van Duivenbooden JC. Test-retest reliability of the Work Ability Index questionnaire. Occup Med. 2002;52(4):177–81.
78. Ahlstrom L, Grimby-Ekman A, Hagberg M, et al. The work ability index and single-item question: associations with sick leave, symptoms, and health – A prospective study of women on long-term sick leave. Scand J Work Environ Heal. 2010;36(5):404–12.
79. El Fassi M, Bocquet V, Majery N, et al. Work ability assessment in a worker population: comparison and determinants of Work Ability Index and Work Ability score. BMC Public Health. 2013;13:305–14.
80. U.S. Department of Labor, Employment and Training Administration. Dictionary of Occupational Titles. 4th ed. Washington; 1991.

For peer review only

81. Hawker GA, Mian S, Kendzerska T, et al. Measures of adult pain: Visual Analog Scale for Pain (VAS Pain), Numeric Rating Scale for Pain (NRS Pain), McGill Pain Questionnaire (MPQ), Short-Form McGill Pain Questionnaire (SF-MPQ), Chronic Pain Grade Scale (CPGS), Short Form-36 Bodily Pain Scale (SF36 BPS), and Measure of Intermittent and Constant Osteoarthritis Pain (ICOAP). Arthritis Care Res. 2011;63(S11):S240–52.

82. Soer R, Groothoff JW, Geertzen JHB, et al. Pain Response of Healthy Workers Following a Functional Capacity Evaluation and Implications for Clinical Interpretation. J Occup Rehabil. 2008;18(3):290–8.

83. Adams LM, Turk DC. Psychosocial Factors and Central Sensitivity Syndromes. Curr Rheumatol Rev. 2015 ; 11(2): 96–108.

84. Sullivan MJL, Bishop SR, Pivik J. The pain catastrophizing scale: development and validation. Psychol Assess. 1995;7(4):524–32.

85. Crombez G, Vlaeyen JWS. Pain Catastrophizing Scale (PCS) - Dutch Version (PCS-DV) [Internet]. 1996. Available from: https://meetinstrumentenzorg.blob.core.windows.net/test-documents/Instrument197/197_3_N.pdf (Accessed 8 Aug 2018).

86. van Damme S, Crombez G, Vlaeyen JWS, et al. De Pain Catastrophizing Scale: Psychometrische karakteristieken en normering. Gedragstherapie. 2000;33:209–20.

87. Rodero B, Luciano J V, Montero-Marín J, et al. Perceived injustice in fibromyalgia: Psychometric characteristics of the Injustice Experience Questionnaire and relationship with pain catastrophising and pain acceptance. J Psychosom Res. 2012;73(2):86–91.

88. van Wilgen CP, Nijs J, Don S, et al. The Injustice Experience Questionnaire: Nederlandstalige consensusvertaling [Internet]. 2014. Available from: http://www.paininmotion.be/storage/app/media//materials/IEQ-Dutch.pdf (Accessed 19 Jul 2018).

89. Derogatis LR. BSI brief symptom inventory: Administration, Scoring, and Procedure Manual (4th Ed.). Minneapolis, MN: National Computer Systems;1993.

90. de Beurs E, Zitman FG. The Brief Symptom Inventory (BSI): Reliability and validity of a practical alternative to SCL-90. Maandbl Geestelijke Volksgezond. 2006;61:120–41.

91. van Ginkel JR, Linting M, Rippe RC, et al. Rebutting Existing Misconceptions About Multiple Imputation as a Method for Handling Missing Data. J Pers Assess. 2019; 1-12.

92. van Wilgen CP, Vuijk PJ, Kregel J, et al. Psychological Distress and Widespread Pain Contribute to the Variance of the Central Sensitization Inventory: A Cross-Sectional Study in Patients with Chronic Pain. Pain

For peer review only

Pract. 2018;18(2):239–46.

93. Arendt-Nielsen L, Yarnitsky D. Experimental and Clinical Applications of Quantitative Sensory Testing Applied to Skin, Muscles and Viscera. J Pain. 2009;10(6):556–72.

94. Backonja M-M, Walk D, Edwards RR, et al. Quantitative Sensory Testing in Measurement of Neuropathic Pain Phenomena and Other Sensory Abnormalities. Clin J Pain. 2009;25(7):641–7.

95. Pavlaković G, Petzke F. The Role of Quantitative Sensory Testing in the Evaluation of Musculoskeletal Pain Conditions. Curr Rheumatol Rep. 2010;12(6):455–61.

96. Krumova EK, Geber C, Westermann A, et al. Neuropathic Pain: Is Quantitative Sensory Testing Helpful? Curr Diab Rep. 2012;12(4):393–402.

97. Uddin Z, MacDermid JC. Quantitative Sensory Testing in Chronic Musculoskeletal Pain. Pain Med. 2016;17(9):1694–703.

98. Martínez-Martínez L-A, Mora T, Vargas A, et al. Sympathetic Nervous System Dysfunction in Fibromyalgia, Chronic Fatigue Syndrome, Irritable Bowel Syndrome, and Interstitial Cystitis. JCR J Clin Rheumatol. 2014;20(3):146–50.

99. Tracy LM, Ioannou L, Baker KS, et al. Meta-analytic evidence for decreased heart rate variability in chronic pain implicating parasympathetic nervous system dysregulation. Pain. 2016;157(1):7–29.

100. Starkweather AR, Heineman A, Storey S, et al. Methods to measure peripheral and central sensitization using quantitative sensory testing: A focus on individuals with low back pain. Appl Nurs Res. 2016;29:237–41.

101. Koenig J, Falvay D, Clamor A, et al. Pneumogastric (Vagus) Nerve Activity Indexed by Heart Rate Variability in Chronic Pain Patients Compared to Healthy Controls: A Systematic Review and Meta-Analysis. Pain Physician. 2016;19(1):E55-78.

102. Kuijer PPFM, Gouttebarge V, Brouwer S, et al. Are performance-based measures predictive of work participation in patients with musculoskeletal disorders? A systematic review. Int Arch Occup Environ Health. 2012;85(2):109–23.

103. Reneman MF, Jorritsma W, Schellekens JMH, et al. Concurrent validity of questionnaire and performance-based disability measurements in patients with chronic nonspecific low back pain. J Occup Rehabil. 2002;12(3):119–29.

104. Brouwer S, Dijkstra PU, Stewart RE, et al. Comparing self-report, clinical examination and functional testing in the assessment of work-related limitations in patients with chronic low back pain. Disabil Rehabil. 2005;27(17):999–1005.

For peer review only

105. Caporaso F, Pulkovski N, Sprott H, et al. How well do observed functional limitations explain the variance in Roland Morris scores in patients with chronic non-specific low back pain undergoing physiotherapy? Eur Spine J. 2012;21(Suppl 2):S187–95.

106. Vanhelst J, Gottrand F, Baquet G, et al. Comparative Interinstrument Reliability of Uniaxial and Triaxial Accelerometers in Free-Living Conditions. Percept Mot Skills. 2012;114(2):584–94.

107. Aadland E, Ylvisåker E. Reliability of the Actigraph GT3X+ Accelerometer in Adults under Free-Living Conditions. PLoS One. 2015;10(8):e0134606.

108. Laborde S, Mosley E, Thayer JF. Heart Rate Variability and Cardiac Vagal Tone in Psychophysiological Research – Recommendations for Experiment Planning, Data Analysis, and Data Reporting. Front Psychol. 2017;08:213.

109. Matthews CE, Hagströmer M, Pober DM, et al. Best practices for using physical activity monitors in population-based research. Med Sci Sports Exerc. 2012;44(Suppl 1):S68-76.

110. Clauw DJ, Williams D, Lauerman W, et al. Pain Sensitivity as a Correlate of Clinical Status in Individuals With Chronic Low Back Pain. Spine. 1999; 24(19), 2035-41.

111. General Assembly of the World Medical Association. World Medical Association Declaration of Helsinki: Ethical principles for medical research involving human subjects. J Am Coll Dent. 2014;81(3):14–8.

112. Dutch Government. The Medical Research Involving Human Subjects Act, in Dutch: Wet medisch-wetenschappelijk onderzoek met mensen (WMO) [Internet]. Available from: https://wetten.overheid.nl/BWBR0009408/2018-08-01 (Accessed 28 Feb 2019).

For peer review only

FIGURE LEGENDS

Figure 1. Flow-chart of the project procedure.

[*] , measurements obtained which do not belong to CAU of the Pain Rehabilitation of the CvR – UMCG.

For peer review only

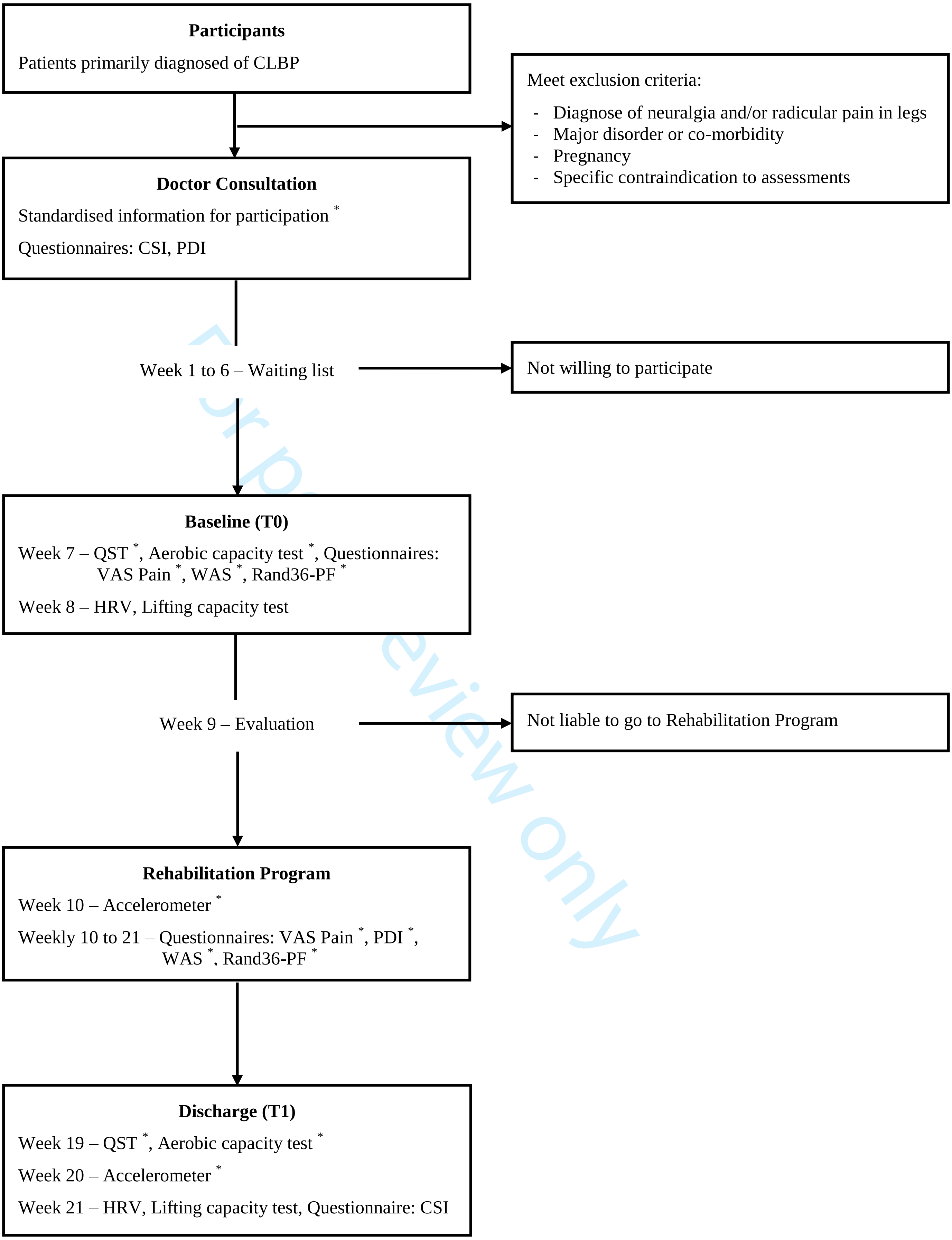

For peer review only

SUPPLEMENTARY FILE

Supplementary material 1. Description of the locations for the QST assessment.

Measuring Location	Description
**1. m. Deltoid – Training location**	In the pars medialis of m. Deltoid, at the contralateral side of the body to the most painful area located; 4 cm below the Acromion.<br>For the CPM test the measurement is at the non-dominant m. Deltoid.
**2. Most painful area**	As stated by the patient. This location is thoroughly described in the form to repeat the measurement in the same exact location at discharge.
**3. Low back – Control location**	Contralateral side of the body mirror-wise to the most painful area located. A specific description of the most painful area is important to identify this location as well.
**4. m. Trapezius – Control location**	In the pars medialis of m. Trapezius, both ipsilateral and contralateral to the most painful area located. At the level of the spine of the Scapula (T3); 4 cm lateral / external to the backbone.
**5. m. Quadriceps – Control location**	In the rectus femoris of m. Quadriceps, both ipsilateral and contralateral to the most painful area located; 15 cm above the upper edge of the Patella.<br>For the CPM test the measurement is at the non-dominant m. Quadriceps.

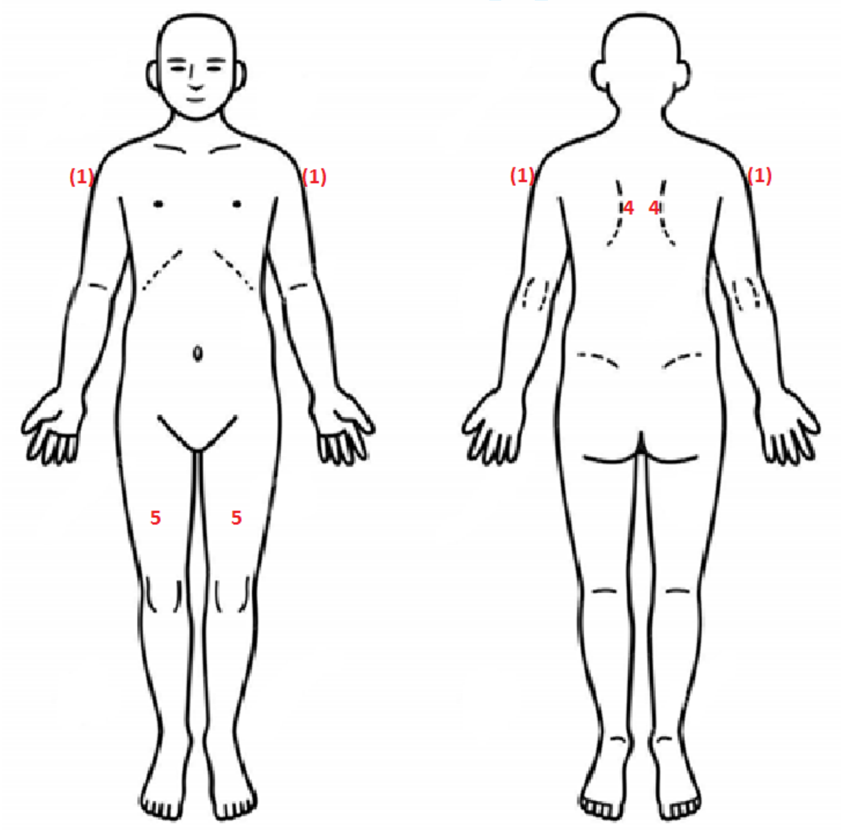

For peer review only - http://bmjopen.bmj.com/site/about/guidelines.xhtml

SPIRIT
STANDARD PROTOCOL ITEMS: RECOMMENDATIONS FOR INTERVENTIONAL TRIALS

SPIRIT 2013 Checklist: Recommended items to address in a clinical trial protocol and related documents*

Section / Item	Item No.	Description	Check
**Administrative information**			
Title	1	Descriptive title identifying the study design, population, interventions, and, if applicable, trial acronym	X
Trial registration	2a	Trial identifier and registry name. If not yet registered, name of intended registry	X
	2b	All items from the World Health Organization Trial Registration Data Set	X
Protocol version	3	Date and version identifier	X
Funding	4	Sources and types of financial, material, and other support	X
Roles and responsibilities	5a	Names, affiliations, and roles of protocol contributors	X
	5b	Name and contact information for the trial sponsor	NA
	5c	Role of study sponsor and funders, if any, in study design; collection, management, analysis, and interpretation of data; writing of the report; and the decision to submit the report for publication, including whether they will have ultimate authority over any of these activities	NA
	5d	Composition, roles, and responsibilities of the coordinating centre, steering committee, endpoint adjudication committee, data management team, and other individuals or groups overseeing the trial, if applicable (see Item 21a for data monitoring committee)	NA
**Introduction**			
Background and rationale	6a	Description of research question and justification for undertaking the trial, including summary of relevant studies (published and unpublished) examining benefits and harms for each intervention	X
	6b	Explanation for choice of comparators	X
Objectives	7	Specific objectives or hypotheses	X
Trial design	8	Description of trial design including type of trial (eg, parallel group, crossover, factorial, single group), allocation ratio, and framework (eg, superiority, equivalence, noninferiority, exploratory)	X

For peer review only

Methods: Participants, interventions, and outcomes

Study setting	9	Description of study settings (eg, community clinic, academic hospital) and list of countries where data will be collected. Reference to where list of study sites can be obtained	**X**
Eligibility criteria	10	Inclusion and exclusion criteria for participants. If applicable, eligibility criteria for study centres and individuals who will perform the interventions (eg, surgeons, psychotherapists)	**X**
Interventions	11a	Interventions for each group with sufficient detail to allow replication, including how and when they will be administered	**NA**
	11b	Criteria for discontinuing or modifying allocated interventions for a given trial participant (eg, drug dose change in response to harms, participant request, or improving/worsening disease)	**NA**
	11c	Strategies to improve adherence to intervention protocols, and any procedures for monitoring adherence (eg, drug tablet return, laboratory tests)	**NA**
	11d	Relevant concomitant care and interventions that are permitted or prohibited during the trial	**NA**
Outcomes	12	Primary, secondary, and other outcomes, including the specific measurement variable (eg, systolic blood pressure), analysis metric (eg, change from baseline, final value, time to event), method of aggregation (eg, median, proportion), and time point for each outcome. Explanation of the clinical relevance of chosen efficacy and harm outcomes is strongly recommended	**X**
Participant timeline	13	Time schedule of enrolment, interventions (including any run-ins and washouts), assessments, and visits for participants. A schematic diagram is highly recommended (see Figure)	**X**
Sample size	14	Estimated number of participants needed to achieve study objectives and how it was determined, including clinical and statistical assumptions supporting any sample size calculations	**X**
Recruitment	15	Strategies for achieving adequate participant enrolment to reach target sample size	**X**

Methods: Assignment of interventions (for controlled trials) **NA**

Allocation:			
Sequence generation	16a	Method of generating the allocation sequence (eg, computer-generated random numbers), and list of any factors for stratification. To reduce predictability of a random sequence, details of any planned restriction (eg, blocking) should be provided in a separate document that is unavailable to those who enrol participants or assign interventions	**NA**

For peer review only - http://bmjopen.bmj.com/site/about/guidelines.xhtml

Allocation concealment mechanism	16b	Mechanism of implementing the allocation sequence (eg, central telephone; sequentially numbered, opaque, sealed envelopes), describing any steps to conceal the sequence until interventions are assigned	**NA**
Implementation	16c	Who will generate the allocation sequence, who will enrol participants, and who will assign participants to interventions	**NA**
Blinding (masking)	17a	Who will be blinded after assignment to interventions (eg, trial participants, care providers, outcome assessors, data analysts), and how	**NA**
	17b	If blinded, circumstances under which unblinding is permissible, and procedure for revealing a participant's allocated intervention during the trial	**NA**
**Methods: Data collection, management, and analysis**			
Data collection methods	18a	Plans for assessment and collection of outcome, baseline, and other trial data, including any related processes to promote data quality (eg, duplicate measurements, training of assessors) and a description of study instruments (eg, questionnaires, laboratory tests) along with their reliability and validity, if known. Reference to where data collection forms can be found, if not in the protocol	**X**
	18b	Plans to promote participant retention and complete follow-up, including list of any outcome data to be collected for participants who discontinue or deviate from intervention protocols	**NA**
Data management	19	Plans for data entry, coding, security, and storage, including any related processes to promote data quality (eg, double data entry; range checks for data values). Reference to where details of data management procedures can be found, if not in the protocol	**X**
Statistical methods	20a	Statistical methods for analysing primary and secondary outcomes. Reference to where other details of the statistical analysis plan can be found, if not in the protocol	**X**
	20b	Methods for any additional analyses (eg, subgroup and adjusted analyses)	**X**
	20c	Definition of analysis population relating to protocol non-adherence (eg, as randomised analysis), and any statistical methods to handle missing data (eg, multiple imputation)	**X**
**Methods: Monitoring**			**NA**
Data monitoring	21a	Composition of data monitoring committee (DMC); summary of its role and reporting structure; statement of whether it is independent from the sponsor and competing interests; and reference to where further details about its charter can be found, if not in the protocol. Alternatively, an explanation of why a DMC is not needed	**NA**

	21b	Description of any interim analyses and stopping guidelines, including who will have access to these interim results and make the final decision to terminate the trial	**NA**
Harms	22	Plans for collecting, assessing, reporting, and managing solicited and spontaneously reported adverse events and other unintended effects of trial interventions or trial conduct	**NA**
Auditing	23	Frequency and procedures for auditing trial conduct, if any, and whether the process will be independent from investigators and the sponsor	**NA**
**Ethics and dissemination**			
Research ethics approval	24	Plans for seeking research ethics committee/institutional review board (REC/IRB) approval	**X**
Protocol amendments	25	Plans for communicating important protocol modifications (eg, changes to eligibility criteria, outcomes, analyses) to relevant parties (eg, investigators, REC/IRBs, trial participants, trial registries, journals, regulators)	**X**
Consent or assent	26a	Who will obtain informed consent or assent from potential trial participants or authorised surrogates, and how (see Item 32)	**X**
	26b	Additional consent provisions for collection and use of participant data and biological specimens in ancillary studies, if applicable	**NA**
Confidentiality	27	How personal information about potential and enrolled participants will be collected, shared, and maintained in order to protect confidentiality before, during, and after the trial	**X**
Declaration of interests	28	Financial and other competing interests for principal investigators for the overall trial and each study site	**X**
Access to data	29	Statement of who will have access to the final trial dataset, and disclosure of contractual agreements that limit such access for investigators	**X**
Ancillary and post-trial care	30	Provisions, if any, for ancillary and post-trial care, and for compensation to those who suffer harm from trial participation	**NA**
Dissemination policy	31a	Plans for investigators and sponsor to communicate trial results to participants, healthcare professionals, the public, and other relevant groups (eg, via publication, reporting in results databases, or other data sharing arrangements), including any publication restrictions	**X**
	31b	Authorship eligibility guidelines and any intended use of professional writers	**X**
	31c	Plans, if any, for granting public access to the full protocol, participant-level dataset, and statistical code	**NA**

For peer review only

Appendices

Informed consent materials	32	Model consent form and other related documentation given to participants and authorised surrogates	**-**
Biological specimens	33	Plans for collection, laboratory evaluation, and storage of biological specimens for genetic or molecular analysis in the current trial and for future use in ancillary studies, if applicable	**NA**

*It is strongly recommended that this checklist be read in conjunction with the SPIRIT 2013 Explanation & Elaboration for important clarification on the items. Amendments to the protocol should be tracked and dated. The SPIRIT checklist is copyrighted by the SPIRIT Group under the Creative Commons "Attribution-NonCommercial-NoDerivs 3.0 Unported" license.

For peer review only
